# Monoselective N-Methylation of Amides, Indoles, and Related Structures Using Quaternary Ammonium Salts as Solid Methylating Agents

**DOI:** 10.1021/acs.orglett.2c02766

**Published:** 2022-10-03

**Authors:** Johanna Templ, Edma Gjata, Filippa Getzner, Michael Schnürch

**Affiliations:** Institute of Applied Synthetic Chemistry, TU Wien, Getreidemarkt 9/163, 1060 Wien, Austria

## Abstract

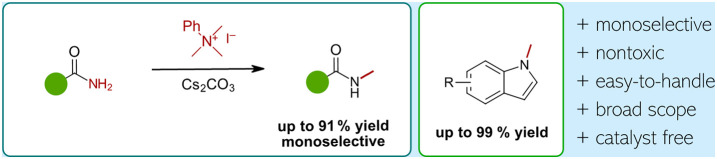

We herein report the use of phenyl trimethylammonium iodide (PhMe_3_NI) as a safe, nontoxic, and easy-to-handle reagent for an absolutely monoselective N-methylation of amides and related compounds as well as for the N-methylation of indoles. In addition, we expanded the method to N-ethylation using PhEt_3_NI. The ease of operational setup, high yields of ≤99%, high functional group tolerance, and especially the excellent monoselectivity for amides make this method attractive for late-stage methylation of bioactive compounds.

Nitrogen-containing compounds are privileged structures in organic chemistry. For example, among thousands of FDA-approved, small molecule drugs, more than 80% contain at least one nitrogen atom with an average of 2.3 nitrogens per drug.^1^ These impressive numbers outline the importance of nitrogen-containing motifs in medicinal chemistry and drug discovery. When checking the top 200 small molecule drugs by retail sales in 2021^2^ (see the bottom of Figure 1 for a selection), one notices the nitrogen atom is found in a majority of pharmaceuticals and herein appears in different structural modifications. Repeatedly occurring nitrogen-containing functionalities include amines, amides, sulfonamides, and N-heterocycles. Simple structural modifications, e.g., alkylation, of such groups often drastically change the physiological and biological properties of pharmaceutically active molecules.^3,4^ Considering alkylation as a late-stage modification in bioactive compounds in general, the simplest and smallest of all alkyl groups, the methyl group, seems to have the most profound impact on altering the biological properties of a molecule.^5−7^ This phenomenon is well-known as the “magic-methyl effect”.^4,8,9^

**Figure 1:**
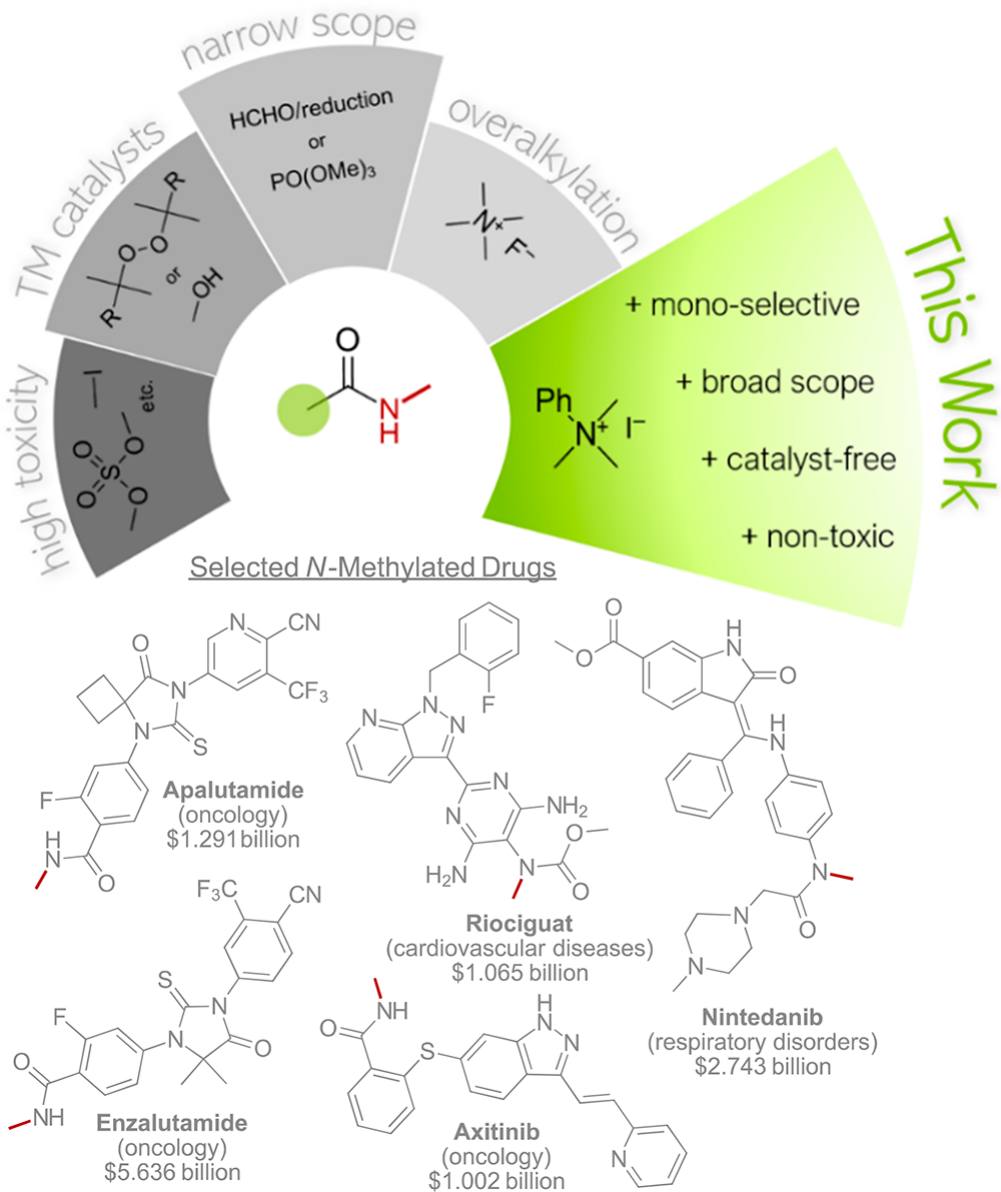
Strategies for the methylation of amides (top) and selected N-methylated small molecule pharmaceuticals and their retail sales in 2021 (bottom).^2^

Hence, new strategies for efficient and selective N-methylation of amides and related structures are of great interest.^10^ However, major challenges with these specific transformations need to be considered (see Figure 1). First, traditionally applied methylating agents, such as iodomethane^11^ or dimethyl sulfate,^12^ often suffer from undesired properties, such as high toxicity, carcinogenicity, and volatility. Some strategies require transition metal catalysts, e.g., when using peroxides^13^ or MeOH^14−16^ as the single-carbon source.

Still others have a relatively narrow substrate scope, which limits the broad application of the respective methylating agent, e.g., when using formaldehyde^17,18^ under reductive conditions or PO(OMe)_3_.^19^ The Schoenebeck group recently reported a safe and metal-free methylation protocol using tetramethylammonium fluoride.^20^ This method is characterized by a relatively broad substrate scope, including amides, N-heterocycles, and alcohols. However, this strategy lacks monoselectivity when methylating primary amides. In general, the tendency of primary amides to undergo overalkylation features the second serious challenge when searching for new N-methylation strategies.

We describe herein a novel, safe, and monoselective protocol for efficient methylation of amides using phenyl trimethylammonium iodide (PhMe_3_NI) as the CH_3_ source under mildly basic conditions, which is characterized by the ease of operational setup. In addition, we demonstrate the broad applicability of this new method by expanding the scope toward N-heterocycles, e.g., indoles, and prove its potential use in the late-stage functionalization of bioactive compounds. Furthermore, the monoselective introduction of an ethyl group can be realized using the related quaternary ammonium salt PhEt_3_NI.

For all optimization screenings, we used 4-fluoro benzyl amide (**1a**) as the substrate. The fluoro substituent enables facile quantification directly from the reaction solution without preceding solvent removal or workup via ^19^F NMR using trifluoro toluene as the internal standard.

We started our investigations by building on our previous results for the selective α-C(sp^3^)-methylation^21^ using PhMe_3_NI as the methylating agent and KOH as the base in toluene at 120 °C (Table 1, entry 1). The mono-N-methylated product was obtained with a moderate yield of 56%. Other hydroxy bases showed significantly lower conversion (entries 2 and 3). Gratifyingly, we found Cs_2_CO_3_ as a mild base giving the mono-N-methylated product (**2a**) in 85% yield (entry 4). All other bases tested turned out to be inefficient (see the Supporting Information for details). Next, we tested different quaternary ammonium salts as the methylating agents. The tetramethylammonium halides gave lower overall yields for **2a**, with a rapid decrease in conversion from the respective fluoride to iodide salts (entries 6–9). Tetramethylammonium fluoride, which was applied for methylating secondary amides toward tertiary ones, gave a 1:1 mixture of mono- and bis-methylated products **2a** and **3a**, respectively (entry 6), and obviously lacks monoselectivity. As anticipated, the phenyl trimethylammonium halides performed best, with the phenyl trimethylammonium iodide outperforming the respective chloride and bromide (entries 4, 10, and 11). We also tested a variety of solvents that are considered to be more environmentally benign such as *t*-BuOH, cyclopentyl methyl ether (CPME),^22^ and anisole.^23^ Indeed, they turned out to be suitable solvents for this specific reaction; however, ∼10–20% lower yields of **2a** were obtained (entries 12–14) compared to those with toluene, which consequently remained the solvent of choice (entry 4).

**Table 1:**
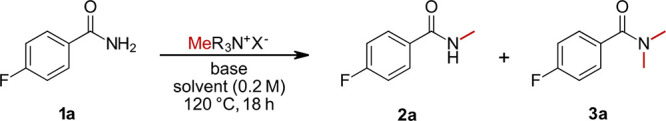
Optimization of the Reaction Conditions^a^

					yield (%)^b^	
entry	solvent	ammonium salt	base	conversion (%)	**2a**	**3a**
1	toluene	PhMe_3_NI	KOH	81	56	19
2	toluene	PhMe_3_NI	NaOH	43	11	7
3	toluene	PhMe_3_NI	LiOH·H_2_O	28	6	0
4	toluene	PhMe_3_NI	Cs_2_CO_3_	91	85	5
5	toluene	PhMe_3_NI	no base	7	0	0
6	toluene	Me_4_NF	Cs_2_CO_3_	97	26	24
7	toluene	Me_4_NCl	Cs_2_CO_3_	73	67	3
8	toluene	Me_4_NBr	Cs_2_CO_3_	31	23	0
9	toluene	Me_4_NI	Cs_2_CO_3_	8	4	0
10	toluene	PhMe_3_NCl	Cs_2_CO_3_	96	78	7
11	toluene	PhMe_3_NBr	Cs_2_CO_3_	99	78	11
12	*t*-BuOH^c^	PhMe_3_NI	Cs_2_CO_3_	79	65	7
13	CPME	PhMe_3_NI	Cs_2_CO_3_	94	74	7
14	anisole	PhMe_3_NI	Cs_2_CO_3_	89	73	5

a Reactions were performed on a 0.35 mmol scale, with 2 equiv of the base and 2 equiv of the ammonium salt under an Ar atmosphere at 120 °C with a reaction time of 18 h.

b Yields were determined by quantitative ^19^F NMR using trifluoro toluene as the internal standard.

c At 100 °C.

In our previous publication on selective α-methylation of aryl ketones,^21^ we could prove that a reaction pathway via thermal decomposition of the methylammonium salt to its respective methyl halide, which in turn could act as the actual methylating agent, can be excluded. Additionally, when the N-methylation of benzyl amide is performed with MeI under basic conditions, the N-bis-methylated product is obtained exclusively, and no monoselectivity is observed.^24^ The latter results corroborate the hypothesis of a direct nucleophilic substitution mechanism rather than a pathway via thermal decomposition to MeI and even more emphasize the importance of finding novel monoselective protocols employing alternative reagents.

We performed additional experiments to demonstrate the remarkable selectivity of this new protocol. When monomethylated amides **2a** and **2b** were subjected again to the best performing reaction conditions (Table 1, entry 4), only 27% and 35% of bis-methylated products **3a** and **3b** were obtained (Scheme 1). For both reactions, mainly unreacted monomethylated starting material was recovered. This underlines the applicability of the developed reaction conditions for selective monomethylation because even when trying to enforce a second methylation, this works only poorly. These findings corroborate the hypothesis that an attachment of a sterically demanding CH_3_ group makes the nitrogen less prone to further deprotonation by a weak base and alters its nucleophilicity. Therefore, a second alkylation via the bulky ammonium salt is slowed significantly.

**Scheme 1:**
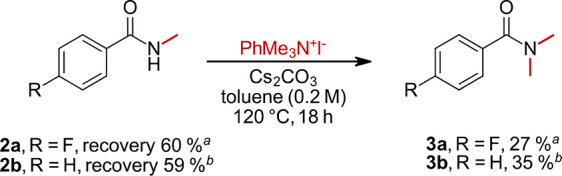
Reaction Using Monomethylated Benzamides as Starting Materials Yield determined by quantitative ^19^F NMR using trifluoro toluene as the internal standard. Isolated yields given.

With the optimized reaction conditions in hand, we applied the N-methylation reaction to various substrates, including amides, indoles, and a variety of structurally related bioactive compounds, to demonstrate the broad applicability of our developed protocol (Scheme 2).

**Scheme 2:**
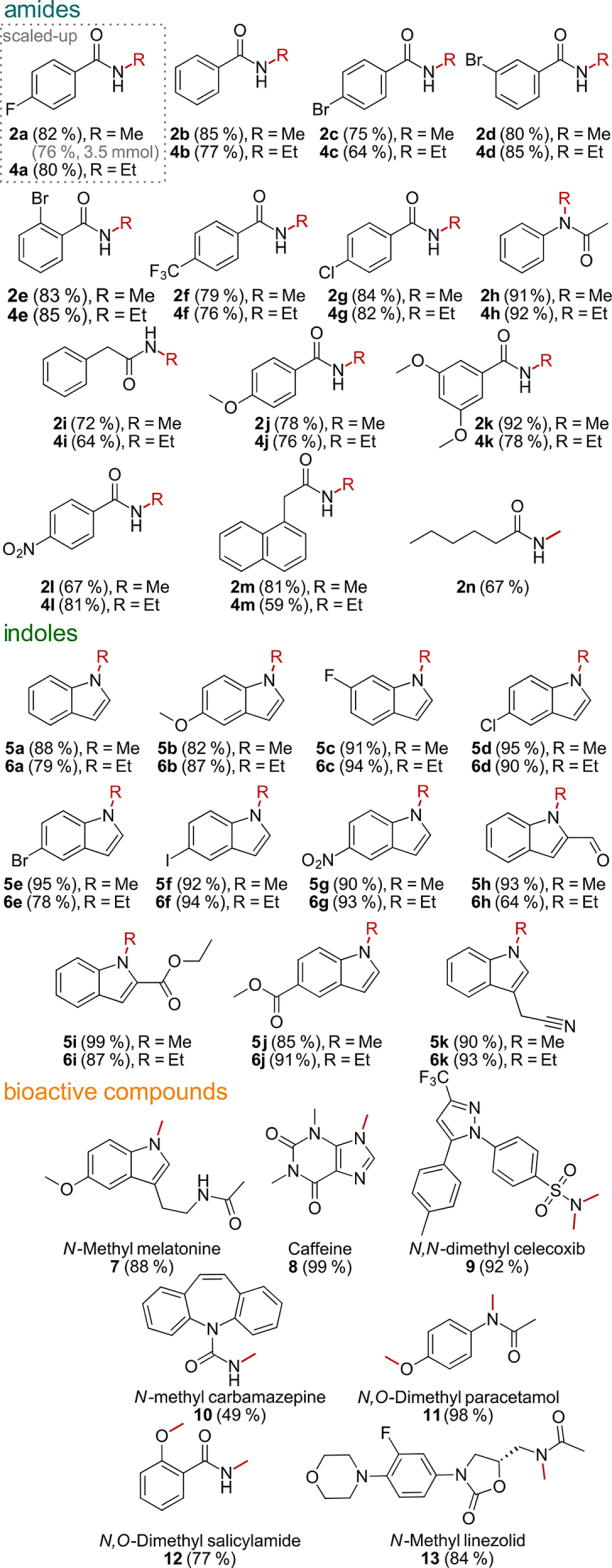
Scope of N-Methylation and N-Ethylation Reactions performed on a 100 mg scale, Cs_2_CO_3_ (2 equiv), ammonium salt (2.5 equiv): PhMe_3_NI (for products **2a–2m**, **5a–5k**, and **9–15**), PhEt_3_NI (for products **4a–4m** and **6a–6k**), toluene (0.23 M), 120 °C for 16–24 h.

In all reactions, *N,N*-dimethylamine is formed as a stoichiometric byproduct from PhMe_3_NI after its methyl group transfer. This byproduct can be either quenched *in situ* by conversion to its water-soluble HCl salt and subsequently removed in a mild acidic workup or, for acid-sensitive compounds, easily removed via column chromatography. The obtained results are compiled in Scheme 2.

The monoalkylated amides were obtained in yields of ≤91% for the methylation (products **2a–2n**) and 92% for the ethylation (products **4a–4m**). In all cases, a variety of functional groups on the benzamide, e.g., halides (products **2a**, **2c–2g**, **4a**, and **4c–4e**), a nitro group (products **2l** and **4l**), ether (products **2j**, **2k**, **4j**, and **4k**), and fused aromatic rings (products **2m** and **4m**), were used. Interestingly, an amide functionality at a benzylic position reacted chemoselectively without substitution at the α-position (products **2i**, **4i**, **2m**, and **4m**). This method, however, is not restricted to *para*-substituted amides but can be used to methylate *ortho*- and *meta*-substituted benzamides with comparable yields (cf. **2c** and **4c** to **2d**, **2e**, **4d**, and **4e**; cf. **2j** and **4j** to **2k** and **4k**).

The aliphatic amide hexanamide could be selectively monomethylated in a moderate yield of 67% (product **2n**). No bis-methylated product could be detected in the crude reaction mixture by NMR and LC-MS analysis, but unreacted starting material could be. This was also true for all other products with moderate yields. Only for products **2i** and **2m** could trace amounts (<8%) of bis-methylated species be detected via crude NMR. The reaction of *N*-acetylaniline also yielded the desired methylated and ethylated products **2h** and **4h**, showing that depending on the specific structure some secondary amides can be alkylated in excellent yields.

Because the experimental p*K*_a_ values in DMSO for **1h** (p*K*_a_ = 21.5^25^) and **2a** (p*K*_a_ = 21.5^26^) are in the same range, we hypothesize that the facile methylation of secondary amide **1h**, in comparison to the methylation of **2a** (see Scheme 1), might be caused by the lower steric demand of planar phenyl groups compared to a bulky methyl substituent directly attached to the nitrogen. Therefore, the nitrogen would be more readily approached by PhMe_3_NI for substrate **1h** than for **2a**. However, as the monomethylation toward secondary amides is much more demanding, we mainly focused on primary amides as starting materials. To further prove this method’s applicability and ease of operational setup, we performed the methylation of **1a** on a 3.52 mmol scale, giving **2a** in a 76% isolated yield.

In addition to amides, the indole motif is considered a privileged heterocyclic structure in biologically active compounds, as well.^27−29^ Hence, we tested whether indoles could be N-methylated and N-ethylated as well with our new protocol. Overall, indole-derived substances performed slightly better in this specific N-alkylation reaction than primary amides. A great range of functional groups was well tolerated, including halides (products **5c–5f** and **6c–6f**), ether (products **5b** and **6b**), nitro (products **5g** and **6g**), aldehyde (products **5h** and **6h**), esters (products **5i**, **5j**, **6i**, and **6j**), and nitrile (products **5k** and **6k**). The described methylation of selected indole derivatives with PhMe_3_NI under mild basic conditions gave yields as high as those of the methylation with Me_4_NF performed by the group of Schönebeck.^20^ In contrast to Me_4_NF, however, the use of anhydrous PhMe_3_NI and storage of the reagent in a glovebox are not required, which makes this presented method even more convenient.

To outline the potential of this method for late-stage functionalization of bioactive molecules, we performed methylation on a selection of established pharmaceuticals. Tryptamine-derived compounds, like melatonin, are methylated exclusively at the indole nitrogen atom in 88% yield (product **7**). Upon subjecting the N-monomethylated melatonin (product **7**) again to the reaction conditions mentioned above, we could observe no further methylation at the nitrogen of the secondary amide. Theophylline can be fully methylated to give caffeine (product **8**) in quantitative yield. The sulfonamide moiety in celecoxib is fully bis-methylated in an excellent yield of 92% (product **9**). Sulfonamides exhibit significantly lower p*K*_a_ values compared to those of benzamides; hence, a monomethylated sulfonamide readily undergoes a second substitution at the nitrogen. As in carbamazepine, a urea-derived functionality is monomethylated in moderate yield (product **10**). From previous results,^21^ we found hydroxy groups being readily methylated. Thus, as expected, paracetamol and salicylamide were methylated at the phenolic position and the amide moiety (products **11** and **12**), and the antibiotic linezolid can be N-methylated at the acetamide moiety with an 84% yield (product **13**).

In conclusion, we described a novel protocol for monoselective methylation and ethylation of amides, indoles, and related structures using solid, nontoxic, and easy-to-handle quaternary ammonium salts under mildly basic conditions. The method can also be applied to complex bioactive compounds and hence for late-stage modification of active pharmaceutical ingredients in drug discovery programs.

## Supporting Information Available

Detailed procedure for quantitative ^19^F NMR measurements, complete optimization screening data, experimental procedures, and characterization data for all compounds isolated (PDF)
