# Supplementary material for: Monoselective N-Methylation of Amides, Indoles, and Related Structures Using Quaternary Ammonium Salts as Solid Methylating Agents
Source: Org Lett. 2022 Oct 3;24(40):7315–9. doi: 10.1021/acs.orglett.2c02766 (PMC9578047; doi:10.1021/acs.orglett.2c02766)
Supplement: Supplementary file 1 — ol2c02766_si_001.pdf [file ol2c02766_si_001.pdf]

Supporting Information

Monoselective *N*-methylation of Amides, Indoles, and related Structures using Quaternary Ammonium Salts as Solid methylating Agents

Johanna Templ, Edma Gjata, Filippa Getzner, Michael Schnürch*

Institute of Applied Synthetic Chemistry, TU Wien, Getreidemarkt 9/163, 1060 Wien, Austria
E-mail: michael.schnuerch@tuwien.ac.at

Contents

General Experimental Details 2
General Procedures 2
Optimization Screening 2
Substrate Scope Methylation, Ethylation and Benzylation 4
Optimization Screening for Amide *N*-Methylation 4
Base: 4
Ammonium Salt: 5
Solvent: 5
Equivalents of $PhMe_3NI$ and Cs_2CO_3 6
Reaction Time: 6
Characterization data for all synthetic compounds 8
Substrate scope methylation 8
Substrate scope ethylation 16
NMR-Spectra 23
References 81

General Experimental Details

All Chemicals were purchased from commercial suppliers and, unless noted otherwise, used without further purification. The 8 mL glass vials were sealed with Wheaton® screw caps containing a PTFE faced 14B styrene-butadiene rubber liner for small-scale reaction above room temperature and heated in a metallic reaction block. All reaction temperatures refer to external temperatures.

^{1}H NMR, ^{13}C NMR, and ^{19}F NMR spectra were recorded on a Bruker Avance UltraShield 400 at ambient temperature. Chemical Shifts (δ) are reported in ppm, using Me_4Si as internal standard. Coupling constants (*J*) are given in Hertz (Hz) and multiplicities are assigned as s = singlet, d = doublet, t = triplet, q = quartet, and m = multiplet.
Quantitative ^{19}F-NMR spectra were recorded in a non-decoupled mode with a prolonged relaxation delay (d1 = 20 s), a narrowed spectral width (SW = 70 ppm), and a modified transmitter excitation frequency (O1T) to place the center of the spectrum between the peaks of interest (for details see Optimization Screening).

Thin Layer Chromatography (TLC) analysis was performed on aluminum-backed unmodified Merck silica gel 60 F_{245} plates. Visualization was realized under UV irradiation or *via* heat staining using a ceric ammonium molybdate aqueous solution. For flash column chromatography, Merck silica gel 60 (40 µm – 63 µm) was used, and purification was either done by hand-column or on a Büchi® Pure C-850 FlashPrep System.

GC-MS analysis was carried out on a Thermo Finnigan Focus GC/DSQ II with a standard capillary column RXi-5Sil MS column (30 m, 0.25 mm ID, 0.25 µm df) using the following standardized temperature program: 2 min at 100 °C, 35 °C/min until 300 °C, 4 min at 300 °C.

HR-MS analysis was performed on an Agilent 6230 LC TOFMS mass spectrometer equipped with an Agilent Dual AJS ESI-Source. The mass spectrometer was connected to a liquid chromatography system of the 1100/1200 series from Agilent Technologies, Palo Alto, CA, USA. The system consisted of a 1200SL binary gradient pump, a degasser, a column thermostat, and an HTC PAL autosampler (CTC Analytics AG, Zwingen, Switzerland). A silica-based Phenomenex C-18 Security Guard Cartridge was used as a stationary phase. Data evaluation was performed using Agilent MassHunter Qualitative Analysis B.07.00. Identification was based on peaks obtained from extracted ion chromatograms (extraction width ± 20 ppm).

General Procedures

Optimization Screening

General Procedure A:

4-Fluorobenzamide **(1a)** (50 mg, 0.352 mmol, 1 equiv), the respective ammonium salt (2 equiv), and the base (2 equiv) were placed in an 8 mL glass vial equipped with a magnetic stirring bar and a septum screw cap. *Via* a cannula, the vial was evacuated and backfilled with argon three times. Subsequently, the solvent (0.23 M) was added *via* syringe, and the evacuation and backfilling cycles were repeated under vigorous stirring so that no boiling delay occurred. The septum screw cap was replaced with a closed Wheaton® screw cap. The inhomogeneous reaction mixture was heated to 120 °C (or 100 °C for solvents with lower boiling points) in a metallic heating block for 18 h.

Sample preparation for quant. ^{19}F-NMR:
The reaction was cooled to room temperature and 100 µL of a solution of trifluorotoluene in $CHCl_3$ (52 mmol/mL) was added to the reaction mixture *via* Eppendorf® pipette. The inhomogeneous mixture was centrifuged, and 0.5 mL of the supernatant solution were transferred to an NMR tube. 0.3 mL $CDCl_3$ were added to the NMR tube, and the liquid content was homogenized thoroughly.

Quant. ^{19}F-NMR instrument parameters and processing:
NMR spectra were shimmed for $CDCl_3$ and recorded with the following changes in acquisition parameters:

- transmitter excitation frequency (O1T) = -87 ppm
- spectral width = 70 ppm
- relaxation delay = 20 s

After standard Fourier transformation, the recorded spectra were processed by MestReNova v12 software as following: [1]

- Apodization along t1: exponential 0.50 Hz
- Zero filling along t1: 512K
- Auto Phase Correction (Algorithms: Global, Selective, Metabonomics, Whitening, Min. Entropy, Baseline Optimization, Regions Analysis; Initial Phase: Zero)
- Auto Baseline Correction along t1: Ablative (5 Points, 10 Passes)

The following ^{19}F-NMR should serve as an example spectrum used for evaluation.

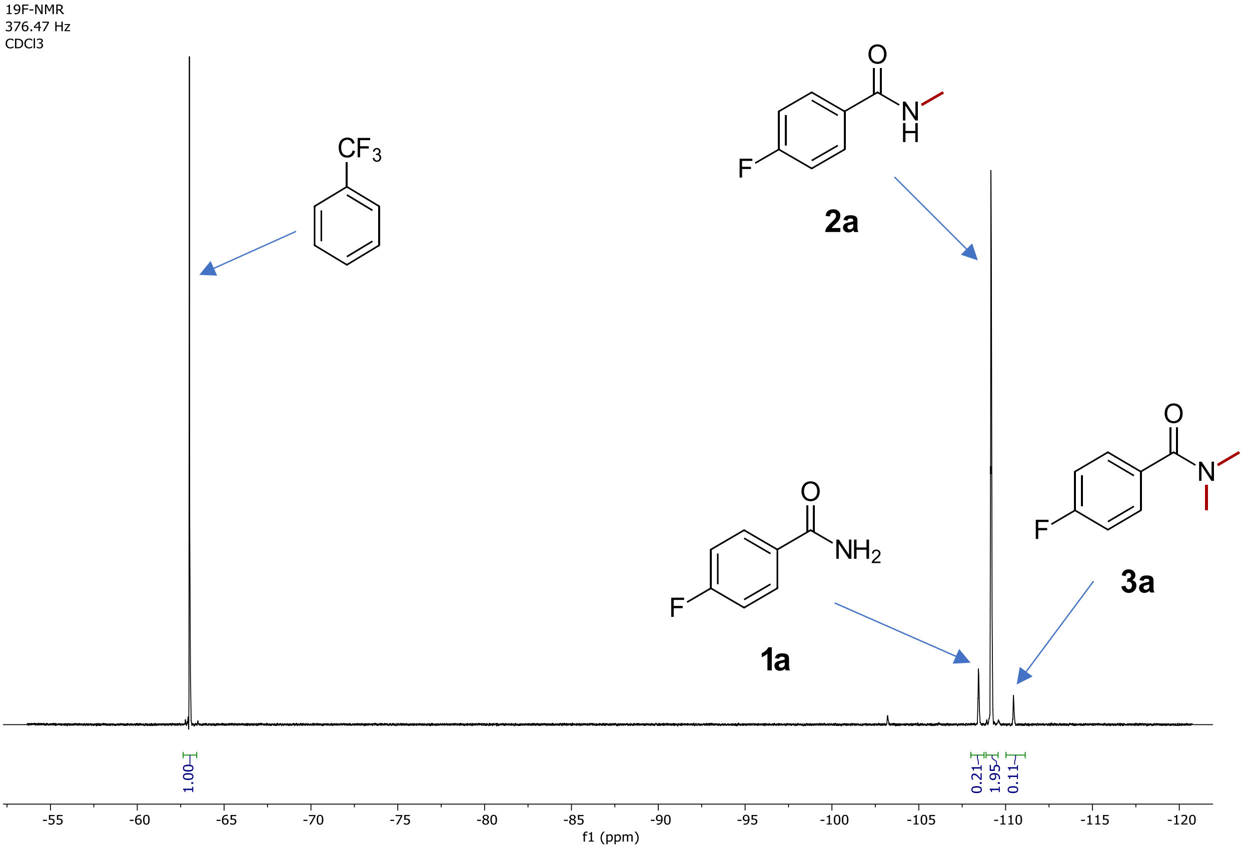

Figure S 1. example spectrum for quantitative ^{19}F NMR used for evaluation

Substrate Scope Methylation, Ethylation and Benzylation

General procedure B:

The starting material (100 mg, 1 equiv), the respective ammonium salt ($PhMe_3NI$, $PhEt_3NI$; 2.5 equiv), and Cs_2CO_3 (2 equiv) were placed in an 8 mL glass vial equipped with a magnetic stirring bar and a septum screw cap. Via a cannula, the vial was evacuated and backfilled with argon three times. Subsequently, toluene (0.23 M) was added via syringe, and the evacuation and backfilling cycles were repeated under vigorous stirring so that no boiling delay occurred. The septum screw cap was replaced with a closed Wheaton® screw cap. The inhomogeneous reaction mixture was heated to 120 °C in a metallic heating block for 15-24 h.

Work-up procedure A:

After the reaction was cooled to room temperature, 2 mL of deion. water were added, and the product was extracted 3 times with 10-15 mL EtOAc. The combined organic phases were washed once with brine, dried over Na_2SO_4, filtered, and concentrated to obtain a crude product which was further purified *via* hand column with unmodified silica gel.

Work-up procedure B:

After the reaction was cooled to room temperature 2 N HCl was added until gas evolution ceased (ca 2 mL). The product was extracted 3 times with 10-15 mL EtOAc, and the combined organic extracts were washed once twice with 3 mL 2 N HCl and once with brine, dried over Na_2SO_4, filtered and concentrated. The obtained crude product was purified *via* hand column using unmodified silica gel.

Optimization Screening for Amide *N*-Methylation

Yields, determined by quant. ^{19}F NMR, for the depicted reaction, are shown in the tables below. The following parameters were screened:

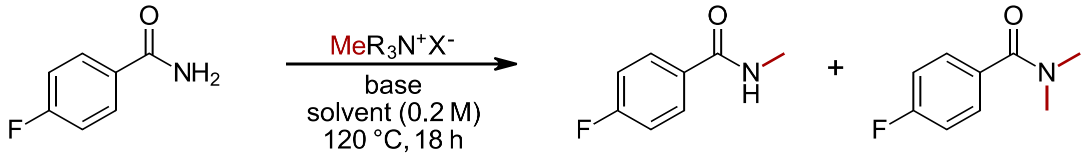

1a **2a** **3a**

Base:

Reactions were performed following the general procedure A using $PhMe_3NI$ (0.704 mmol, 2 equiv) as methylating agent, 0.704 mmol (2 equiv) of the respective base and toluene (1.5 mL, 0.23 M) as solvent at 120 °C for 18 h.

- KOH [CAS: 1310-58-3]
- NaOH [CAS: 1310-73-2]
- $LiOH \cdot H_2O$ [CAS: 1310-66-3]
- K_2CO_3 [CAS: 584-08-7]
- Cs_2CO_3 [CAS: 534-17-8]
- LiO^{t}Bu [CAS: 1907-33-1]

- KO^{t}Bu [CAS: 865-47-4]

Table S 1. Base Screening

entry	solvent	ammonium salt	base	conversion (%)	yield (%)	
					**2a**	**3a**
1	toluene	$PhMe_3NI$	KOH	81	56	19
2	toluene	$PhMe_3NI$	NaOH	43	11	7
3	toluene	$PhMe_3NI$	LiOH * H_2O	28	6	0
4	toluene	$PhMe_3NI$	K_2CO_3	7	6	0
5	toluene	$PhMe_3NI$	Cs_2CO_3	91	85	5
6	toluene	$PhMe_3NI$	LiO^{t}Bu	9	2	5
7	toluene	$PhMe_3NI$	KO^{t}Bu	69	16	0
8	toluene	$PhMe_3NI$	no base	7	0	0

Ammonium Salt:

Reactions were performed following the general procedure A using Cs_2CO_3 (0.704 mmol, 2 equiv) as the base, 0.704 mmol (2 equiv) of the respective ammonium salt and toluene (1.5 mL, 0.23 M) as solvent at 120 °C for 18 h.

- Me_4NF [CAS: 373-68-2]
- Me_4NCl [CAS: 75-57-0]
- Me_4NBr [CAS: 64-20-0]
- Me_4NI [CAS: 75-58-1]
- Me_3PhNCl [CAS: 138-24-9]
- Me_3PhNBr [CAS: 16056-11-4]
- Me_3PhNI [CAS: 98-04-4]

Table S 2. Ammonium Salt Screening

entry	solvent	ammonium salt	base	conversion (%)	yield (%)	
					**2a**	**3a**
1	toluene	Me_4NF	Cs_2CO_3	97	26	24
2	toluene	Me_4NCl	Cs_2CO_3	73	67	3
3	toluene	Me_4NBr	Cs_2CO_3	31	23	0
4	toluene	Me_4NI	Cs_2CO_3	8	4	0
5	toluene	$PhMe_3NCl$	Cs_2CO_3	96	78	7
6	toluene	$PhMe_3NBr$	Cs_2CO_3	99	78	11
7	toluene	$PhMe_3NI$	Cs_2CO_3	95	83	6

Solvent:

Reactions were performed following the general procedure A using $PhMe_3NI$ (0.704 mmol, 2 equiv) as methylating agent and Cs_2CO_3 (0.704 mmol, 2 equiv) as base with 1.5 mL solvent (0.23 M) at respective temperatures for 18 h.

- *t*-BuOH [CAS: 75-65-0] at 100 °C

- toluene [CAS: 108-88-3] at 120 °C
- CPME [CAS: 5614-37-9] at 120 °C
- anisole [CAS: 100-66-3] at 120 °C

Table S 3. Solvent Screening

entry	solvent	ammonium salt	base	conversion (%)	yield (%) **2a**	yield (%) **3a**
1	*t*-BuOH	$PhMe_3NI$	Cs_2CO_3	79	65	7
2	toluene	$PhMe_3NI$	Cs_2CO_3	95	84	6
3	CPME	$PhMe_3NI$	Cs_2CO_3	94	74	7
4	anisole	$PhMe_3NI$	Cs_2CO_3	89	73	5

Equivalents of $PhMe_3NI$ and Cs_2CO_3

Reactions were performed following the general procedure A using $PhMe_3NI$ as methylating agent and Cs_2CO_3 as base with toluene (1.5 mL, 0.23 M) as solvent at 120 °C for 18 h.

Table S 4. Equivalents of $PhMe_3NI$ and Cs_2CO_3 Screening

entry	solvent	$PhMe_3NI$ [mmol] (equiv)	Cs_2CO_3 [mmol] (equiv)	conversion (%)	yield (%) **2a**	yield (%) **3a**
1	toluene	0.352 (1)	0.704 (2)	74	60	2
2	toluene	0.704 (2)	0.704 (2)	92	74	4
3	toluene	1.056 (3)	0.704 (2)	98	77	8
4	toluene	0.352 (1)	0.352 (1)	55	43	1
5	toluene	0.704 (2)	0.352 (1)	66	52	1

Reaction Time:

Reactions were performed following the general procedure A using $PhMe_3NI$ (0.704 mmol, 2 equiv) as methylating agent and Cs_2CO_3 (0.704 mmol, 2 equiv) as base with toluene (1.5 mL, 0.23 M) as solvent at 120 °C for the respective reaction time.

Table S 5. Reaction Time Screening

entry	time [h]	conversion (%)	yield (%) **2a**	yield (%) **3a**
1	0.5	45	35	0
2	1	56	56	0
3	2	65	63	1
4	3	63	62	1
5	5	76	67	2
6	7	89	82	4
7	9	92	82	4
8	24	92	82	4

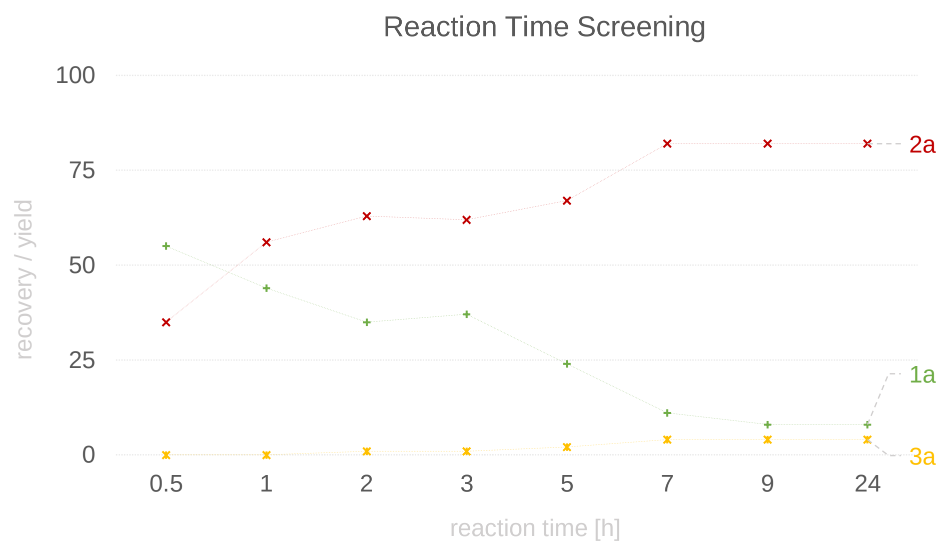
Reaction Time Screening
100
75
50
25
0
recovery / yield
0.5
1
2
3
5
7
9
24
reaction time [h]
2a
1a
3a

Characterization data for all synthetic compounds

All compounds synthesized are described in the literature, except **11** and **15**. For known compounds, spectral data is in agreement with the literature.

Substrate scope methylation

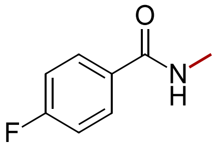

***N*-Methyl-4-fluorobenzamide[2] (2a) [CAS: 701-49-5]**
Prepared, following the general procedure from commercially available starting material with a reaction time of 20 h. Work-up procedure A was followed (10 g silica, DCM:MeOH 100:1, 50:1), yielding 88 mg (82 %) of the title compound as white crystals.

Scaled up synthesis: The synthesis of **2a** was additionally performed on a 3.52 mmol scale. The starting material (0.5 g, 3.52 mmol, 1 equiv), $PhMe_3NI$ (2.36 g, 8.80 mmol, 2.5 equiv), and Cs_2CO_3 (2.32 g, 7.04 mmol, 2 equiv) were placed in a 50 mL pressure flask equipped with a magnetic stirring bar. For degassing, the opening of the flask was temporarily covered with a septum. *Via* a cannula, the flask was evacuated and backfilled with argon three times. Subsequently, toluene (15 mL) was added *via* syringe, and the evacuation and backfilling cycles were repeated under vigorous stirring so that no boiling delay occurred. The septum screw cap was replaced with a closed screw cap. The inhomogeneous reaction mixture was heated to 120 °C in an oil bath for 20 h. After complete conversion the reaction was cooled to room temperature.
10 mL H_2O were added, and the product was extracted 4 times with 40 mL EtOAc each. The combined organic extracts were washed three times with 5 mL 2 N HCl each and once with brine, dried over Na_2SO_4, filtered, and concentrated. The crude product was purified by column chromatography (50 g silica, DCM:MeOH 100:1, 30:1), yielding 407 mg (76 %) of the title compound as white crystals. Analytical data was in accordance with previous measurements.

^{1}H NMR (400 MHz, $CDCl_3$) δ 7.82 – 7.72 (m, 2H), 7.07 – 6.98 (m, 2H), 6.93 (bs, 1H), 2.92 (d, J = 4.8 Hz, 3H).

^{13}C{^{1}H} NMR (101 MHz, $CDCl_3$) δ 167.5, 164.6 (d, J = 251.5 Hz), 130.8 (d, J = 3.2 Hz), 129.3 (d, J = 8.8 Hz), 115.4 (d, J = 21.8 Hz), 26.9.

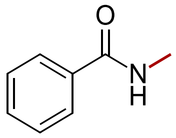

***N*-Methylbenzamide[2] (2b) [CAS: 613-93-4]**

Prepared, following the general procedure from commercially available starting material with a reaction time of 17 h. Work-up procedure A was followed (10 g silica, DCM:MeOH 100:1, 75:1, 50:1), yielding 95 mg (85 %) of the title compound as a colorless oil.

^{1}H NMR (400 MHz, $CDCl_3$) δ 7.80 – 7.72 (m, 2H), 7.50 – 7.41 (m, 1H), 7.41 – 7.32 (m, 2H), 6.59 (s, 1H), 2.97 (d, J = 4.9 Hz, 3H).

^{13}C{^{1}H} NMR (101 MHz, $CDCl_3$) δ 168.4, 134.7, 131.3, 128.5, 126.9, 26.9.

HRMS (ESI): m/z $[M+H]^+$ calcd. for $C_8H_{10}NO$: 136.0757; found: 136.0759

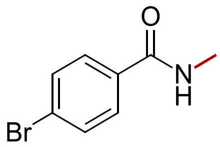

4-Bromo-*N*-methylbenzamide[2] (2c) [CAS: 27466-83-7]

Prepared, following the general procedure from commercially available starting material with a reaction time of 18 h. Work-up procedure A was followed (10 g silica, DCM:MeOH 100:1, 50:1), yielding 79 mg (75 %) of the title compound as slightly blue crystals.

^{1}H NMR (400 MHz, $CDCl_3$) δ 7.65 – 7.58 (m, 2H), 7.57 – 7.49 (m, 2H), 6.42 (s, 1H), 2.97 (d, *J* = 4.8 Hz, 3H).

^{13}C{^{1}H} NMR (101 MHz, $CDCl_3$) δ 167.4, 133.5, 131.8, 128.6, 126.1, 27.0.

HRMS (ESI): m/z $[M+H]^+$ calcd. for C_8H_9BrNO: 213.9862; found: 213.9868

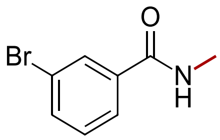

3-Bromo-*N*-methylbenzamide[3] (2d) [CAS: 49834-22-2]

Prepared, following the general procedure from commercially available starting material with a reaction time of 17 h. Work-up procedure A was followed (10 g silica, DCM:MeOH 100:1), yielding 84 mg (80 %) of the title compound as white amorphous solid.

^{1}H NMR (400 MHz, $CDCl_3$) δ 7.90 (t, *J* = 1.8 Hz, 1H), 7.67 (ddd, *J* = 7.8, 1.7, 1.1 Hz, 1H), 7.56 (ddd, *J* = 8.0, 2.0, 1.0 Hz, 1H), 7.24 (t, *J* = 7.9 Hz, 1H), 6.80 (s, 1H), 2.96 (d, *J* = 4.8 Hz, 3H).

^{13}C{^{1}H} NMR (101 MHz, $CDCl_3$) 167.0, 136.6, 134.3, 130.2, 130.1, 125.6, 122.7, 27.0

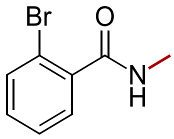

2-Bromo-*N*-methylbenzamide[4] (2e) [CAS: 61436-88-2]

Prepared, following the general procedure from commercially available starting material with a reaction time of 22 h. Work-up procedure A was followed (10 g silica, DCM:MeOH 100:1), yielding 87 mg (83 %) of the title compound as white amorphous solid.

^{1}H NMR (400 MHz, $CDCl_3$) δ 7.55 (dd, *J* = 7.9, 1.3 Hz, 1H), 7.46 (dd, *J* = 7.6, 1.8 Hz, 1H), 7.34 – 7.20 (m, 2H), 6.17 (s, 1H), 2.97 (d, *J* = 4.9 Hz, 3H).

^{13}C{^{1}H} NMR (101 MHz, $CDCl_3$) δ 168.4, 138.0, 133.3, 131.2, 129.5, 127.5, 119.3, 26.8.

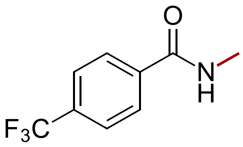

***N*-Methyl-4-(trifluoromethyl) benzamide[3] (2f) [CAS: 65017-76-7]**

Prepared, following the general procedure from commercially available starting material with a reaction time of 18 h. Work-up procedure A was followed (10 g silica, DCM:MeOH 100:1), yielding 85 mg (79 %) of the title compound as white crystals.

^{1}H NMR (400 MHz, $CDCl_3$) δ 7.86 (dt, *J* = 8.0, 0.8 Hz, 2H), 7.70 – 7.63 (m, 2H), 6.46 (s, 1H), 3.01 (d, *J* = 4.9 Hz, 3H).

^{13}C{^{1}H} NMR (101 MHz, $CDCl_3$) δ 167.1, 138.0, 133.2 (q, *J* = 32.8 Hz), 127.6, 125.7 (q, *J* = 3.8 Hz), 122.4 (q, *J* = 273.4 Hz), 27.0.

^{19}F NMR{^{1}H} (376 MHz, $CDCl_3$) δ -62.97.

HRMS (ESI): m/z $[M+H]^+$ calcd. for $C_9H_9F_3NO$: 204.0631; found: 204.0634

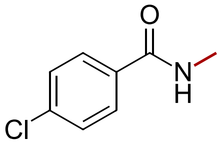

4-Chloro-*N*-methylbenzamide (2g) [2] [CAS: 6873-44-5]

Prepared, following the general procedure from commercially available starting material with a reaction time of 20 h. Work-up procedure A was followed (10 g silica, DCM:MeOH 100:1, 50:1), yielding 90 mg (84 %) of the title compound as white crystals.

^{1}H NMR (400 MHz, $CDCl_3$) δ 7.73 – 7.65 (m, 2H), 7.40 – 7.32 (m, 2H), 6.45 (s, 1H), 2.98 (d, *J* = 4.8 Hz, 3H).

^{13}C{^{1}H} NMR (101 MHz, $CDCl_3$) δ 167.3, 137.6, 133.0, 128.8, 128.4, 27.0.

HRMS (ESI): m/z $[M+H]^+$ calcd. for C_8H_9ClNO: 170.0367; found: 170.0369

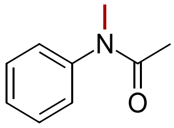

***N*-Acetyl-*N*-methylaniline[5] (2h) [CAS: 579-10-2]**

Prepared, following the general procedure from commercially available starting material with a reaction time of 20 h. Work-up procedure A was followed (10 g silica, DCM:MeOH 50:1), yielding 97 mg (91 %) of the title compound as a colorless oil.

^{1}H NMR (400 MHz, $CDCl_3$) δ 7.38 (dd, *J* = 8.5, 6.9 Hz, 2H), 7.29 (t, *J* = 7.4 Hz, 1H), 7.20 – 7.09 (m, 2H), 3.22 (s, 3H), 1.83 (s, 3H).

^{13}C{^{1}H} NMR (101 MHz, $CDCl_3$) δ 170.5, 144.64, 129.7, 127.7, 127.1, 37.1, 22.4.

HRMS (ESI): m/z $[M+H]^+$ calcd. for $C_9H_{12}NO$: 150.0914; found: 150.0915

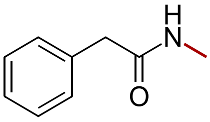

***N*-Methylphenylacetamide[6] (2i) [CAS: 6830-82-6]**

Prepared, following the general procedure from commercially available starting material with a reaction time of 16 h. Work-up procedure A was followed (10 g silica, DCM:MeOH 100:1, 50:1), yielding 80 mg (72 %) of the title compound a colorless oil.

^{1}H NMR (400 MHz, $CDCl_3$) δ 7.41 – 7.19 (m, 5H), 5.69 (s, 1H), 3.55 (s, 2H), 2.74 (d, *J* = 4.9 Hz, 3H).

^{13}C{^{1}H} NMR (101 MHz, $CDCl_3$) δ 171.7, 135.0, 129.5, 129.0, 127.3, 43.7, 26.5.

HRMS (ESI): m/z $[M+H]^+$ calcd. for $C_9H_{12}NO$: 150.0914; found: 150.0917

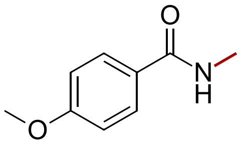

4-Methoxy-*N*-methylbenzamide[2] (2j) [CAS:3400-22-4]

Prepared, following the general procedure from commercially available starting material with a reaction time of 20 h. Work-up procedure A was followed (10 g silica, DCM:MeOH 100:1, 50:1), yielding 83 mg (78 %) of the title compound a colorless oil.

^{1}H NMR (400 MHz, $CDCl_3$) δ 7.77 – 7.69 (m, 2H), 6.90 – 6.82 (m, 2H), 6.53 (s, 1H), 3.80 (s, 3H), 2.94 (d, *J* = 4.8 Hz, 3H).

^{13}C{^{1}H} NMR (101 MHz, $CDCl_3$) δ 167.9, 162.0, 128.7, 127.0, 113.7, 55.4, 26.8.

HRMS (ESI): m/z $[M+H]^+$ calcd. for $C_9H_{12}NO_2$: 166.0862; found: 166.0864

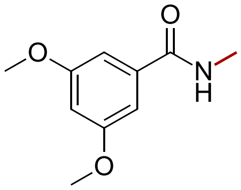

3,5-Dimethoxy-*N*-methylbenzamide[7] (2k) [CAS: 74826-21-4]

Prepared, following the general procedure from commercially available starting material with a reaction time of 17 h. Work-up procedure A was followed (10 g silica, DCM:MeOH 100:1), yielding 97 mg (92 %) of the title compound an oily liquid.

^{1}H NMR (400 MHz, $CDCl_3$) δ 6.88 (d, *J* = 2.3 Hz, 2H), 6.63 (bs, 1H), 6.54 – 6.39 (m, 1H), 3.75 (s, 6H), 2.92 (d, *J* = 4.8 Hz, 3H).

^{13}C{^{1}H} NMR (101 MHz, $CDCl_3$) δ 168.2, 160.8, 136.9, 104.9, 103.5, 101.5, 55.5, 26.8.

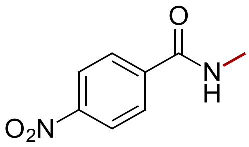

N-Methyl-4-nitrobenzamide[8] (2l) [CAS: 2585-23-1]

Prepared, following the general procedure from commercially available starting material with a reaction time of 11 h. Work-up procedure A was followed (10 g silica, DCM:MeOH 100:1), yielding 71 mg (67 %) of the title compound as yellow solid.

^{1}H NMR (400 MHz, DMSO) δ 8.77 (s, 1H), 8.35 – 8.26 (m, 2H), 8.09 – 8.01 (m, 2H), 2.81 (d, *J* = 4.6 Hz, 3H).

^{13}C{^{1}H} NMR (101 MHz, DMSO) δ 164.9, 148.9, 140.1, 128.5, 123.5, 26.3.

HRMS (ESI): m/z $[M+H]^+$ calcd. for $C_8H_9N_2O_3$: 181.0608; found: 181.0611

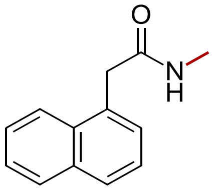

***N*-Methyl-α-naphthylacetamide (2m) [CAS:1136-81-8]**

Prepared, following the general procedure from commercially available starting material with a reaction time of 20 h. Work-up procedure A was followed (10 g silica, LP:EA 4:1, 2:3, 1:10), yielding 61 mg (58 %) of the title compound as white crystals.

^{1}H NMR (400 MHz, $CDCl_3$) δ 8.00 – 7.91 (m, 1H), 7.91 – 7.84 (m, 1H), 7.82 (dt, *J* = 8.1, 1.2 Hz, 1H), 7.59 – 7.48 (m, 2H), 7.45 (dd, *J* = 8.2, 6.9 Hz, 1H), 7.42 – 7.34 (m, 1H), 5.38 (s, 1H), 4.01 (s, 2H), 2.66 (d, *J* = 4.9 Hz, 3H).

^{13}C{^{1}H} NMR (101 MHz, $CDCl_3$) δ 171.5, 134.0, 132.1, 131.2, 128.8, 128.5, 128.5, 126.9, 126.3, 125.7, 123.9, 41.7, 26.5.

HRMS (ESI): m/z $[M+H]^+$ calcd. for $C_{13}H_{14}NO$: 200.1070; found: 200.1072

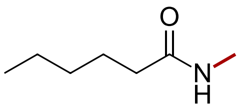

***N*-Methylhexanamide (2n)[9] [CAS: 3418-05-1]**

Prepared, following the general procedure from commercially available starting material with a reaction time of 14 h. Work-up procedure B was followed (10 g silica, DCM:MeOH 100:1), yielding 74 mg (67 %) of the title compound as a colorless oil.

^{1}H NMR (400 MHz, $CDCl_3$) δ 6.14 (s, 1H), 2.73 (d, *J* = 4.8 Hz, 3H), 2.12 (d, *J* = 7.4 Hz, 2H), 1.63 – 1.51 (m, 2H), 1.33 – 1.16 (m, 4H), 0.83 (t, *J* = 6.9 Hz, 3H).

^{13}C{^{1}H} NMR (101 MHz, $CDCl_3$) δ 174.1, 36.6, 31.5, 26.2, 25.5, 22.4, 13.9.

1-Methylindole (5a)[5] [CAS: 603-76-9]

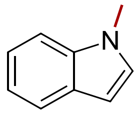

Prepared, following the general procedure from commercially available starting material with a reaction time of 16 h. Work-up procedure B was followed (10 g silica, LP:EA 30:1), yielding 97 mg (88 %) of the title compound as a colorless oil.

^{1}H NMR (400 MHz, $CDCl_3$) δ 7.72 (dt, *J* = 7.9, 1.0 Hz, 1H), 7.39 (dq, *J* = 8.2, 0.9 Hz, 1H), 7.35 – 7.27 (m, 1H), 7.24 – 7.15 (m, 1H), 7.10 (d, *J* = 3.1 Hz, 1H), 6.57 (dd, *J* = 3.1, 0.9 Hz, 1H), 3.83 (s, 3H).

^{13}C{^{1}H} NMR (101 MHz, $CDCl_3$) δ 136.8, 128.8, 128.5, 121.5, 120.9, 119.3, 109.2, 100.9, 32.8.

HRMS (ESI): m/z $[M+H]^+$ calcd. for $C_9H_{10}N$: 132.0808; found: 132.0812

5-Methoxy-1-methylindole (5b)[5] [CAS: 2521-13-3]

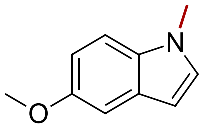

Prepared, following the general procedure from commercially available starting material with a reaction time of 19 h. Work-up procedure B was followed (10 g silica, LP:EA 50:1, 40:1), yielding 89 mg (82 %) of the title compound as a colorless oil.

^{1}H NMR (400 MHz, $CDCl_3$) δ 7.26 – 7.19 (m, 1H), 7.12 (d, *J* = 2.5 Hz, 1H), 7.03 (d, *J* = 3.1 Hz, 1H), 6.91 (dd, *J* = 8.8, 2.5 Hz, 1H), 6.42 (dd, *J* = 3.1, 0.8 Hz, 1H), 3.87 (s, 3H), 3.77 (s, 3H).

^{13}C{^{1}H} NMR (101 MHz, $CDCl_3$) δ 154.1, 132.2, 129.4, 128.9, 111.9, 110.0, 102.6, 100.5, 56.0, 33.0.

HRMS (ESI): m/z $[M+H]^+$ calcd. for $C_{10}H_{12}NO$: 162.0914; found: 162.0917

6-Fluoro-1-methylindole [5] (5c) [CAS: 441715-92-0]

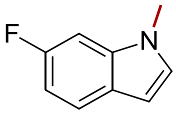

Prepared, following the general procedure from commercially available starting material with a reaction time of 15 h. Work-up procedure B was followed (10 g silica, LP:EA 10:1), yielding 98 mg (91 %) of the title compound as a colorless oil.

^{1}H NMR (400 MHz, $CDCl_3$) δ 7.64 – 7.56 (m, 1H), 7.10 – 6.98 (m, 2H), 7.01 – 6.90 (m, 1H), 6.54 (dd, *J* = 3.2, 0.9 Hz, 1H), 3.75 (s, 3H).

^{13}C{^{1}H} NMR (101 MHz, $CDCl_3$) δ 159.9 (d, *J* = 237.2 Hz), 136.8 (d, *J* = 12.1 Hz), 129.3 (d, *J* = 3.8 Hz), 125.0, 121.6 (d, *J* = 10.0 Hz), 108.0 (d, *J* = 24.5 Hz), 101.1, 95.6 (d, *J* = 26.1 Hz), 32.8.

HRMS (ESI): m/z $[M+H]^+$ calcd. for C_9H_9FN: 150.0714; found: 150.0718

5-Chloro-1-methylindole [10] (5d) [CAS: 112398-75-1]

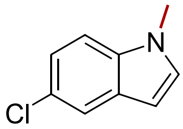

Prepared, following the general procedure from commercially available starting material with a reaction time of 16 h. Work-up procedure B was followed (10 g silica, LP:EA 10:1), yielding 102 mg (95 %) of the title compound as a colorless oil.

^{1}H NMR (400 MHz, $CDCl_3$) δ 7.66 – 7.60 (m, 1H), 7.28 – 7.16 (m, 2H), 7.08 (d, *J* = 3.1 Hz, 1H), 6.46 (dd, *J* = 3.1, 0.8 Hz, 1H), 3.77 (s, 3H).

^{13}C{^{1}H} NMR (101 MHz, $CDCl_3$) δ 135.2, 130.2, 129.5, 125.1, 121.8, 120.2, 110.3, 100.6, 33.0.

HRMS (ESI): m/z $[M+H]^+$ calcd. for C_9H_9ClN: 166.0418; found: 166.0420

5-Bromo-1-methylindole[10] (5e) [CAS: 10075-52-2]

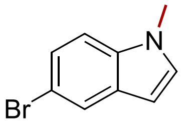

Prepared, following the general procedure from commercially available starting material with a reaction time of 15 h. Work-up procedure B was followed (10 g silica, LP:EA 10:1), yielding 101 mg (95 %) of the title compound as a colorless oil.

^{1}H NMR (400 MHz, $CDCl_3$) δ 7.78 (dd, *J* = 1.9, 0.6 Hz, 1H), 7.32 (ddd, *J* = 8.7, 1.9, 0.4 Hz, 1H), 7.19 (dt, *J* = 8.7, 0.7 Hz, 1H), 7.05 (d, *J* = 3.1 Hz, 1H), 6.45 (dd, *J* = 3.1, 0.9 Hz, 1H), 3.76 (s, 3H).

^{13}C{^{1}H} NMR (101 MHz, $CDCl_3$) δ 135.4, 130.2, 130.0, 124.3, 123.3, 112.7, 110.7, 100.6, 33.0.

HRMS (ESI): m/z $[M+H]^+$ calcd. for C_9H_9BrN: 209.9913; found: 209.9914

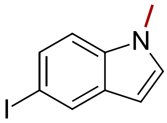

5-Iodo-1-methylindole [5] (5f) [CAS: 280563-07-7]

Prepared, following the general procedure from commercially available starting material with a reaction time of 17 h. Work-up procedure B was followed (10 g silica, LP:EA 10:1), yielding 92 mg (92 %) of the title compound as a colorless oil.

^{1}H NMR (400 MHz, $CDCl_3$) δ 7.97 (dd, *J* = 1.7, 0.6 Hz, 1H), 7.47 (dd, *J* = 8.6, 1.7 Hz, 1H), 7.10 (dt, *J* = 8.6, 0.7 Hz, 1H), 7.01 (d, *J* = 3.1 Hz, 1H), 6.41 (dd, *J* = 3.1, 0.9 Hz, 1H), 3.76 (s, 3H).

^{13}C{^{1}H} NMR (101 MHz, $CDCl_3$) δ 135.8, 131.1, 129.8, 129.7, 129.7, 111.3, 100.3, 82.9, 33.0.

HRMS (ESI): m/z $[M+H]^+$ calcd. for C_9H_9IN: 257.9775; found: 257.9777

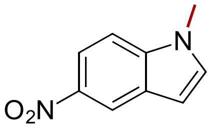

1-Methyl-5-nitroindole [11] (5g) [CAS: 29906-67-0]

Prepared, following the general procedure from commercially available starting material with a reaction time of 17 h. Work-up procedure B was followed (10 g silica, LP:EA 10:1), yielding 98 mg (90 %) of the title compound as yellow crystals.

^{1}H NMR (400 MHz, $CDCl_3$) δ 8.55 (d, *J* = 2.3 Hz, 1H), 8.10 (dd, *J* = 9.1, 2.2 Hz, 1H), 7.31 (d, *J* = 9.1 Hz, 1H), 7.20 (d, *J* = 3.2 Hz, 1H), 6.65 (dd, *J* = 3.2, 0.8 Hz, 1H), 3.85 (s, 3H).

^{13}C{^{1}H} NMR (101 MHz, $CDCl_3$) δ 141.6, 139.5, 132.1, 127.7, 118.2, 117.2, 109.1, 103.9, 33.3.

HRMS (ESI): m/z $[M+H]^+$ calcd. for $C_9H_9N_2O_2$: 177.0659; found: 177.0661

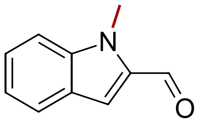

1-Methyl-2-indolecarbaldehyde [12] (5h) [CAS: 27421-51-8]

Prepared, following the general procedure from commercially available starting material with a reaction time of 17 h. Work-up procedure B was followed (10 g silica, LP:EA 10:1), yielding 99 mg (93 %) of the title compound as yellow solid.

^{1}H NMR (400 MHz, $CDCl_3$) δ 9.89 (s, 1H), 7.74 (dt, *J* = 8.2, 1.0 Hz, 1H), 7.48 – 7.35 (m, 2H), 7.24 (d, *J* = 0.9 Hz, 1H), 7.21 – 7.14 (m, 1H), 4.09 (s, 3H).

^{13}C{^{1}H} NMR (101 MHz, $CDCl_3$) δ 182.9, 140.9, 135.7, 126.9, 126.3, 123.4, 121.0, 117.5, 110.4, 31.6.

HRMS (ESI): m/z $[M+H]^+$ calcd. for $C_{10}H_{10}NO$: 160.0757; found: 160.0760

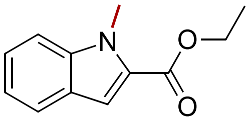

Ethyl 1-methyl-2-indolecarboxylate [13] (5i) [CAS: 18450-24-3]

Prepared, following the general procedure from commercially available starting material with a reaction time of 23 h. Work-up procedure B was followed (10 g silica, LP:EA 30:1), yielding 106 mg (99 %) of the title compound as off-white solid.

^{1}H NMR (400 MHz, $CDCl_3$) δ 7.70 (dt, *J* = 8.0, 1.0 Hz, 1H), 7.43 – 7.29 (m, 3H), 7.21 – 7.13 (m, 1H), 4.40 (q, *J* = 7.1 Hz, 2H), 4.09 (s, 3H), 1.44 (t, *J* = 7.1 Hz, 3H).

^{13}C{^{1}H} NMR (101 MHz, $CDCl_3$) δ 162.3, 139.7, 128.1, 125.9, 125.0, 122.6, 120.6, 110.3, 110.1, 60.6, 31.6, 14.4.

HRMS (ESI): m/z $[M+H]^+$ calcd. for $C_{12}H_{14}NO_2$: 204.1019; found: 204.1022

Methyl 1-methyl-5-indolecarboxylate [11] (5j) [CAS: 128742-76-7]

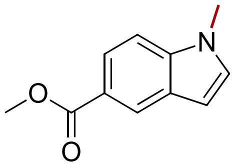

Prepared, following the general procedure from commercially available starting material with a reaction time of 23 h. Work-up procedure B was followed (10 g silica, LP:EA 30:1, 20:1, 10:1), yielding 91 mg (85 %) of the title compound as white crystals.

^{1}H NMR (400 MHz, $CDCl_3$) δ 8.31 (dd, *J* = 1.7, 0.7 Hz, 1H), 7.84 (dd, *J* = 8.7, 1.7 Hz, 1H), 7.22 (dt, *J* = 8.7, 0.8 Hz, 1H), 7.01 (d, *J* = 3.2 Hz, 1H), 6.49 (dd, *J* = 3.2, 0.9 Hz, 1H), 3.84 (s, 3H), 3.71 (s, 3H).

^{13}C{^{1}H} NMR (101 MHz, $CDCl_3$) δ 168.3, 139.2, 130.3, 128.0, 124.0, 123.0, 121.4, 108.9, 102.7, 51.9, 33.1.

HRMS (ESI): m/z $[M+H]^+$ calcd. for $C_{11}H_{12}NO_2$: 190.0863; found: 190.0865

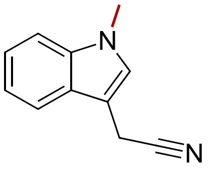

(1-Methyl-3-indolyl)acetonitrile [14] (5k) [CAS: 51584-17-9]

Prepared, following the general procedure from commercially available starting material with a reaction time of 18 h. Work-up procedure B was followed (10 g silica, LP:EA 20:1, 10:1), yielding 96 mg (90 %) of the title compound as a yellow oil.

^{1}H NMR (400 MHz, $CDCl_3$) δ 7.60 (dt, *J* = 7.9, 1.0 Hz, 1H), 7.39 – 7.29 (m, 2H), 7.25 – 7.17 (m, 1H), 7.08 (t, *J* = 1.1 Hz, 1H), 3.81 (d, *J* = 1.1 Hz, 2H), 3.76 (s, 3H).

^{13}C{^{1}H} NMR (101 MHz, $CDCl_3$) δ 137.1, 127.4, 126.4, 122.4, 119.7, 118.3, 118.2, 109.7, 102.9, 32.8, 14.2.

HRMS (ESI): m/z $[M+H]^+$ calcd. for $C_{11}H_{11}N_2$: 171.0917; found: 171.0920

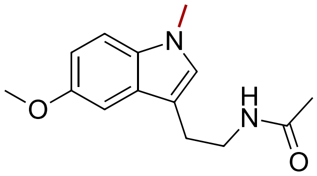

***N*-Methylmelatonine [15] (7) [CAS: 53350-25-7]**

Prepared, following the general procedure from the commercially available, bioactive compound melatonine with a reaction time of 23 h. Work-up procedure A was followed (10 g silica, DCM:MeOH 100:1), yielding 91 mg (88 %) of the title compound as a colorless oil.

^{1}H NMR (400 MHz, $CDCl_3$) δ 7.18 (dd, *J* = 8.9, 0.5 Hz, 1H), 7.03 (d, *J* = 2.4 Hz, 1H), 6.89 (dd, *J* = 8.9, 2.4 Hz, 1H), 6.85 (s, 1H), 5.78 (s, 1H), 3.85 (s, 3H), 3.71 (s, 3H), 3.55 (td, *J* = 6.8, 5.7 Hz, 2H), 2.91 (td, *J* = 6.8, 0.9 Hz, 2H), 1.92 (s, 3H).

^{13}C{^{1}H} NMR (101 MHz, $CDCl_3$) δ 170.1, 153.8, 132.5, 128.1, 127.4, 112.0, 110.9, 110.1, 100.6, 56.0, 39.9, 32.8, 25.2, 23.4.

HRMS (ESI): m/z $[M+H]^+$ calcd. for $C_{14}H_{19}N_2O_2$: 247.1441; found: 247.1444

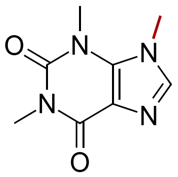

Caffeine (8) [CAS: 69-22-7]

Prepared, following the general procedure from commercially available Theophyllin with a reaction time of 20 h. After coolin the reaction to room temperature, 2 mL deion. water were added and the product was extracted 4 times with 10 mL DCM. The combined organic extracts were washed once with brine, dried over Na2SO4, filtered and concentrated. The crude product was purified by trituration with hexane, to obtain 100 mg (99 %) as off-white powder.

^{1}H NMR (400 MHz, $CDCl_3$) δ 7.50 (s, 1H), 3.98 (s, 3H), 3.58 (s, 3H), 3.40 (s, 3H).

^{13}C{^{1}H} NMR (101 MHz, $CDCl_3$) δ 155.5, 151.8, 148.8, 141.5, 107.7, 33.7, 29.8, 28.0.

HRMS (ESI): m/z $[M+H]^+$ calcd. for $C_8H_{11}N_4O_2$: 195.0877; found: 195.0879

_N,N_-Dimethyl celecoxib (9) [CAS: 2412733-30-1]

Prepared, following the general procedure from commercially available, bioactive celecoxib with a reaction time of 20 h. Work-up procedure A was followed (10 g silica, LP:EA 100:1, 70:1, 50:1, 10:1, 5:1), yielding 97 mg (92 %) of the title compound as slightly blue crystals.

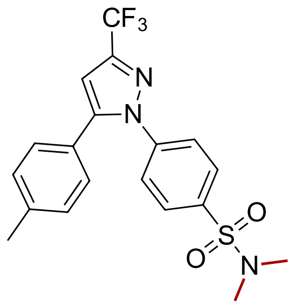

^{1}H NMR (400 MHz, $CDCl_3$) δ 7.80 – 7.72 (m, 2H), 7.54 – 7.46 (m, 2H), 7.17 (d, *J* = 7.8 Hz, 2H), 7.10 (d, *J* = 8.2 Hz, 2H), 6.75 (s, 1H), 2.70 (s, 6H), 2.38 (s, 3H).

^{13}C NMR (101 MHz, $CDCl_3$) δ 145.4, 144.2 (q, *J* = 38.5 Hz), 142.7, 139.9, 135.2, 129.8, 128.8, 128.8, 125.8, 125.7, 121.1 (q, *J* = 272.4 Hz), 106.36 (d, *J* = 2.1 Hz), 37.9, 21.4.

HRMS (ESI): m/z $[M+H]^+$ calcd. for: $C_{19}H_{19}F_3N_3O_2S$: 410.1145; found: 410.1145

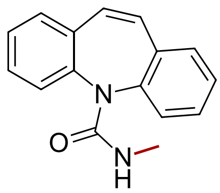

_N_-Methyl carbamazepine[16] (10) [CAS: 41359-02-8]

Prepared, following the general procedure from commercially available, bioactive carbamazepine with a reaction time of 16 h. Work-up procedure A was followed (10 g, LP:EA 20:1, 1:1, 1:2), yielding 50 mg (49 %) as a white powder.

^{1}H NMR (400 MHz, $CDCl_3$) δ 7.49 – 7.28 (m, 8H), 6.91 (s, 2H), 4.26 (s, 1H), 2.70 (d, *J* = 4.8 Hz, 3H).

^{13}C NMR (101 MHz, $CDCl_3$) δ 156.9, 140.1, 135.3, 130.5, 129.6, 129.4, 129.2, 127.6, 27.3.

HRMS (ESI): m/z $[M+H]^+$ calcd. for: $C_{16}H_{15}N_2O$: 251.1179; found: 251.1179

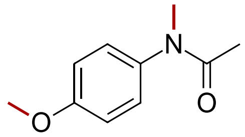

_N,O_-Dimethyl paracetamol[5] (11) [CAS: 35813-38-8]

Paracetamol (100 mg, 1 equiv), $PhMe_3NI$ (2 equiv), and Cs_2CO_3 (2 equiv) were placed in an 8 mL glass vial equipped with a magnetic stirring bar and a septum screw cap. Via a cannula, the vial was evacuated and backfilled with argon three times. Subsequently, toluene (0.23 M) was added *via* syringe, and the evacuation and backfilling cycles were repeated under vigorous stirring so that no boiling delay occurred. The septum screw cap was replaced with a closed Wheaton® screw cap. The inhomogeneous reaction mixture was heated to 120 °C in a metallic heating block for 4 h. The reaction was cooled to room temperature and another 2 equiv $PhMe_3NI$, and Cs_2CO_3 each were added. The reaction was again heated up to 120 °C and stirred at respective temperature for 18 h. Work-up procedure A was followed (10 g silica, LP:EA 2:1, 1:1), yielding 116 mg (98 %) of the title compound.

^{1}H NMR (400 MHz, $CDCl_3$) δ 7.29 – 7.21 (m, 1H), 7.11 – 7.02 (m, 1H), 3.98 (s, 1H), 3.37 (s, 1H), 2.00 (s, 1H).

^{13}C NMR (101 MHz, $CDCl_3$) δ 171.0, 158.9, 137.6, 128.2, 114.9, 55.5, 37.4, 22.4.

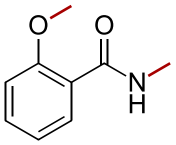

_O,N_-Dimethyl salicylamide[17] (12) [CAS: 3400-35-9]

Salicylamide (100 mg, 1 equiv), $PhMe_3NI$ (2 equiv), and Cs_2CO_3 (2 equiv) were placed in an 8 mL glass vial equipped with a magnetic stirring bar and a septum screw cap. Via a cannula, the vial was evacuated and backfilled with argon three times. Subsequently, toluene (0.23 M) was added *via* syringe, and the evacuation and backfilling cycles were repeated under vigorous stirring so that no boiling delay occurred. The septum screw cap was replaced with a closed Wheaton® screw cap. The inhomogeneous reaction mixture was heated to 120 °C in a metallic heating block for 3 h. The reaction was cooled to room temperature and

another 2 equiv $PhMe_3NI$, and Cs_2CO_3 each were added. The reaction was again heated up to 120 °C and stirred at respective temperature for 17 h. Work-up procedure A was followed (10 g silica, LP:EA 2:1, 1:1), yielding 93 mg (77 %) of the title compound as a colorless oil.

^{1}H NMR (400 MHz, $CDCl_3$) δ 8.18 (dq, *J* = 7.8, 2.1 Hz, 1H), 7.81 (s, 1H), 7.45 – 7.35 (m, 1H), 7.08 – 6.98 (m, 1H), 6.98 – 6.89 (m, 1H), 3.95 – 3.89 (m, 3H), 3.01 – 2.94 (m, 3H).

^{13}C NMR (101 MHz, $CDCl_3$) δ 166.0, 157.4, 132.6, 132.2, 121.5, 121.2, 111.2, 55.9, 26.5.

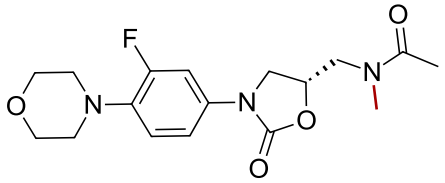

N-Methyl linezolid (13) [CAS: 3400-35-9]

Linezolid (100 mg, 1 equiv), $PhMe_3NI$ (2 equiv), and Cs_2CO_3 (2 equiv) were placed in an 8 mL glass vial equipped with a magnetic stirring bar and a septum screw cap. *Via* a cannula, the vial was evacuated and backfilled with argon three times. Subsequently, toluene (0.23 M) was added *via* syringe, and the evacuation and backfilling cycles were repeated under vigorous stirring so that no boiling delay occurred. The septum screw cap was replaced with a closed Wheaton® screw cap. The inhomogeneous reaction mixture was heated to 120 °C in a metallic heating block for 4 h. The reaction was cooled to room temperature and another 2 equiv $PhMe_3NI$, and Cs_2CO_3 each were added. The reaction was again heated up to 120 °C and stirred at respective temperature for 19 h. Work-up procedure A was followed (10 g silica, EA:MeOH 25:1), yielding 93 mg (77 %) of the title compound as a colorless oil.

^{1}H NMR (400 MHz, $CDCl_3$) δ 7.43 (dd, *J* = 14.4, 2.6 Hz, 1H), 7.13 – 7.02 (m, 1H), 6.90 (t, *J* = 9.1 Hz, 1H), 4.83 (qd, *J* = 6.7, 3.1 Hz, 1H), 4.00 (t, *J* = 8.9 Hz, 1H), 3.90 (dd, *J* = 14.5, 3.2 Hz, 1H), 3.87 – 3.80 (m, 4H), 3.73 (dd, *J* = 9.2, 6.9 Hz, 1H), 3.50 (dd, *J* = 14.5, 6.4 Hz, 1H), 3.16 (s, 3H), 3.06 – 2.99 (m, 4H), 2.10 (s, 3H).

^{13}C NMR (101 MHz, $CDCl_3$) δ 172.0, 156.8, 154.4, 136.5 (d, *J* = 9.0 Hz), 133.2 (d, *J* = 10.5 Hz), 118.9 (d, *J* = 4.3 Hz), 113.9 (d, *J* = 3.5 Hz), 107.6 (d, *J* = 26.5 Hz), 72.6, 67.0, 51.1 (d, *J* = 3.2 Hz), 50.9, 48.1, 38.6, 21.8.

^{19}F NMR (376 MHz, $CDCl_3$) δ -120.2 (dd, *J* = 14.4, 9.4 Hz).

HRMS (ESI): m/z $[M+H]^+$ calcd. for: $C_{17}H_{23}FN_3O_4$: 352.1667; found: 352.1667

Substrate scope ethylation

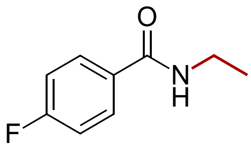

N-Ethyl-4-fluorobenzamide[18] (4a) [CAS: 772-18-9]

Prepared, following the general procedure from commercially available starting material with a reaction time of 12 h. Work-up procedure A was followed (10 g silica, DCM:MeOH 100:1, 50:1), yielding 94 mg (80 %) of the title compound as white crystals.

^{1}H NMR (400 MHz, $CDCl_3$) δ 7.81 – 7.71 (m, 2H), 7.10 – 7.00 (m, 2H), 6.43 (s, 1H), 3.45 (qd, *J* = 7.3, 5.6 Hz, 2H), 1.21 (t, *J* = 7.3 Hz, 3H).

^{13}C{^{1}H} NMR (101 MHz, $CDCl_3$) δ 166.5, 164.6 (d, *J* = 251.4 Hz), 131.0 (d, *J* = 3.2 Hz), 129.2 (d, *J* = 8.8 Hz), 115.5 (d, *J* = 21.8 Hz), 35.1, 14.9.

^{19}F{^{1}H} NMR (376 MHz) δ -108.7

N-Ethylbenzamide[19] (4b) [CAS: 614-17-5]

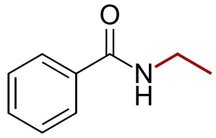

Prepared, following the general procedure from commercially available starting material with a reaction time of 12 h. Work-up procedure A was followed (10 g silica, DCM:MeOH 100:1, 50:1), yielding 95 mg (77 %) of the title compound as a colorless oil.

^{1}H NMR (400 MHz, CDCl$_3$) δ 7.80 – 7.72 (m, 2H), 7.49 – 7.41 (m, 1H), 7.41 – 7.30 (m, 2H), 6.56 (s, 1H), 3.45 (qd, *J* = 7.3, 5.6 Hz, 2H), 1.20 (t, *J* = 7.3 Hz, 3H).

^{13}C{^{1}H} NMR (101 MHz, CDCl$_3$) δ 167.6, 134.8, 131.2, 128.5, 126.9, 34.9, 14.9.

4-Bromo-N-ethylbenzamide[18] (4c) [CAS: 41882-25-1]

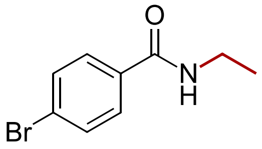

Prepared, following the general procedure from commercially available starting material with a reaction time of 12 h. Work-up procedure A was followed (10 g silica, DCM:MeOH 100:1, 50:1), yielding 71 mg (64 %) of the title compound as white crystals.

^{1}H NMR (400 MHz, CDCl$_3$) δ 7.66 – 7.58 (m, 2H), 7.57 – 7.48 (m, 2H), 6.30 (s, 1H), 3.46 (qd, *J* = 7.3, 5.6 Hz, 2H), 1.23 (t, *J* = 7.3 Hz, 3H).

^{13}C{^{1}H} NMR (101 MHz, CDCl$_3$) δ 166.6, 133.7, 131.8, 128.6, 126.0, 35.1, 14.9.

3-Bromo-N-ethylbenzamide[20] (4d) [CAS: 26819-10-3]

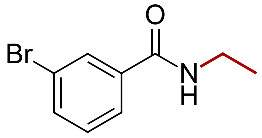

Prepared, following the general procedure from commercially available starting material with a reaction time of 17 h. Work-up procedure A was followed (10 g silica, DCM:MeOH 100:1), yielding 95 mg (85 %) of the title compound as a waxy solid.

^{1}H NMR (400 MHz, CDCl$_3$) δ 7.89 (t, *J* = 1.9 Hz, 1H), 7.66 (dt, *J* = 7.9, 1.3 Hz, 1H), 7.57 (ddd, *J* = 8.0, 2.0, 1.0 Hz, 1H), 7.25 (d, *J* = 15.7 Hz, 1H), 6.52 (s, 1H), 3.45 (qd, *J* = 7.3, 5.6 Hz, 2H), 1.22 (t, *J* = 7.3 Hz, 3H).

^{13}C{^{1}H} NMR (101 MHz, CDCl$_3$) 166.2, 136.8, 134.2, 130.2, 130.1, 125.5, 122.7, 35.1, 14.8.

2-Bromo-N-ethylbenzamide[20] (4e) [CAS: 80031-02-3]

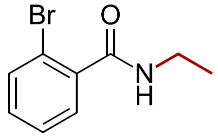

Prepared, following the general procedure from commercially available starting material with a reaction time of 17 h. Work-up procedure A was followed (10 g silica, DCM:MeOH 100:1), yielding 95 mg (85 %) of the title compound as a waxy solid.

^{1}H NMR (400 MHz, CDCl$_3$) 7.53 (d, *J* = 7.9 Hz, 1H), 7.45 (d, *J* = 7.3 Hz, 1H), 7.30 (t, *J* = 7.4 Hz, 1H), 7.22 (td, *J* = 7.7, 1.8 Hz, 1H), 6.13 (s, 1H), 3.50 – 3.38 (m, 2H), 1.22 (t, *J* = 7.2 Hz, 3H).

^{13}C{^{1}H} NMR (101 MHz, CDCl$_3$) 167.6, 138.1, 133.3, 131.1, 129.4, 127.5, 119.3, 35.0, 14.7.

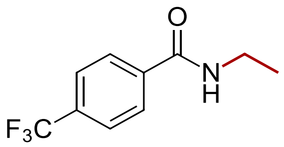

N-Ethyl-4-(trifluoromethyl) benzamide[21] (4f) [CAS: 1379773-10-0]

Prepared, following the general procedure from commercially available starting material with a reaction time of 18 h. Work-up procedure A was followed (10 g silica, DCM:MeOH 100:1), yielding 88 mg (76 %) of the title compound as white crystals.

^{1}H NMR (400 MHz, $CDCl_3$) δ 7.85 (d, *J* = 7.9 Hz, 2H), 7.63 (d, *J* = 7.8 Hz, 2H), 6.59 (s, 1H), 3.47 (qd, *J* = 7.3, 5.6 Hz, 2H), 1.23 (t, *J* = 7.3 Hz, 3H).

^{13}C{^{1}H} NMR (101 MHz, $CDCl_3$) δ 166.4, 138.1, 133.1 (q, *J* = 32.8 Hz), 127.4, 125.6 (q, *J* = 3.8 Hz), 124.5 (q, *J* = 271.6 Hz), 35.2, 14.8.

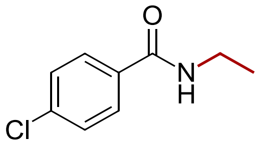

4-Chloro-N-ethylbenzamide[19] (4g) [CAS: 26930-17-6]

Prepared, following the general procedure from commercially available starting material with a reaction time of 12 h. Work-up procedure A was followed (10 g silica, DCM:MeOH 100:1, 50:1), yielding 95 mg (82 %) of the title compound as white crystals.

^{1}H NMR (400 MHz, $CDCl_3$) δ 7.73 – 7.64 (m, 2H), 7.39 – 7.30 (m, 2H), 6.46 (s, 1H), 3.44 (qd, *J* = 7.3, 5.6 Hz, 2H), 1.21 (t, *J* = 7.3 Hz, 3H).

^{13}C{^{1}H} NMR (101 MHz, $CDCl_3$) δ 166.5, 137.5, 133.2, 128.7, 128.4, 35.1, 14.9.

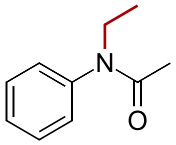

N-Ethylacetanilide[22] (4h) [CAS: 529- 65-7]

Prepared, following the general procedure from commercially available starting material with a reaction time of 17 h. Work-up procedure A was followed (10 g silica, DCM:MeOH 50:1), yielding 108 mg (92 %) of the title compound as slightly blue oil.

^{1}H NMR (400 MHz, $CDCl_3$) δ 7.43 – 7.34 (m, 2H), 7.34 – 7.26 (m, 1H), 7.16 – 7.08 (m, 2H), 3.71 (q, *J* = 7.1 Hz, 2H), 1.78 (s, 3H), 1.07 (t, *J* = 7.2 Hz, 3H).

^{13}C{^{1}H} NMR (101 MHz, $CDCl_3$) δ 169.9, 142.9, 129.6, 128.2, 127.8, 43.8, 22.8, 13.0.

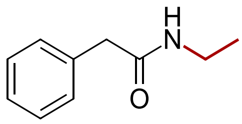

N-Ethylphenylacetamide[6] (4i) [CAS: 5465-00-9]

Prepared, following the general procedure from commercially available starting material with a reaction time of 17 h. Work-up procedure A was followed (10 g silica, DCM:MeOH 50:1), yielding 77 mg (64 %) of the title compound a colorless oil.

^{1}H NMR (400 MHz, $CDCl_3$) δ 7.51 – 7.09 (m, 6H), 5.66 (s, 1H), 3.54 (s, 2H), 3.23 (qd, *J* = 7.3, 5.6 Hz, 2H), 1.05 (t, *J* = 7.3 Hz, 3H)

^{13}C{^{1}H} NMR (101 MHz, $CDCl_3$) δ 170.9, 135.1, 129.4, 128.9, 127.2, 43.8, 34.5, 14.7.

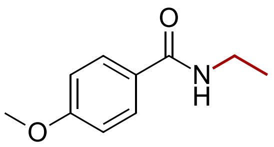

_N_-Ethyl-4-methoxybenzamide[23] (4j) [CAS: 7403-41-0]

Prepared, following the general procedure from commercially available starting material with a reaction time of 18 h. Work-up procedure A was followed (10 g silica, DCM:MeOH 100:1, 50:1), yielding 90 mg (76 %) of the title compound as amorphous solid.

^{1}H NMR (400 MHz, $CDCl_3$) δ 7.73 (d, *J* = 8.8 Hz, 2H), 6.91 (d, *J* = 8.8 Hz, 2H), 6.06 (s, 1H), 3.84 (s, 3H), 3.48 (qd, *J* = 7.2, 5.6 Hz, 2H), 1.24 (t, *J* = 7.3 Hz, 3H).

^{13}C{^{1}H} NMR (101 MHz, $CDCl_3$) δ 167.0, 162.1, 128.7, 127.2, 113.8, 55.5, 34.9, 15.1.

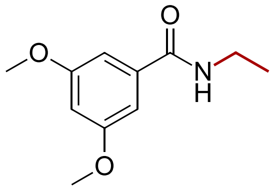

_N_-Ethyl-3,5-dimethoxbenzamide (4k) [CAS: 120301-09-9]

Prepared, following the general procedure from commercially available starting material with a reaction time of 17 h. Work-up procedure A was followed (10 g silica, DCM:MeOH 100:1), yielding 88 mg (78 %) of the title compound an amorphous solid..

^{1}H NMR (400 MHz, $CDCl_3$) δ 6.87 (d, *J* = 2.3 Hz, 2H), 6.56 – 6.50 (m, 1H), 6.33 (s, 1H), 3.82 – 3.74 (m, 6H), 3.44 (qd, *J* = 7.2, 5.9 Hz, 2H), 1.21 (t, *J* = 7.3 Hz, 3H).

^{13}C{^{1}H} NMR (101 MHz, $CDCl_3$) δ 167.3, 160.9, 137.1, 104.8, 103.4, 55.6, 35.0, 14.9.

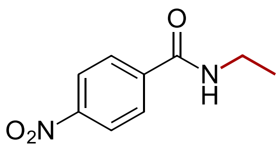

_N_-Ethyl-4-nitrobenzamide[24] (4l) [CAS: 50445-50-6]

Prepared, following the general procedure from commercially available starting material with a reaction time of 11 h. Work-up procedure A was followed (10 g silica, DCM:MeOH 100:1, 50:1), yielding 93 mg (81 %) of the title compound as yellow amorphous solid.

^{1}H NMR (400 MHz, $CDCl_3$) δ 8.22 (d, *J* = 8.9 Hz, 2H), 7.92 (d, *J* = 8.8 Hz, 2H), 6.63 (s, 1H), 3.48 (qd, *J* = 7.3, 5.6 Hz, 2H), 1.24 (t, *J* = 7.3 Hz, 3H).

^{13}C{^{1}H} NMR (101 MHz, $CDCl_3$) δ 165.5, 149.5, 140.4, 128.2, 123.8, 35.4, 14.7.

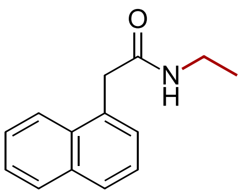

_N_-Ethyl-α-naphthylacetamide (4m) [CAS: 1140-55-2]

Prepared, following the general procedure from commercially available starting material with a reaction time of 12 h. Work-up procedure A was followed (10 g silica, LP:EA 4:1, 2:3, 1:10), yielding 66 mg (59 %) of the title compound as a colorless waxy solid.

^{1}H NMR (400 MHz, $CDCl_3$) δ 8.00 – 7.91 (m, 1H), 7.91 – 7.84 (m, 1H), 7.84 – 7.75 (m, 1H), 7.58 – 7.49 (m, 2H), 7.49 – 7.36 (m, 2H), 5.36 (s, 1H), 4.00 (s, 2H), 3.16 (qd, *J* = 7.2, 5.7 Hz, 2H), 0.93 (t, *J* = 7.3 Hz, 3H).

^{13}C{^{1}H} NMR (101 MHz, $CDCl_3$) δ 170.7, 134.0, 132.1, 131.3, 128.8, 128.5, 128.4, 126.8, 126.2, 125.7, 123.9, 41.9, 34.5, 14.7.

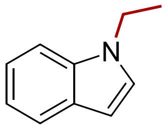

1-Ethylindole[25] (6a) [CAS: 10604-59-8]

Prepared, following the general procedure from commercially available starting material with a reaction time of 18 h. Work-up procedure B was followed (10 g silica, LP:EA 30:1), yielding 97 mg (79 %) of the title compound as a colorless oil.

^{1}H NMR (400 MHz, $CDCl_3$) δ 7.79 – 7.68 (m, 1H), 7.48 – 7.41 (m, 1H), 7.36 – 7.17 (m, 3H), 6.64 – 6.57 (m, 1H), 4.23 (q, *J* = 7.3 Hz, 2H), 1.54 (t, *J* = 7.3 Hz, 3H).

^{13}C{^{1}H} NMR (101 MHz, $CDCl_3$) δ 135.7, 128.7, 127.0, 121.4, 121.0, 119.3, 109.3, 101.1, 41.0, 15.5.

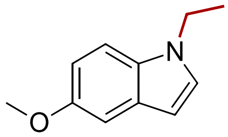

1-Ethyl-5-methoxyindole[25] (6b) [CAS: 46182-32-5]

Prepared, following the general procedure from commercially available starting material with a reaction time of 21 h. Work-up procedure B was followed (10 g silica, LP:EA 50:1), yielding 103 mg (87 %) of the title compound as a waxy solid.

^{1}H NMR (400 MHz, $CDCl_3$) δ 7.39 (d, *J* = 8.9 Hz, 1H), 7.25 (dd, *J* = 9.5, 2.8 Hz, 2H), 7.04 (dd, *J* = 8.9, 2.5 Hz, 1H), 6.57 (d, *J* = 3.2 Hz, 1H), 4.27 (q, *J* = 7.3 Hz, 2H), 4.00 (s, 3H), 1.59 (t, *J* = 7.3 Hz, 3H).

^{13}C{^{1}H} NMR (101 MHz, $CDCl_3$) δ 154.0, 131.1, 129.0, 127.5, 111.8, 110.0, 102.6, 100.6, 55.9, 41.1, 15.5.

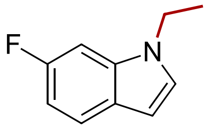

1-Ethyl-5-fluoroindole (6c) [CAS: 613684-38-1]

Prepared, following the general procedure from commercially available starting material with a reaction time of 18 h. Work-up procedure B was followed (10 g silica, LP:EA 30:1), yielding 111 mg (94 %) of the title compound as a colorless oil.

^{1}H NMR (400 MHz, $CDCl_3$) δ 7.57 (dd, *J* = 8.6, 5.4 Hz, 1H), 7.12 (d, *J* = 3.2 Hz, 1H), 7.09 – 6.99 (m, 1H), 6.96 – 6.84 (m, 1H), 6.51 (dd, *J* = 3.2, 0.9 Hz, 1H), 4.12 (q, *J* = 7.3 Hz, 2H), 1.47 (t, *J* = 7.3 Hz, 3H).

^{13}C{^{1}H} NMR (101 MHz, $CDCl_3$) δ 159.8 (d, *J* = 236.9 Hz), 135.8 (d, *J* = 12.0 Hz), 127.5 (d, *J* = 3.6 Hz), 125.1, 121.7 (d, *J* = 10.2 Hz), 108.0 (d, *J* = 24.6 Hz), 101.3, 95.7 (d, *J* = 26.2 Hz), 41.2, 15.3.

^{19}F{^{1}H} NMR (376 MHz, $CDCl_3$) δ -121.3

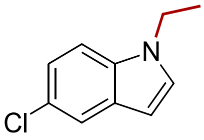

5-Chloro-1-ethylindole[25] (6d) [CAS: 112194-57-7]

Prepared, following the general procedure from commercially available starting material with a reaction time of 18 h. Work-up procedure B was followed (10 g silica, LP:EA 30:1), yielding 104 mg (90 %) of the title compound as a colorless oil.

^{1}H NMR (400 MHz, $CDCl_3$) δ 7.64 – 7.59 (m, 1H), 7.32 – 7.22 (m, 1H), 7.22 – 7.10 (m, 2H), 6.45 (dd, *J* = 3.2, 0.8 Hz, 1H), 4.16 (q, *J* = 7.3 Hz, 2H), 1.47 (t, *J* = 7.3 Hz, 3H).

^{13}C{^{1}H} NMR (101 MHz, $CDCl_3$) δ 134.2, 129.7, 128.4, 125.0, 121.7, 120.3, 110.3, 100.8, 41.2, 15.5.

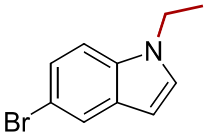

5-Bromo-1-ethylindole[26] (6e) [CAS: 195253-49-7]

Prepared, following the general procedure from commercially available starting material with a reaction time of 18 h. Work-up procedure B was followed (10 g silica, LP:EA 30:1), yielding 88 mg (78 %) of the title compound as an amorphous solid.

^{1}H NMR (400 MHz, $CDCl_3$) δ 7.72 (t, *J* = 2.3 Hz, 1H), 7.28 – 7.13 (m, 2H), 7.07 (d, *J* = 3.2 Hz, 1H), 6.48 – 6.28 (m, 1H), 4.08 (q, *J* = 7.3 Hz, 2H), 1.40 (t, *J* = 7.3 Hz, 3H).

^{13}C{^{1}H} NMR (101 MHz, $CDCl_3$) δ 134.4, 130.4, 128.2, 124.2, 123.4, 112.6, 110.8, 100.7, 41.2, 15.4.

1-Ethyl-5-iodoindole (6f) [CAS: 2413600-46-9]

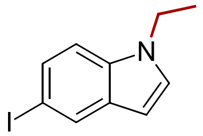

Prepared, following the general procedure from commercially available starting material with a reaction time of 17 h. Work-up procedure B was followed (10 g silica, LP:EA 30:1), yielding 100 mg (94 %) of the title compound as a colorless oil.

^{1}H NMR (400 MHz, CDCl₃) δ 7.98 (d, *J* = 1.7 Hz, 1H), 7.46 (dd, *J* = 8.6, 1.7 Hz, 1H), 7.13 (dd, *J* = 8.7, 0.8 Hz, 1H), 7.08 (d, *J* = 3.2 Hz, 1H), 6.43 (dd, *J* = 3.1, 0.9 Hz, 1H), 4.14 (q, *J* = 7.3 Hz, 2H), 1.46 (t, *J* = 7.3 Hz, 3H).

^{13}C{^{1}H} NMR (101 MHz, CDCl₃) δ 134.8, 131.3, 129.8, 129.6, 127.9, 111.3, 100.5, 82.8, 41.2, 15.4.

1-Ethyl-5-nitroindole (6g) [CAS: 193977-99-0]

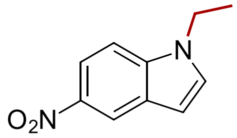

Prepared, following the general procedure from commercially available starting material with a reaction time of 18 h. Work-up procedure B was followed (10 g silica, LP:EA 30:1), yielding 109 mg (93 %) of the title compound as yellow solid.

^{1}H NMR (400 MHz, CDCl₃) δ 8.56 (d, *J* = 2.2 Hz, 1H), 8.09 (dd, *J* = 9.1, 2.3 Hz, 1H), 7.34 (dt, *J* = 9.1, 0.7 Hz, 1H), 7.27 (s, 1H), 6.66 (dd, *J* = 3.3, 0.9 Hz, 1H), 4.22 (q, *J* = 7.3 Hz, 2H), 1.49 (t, *J* = 7.3 Hz, 3H).

^{13}C{^{1}H} NMR (101 MHz, CDCl₃) δ 141.5, 138.6, 130.3, 127.8, 118.3, 117.1, 109.1, 104.0, 41.6, 15.4.

1-Ethyl-2-indolecarbaldehyde (6h) [CAS: 40913-43-7]

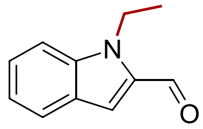

Prepared, following the general procedure from commercially available starting material with a reaction time of 18 h. Work-up procedure B was followed (10 g silica, LP:EA 10:1), yielding 74 mg (64 %) of the title compound as yellow oil.

^{1}H NMR (400 MHz, CDCl₃) δ 9.89 (s, 1H), 7.75 (dt, *J* = 8.2, 1.0 Hz, 1H), 7.47 – 7.37 (m, 2H), 7.26 (d, *J* = 0.5 Hz, 1H), 7.19 (dt, *J* = 8.0, 3.9 Hz, 1H), 4.62 (q, *J* = 7.2 Hz, 2H), 1.40 (t, *J* = 7.2 Hz, 3H).

^{13}C{^{1}H} NMR (101 MHz, CDCl₃) δ182.6, 139.9, 135.1, 126.9, 126.5, 123.5, 120.9, 117.9, 110.4, 39.7, 15.6.

Ethyl 1-ethyl-2-indolecarboxylate[27] (6i) [CAS: 40913-41-5]

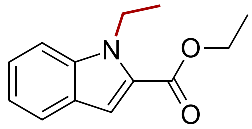

Prepared, following the general procedure from commercially available starting material with a reaction time of 18 h. Work-up procedure B was followed (10 g silica, LP:EA 30:1), yielding 100 mg (87 %) of the title compound as off-white solid.

^{1}H NMR (400 MHz, CDCl₃) δ 7.64 (dt, *J* = 8.0, 1.0 Hz, 1H), 7.35 (dq, *J* = 8.5, 1.0 Hz, 1H), 7.32 – 7.24 (m, 2H), 7.15 – 7.06 (m, 1H), 4.58 (q, *J* = 7.1 Hz, 2H), 4.34 (q, *J* = 7.1 Hz, 2H), 1.41 – 1.32 (m, 6H).

^{13}C{^{1}H} NMR (101 MHz, CDCl₃) δ 162.0, 138.7, 127.3, 126.1, 124.9, 122.7, 120.5, 110.4, 110.3, 60.5, 39.6, 15.70, 14.4.

Methyl 1-ethyl-5-indolecarboxylate (6j) [CAS: 1205532-00-8]

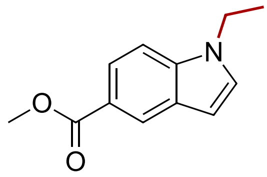

Prepared, following the general procedure from commercially available starting material with a reaction time of 14 h. Work-up procedure B was followed (10 g silica, DCM:MeOH 100:1), yielding 105 mg (91 %) of the title compound as a colorless oil.

^{1}H NMR (400 MHz, $CDCl_3$) δ 8.42 (dd, *J* = 1.7, 0.7 Hz, 1H), 7.93 (dd, *J* = 8.7, 1.7 Hz, 1H), 7.34 (dt, *J* = 8.7, 0.8 Hz, 1H), 7.16 (d, *J* = 3.2 Hz, 1H), 6.60 (dd, *J* = 3.2, 0.9 Hz, 1H), 4.16 (q, *J* = 7.3 Hz, 2H), 3.94 (s, 3H), 1.45 (t, *J* = 7.3 Hz, 3H).

^{13}C{^{1}H} NMR (101 MHz, $CDCl_3$) δ 168.3, 138.2, 128.5, 128.1, 124.0, 122.7, 121.3, 108.9, 102.7, 51.8, 41.1, 15.4.

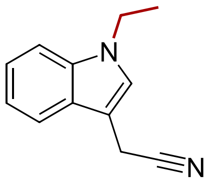

(1-Ethyl-3-indolyl)acetonitrile (6k) [CAS: 851041-59-3]

Prepared, following the general procedure from commercially available starting material with a reaction time of 18 h. Work-up procedure B was followed (10 g silica, LP:EA 40:1, 20:1, 10:1), yielding 108 mg (93 %) of the title compound as a yellow oil.

^{1}H NMR (400 MHz, $CDCl_3$) δ 7.72 (dt, *J* = 7.9, 1.0 Hz, 1H), 7.50 (dt, *J* = 8.2, 1.0 Hz, 1H), 7.46 – 7.37 (m, 1H), 7.37 – 7.28 (m, 1H), 7.26 (d, *J* = 1.1 Hz, 1H), 4.25 (q, *J* = 7.3 Hz, 2H), 3.92 (d, *J* = 1.1 Hz, 2H), 1.58 (t, *J* = 7.3 Hz, 3H).

^{13}C{^{1}H} NMR (101 MHz, $CDCl_3$) δ 136.1, 126.5, 125.6, 122.2, 119.6, 118.3, 118.2, 109.7, 103.0, 40.9, 15.4, 14.2.

NMR-Spectra

***N*-Methyl -4-fluorobenzamide (2a)**

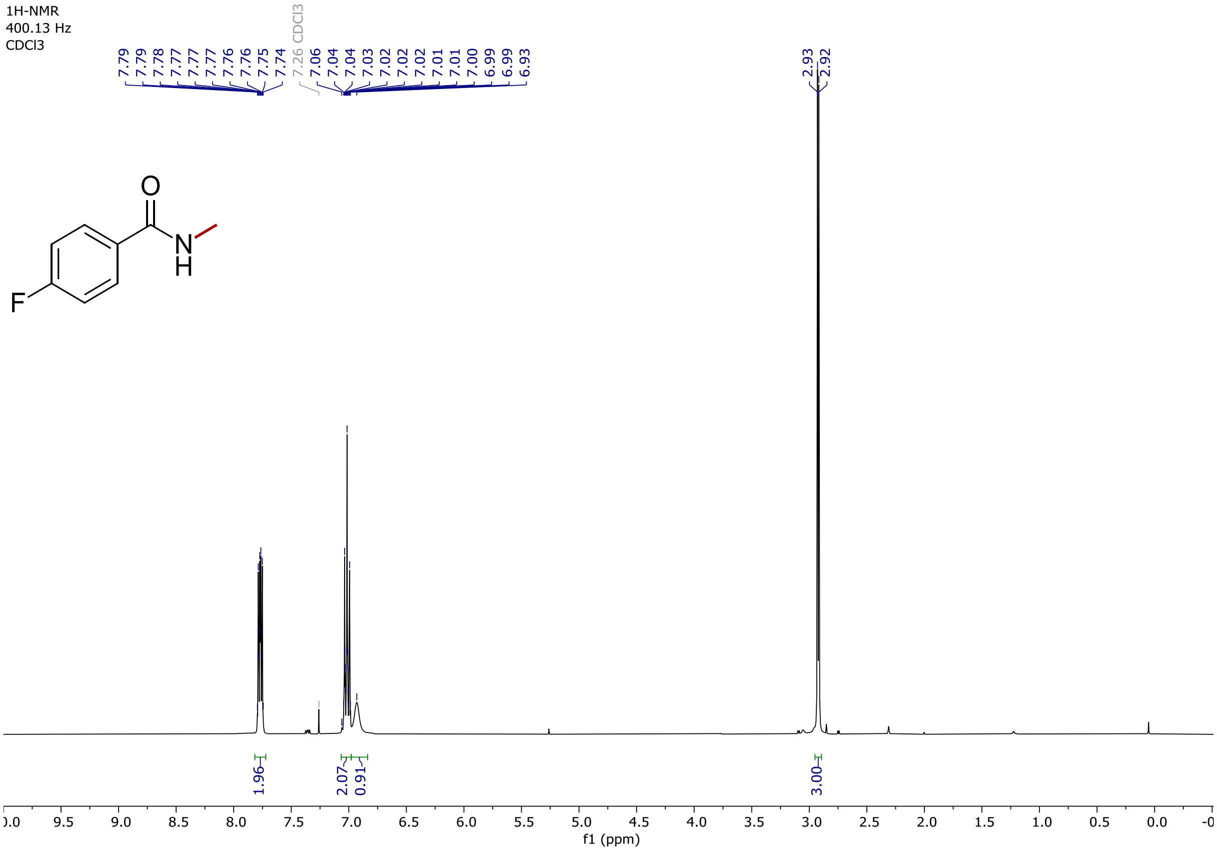

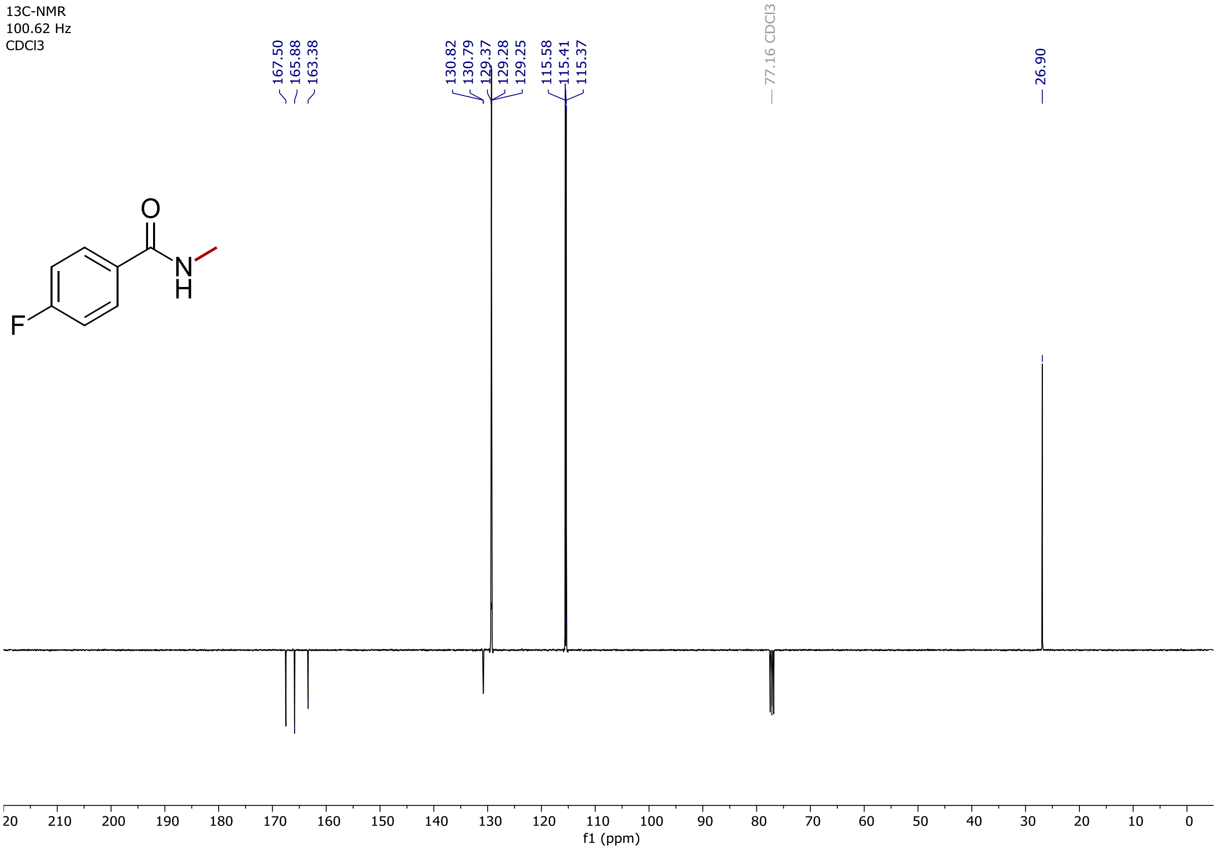

N-Methylbenzamide (2b)

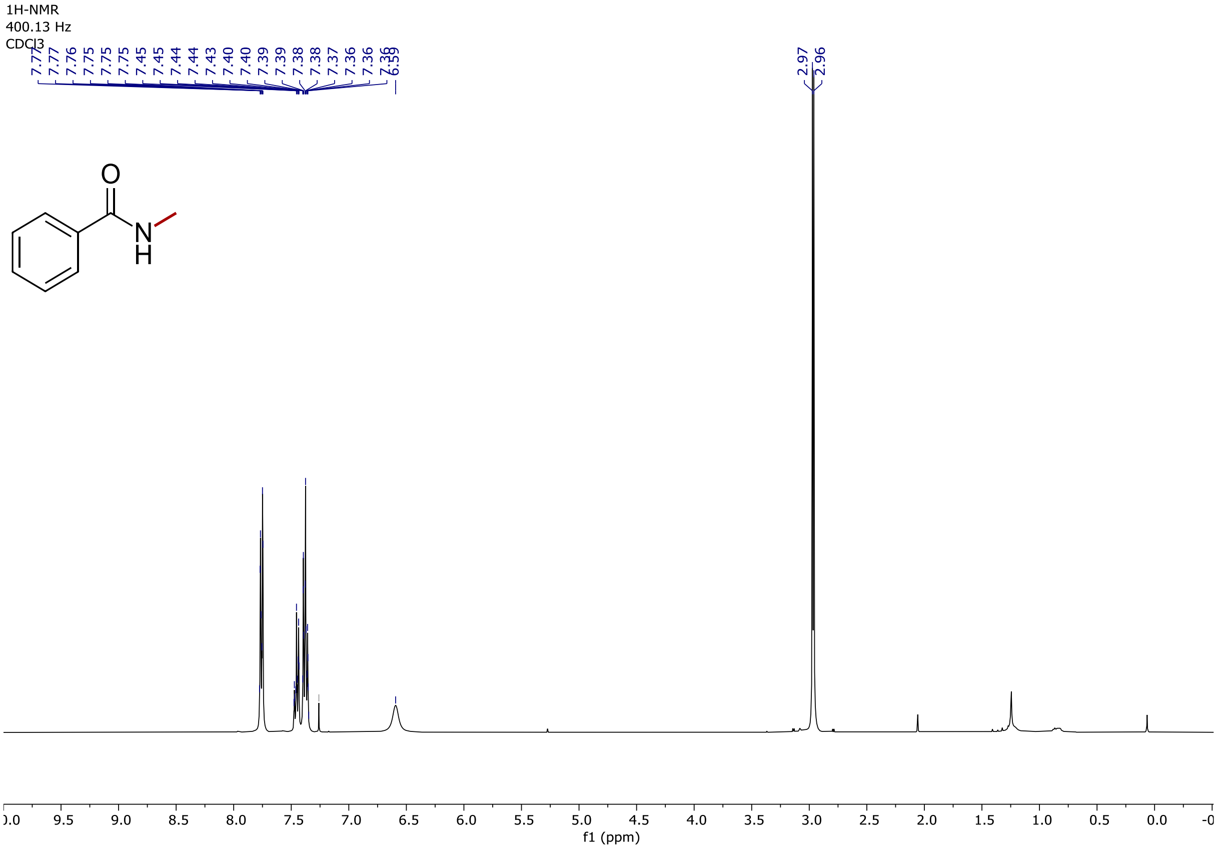

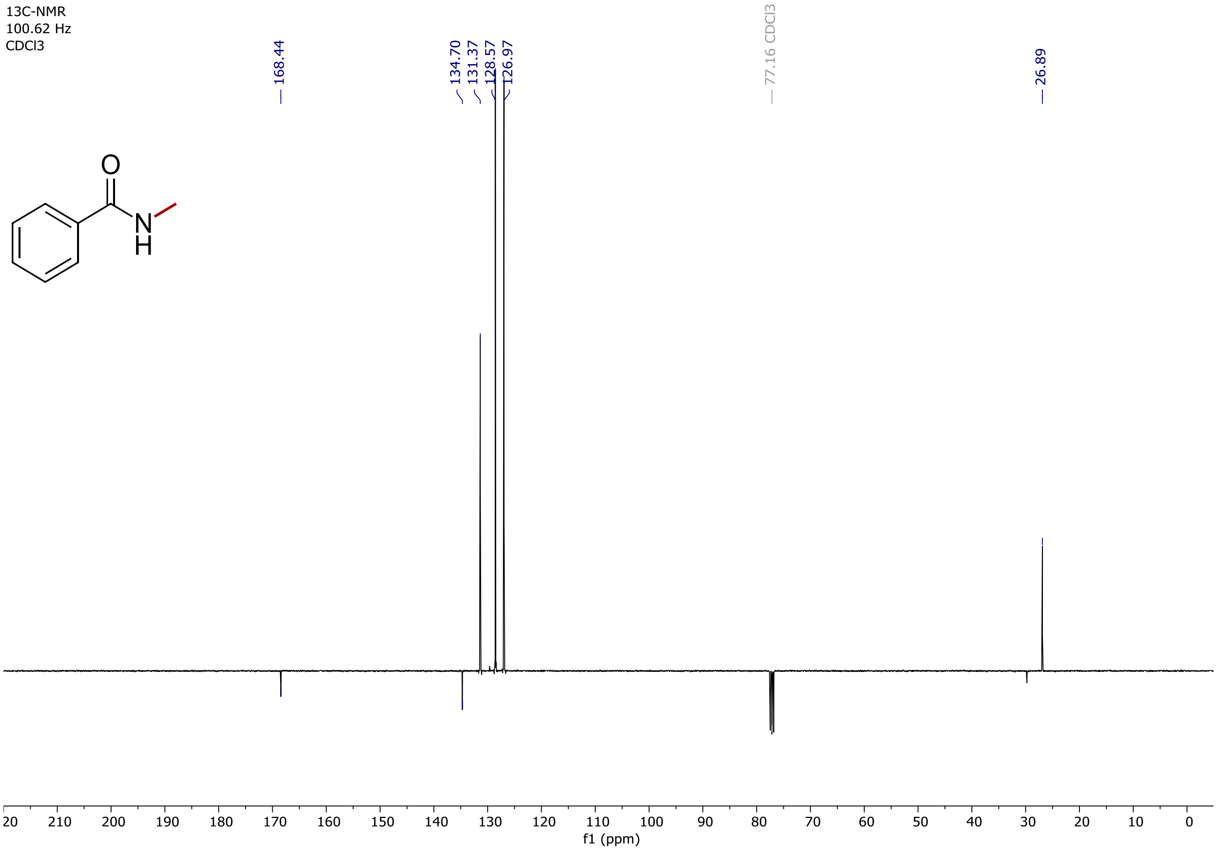

N-Methyl-4-bromobenzamide (2c)

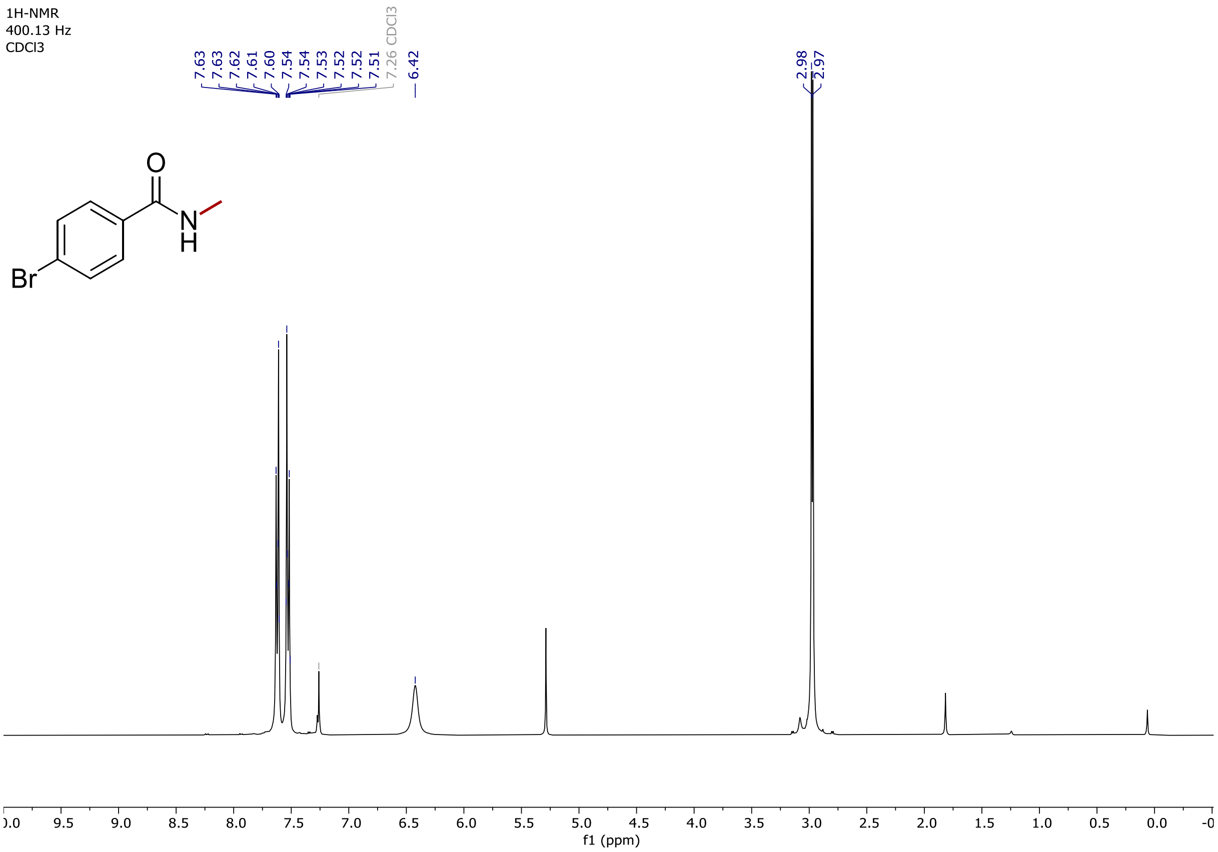

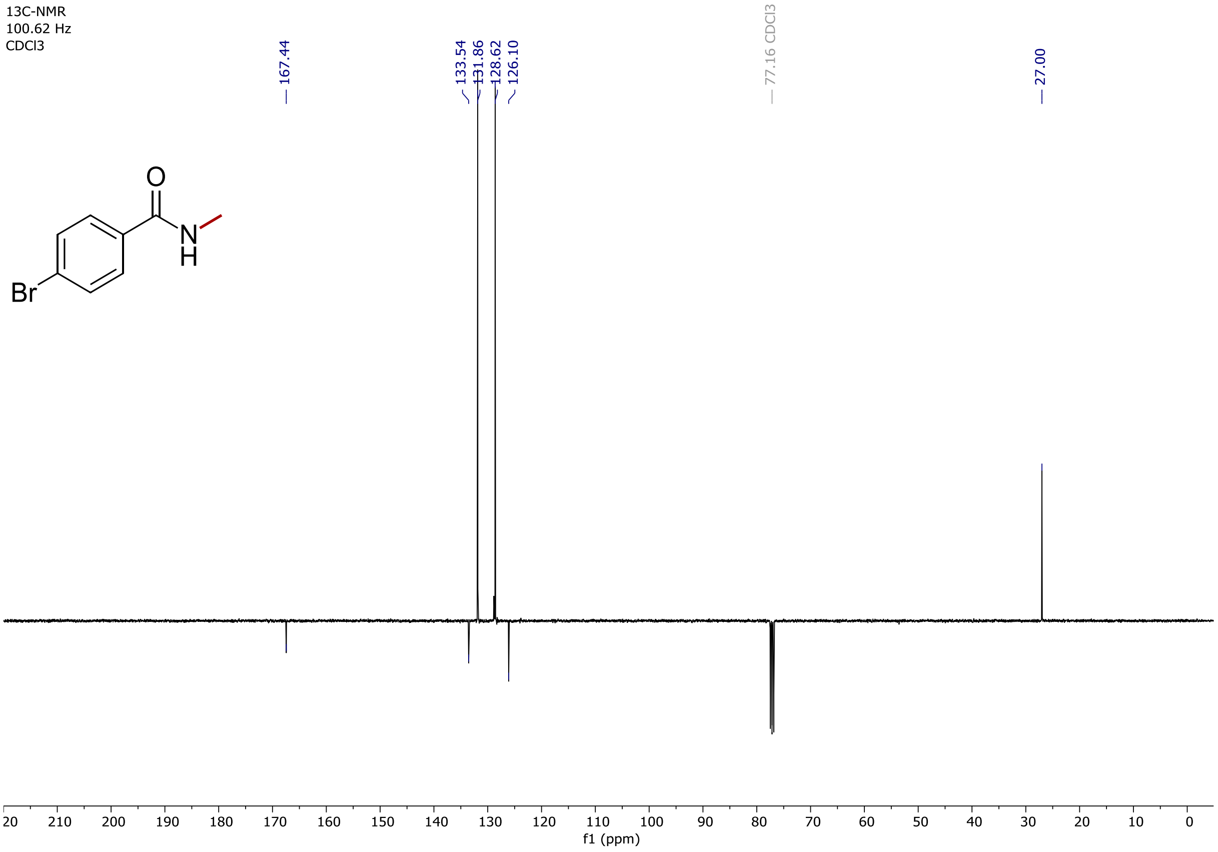

3-Bromo-*N*-methylbenzamide (2d)

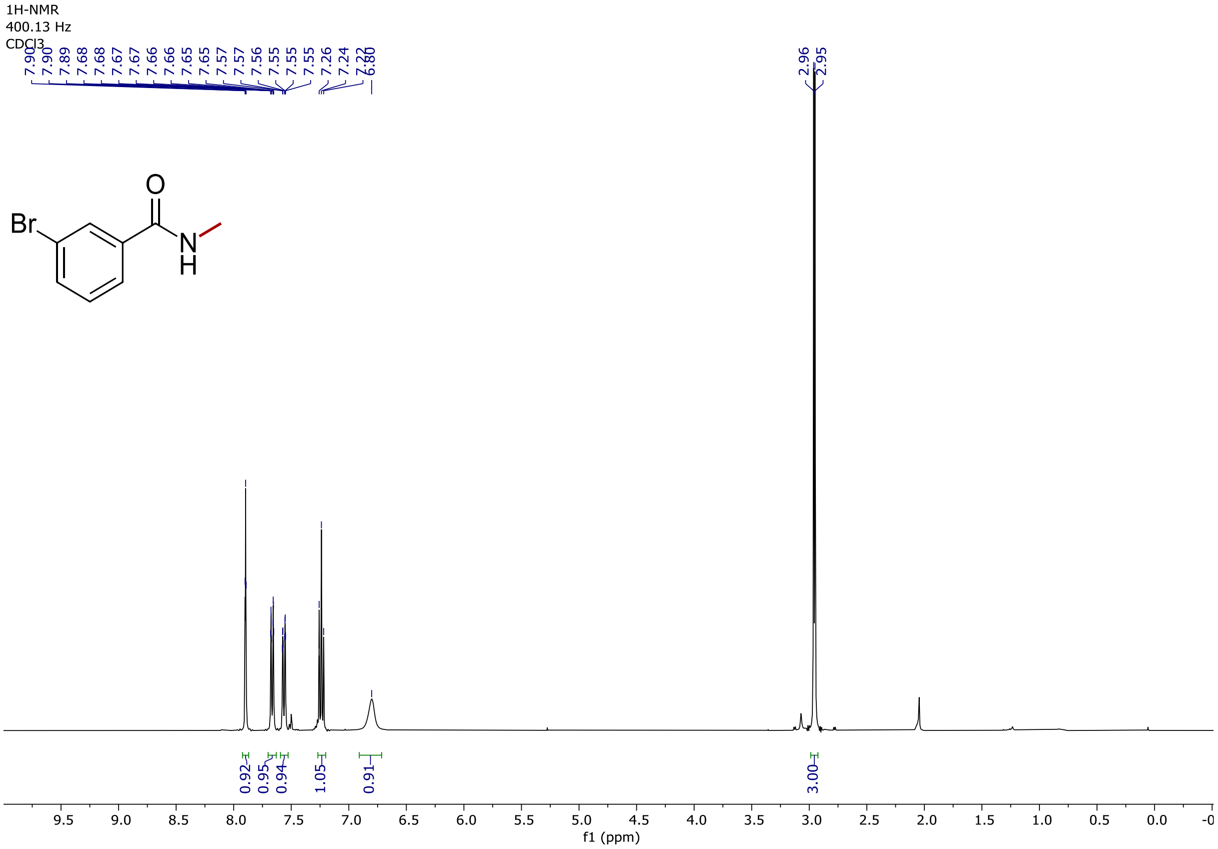

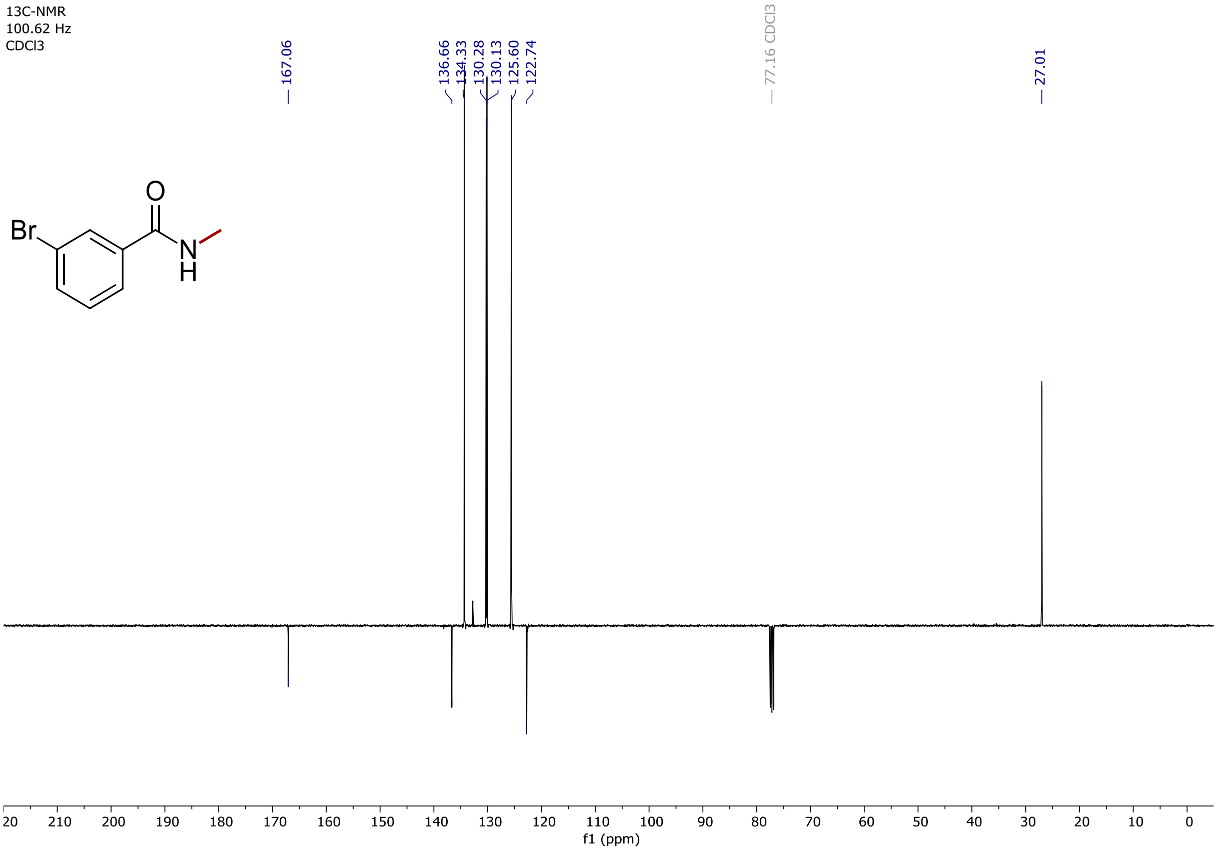

2-Bromo-*N*-methylbenzamide (2e)

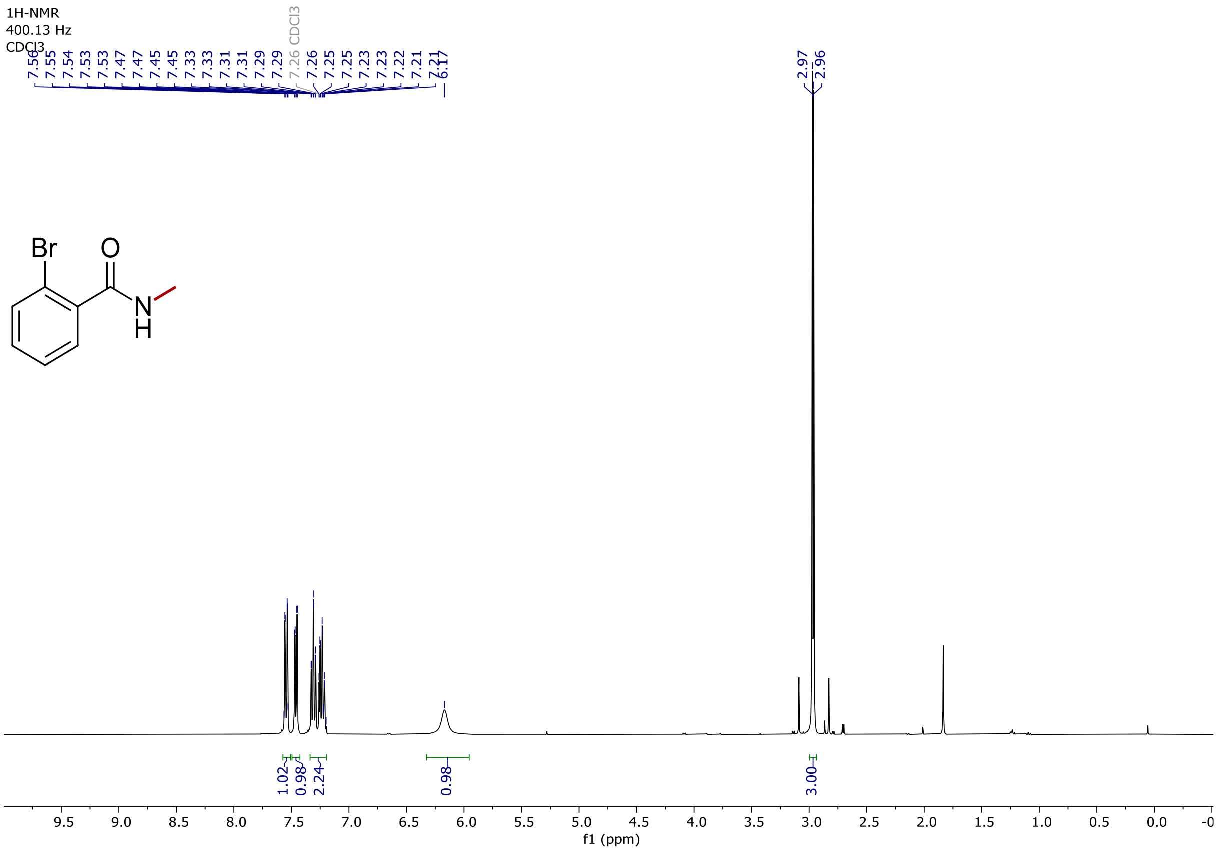

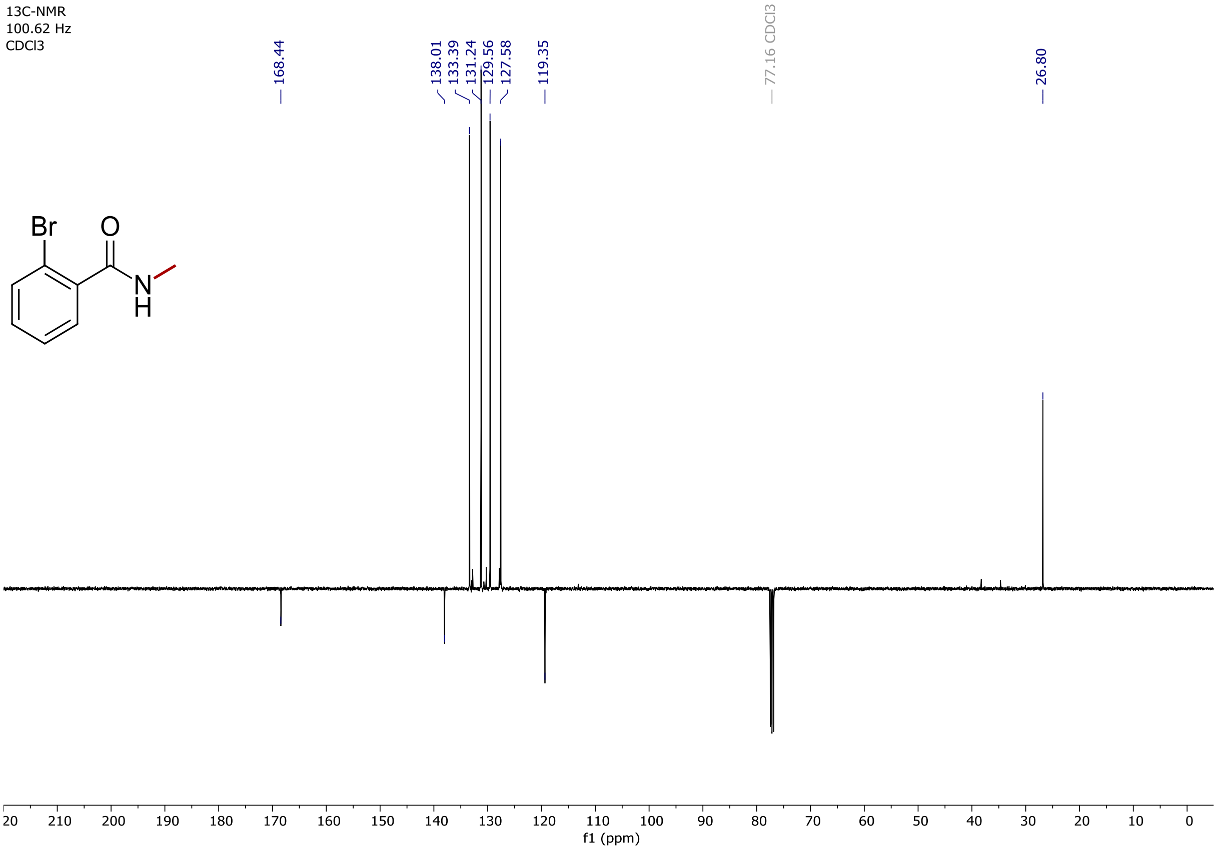

N-Methyl-4-(trifluoromethyl)benzamide (2f)

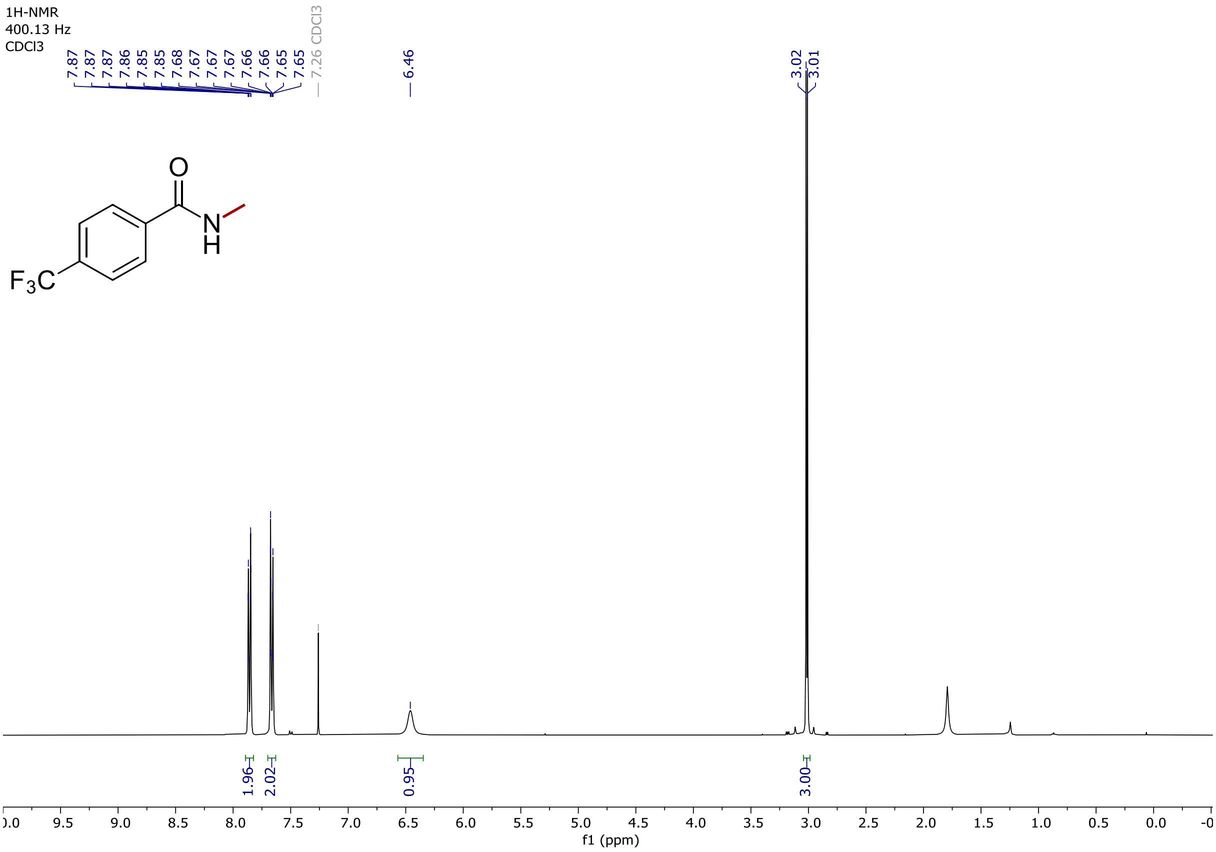

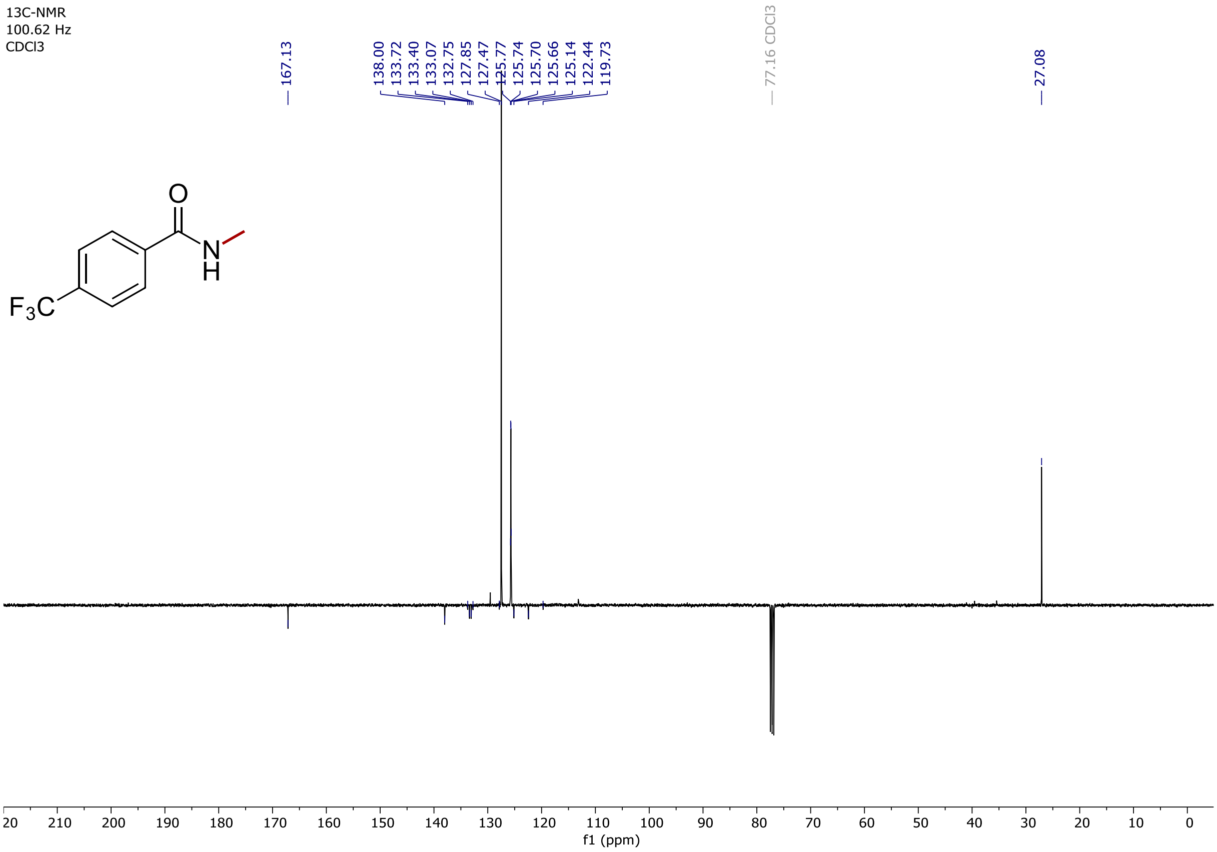

19F-NMR
376.46 Hz
CDCl3

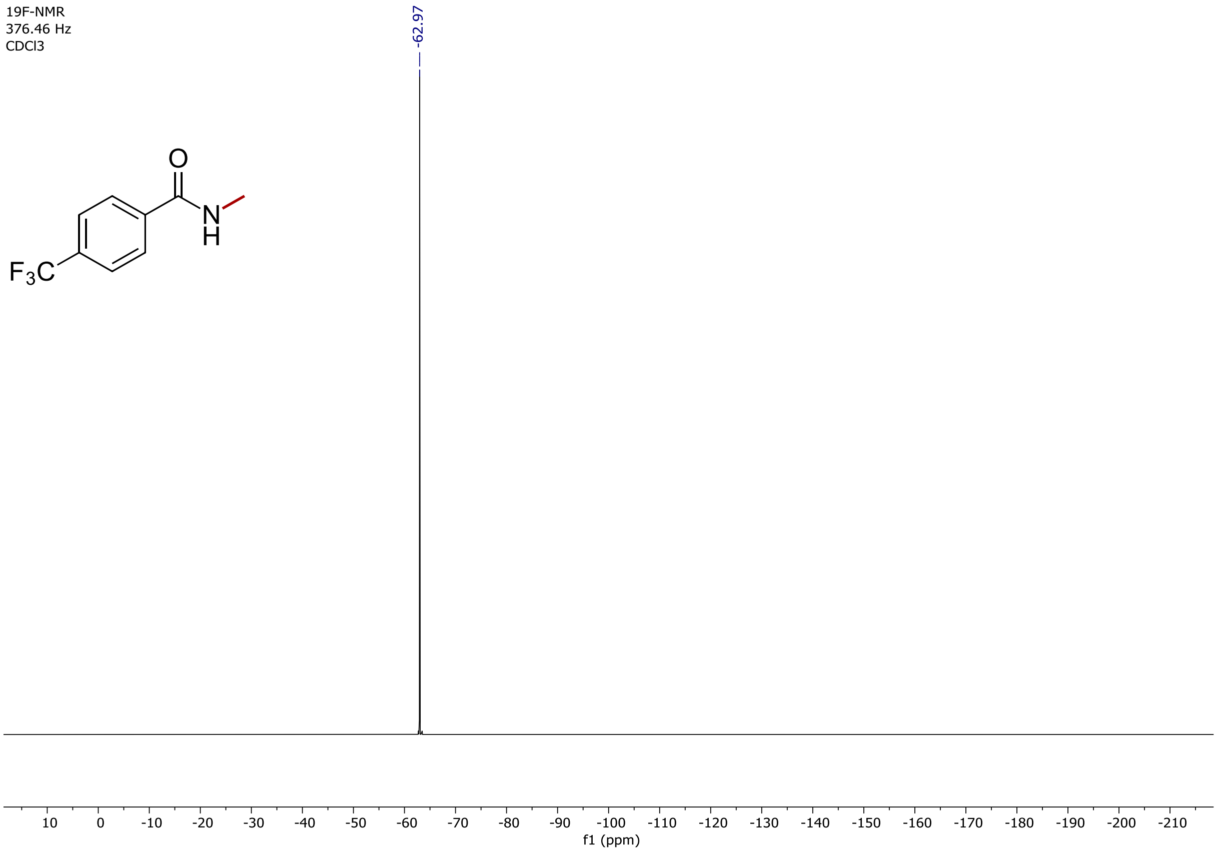

4-Chloro-*N*-methylbenzamide (2g)

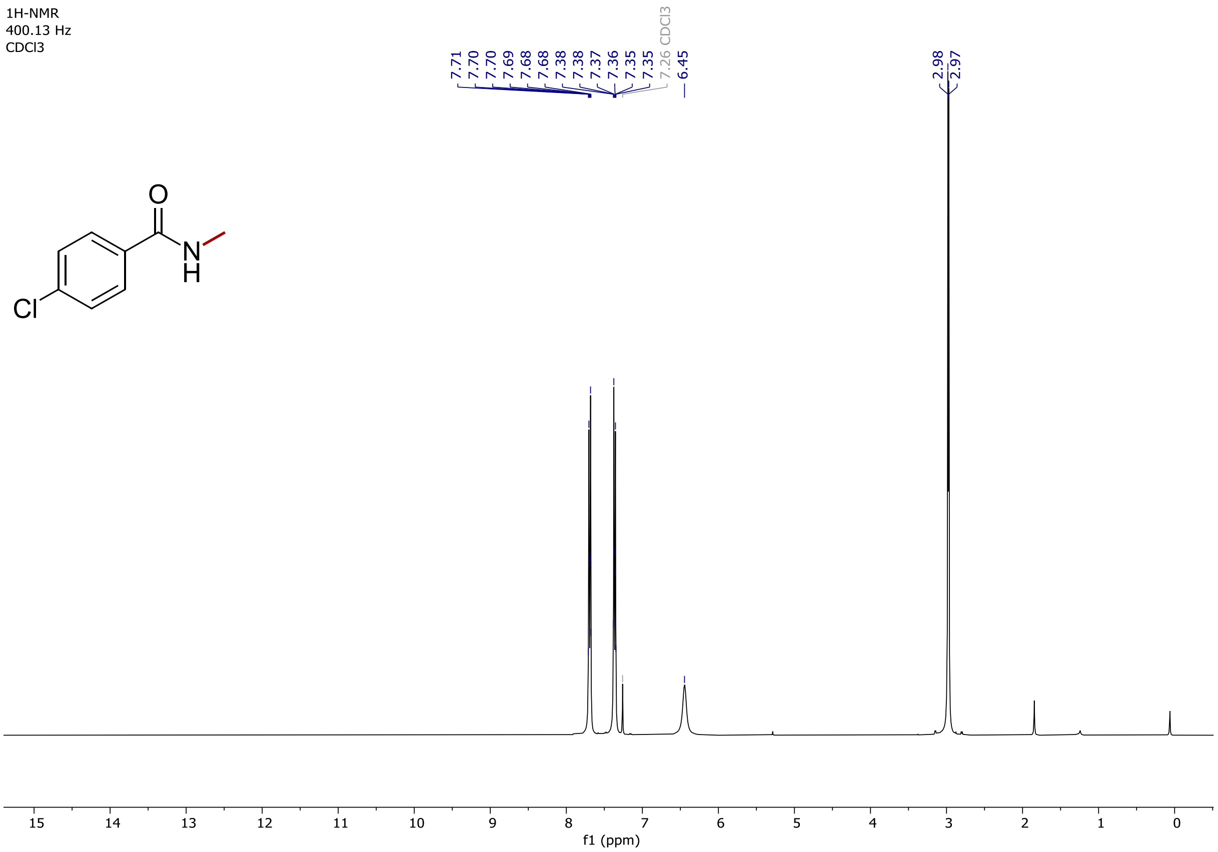

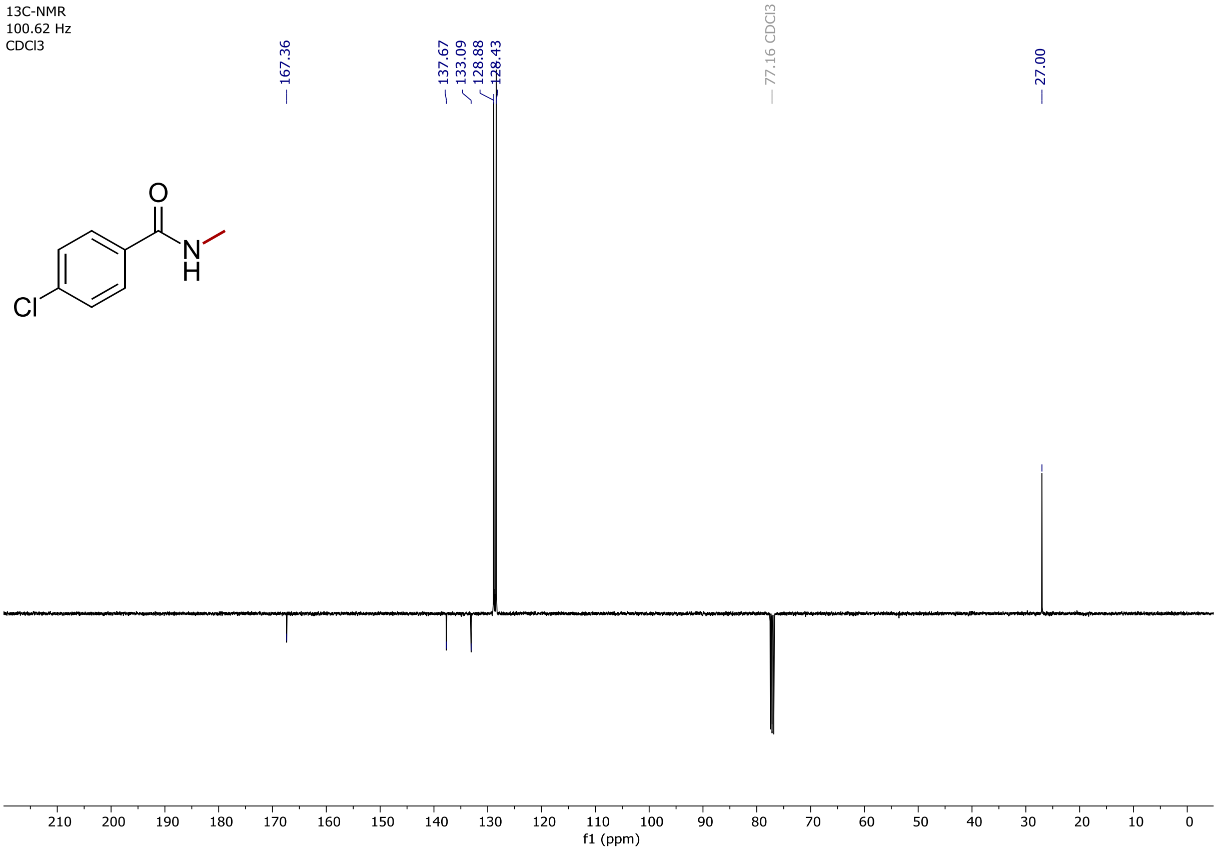

N-Acetyl-*N*-methylaniline (2h)

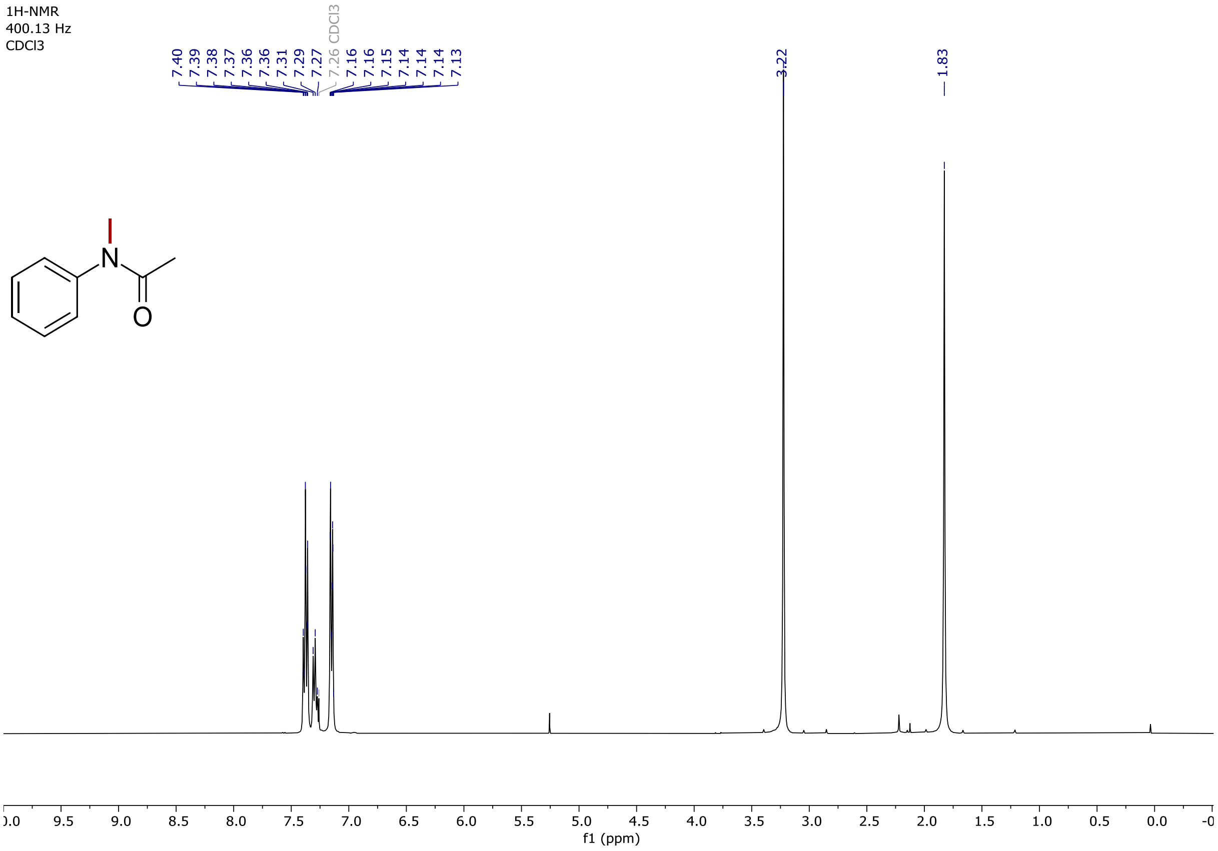

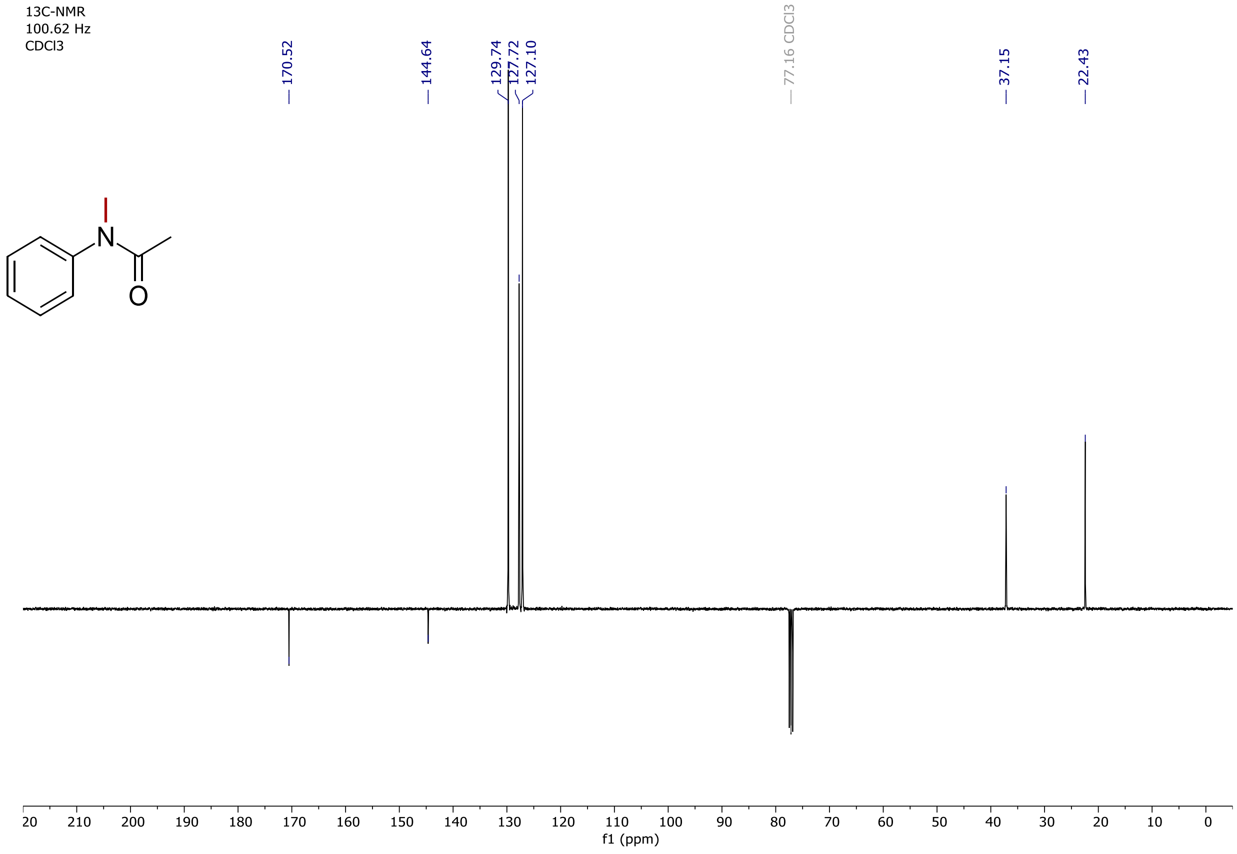

***N*-Methylphenylacetamide (2i)**

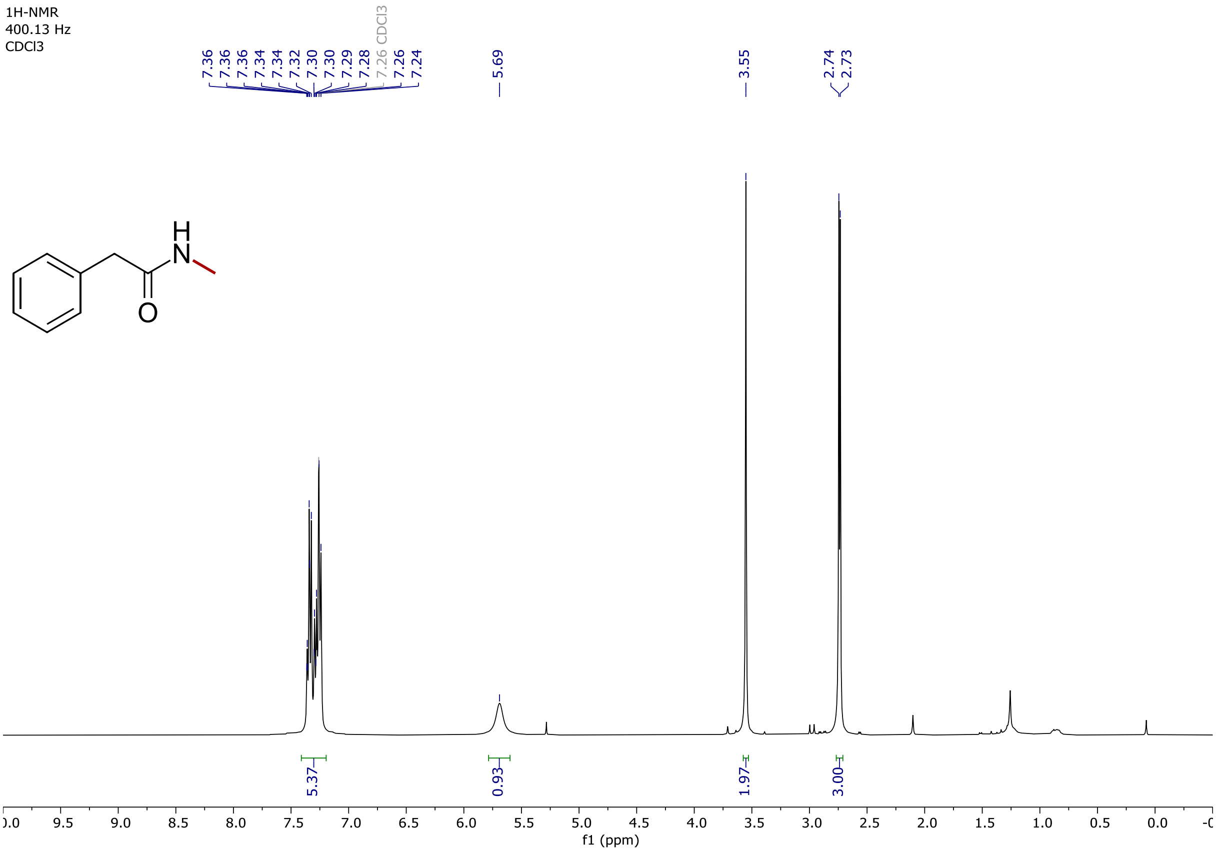

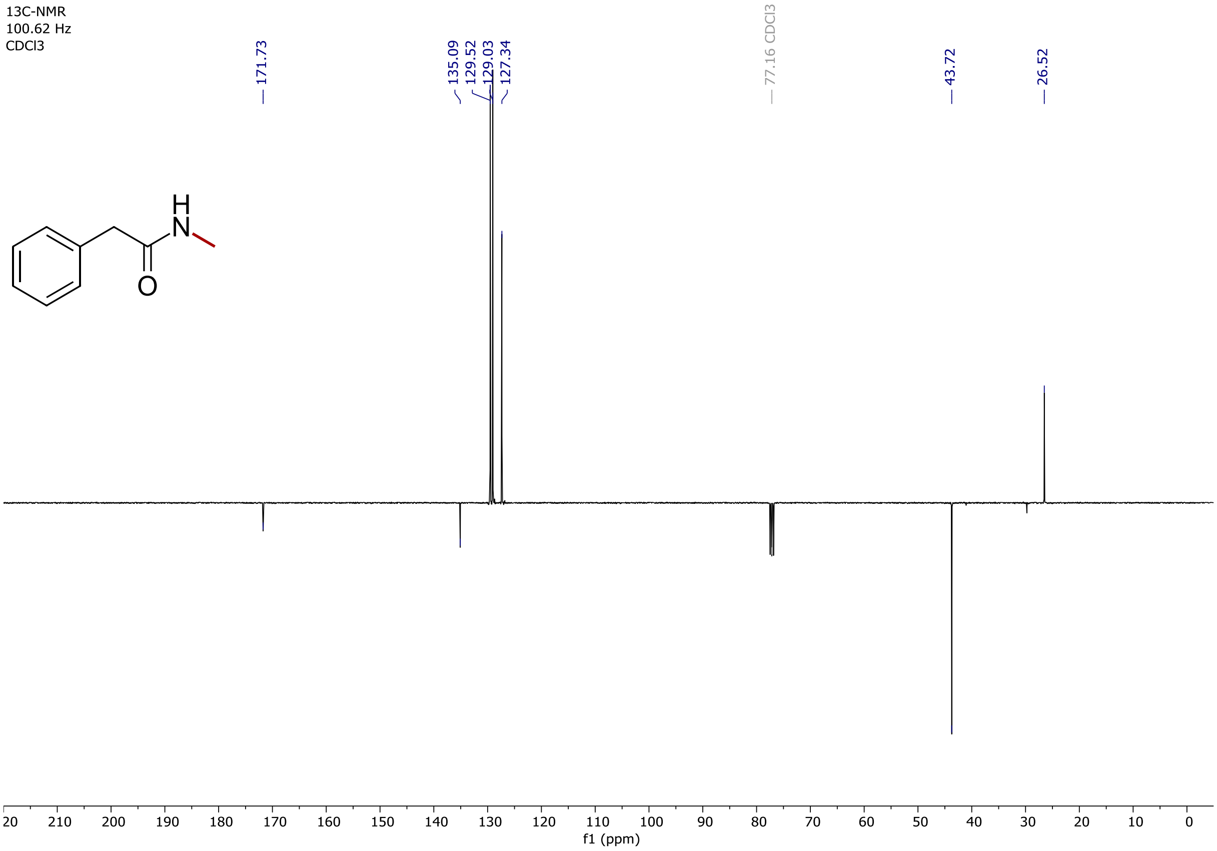

4-Methoxy-*N*-methylbenzamide (2j)

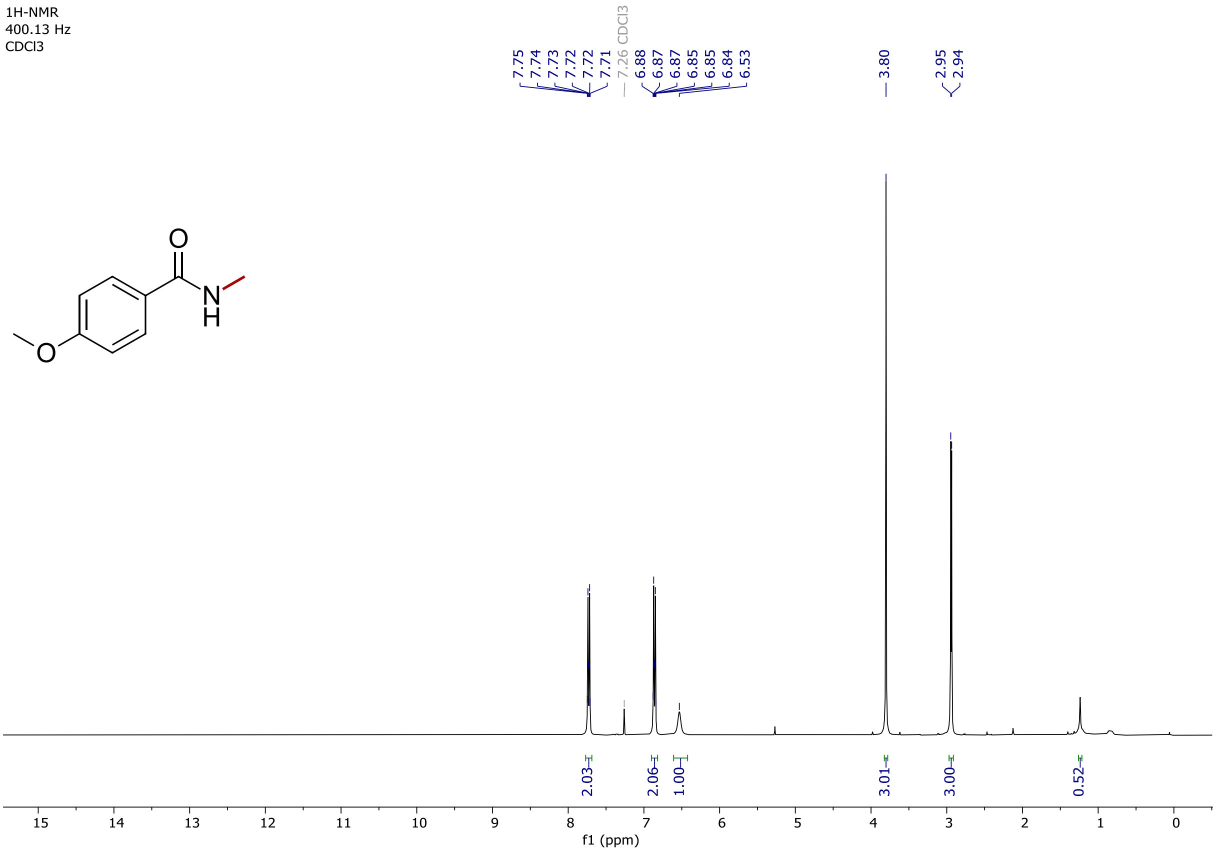

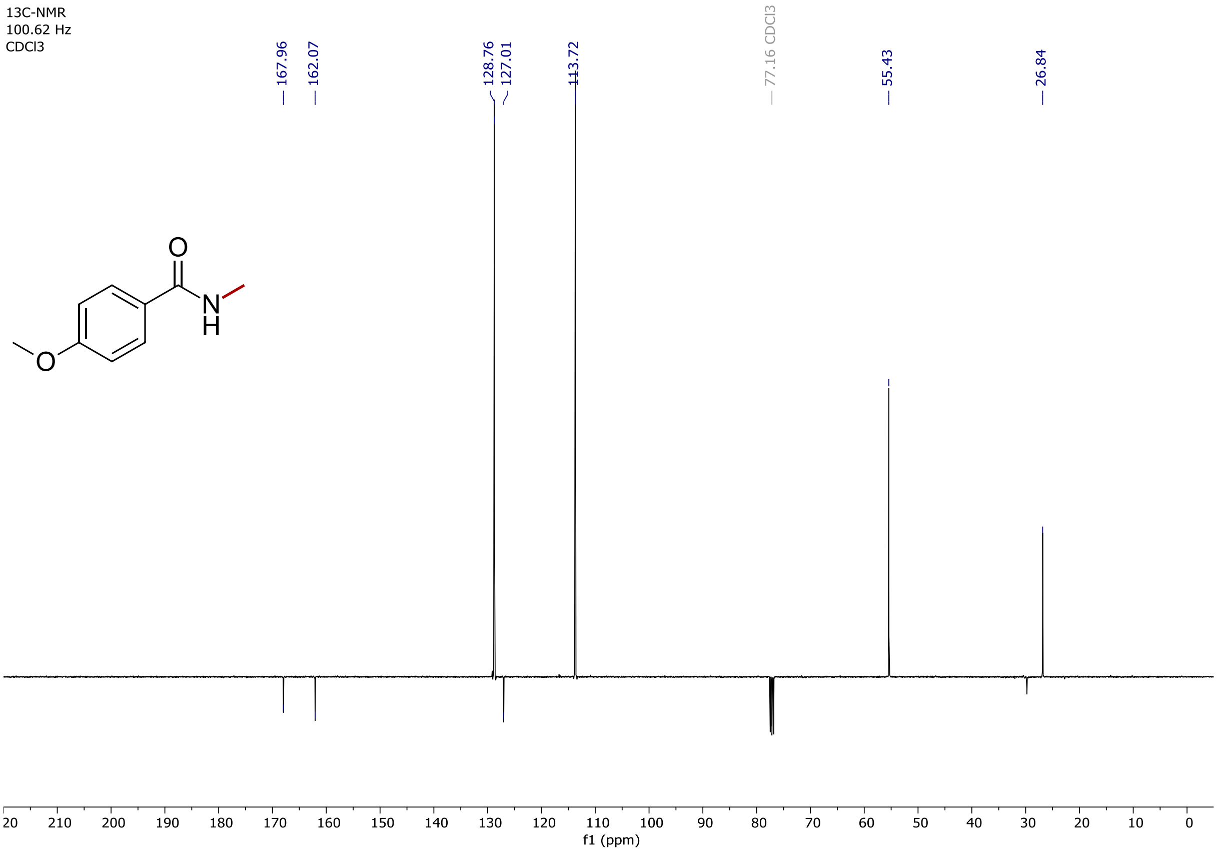

3,5-Dimethoxy-*N*-methylbenzamide (2k)

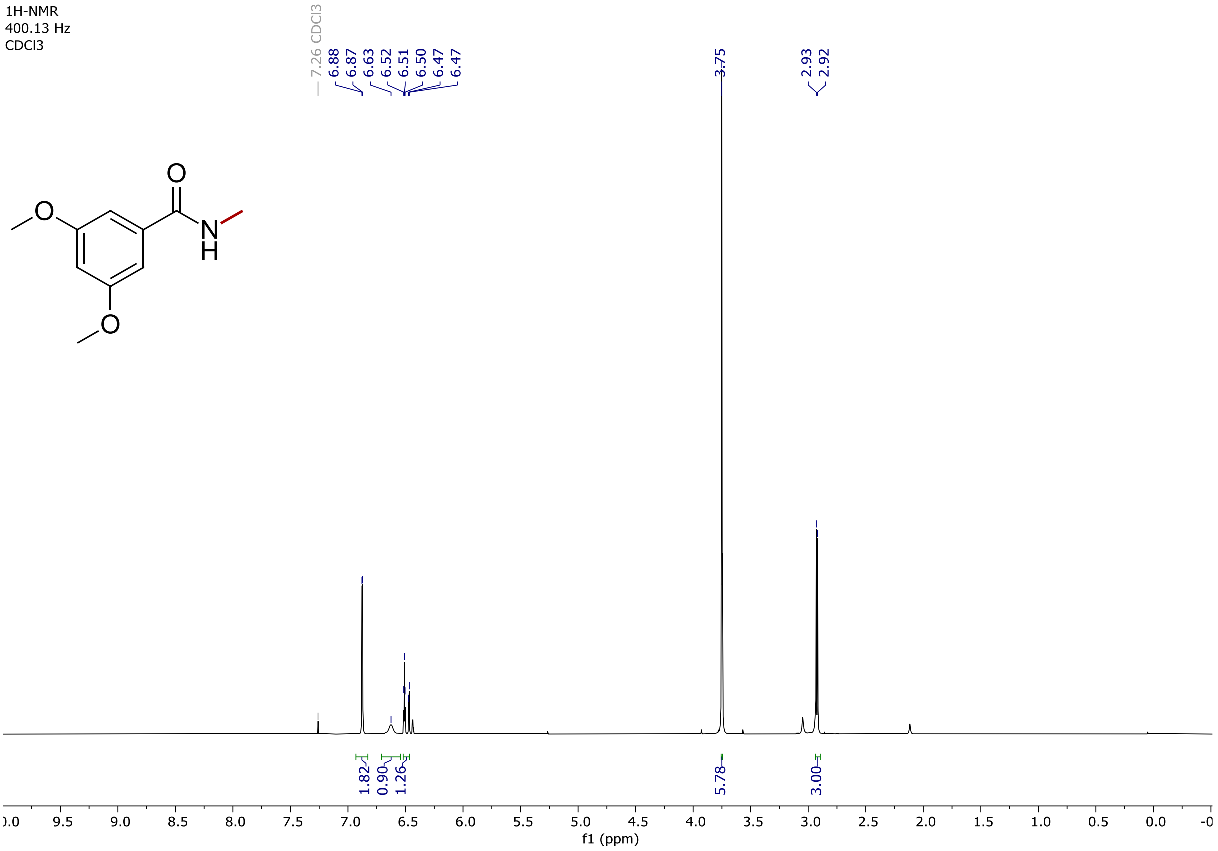

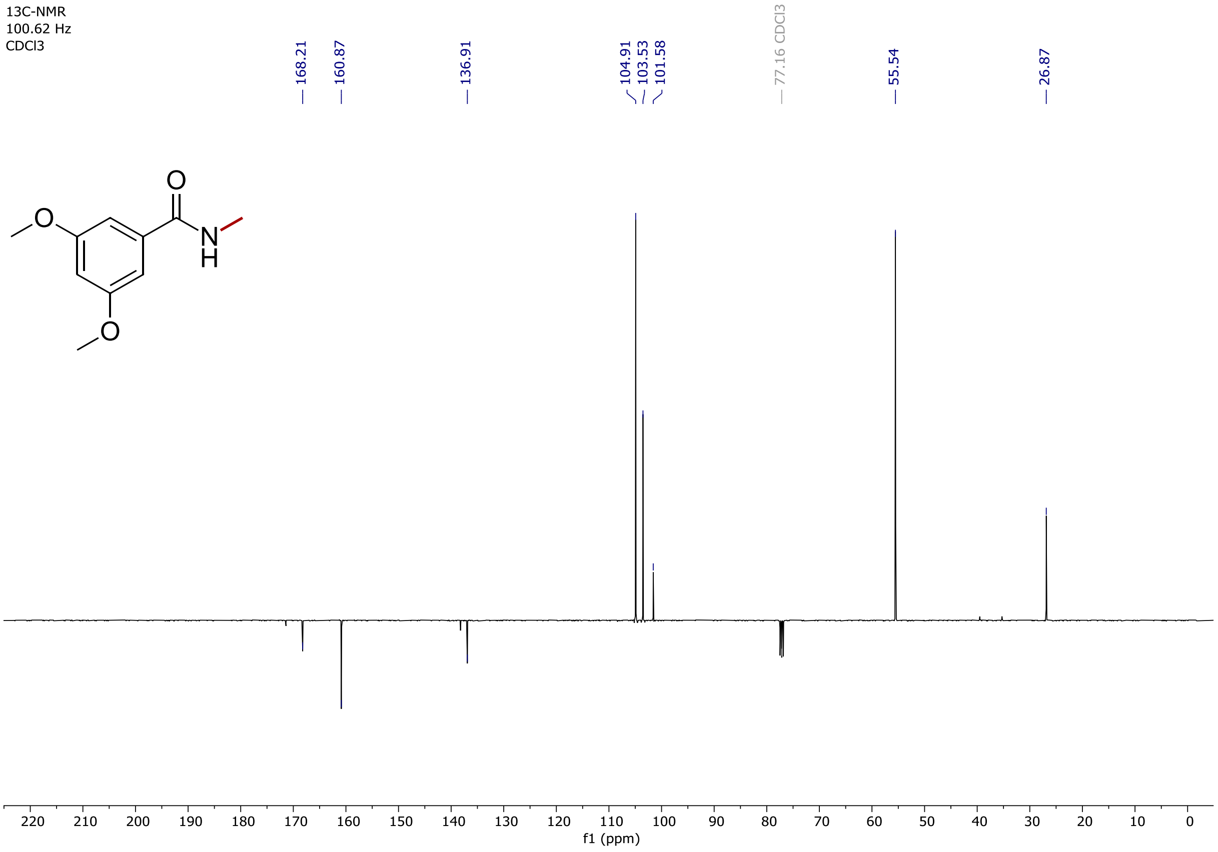

N-Methyl-4-nitrobenzamide (2l)

1H-NMR
400.13 Hz
DMSO

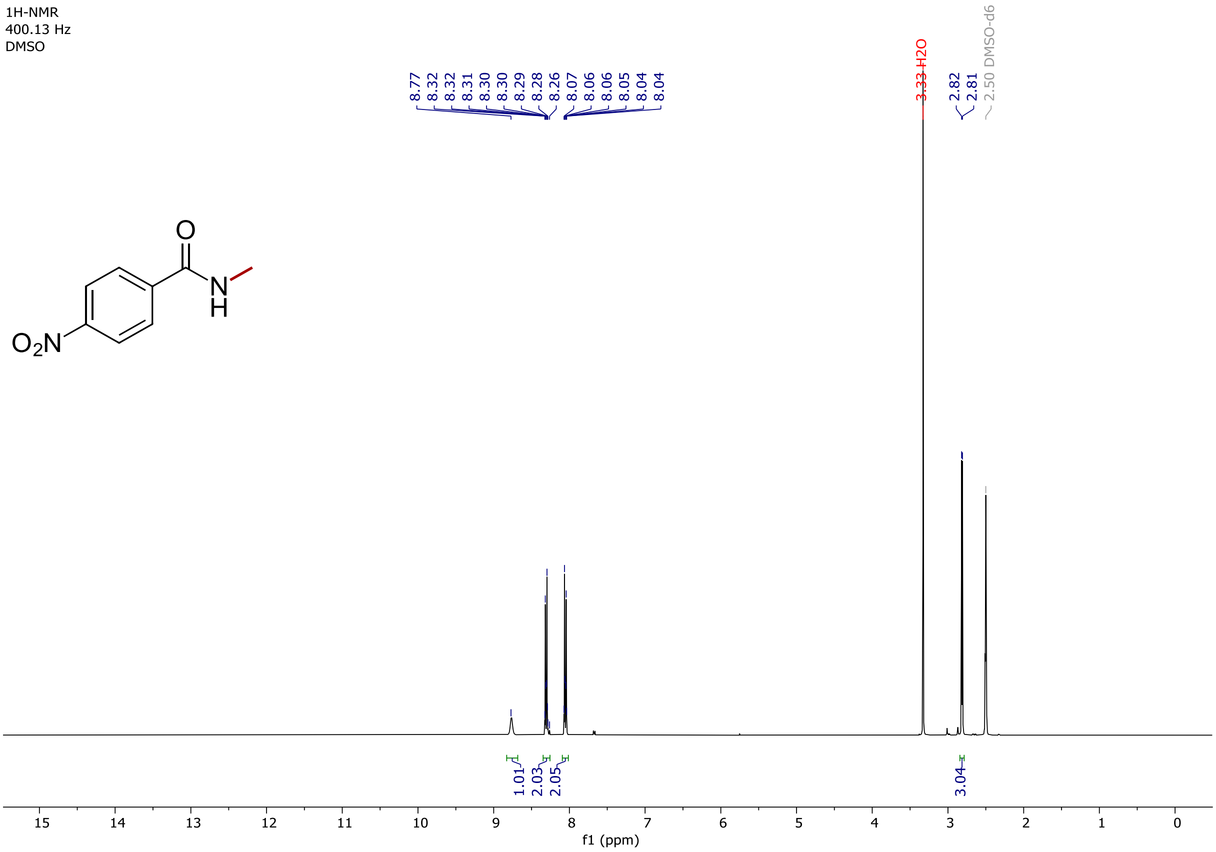

13C-NMR
100.62 Hz
DMSO

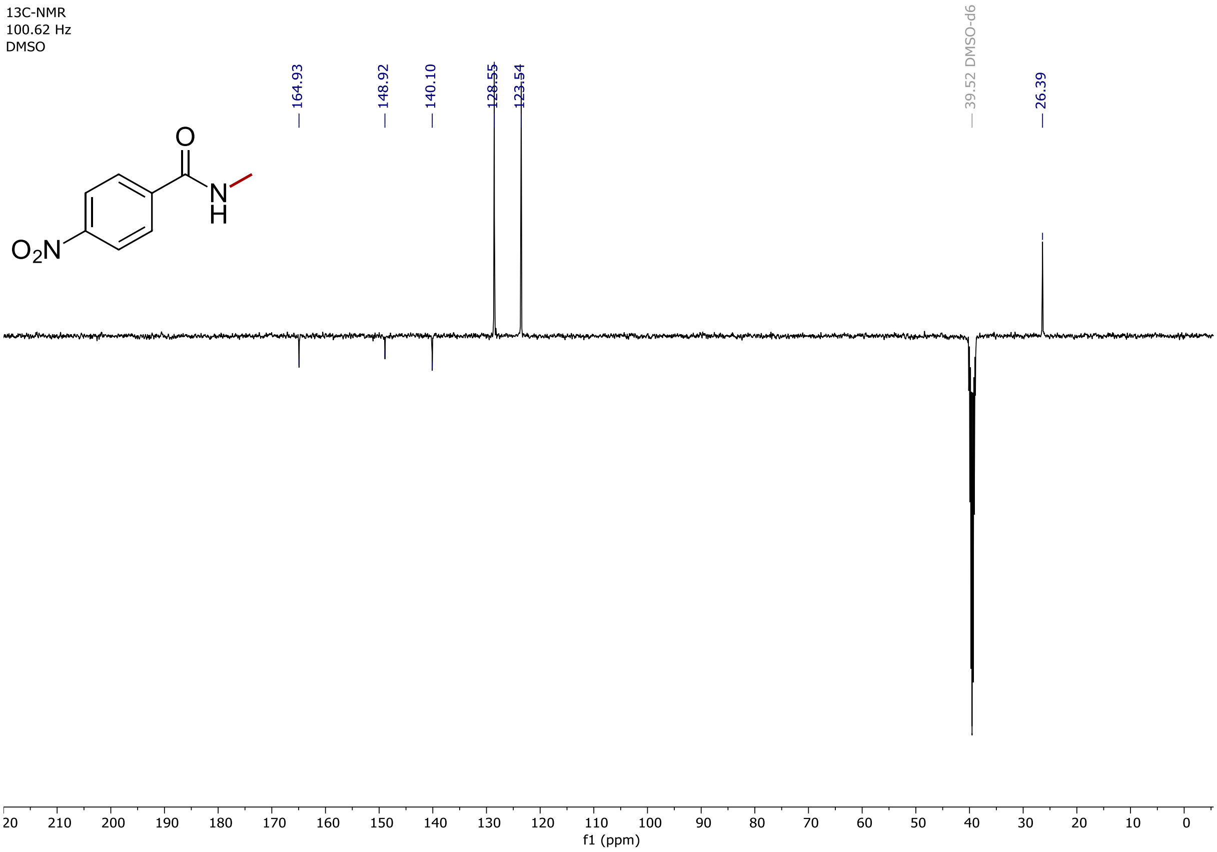

N-Methyl-α-naphthylacetamide (2m)

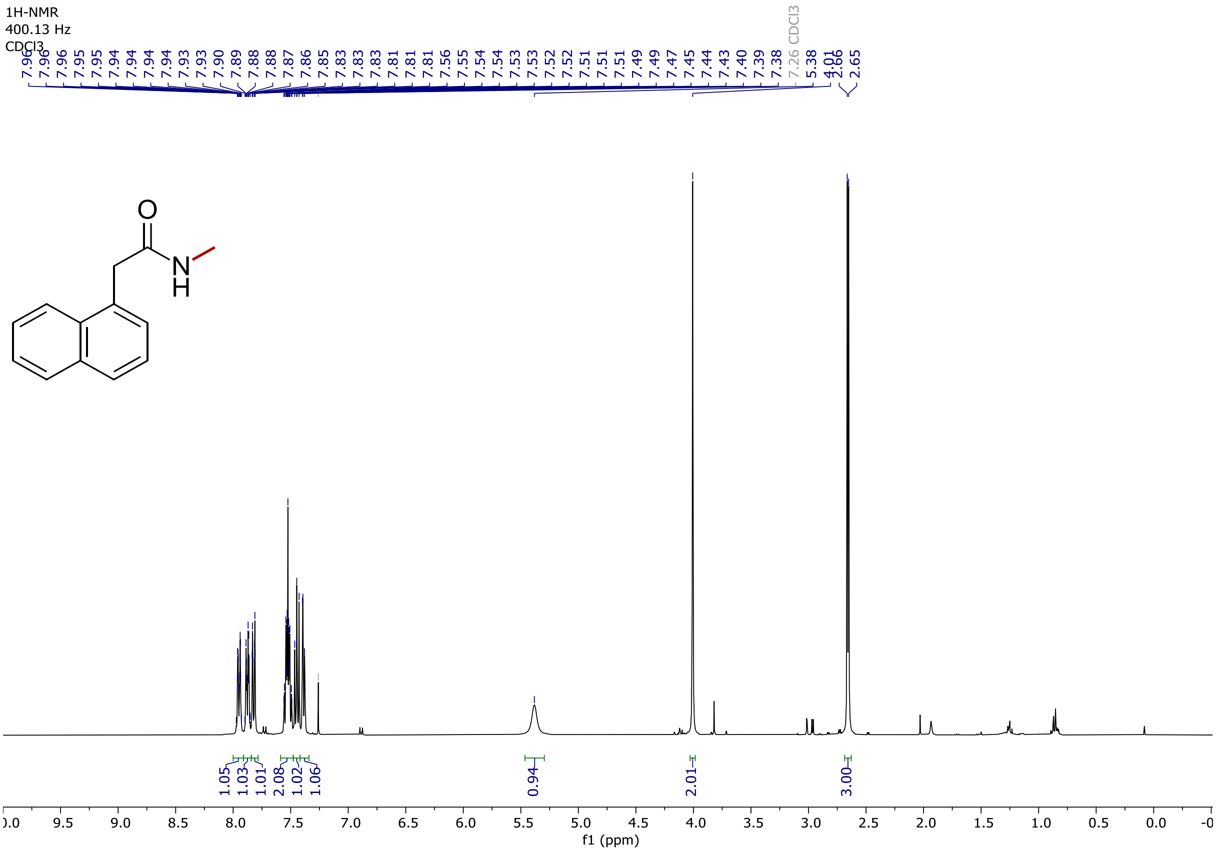

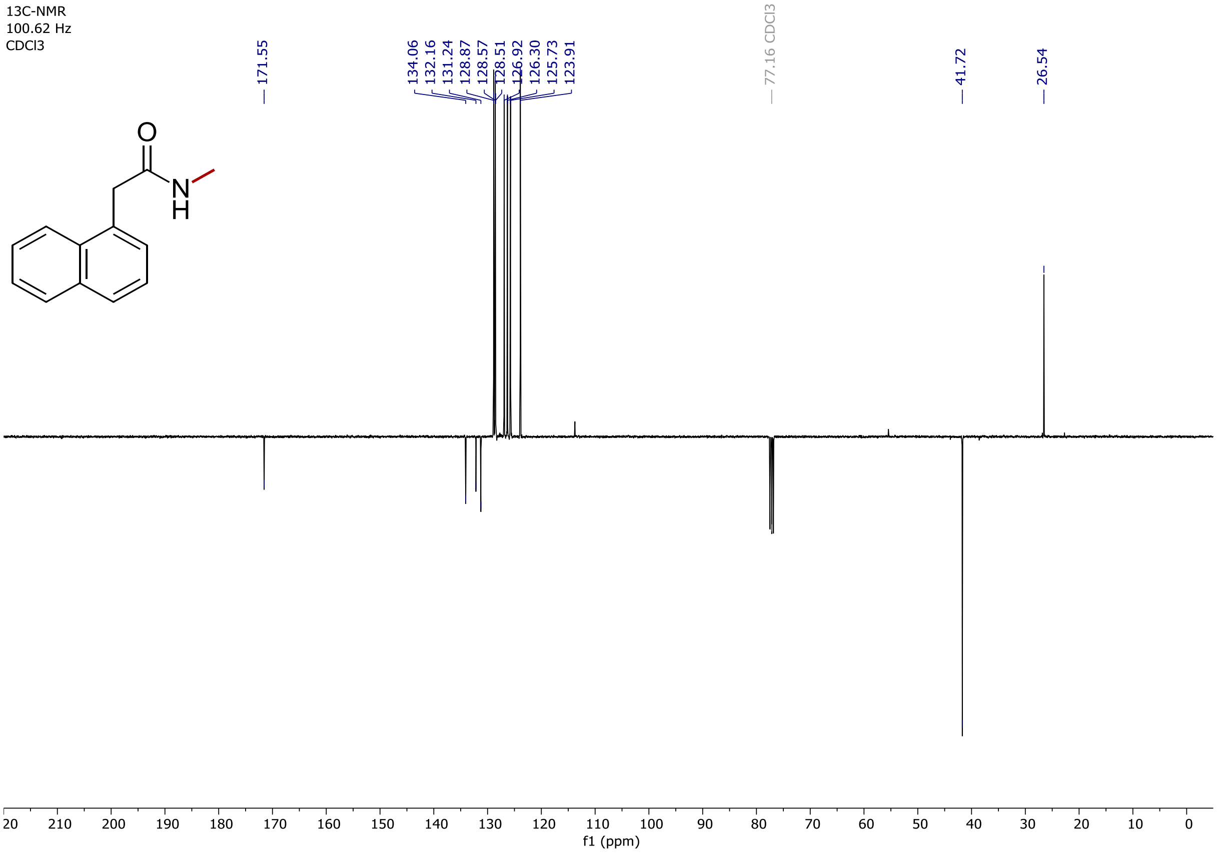

N-Methylhexanamide (2n)

1H-NMR
400.13 Hz
CDCl3

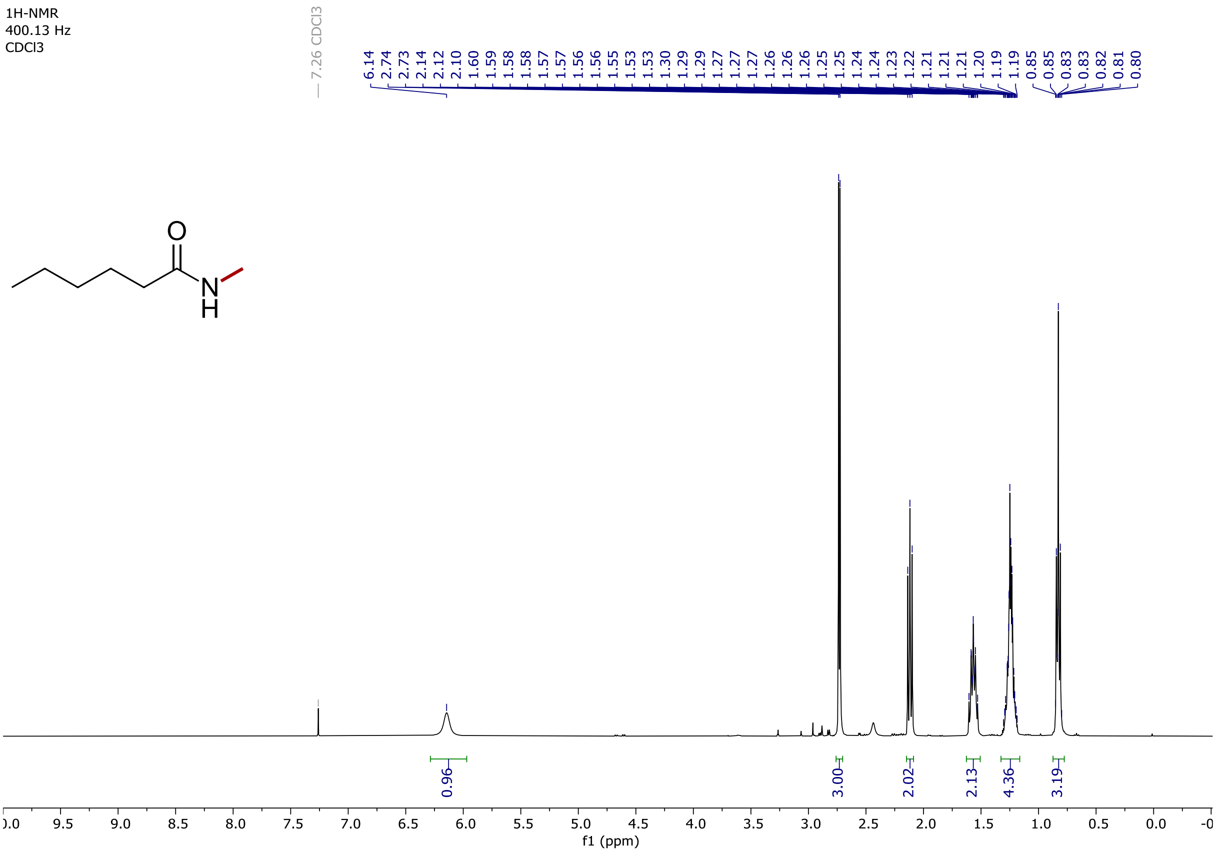

13C-NMR
100.62 Hz
CDCl3

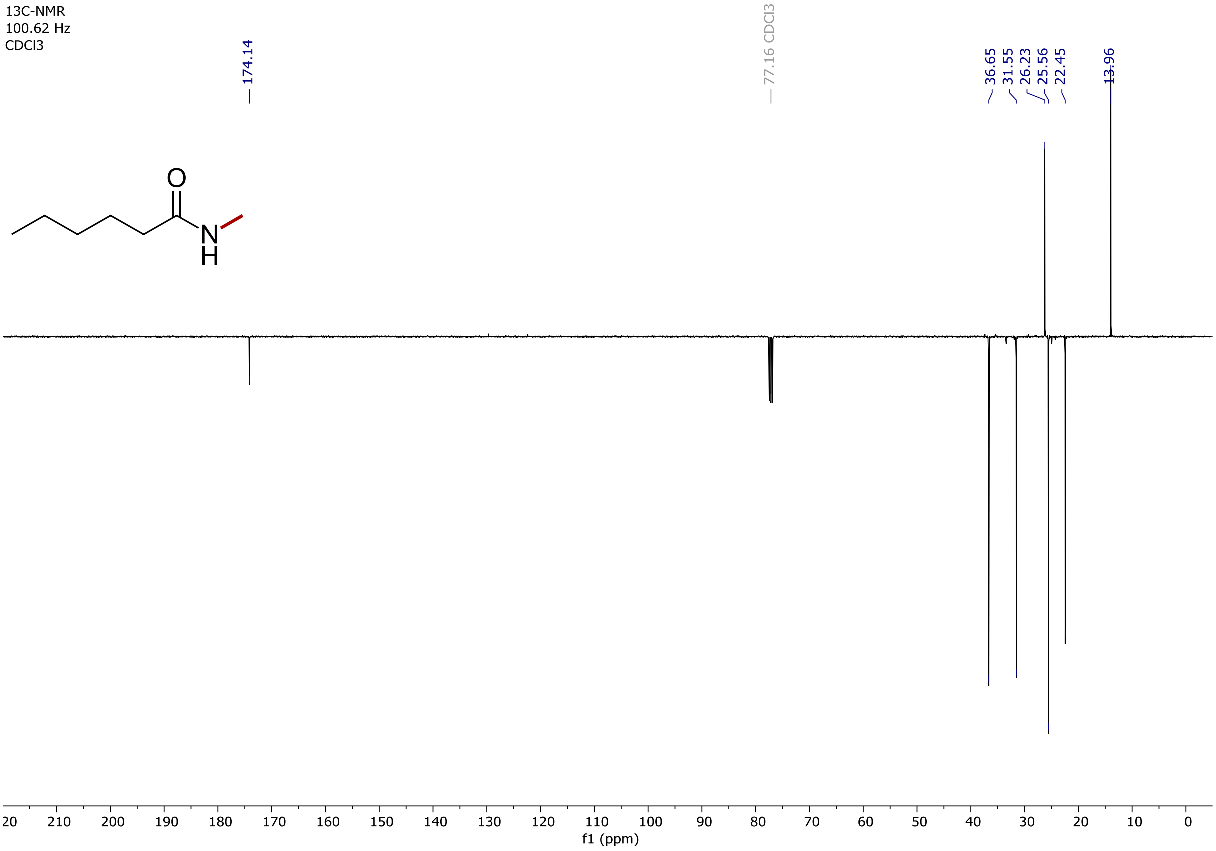

1-Methylindole (5a)

1H-NMR
400.13 Hz
CDCl3

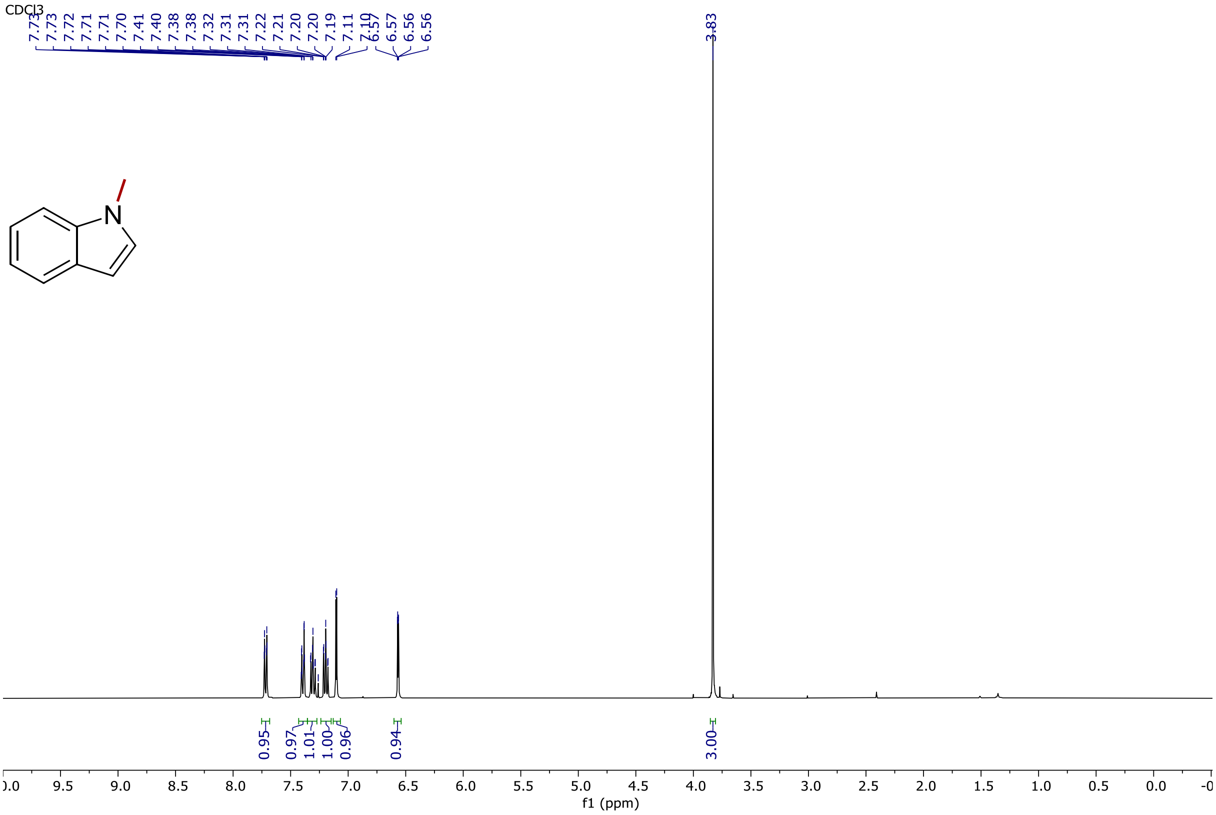

13C-NMR
100.62 Hz
CDCl3

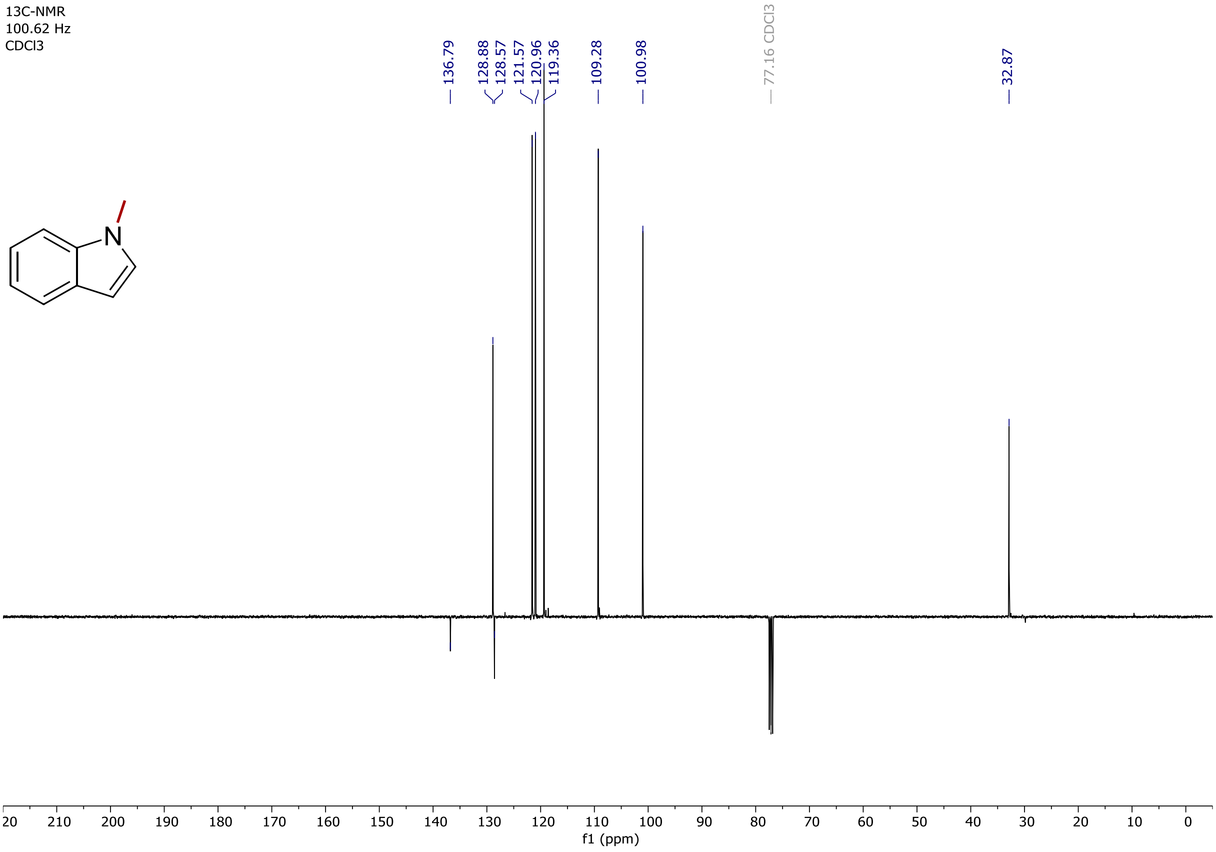

5-Methoxy-1-methylindole (5b)

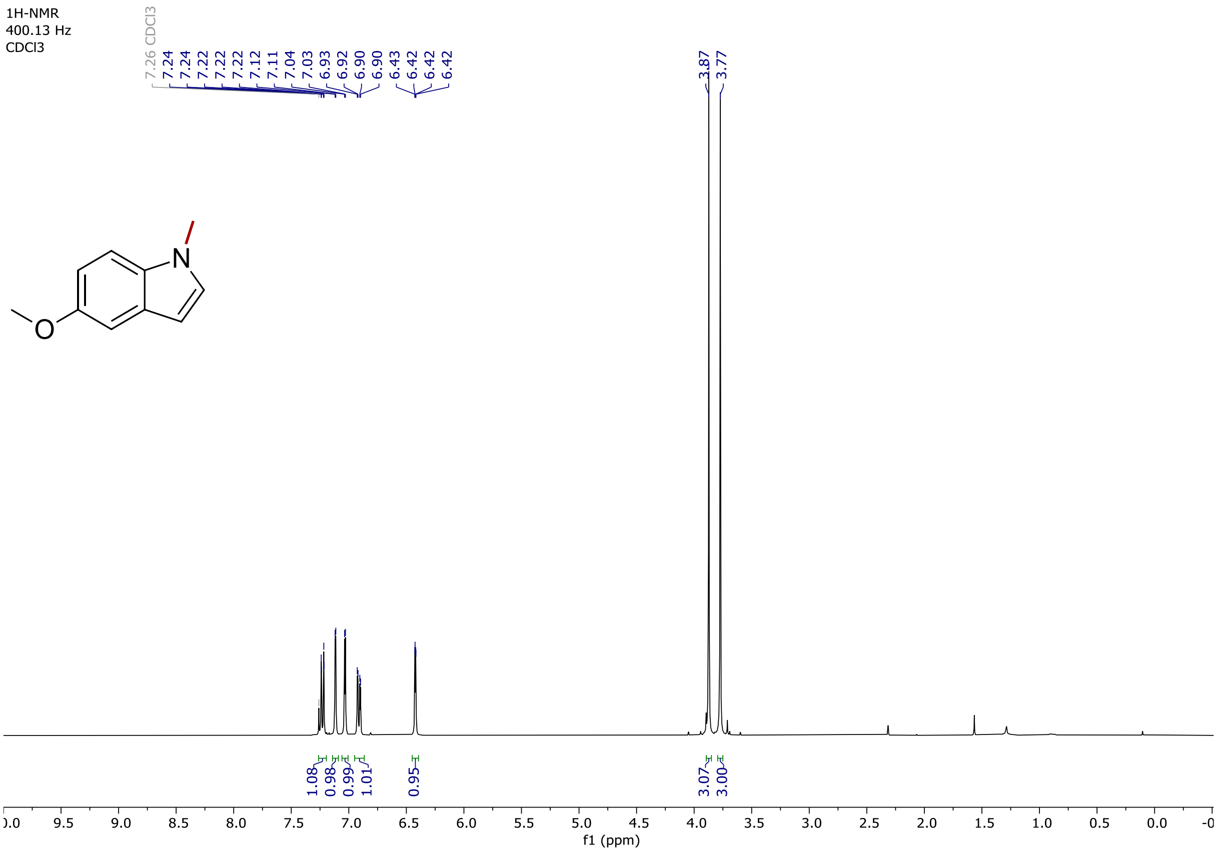

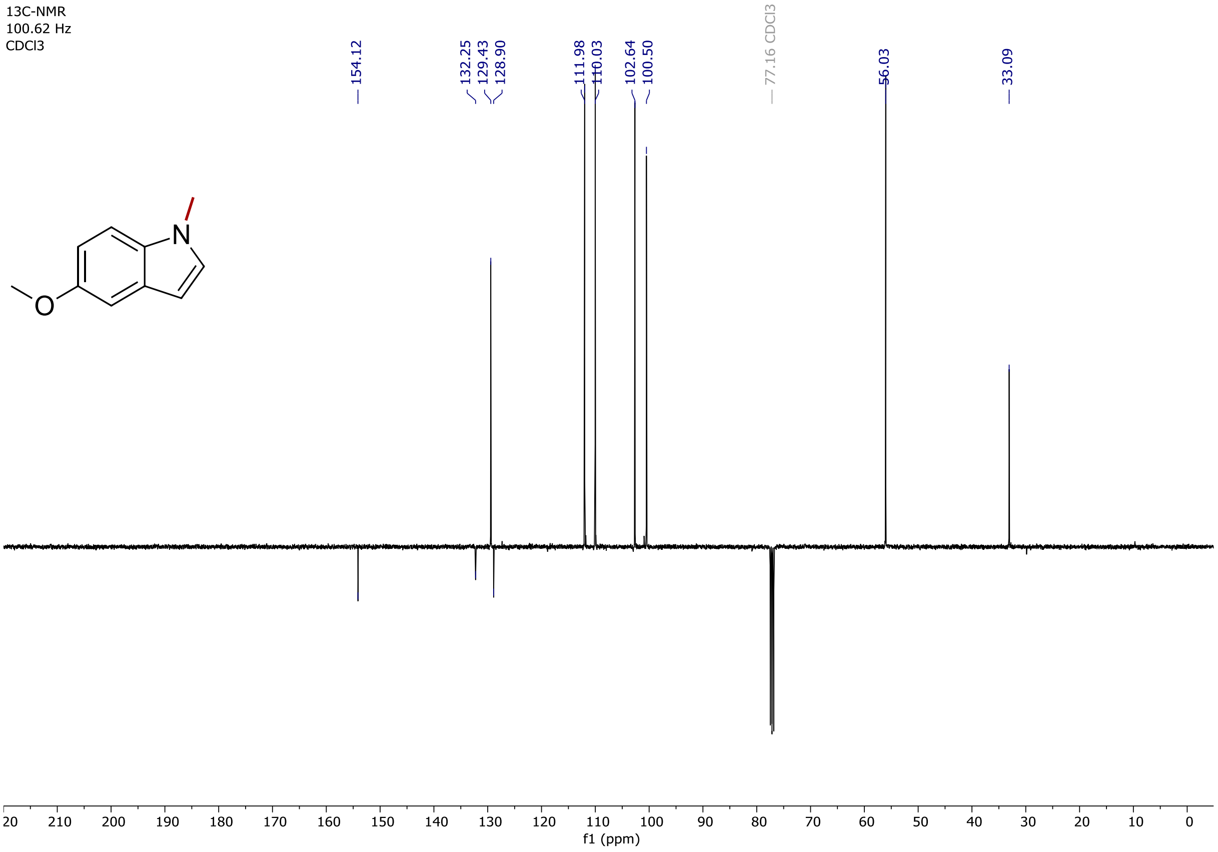

6-Fluoro-1-methylindole (5c)

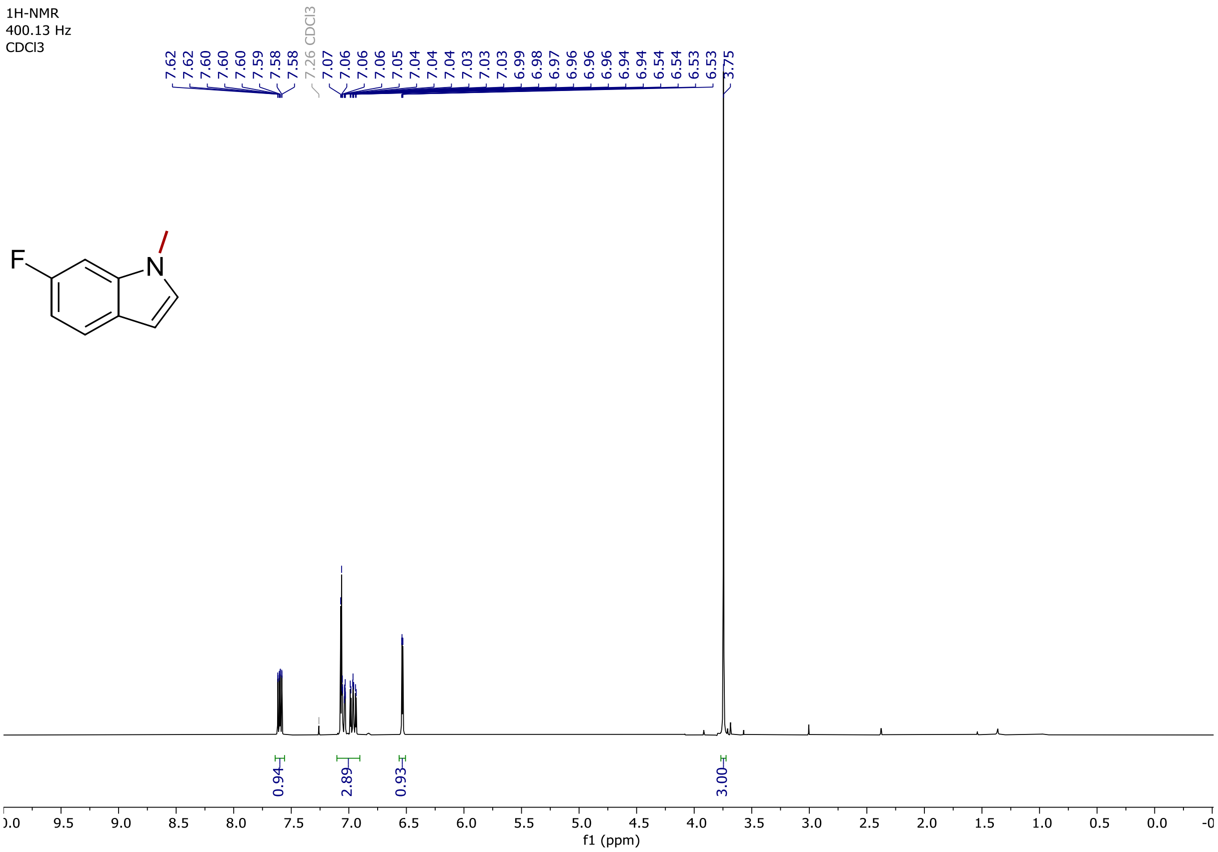

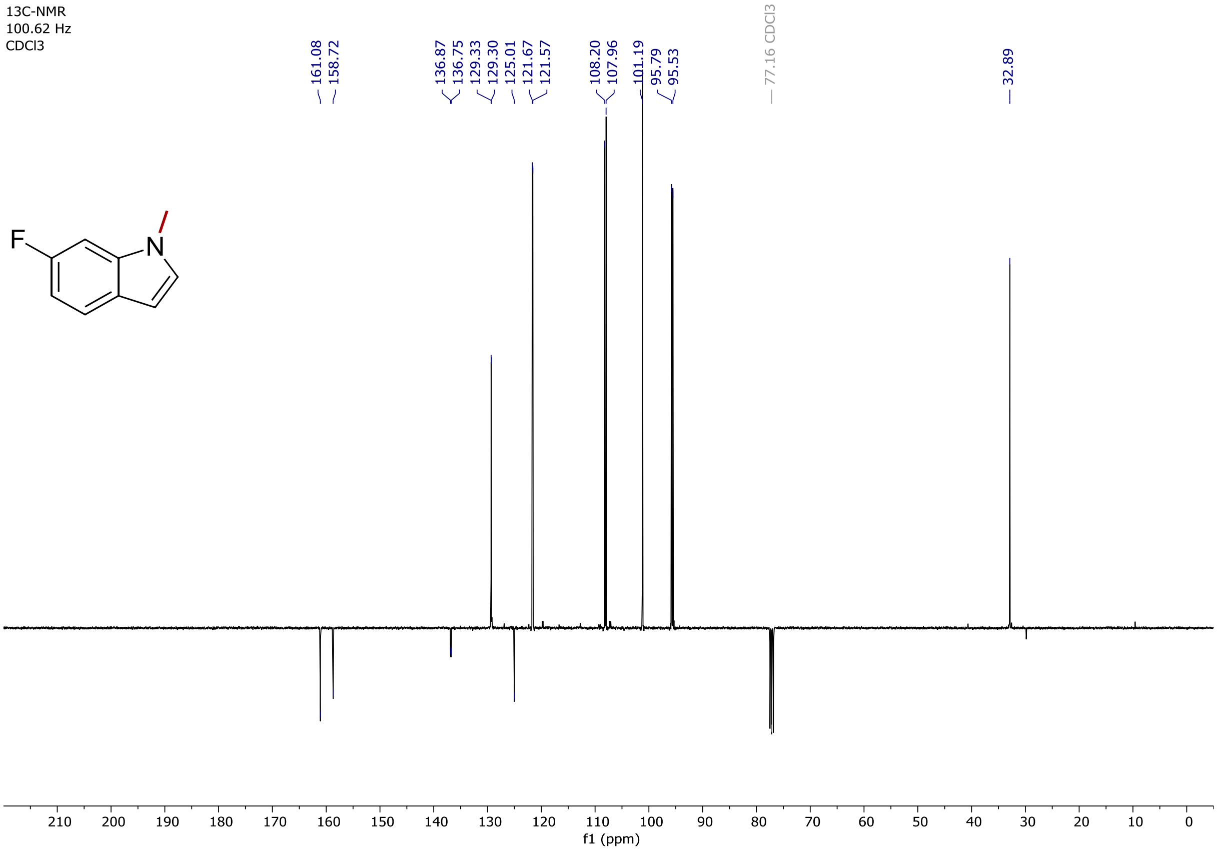

5-Chloro-1-methylindole (5d)

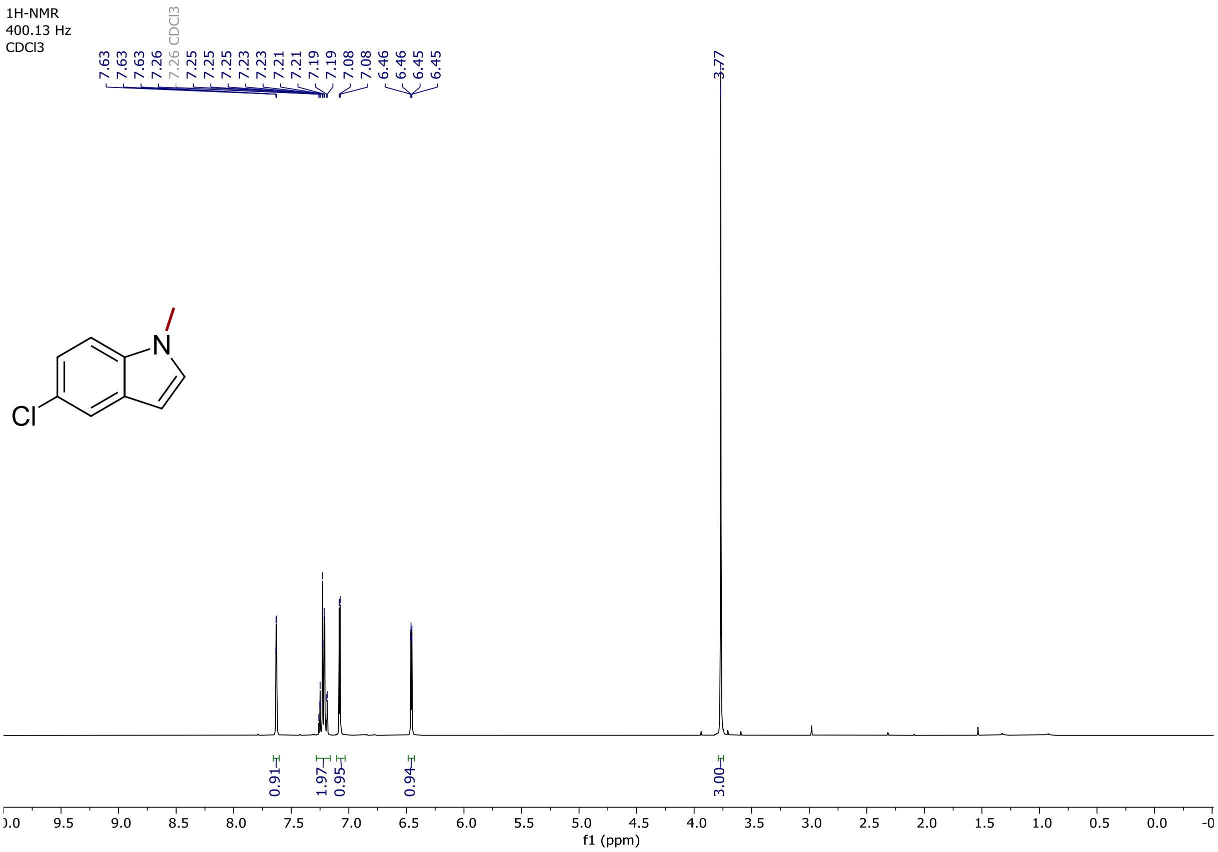

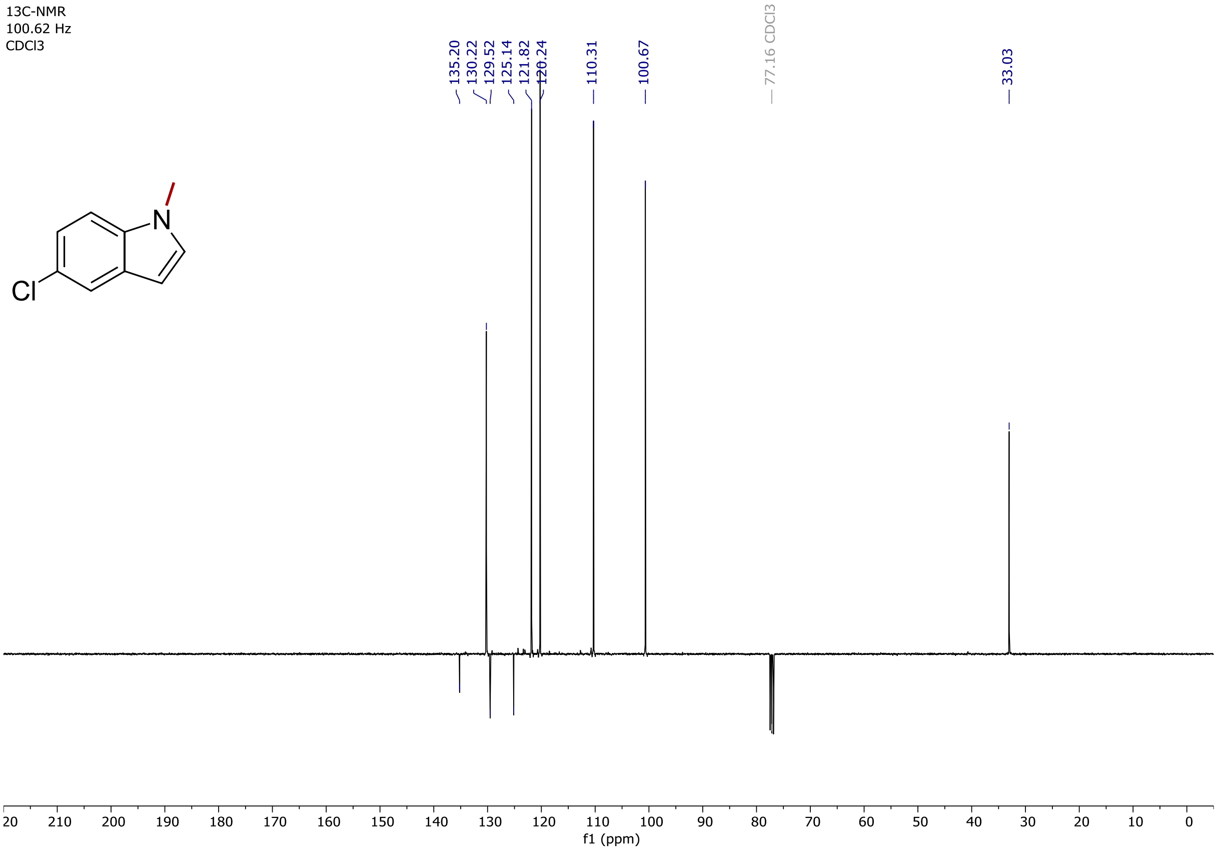

5-Bromo-1-methylindole (5e)

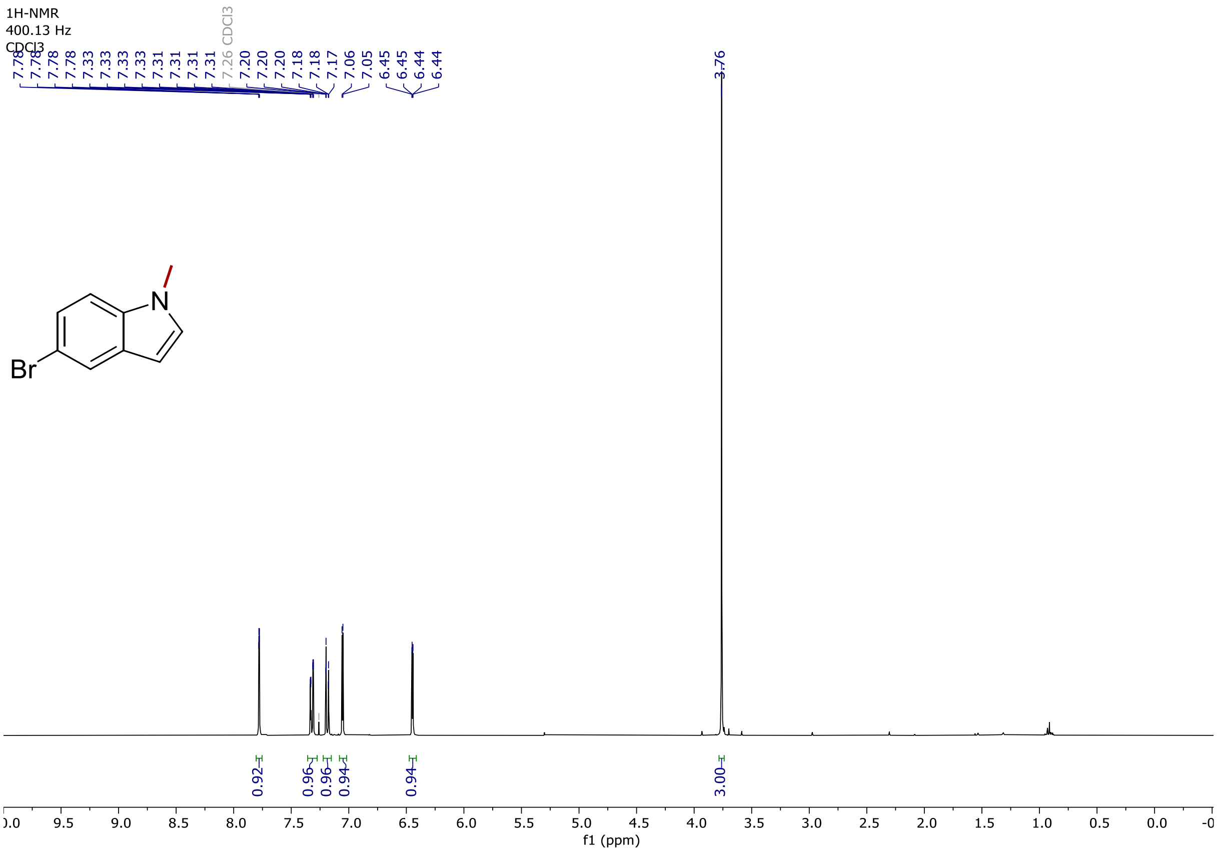

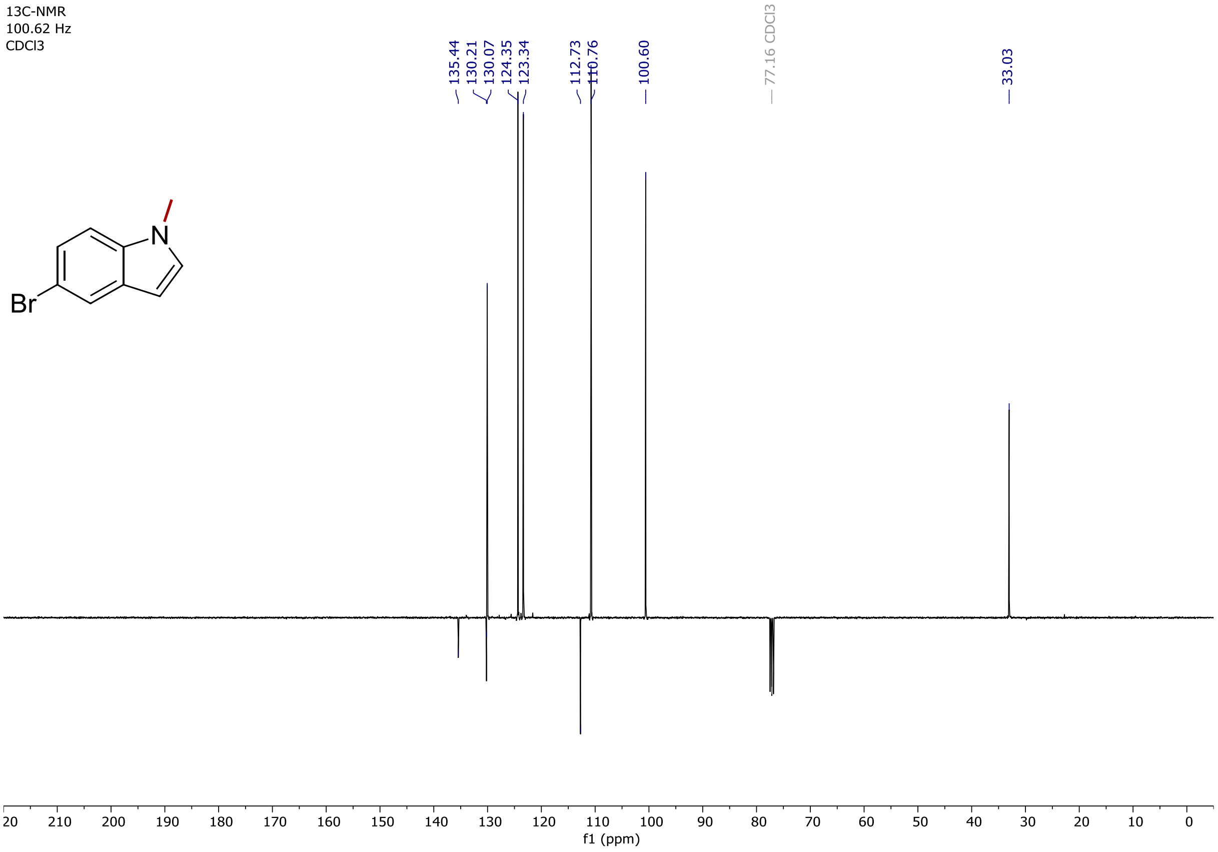

5-Iodo-1-methylindole (5f)

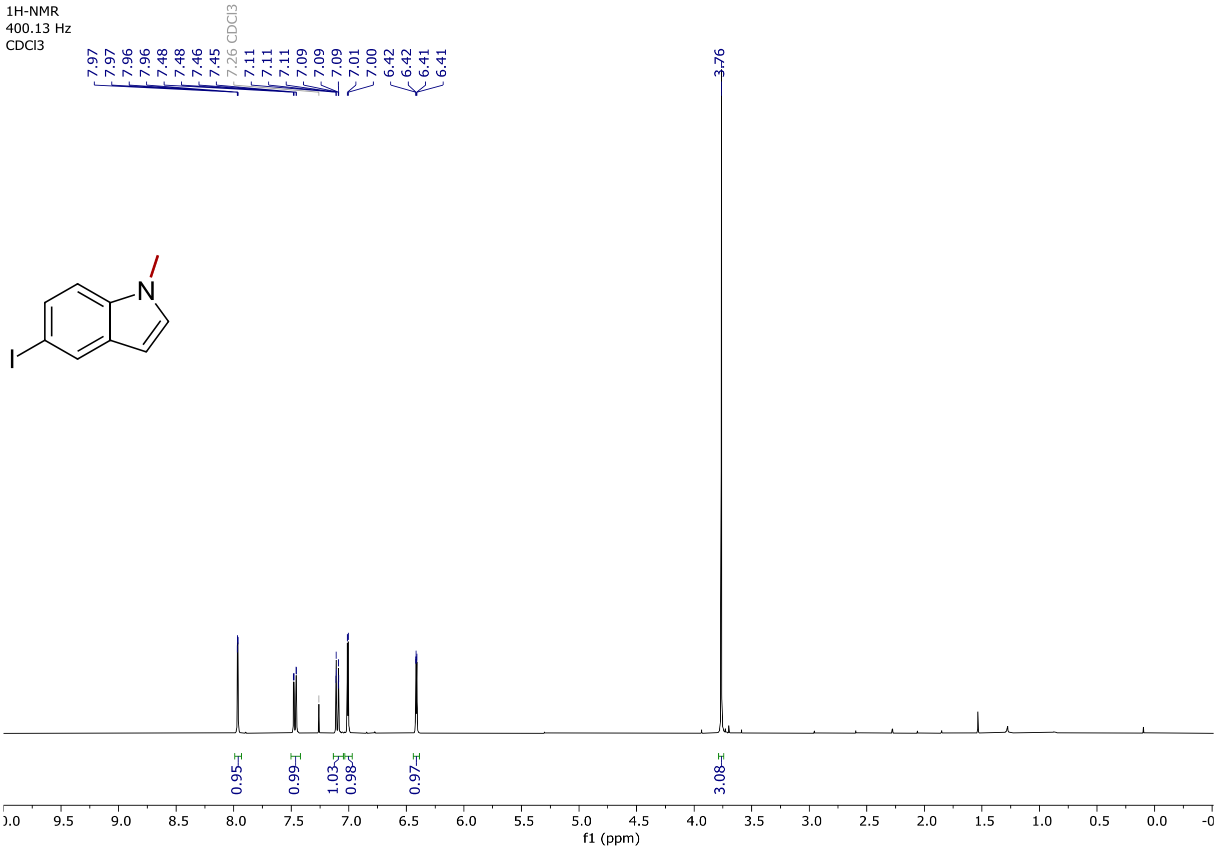

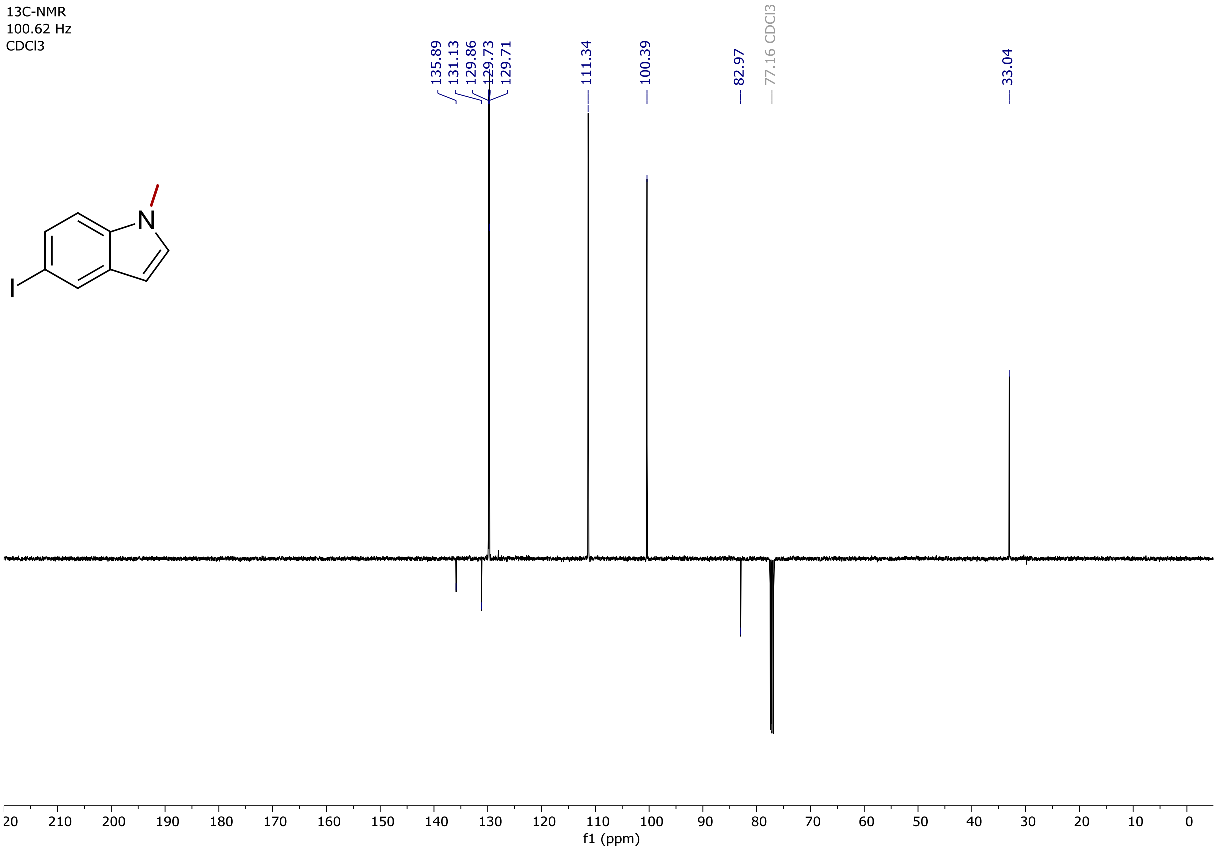

1-Methyl-5-nitroindole (5g)

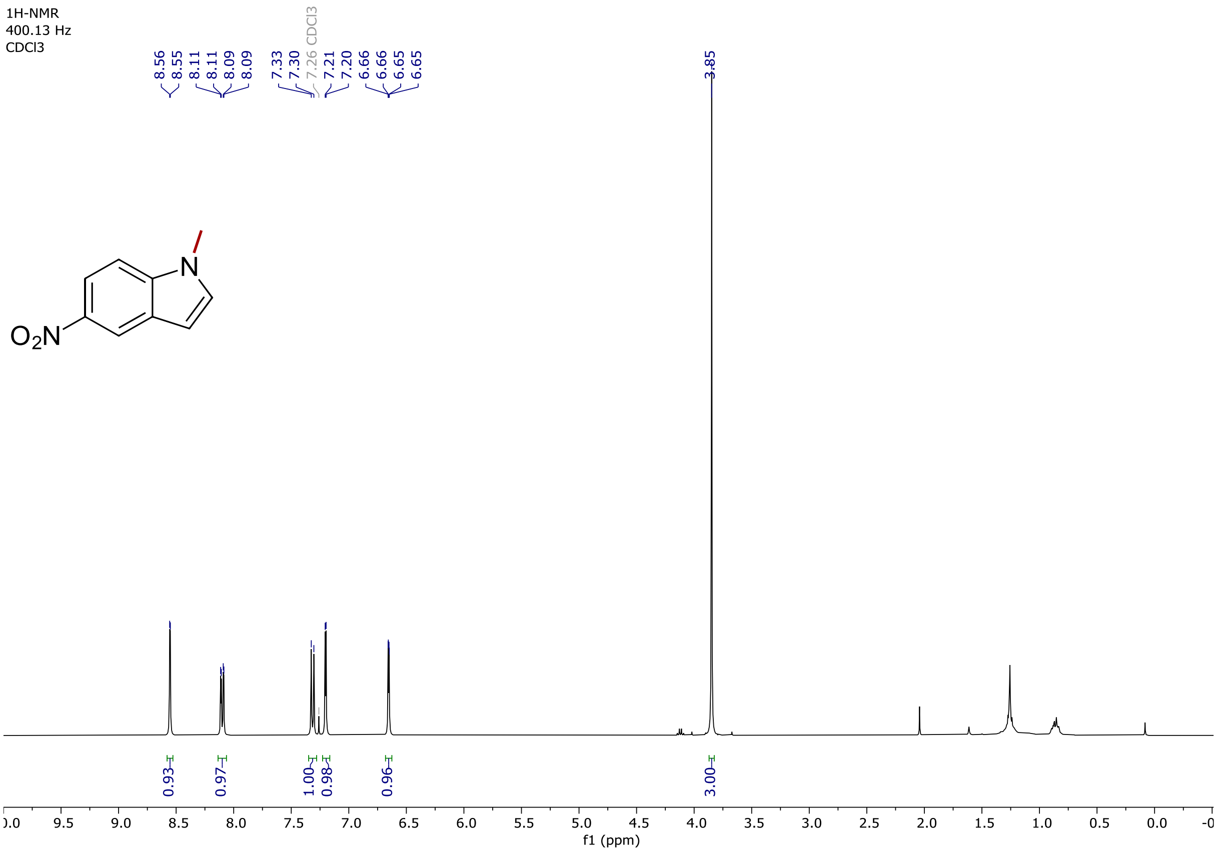

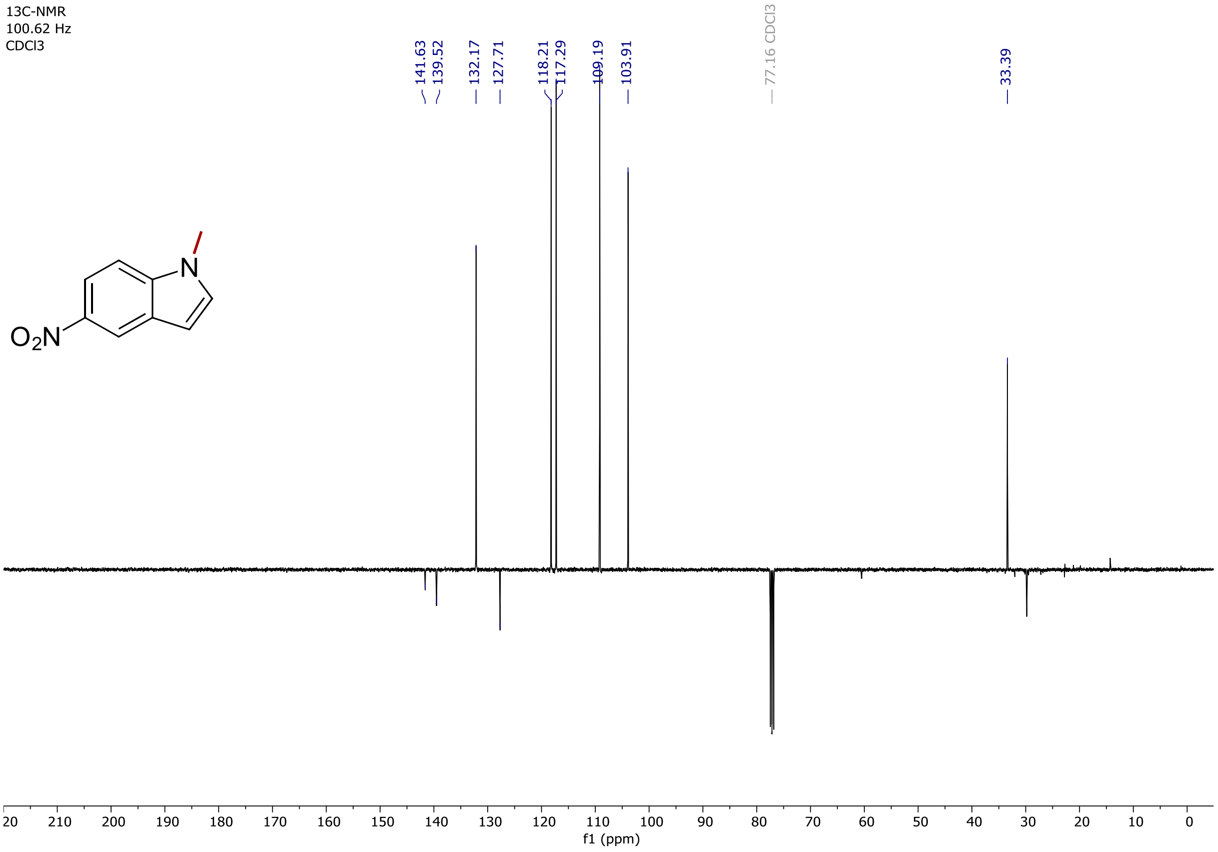

1-Methyl-2-indolecarbaldehyde (5h)

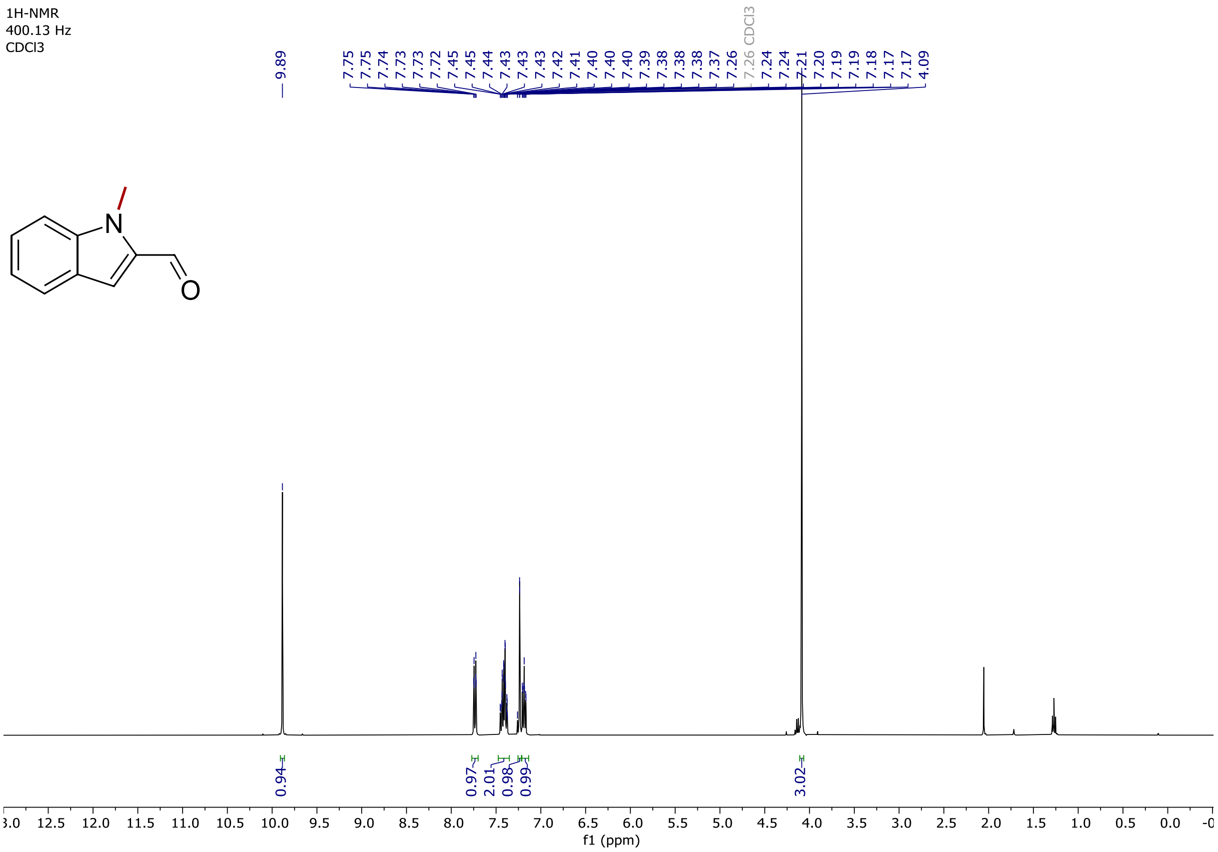

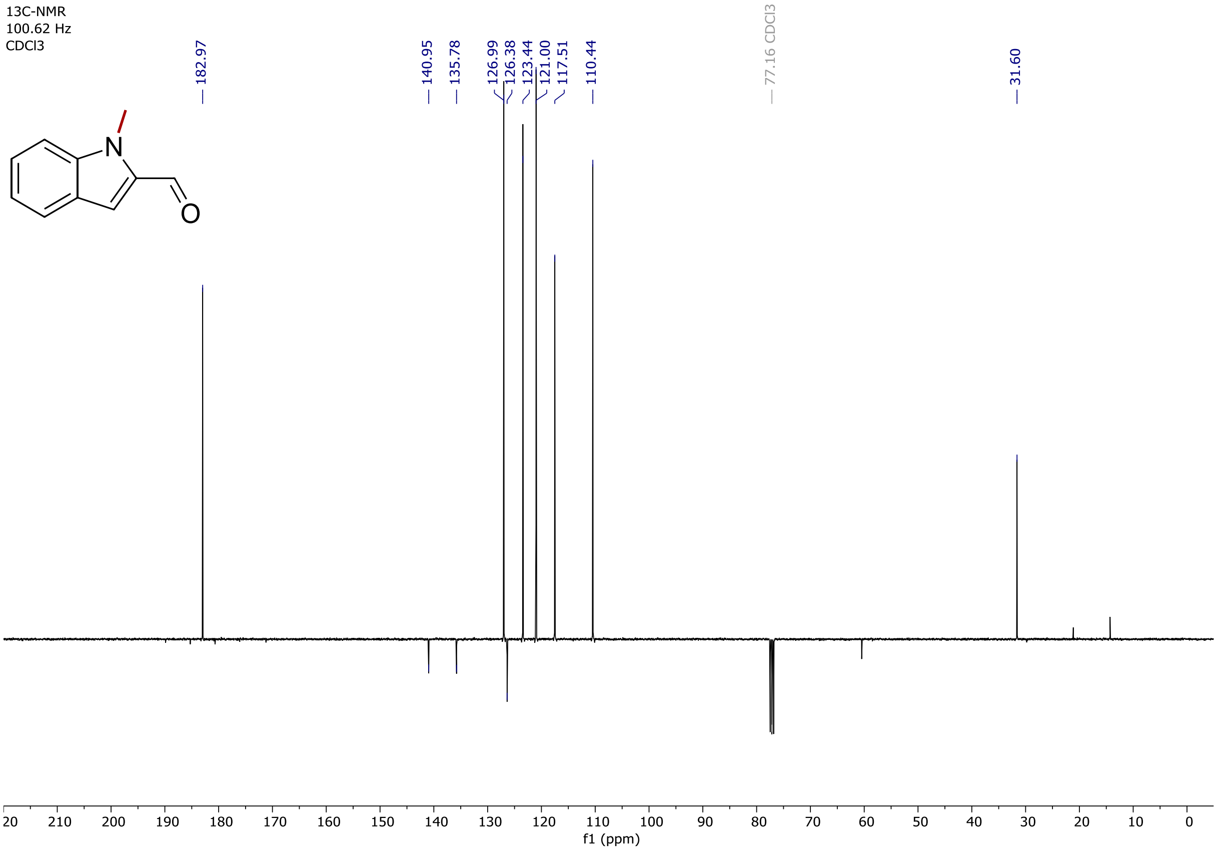

Ethyl 1-methyl-2-indolecarboxylate (5i)

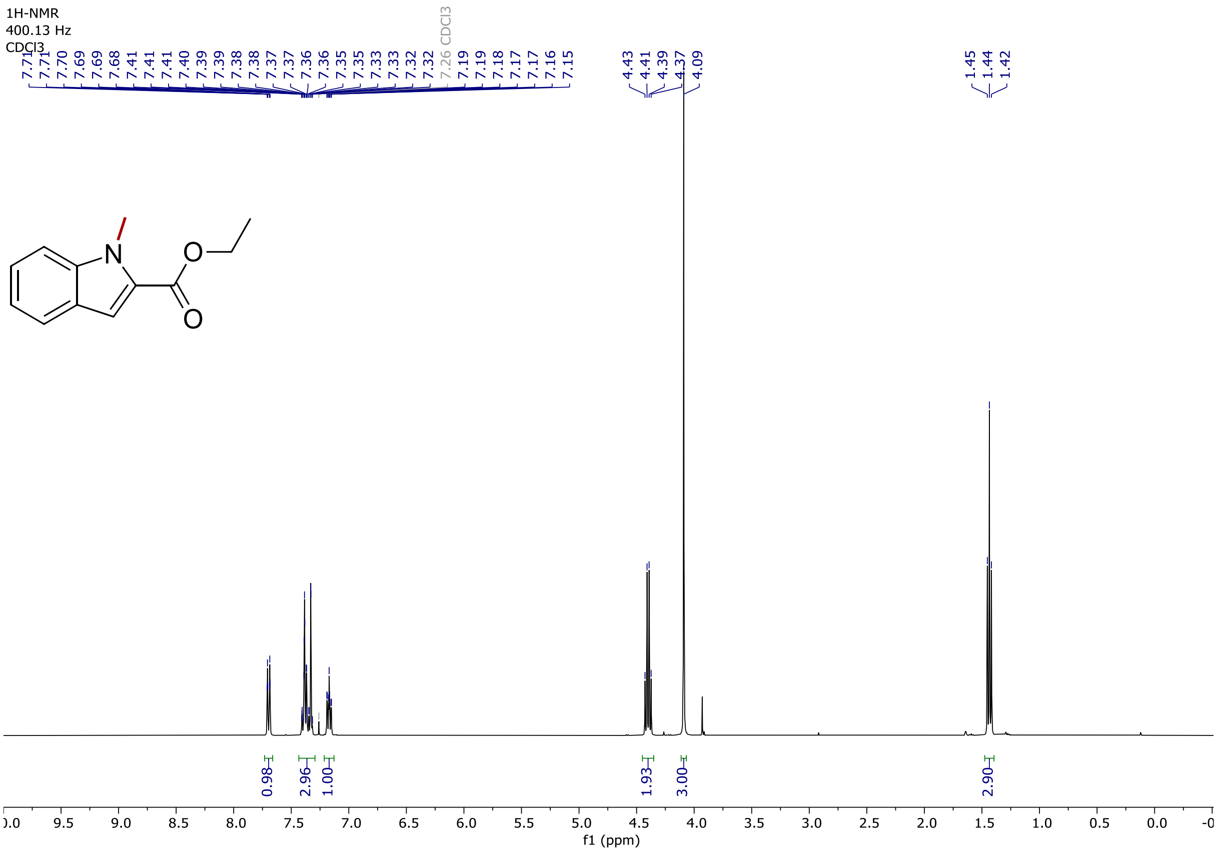

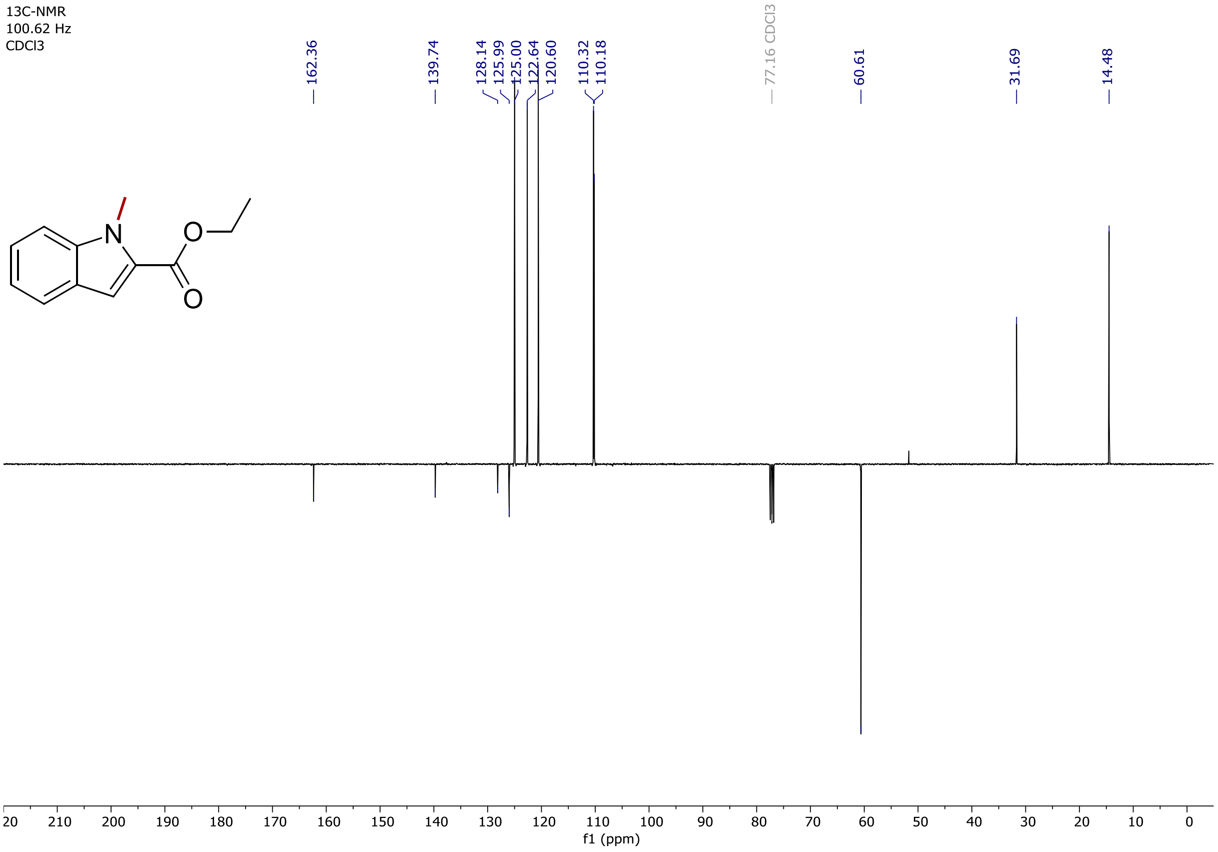

Methyl 1-methyl-5-indolecarboxylate (5j)

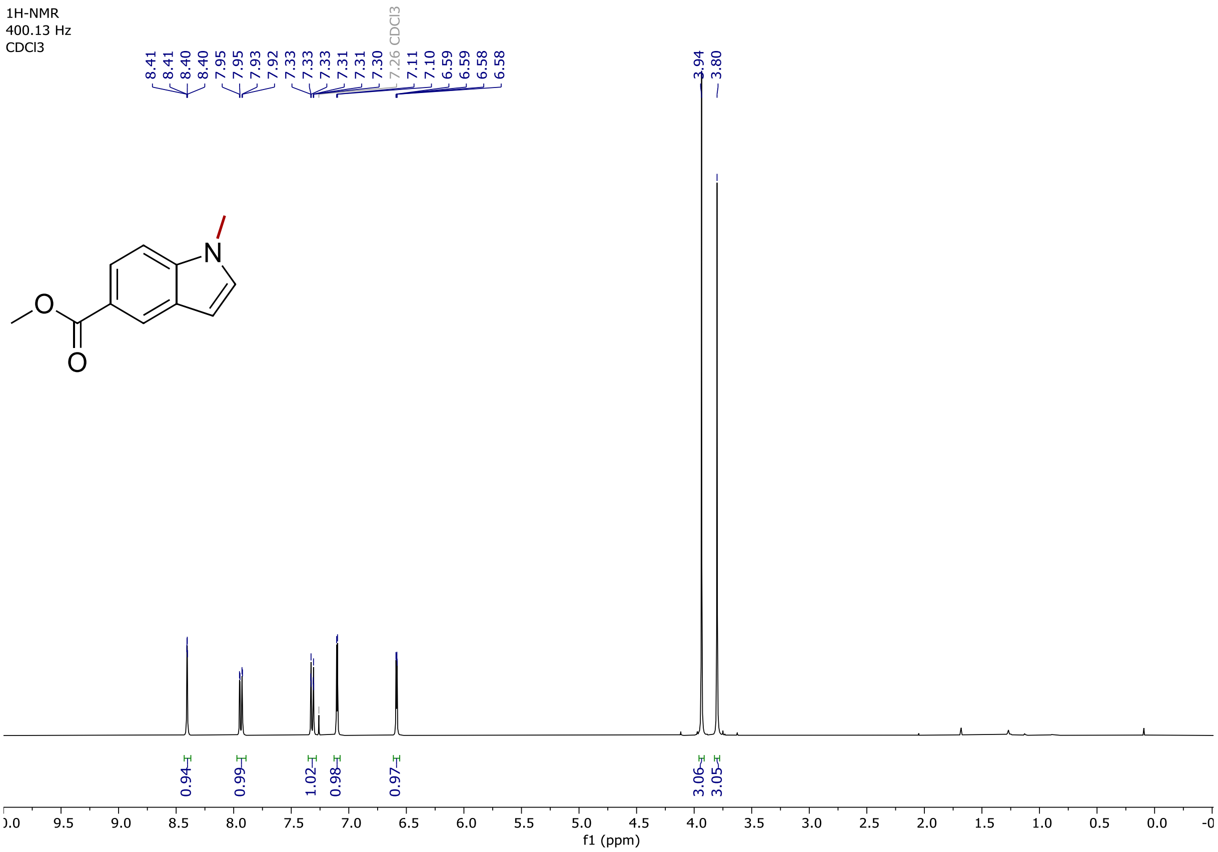

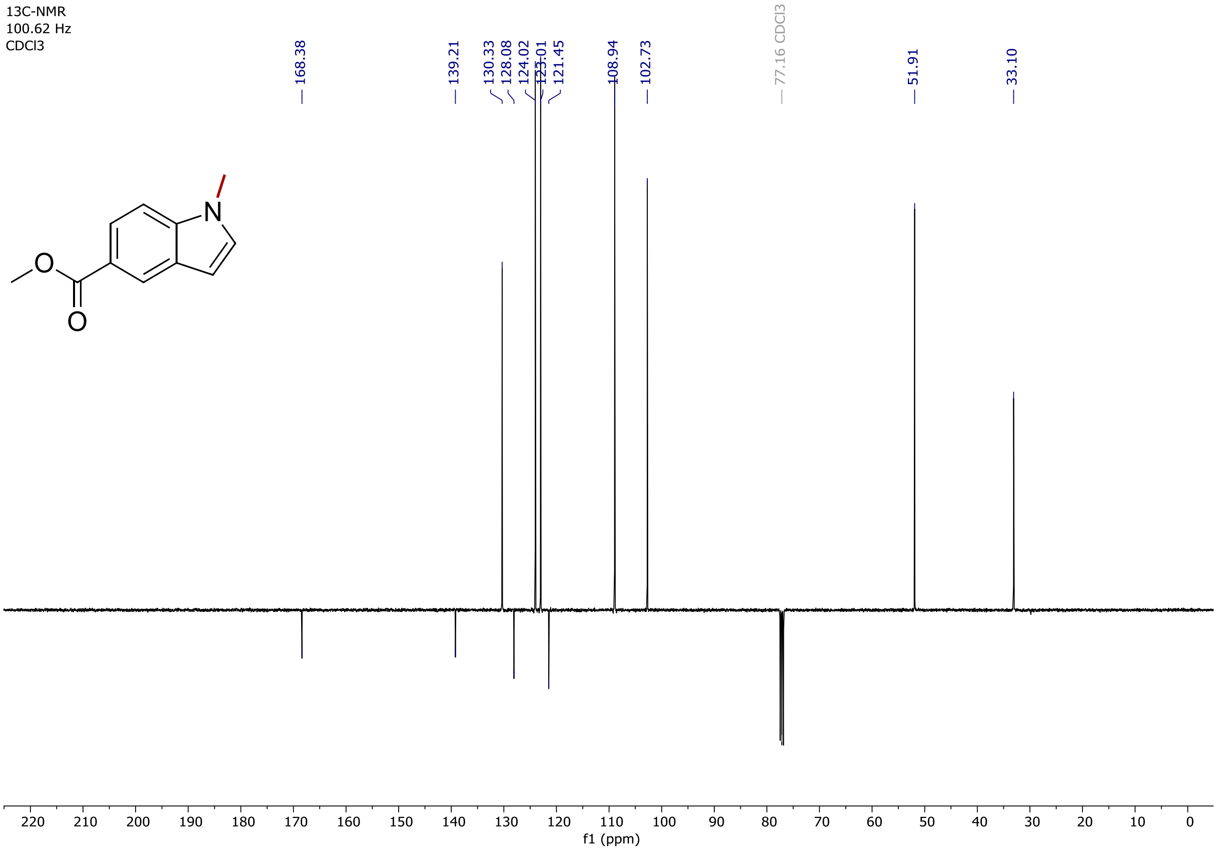

(1-Methyl-3-indolyl)acetonitrile (5k)

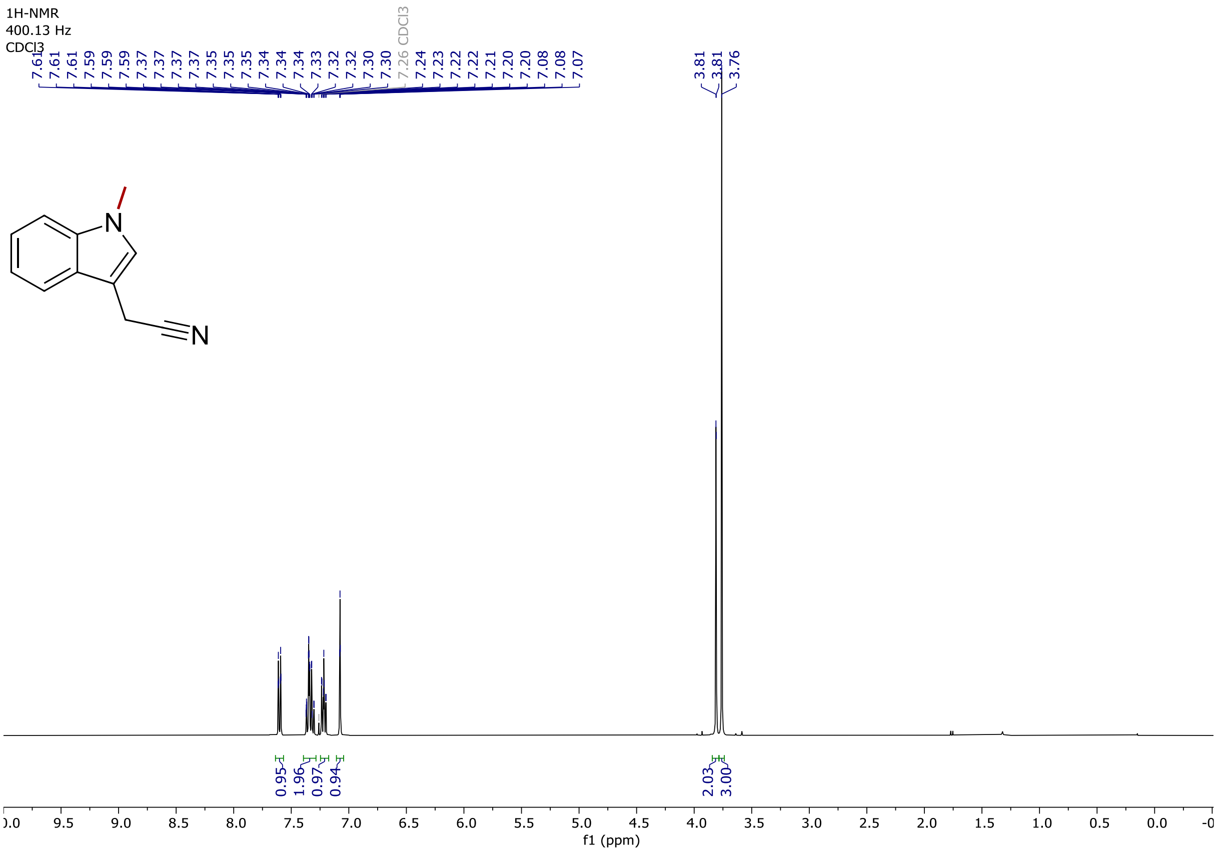

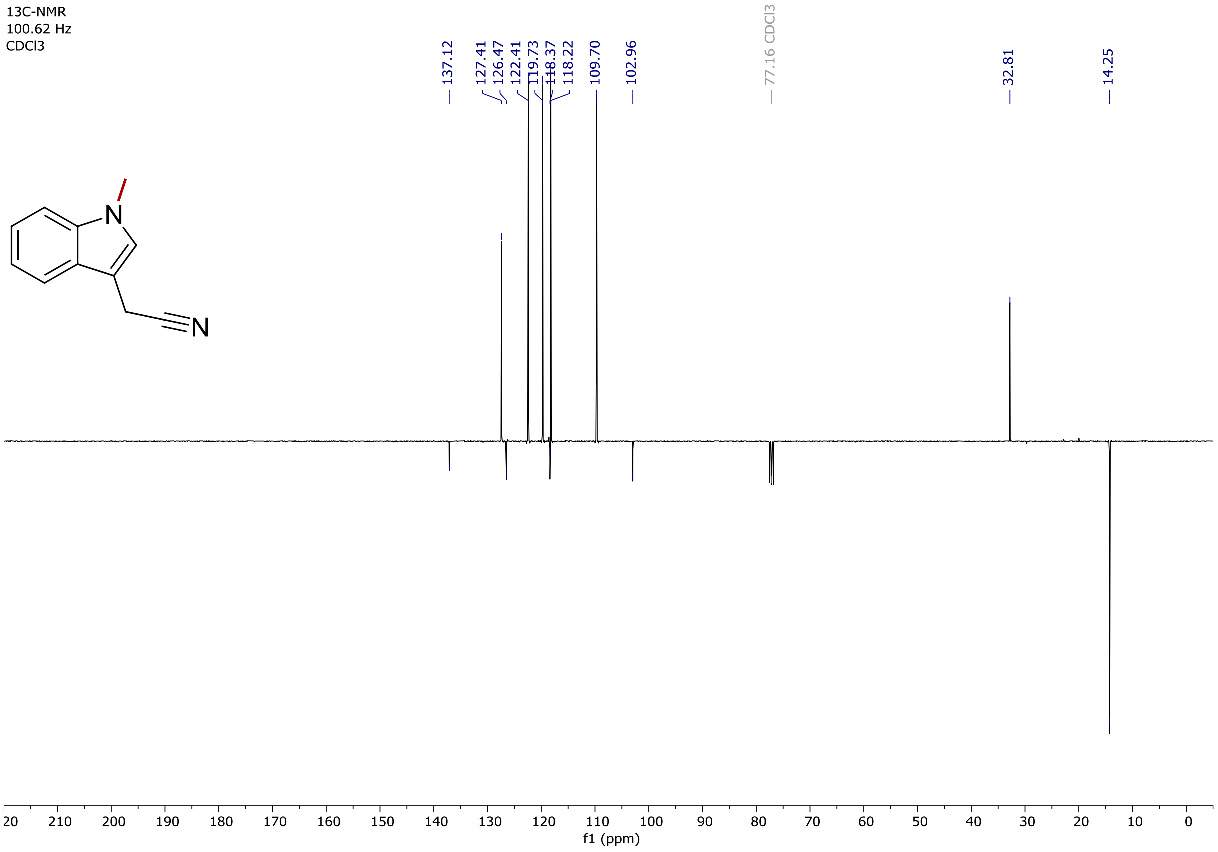

***N*-Methylmelatonine (9)**

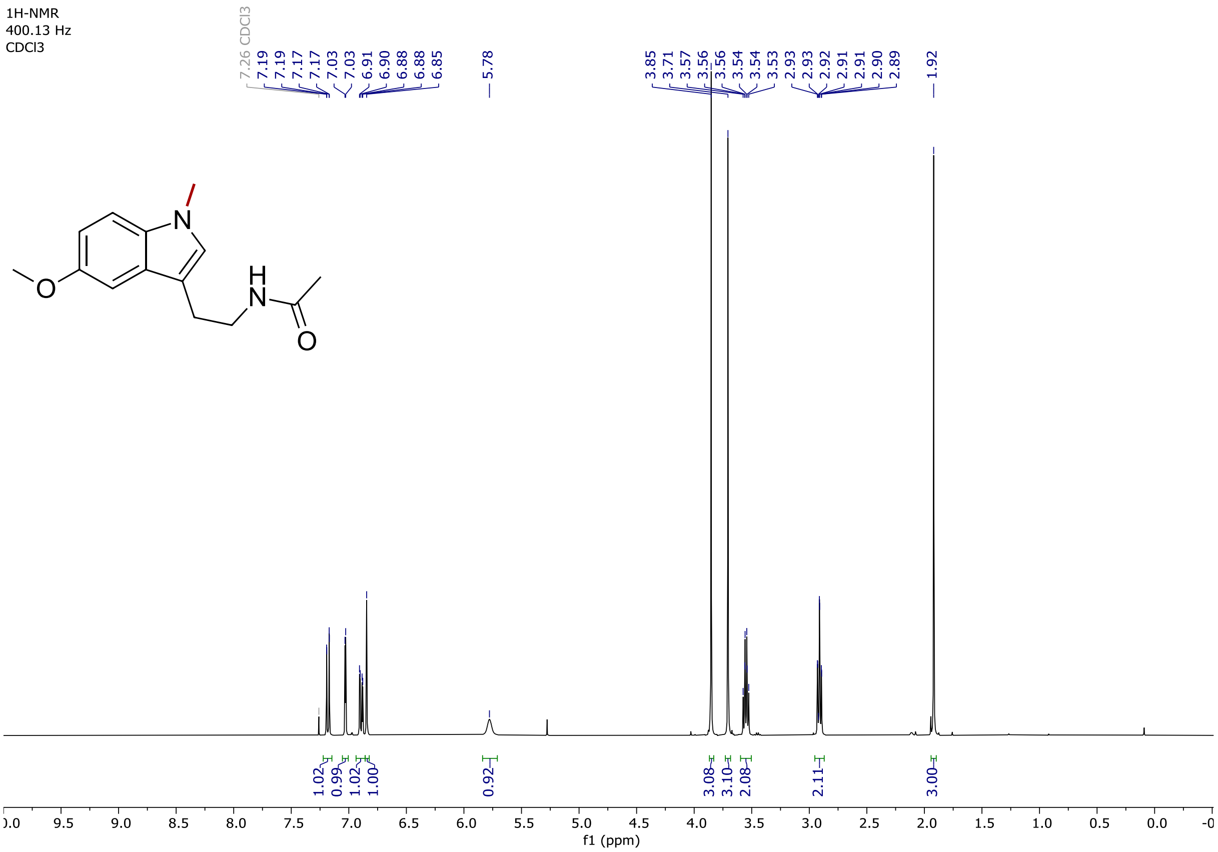

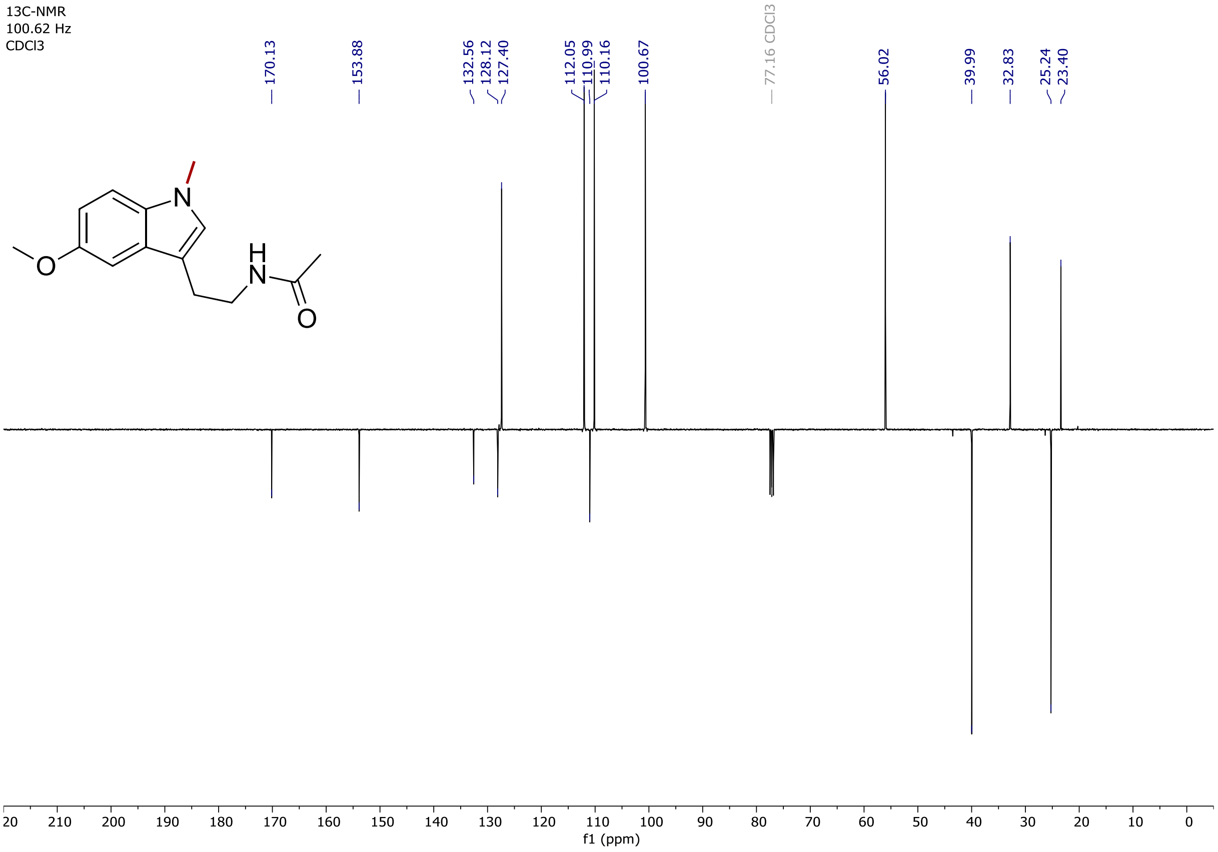

Caffeine (10)

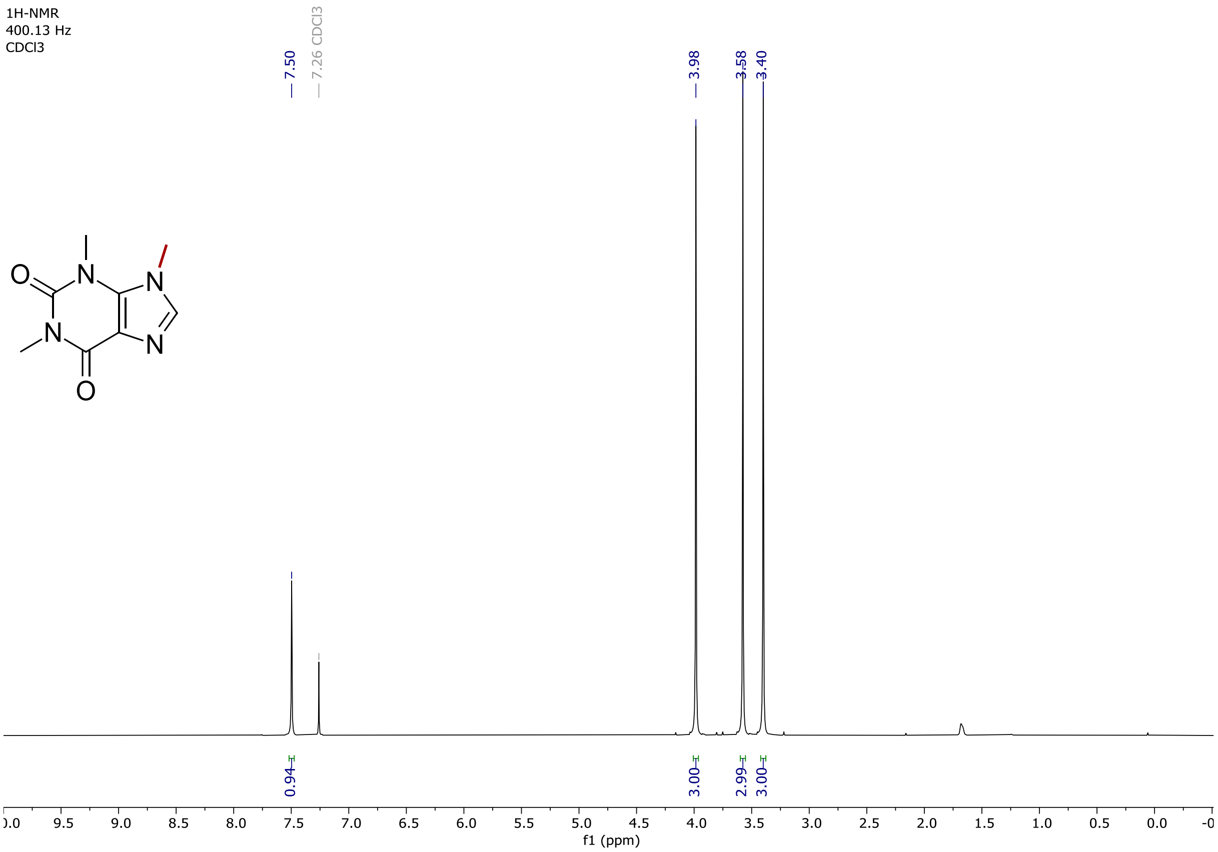

1H-NMR
400.13 Hz
CDCl3
7.50
7.26 CDCl3
3.98
3.58
3.40
0.94
3.00
2.99
3.00
f1 (ppm)

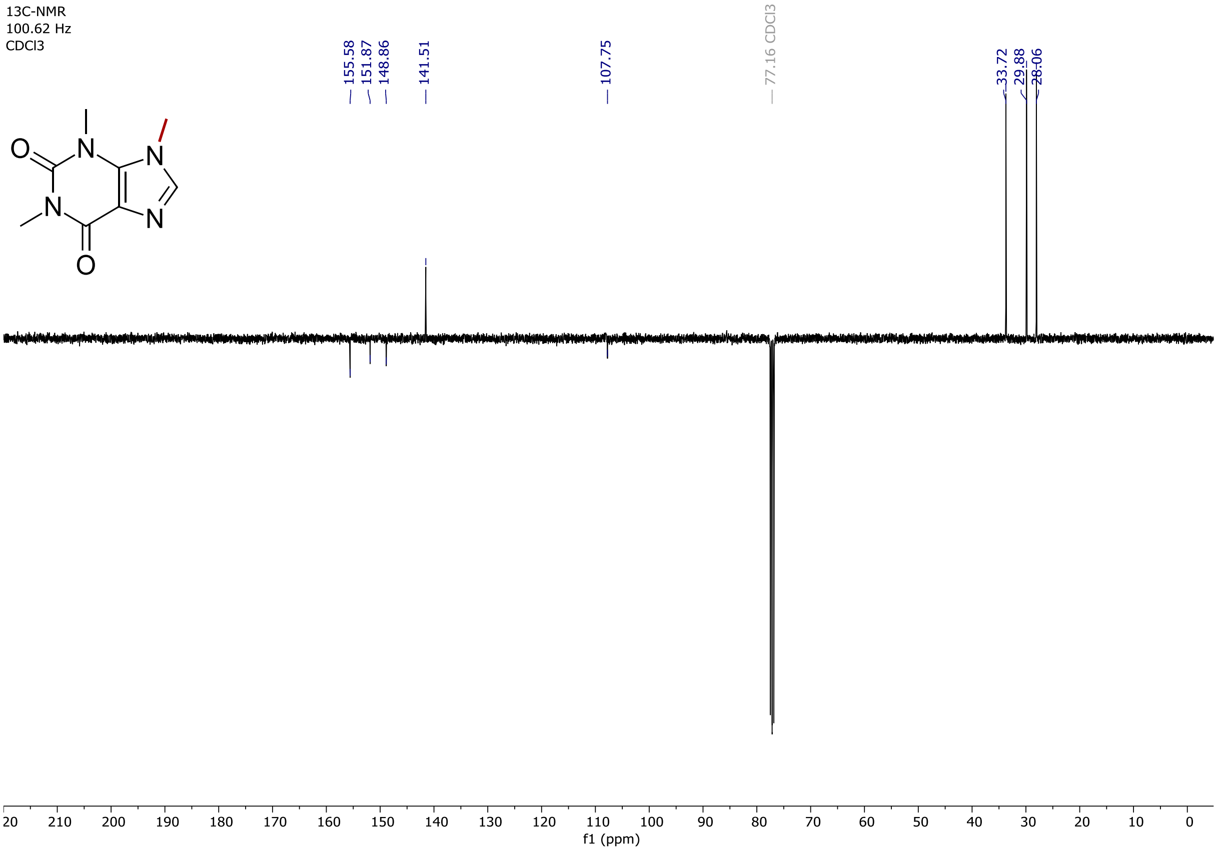

13C-NMR
100.62 Hz
CDCl3
155.58
151.87
148.86
141.51
107.75
77.16 CDCl3
33.72
29.88
28.06
f1 (ppm)

***N,N*-Dimethyl celecoxib (11)**

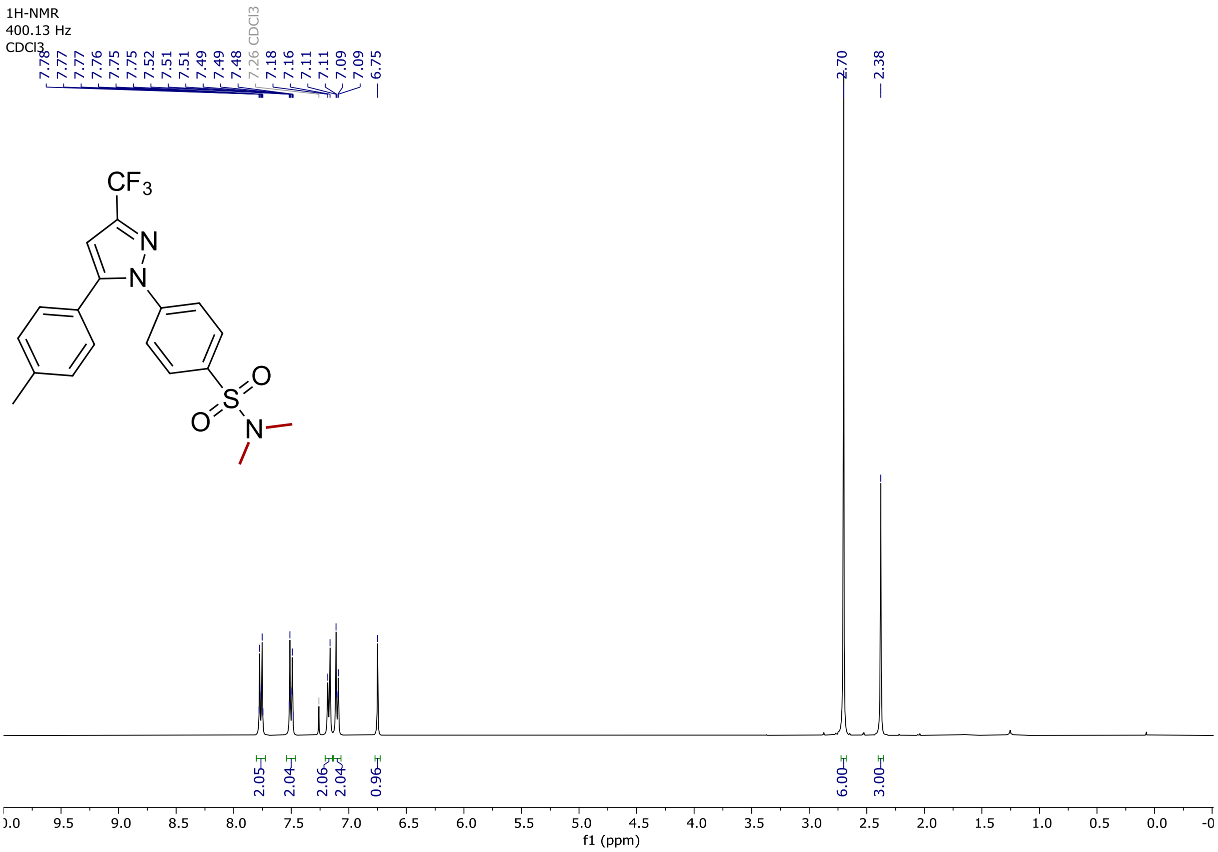

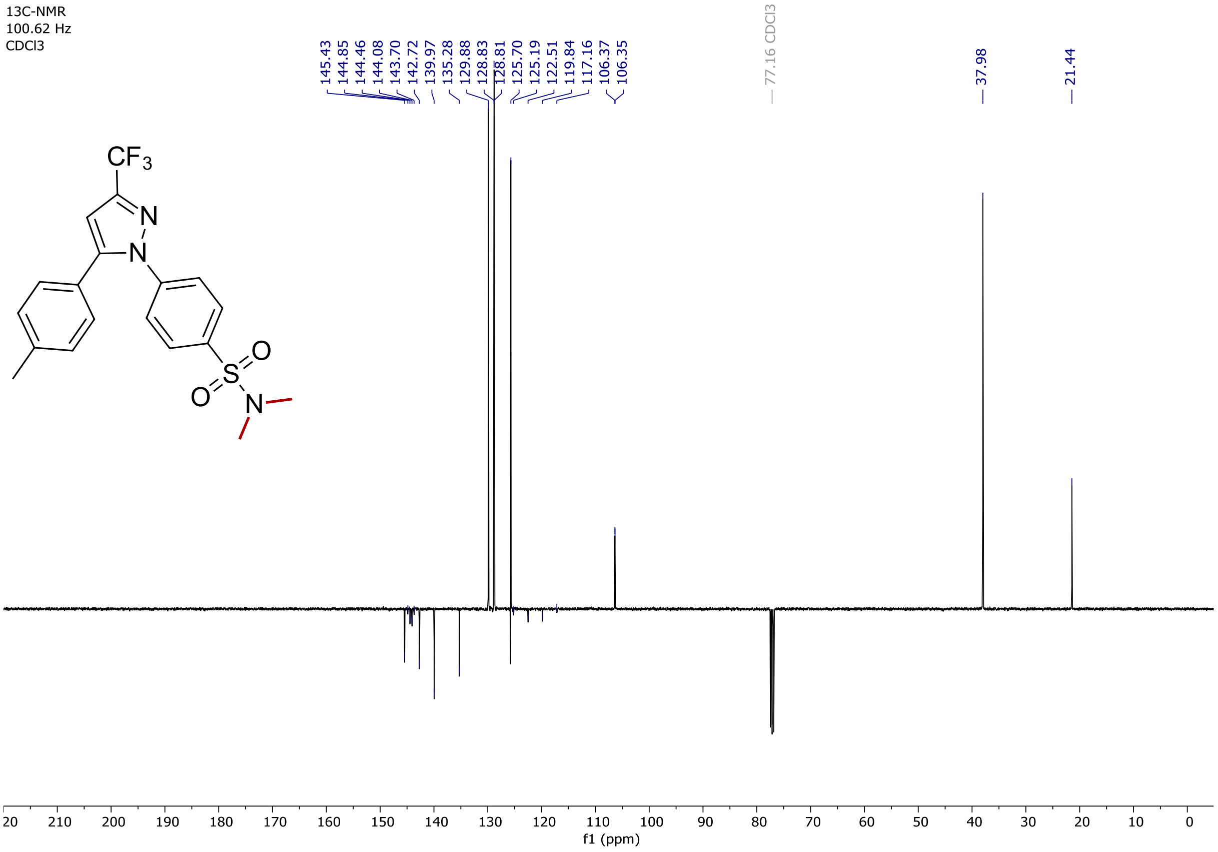

N-Methyl carbamazepine (12)

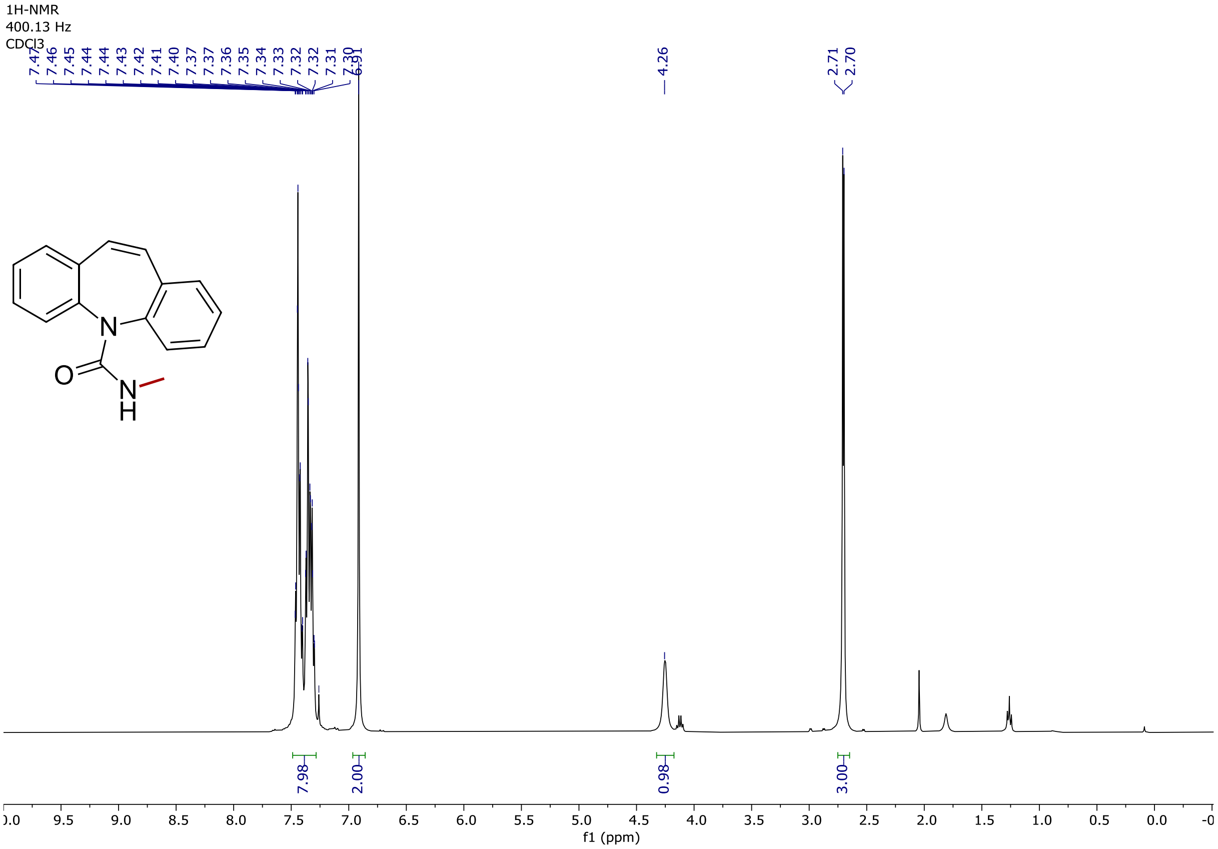

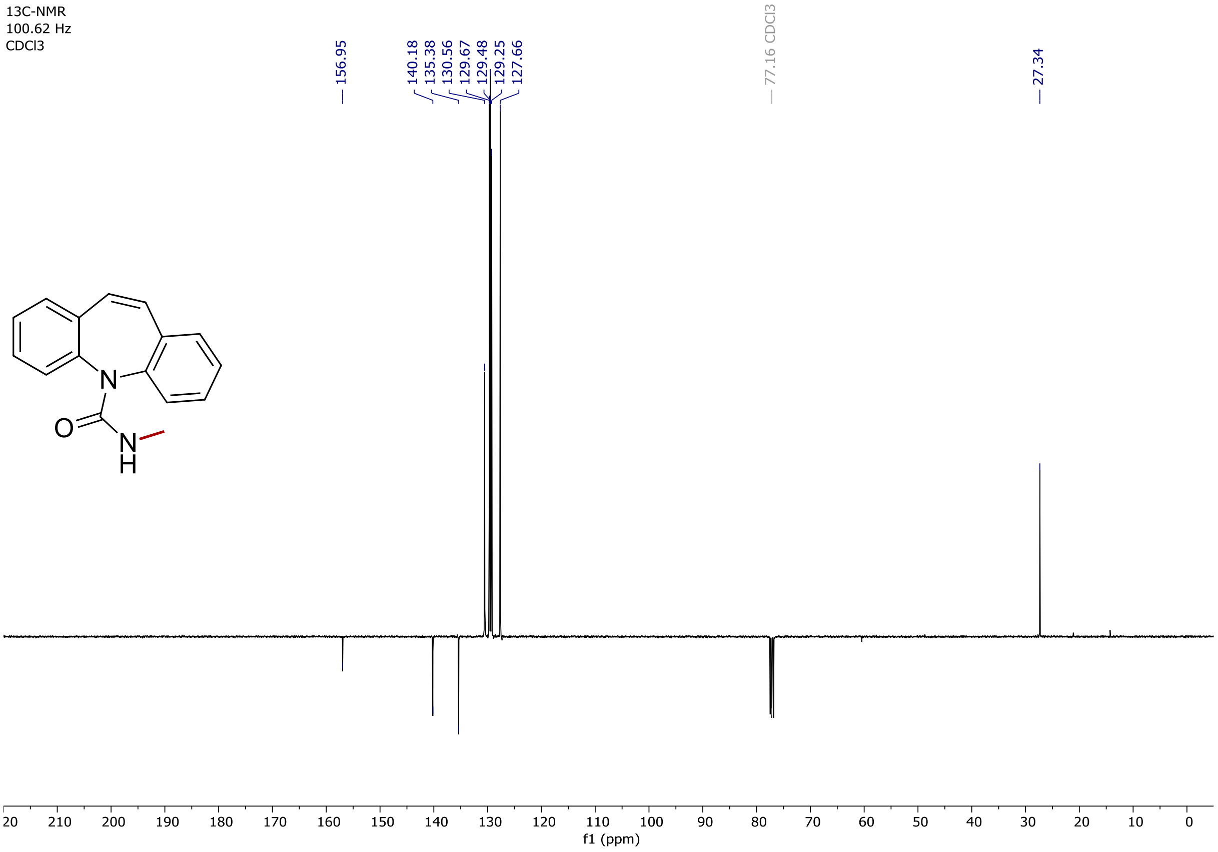

***O,N*-Dimethyl paracetamol (13)**

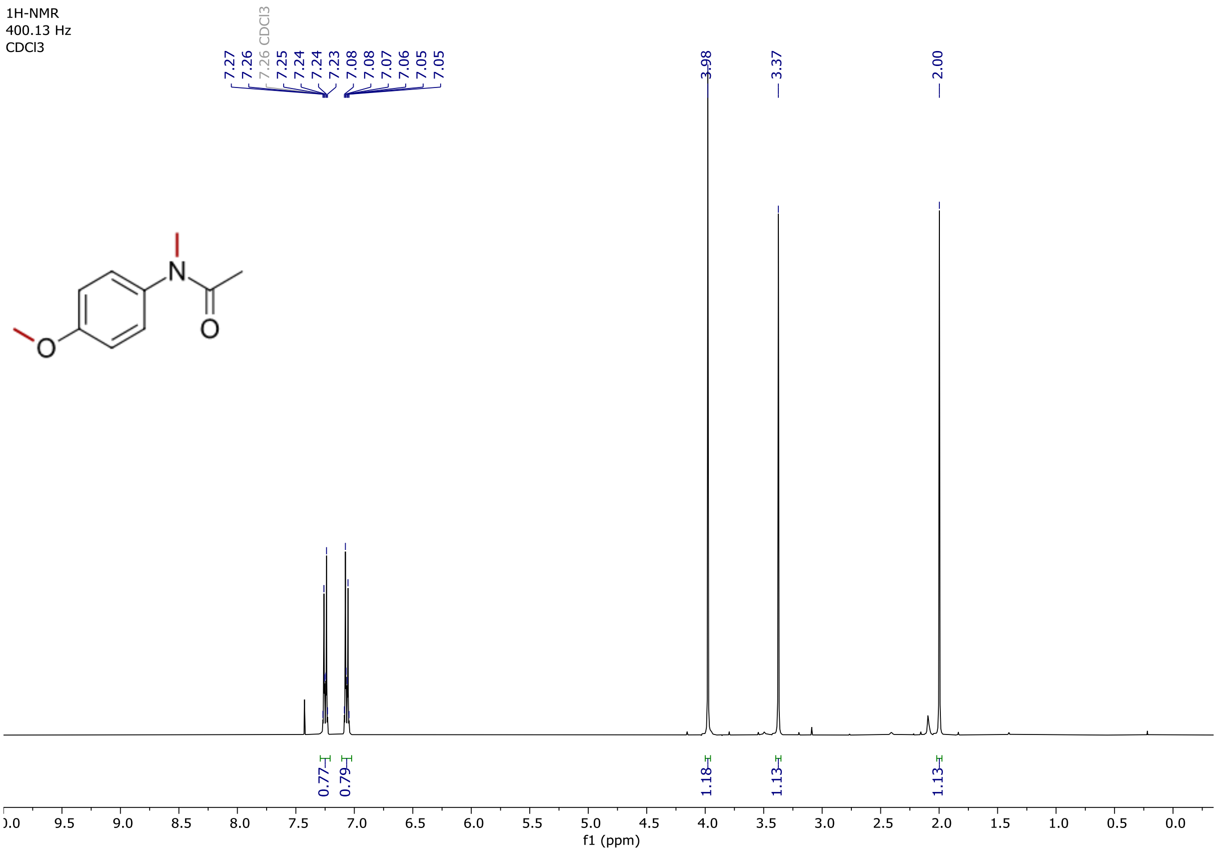

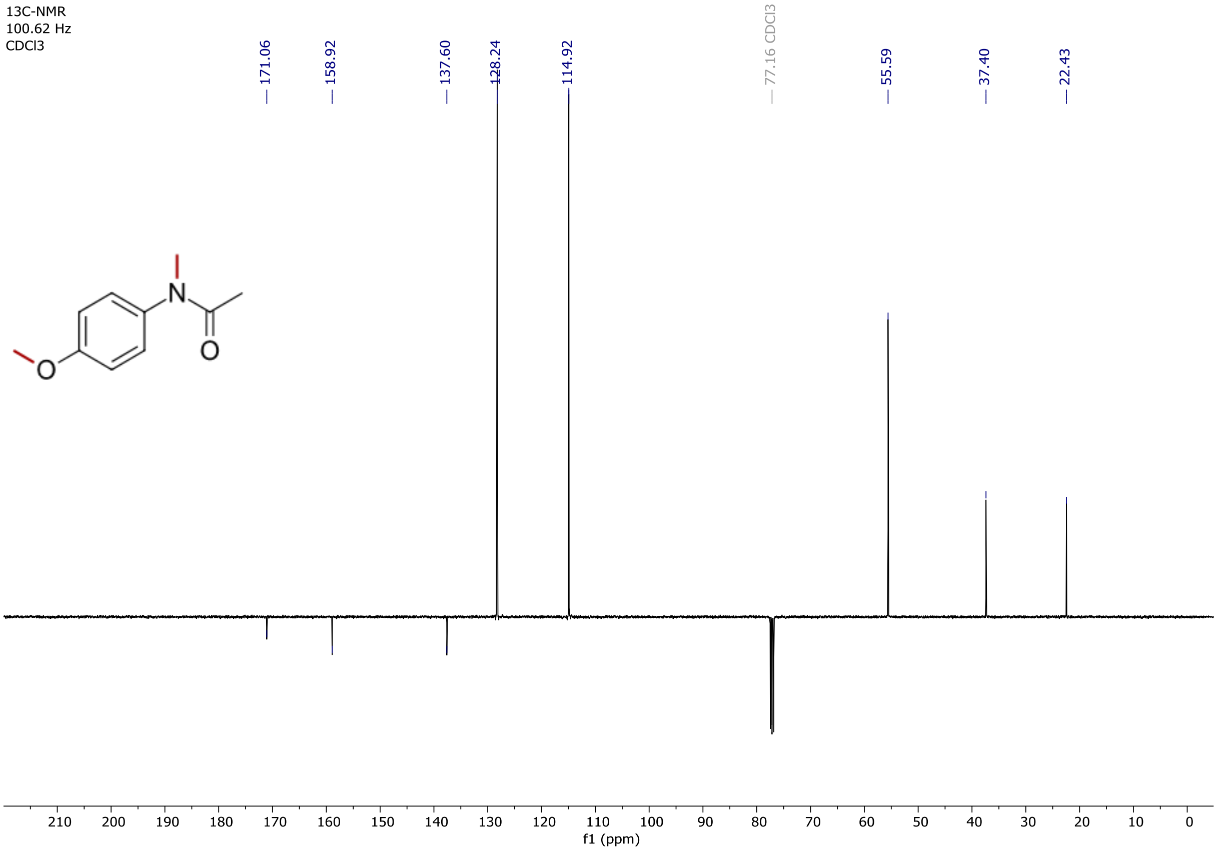

O,N-Dimethyl salicylamide (14)

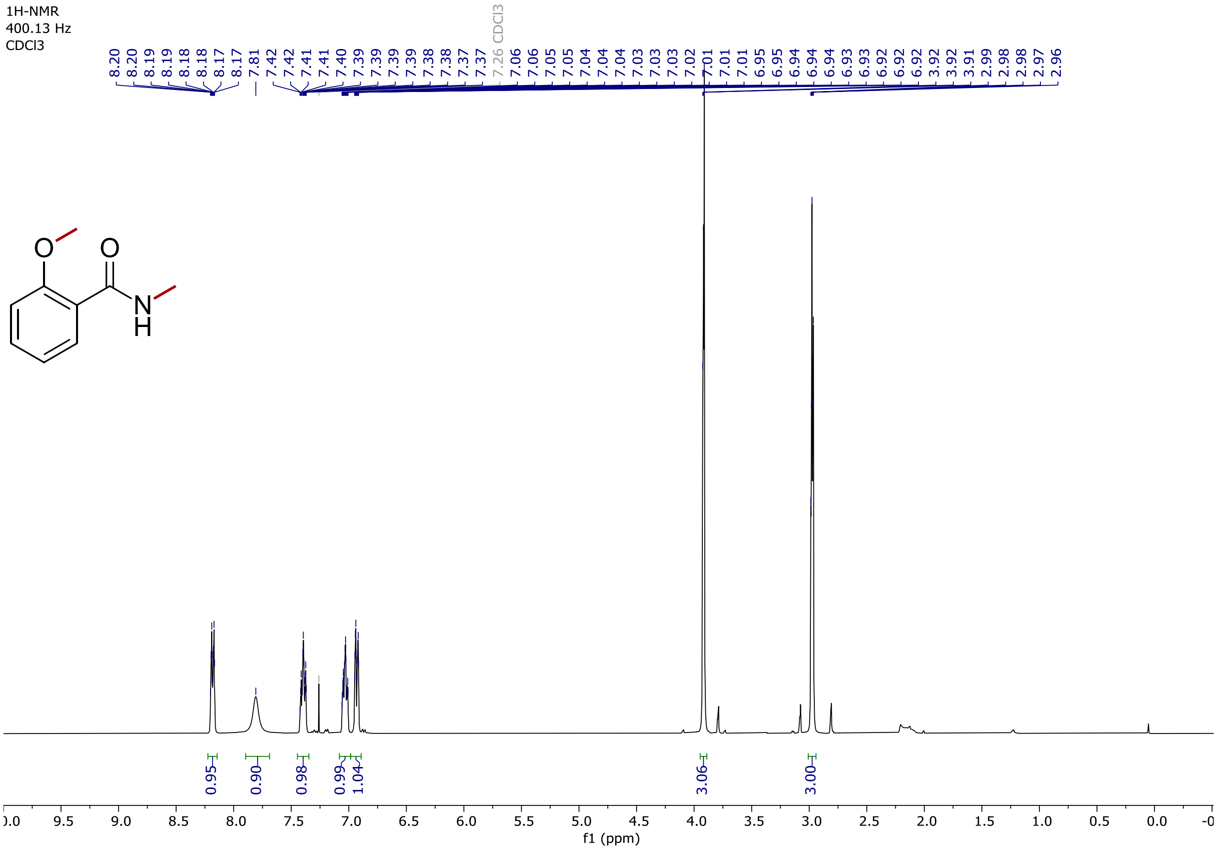

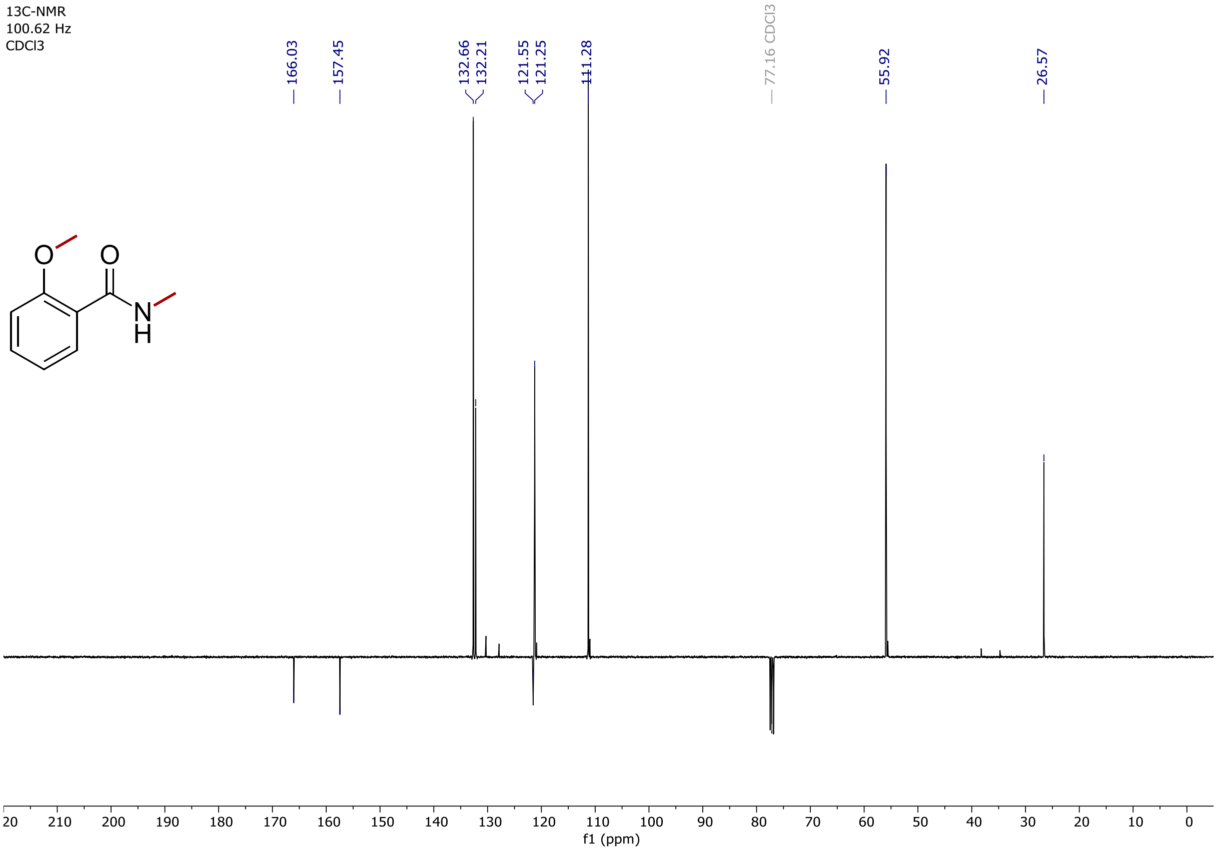

***N*-Methyl linezolid (15)**

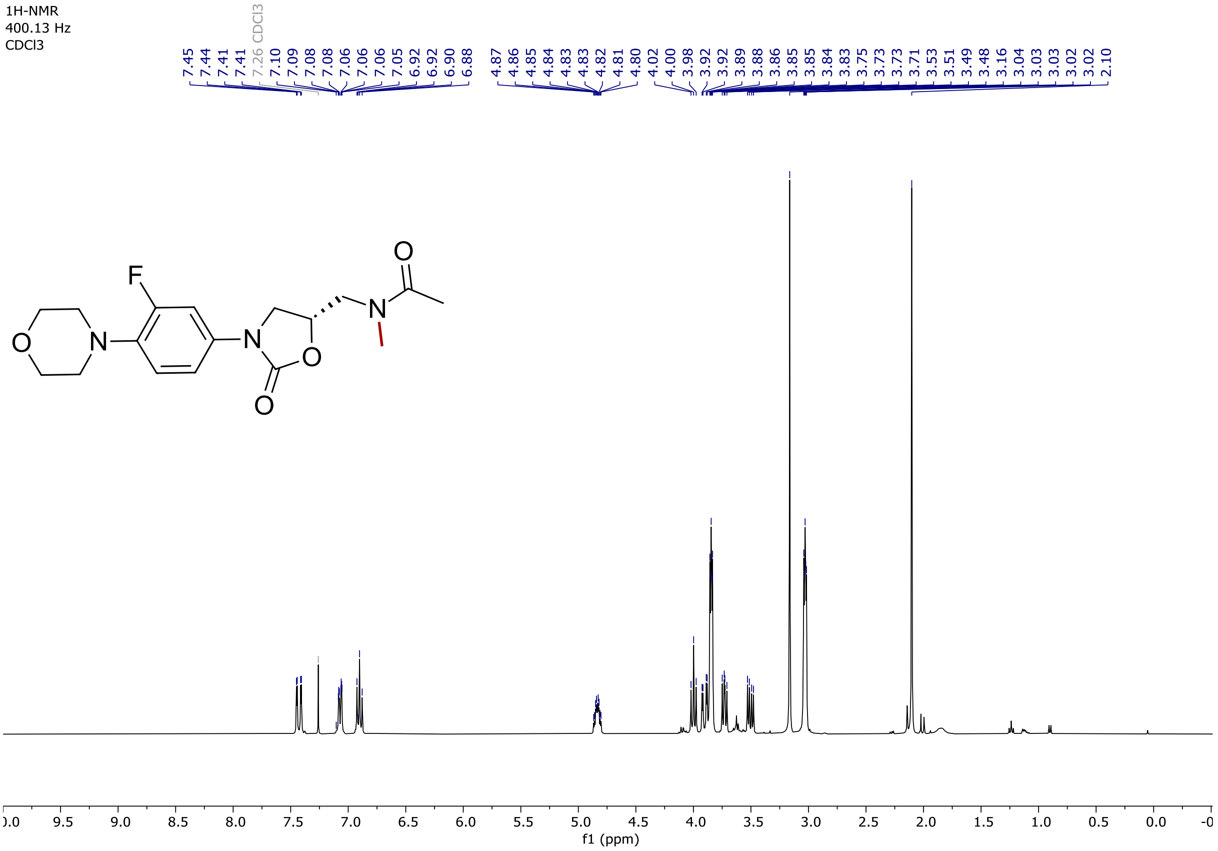

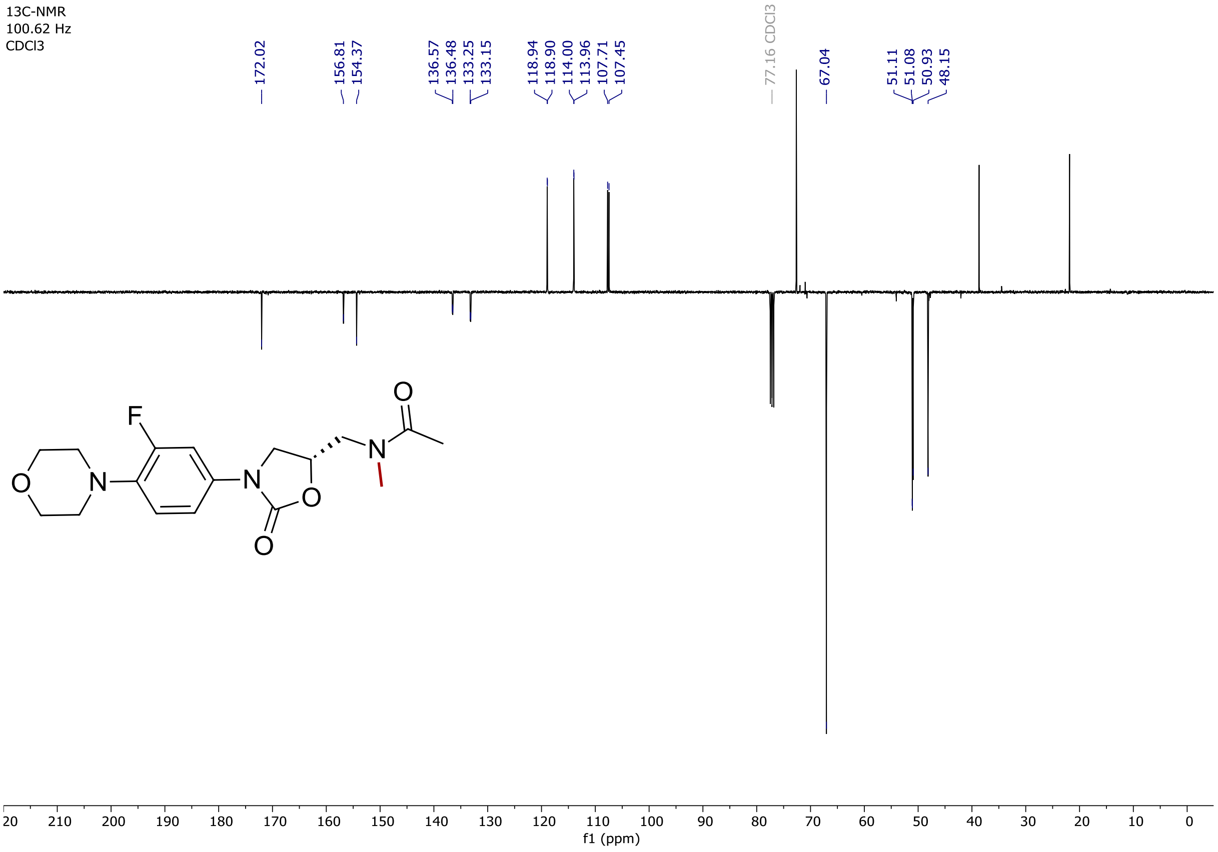

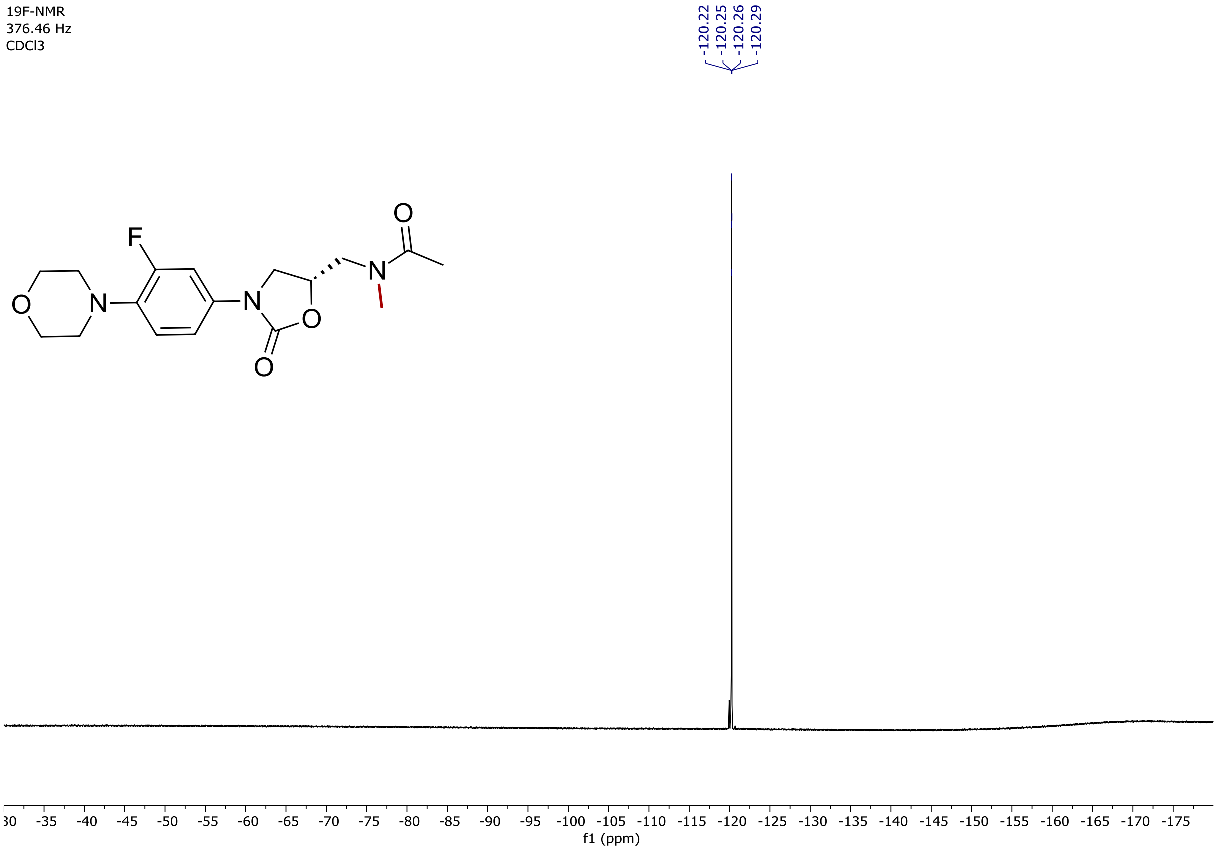
19F-NMR
376.46 Hz
CDCl3
-120.22
-120.25
-120.26
-120.29
F
O
N
N
O
O
N
O
f1 (ppm)

***N*-Ethyl-4-fluorobenzamide (4a)**

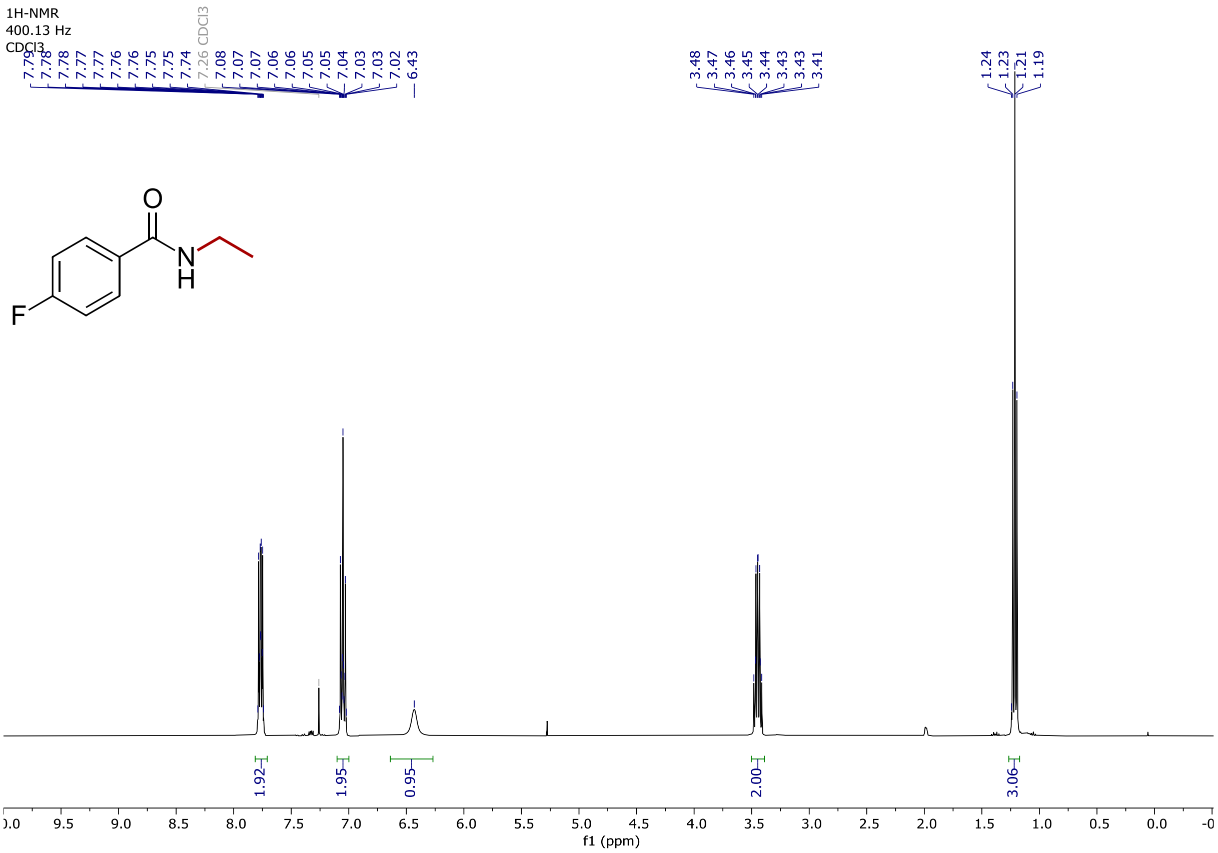

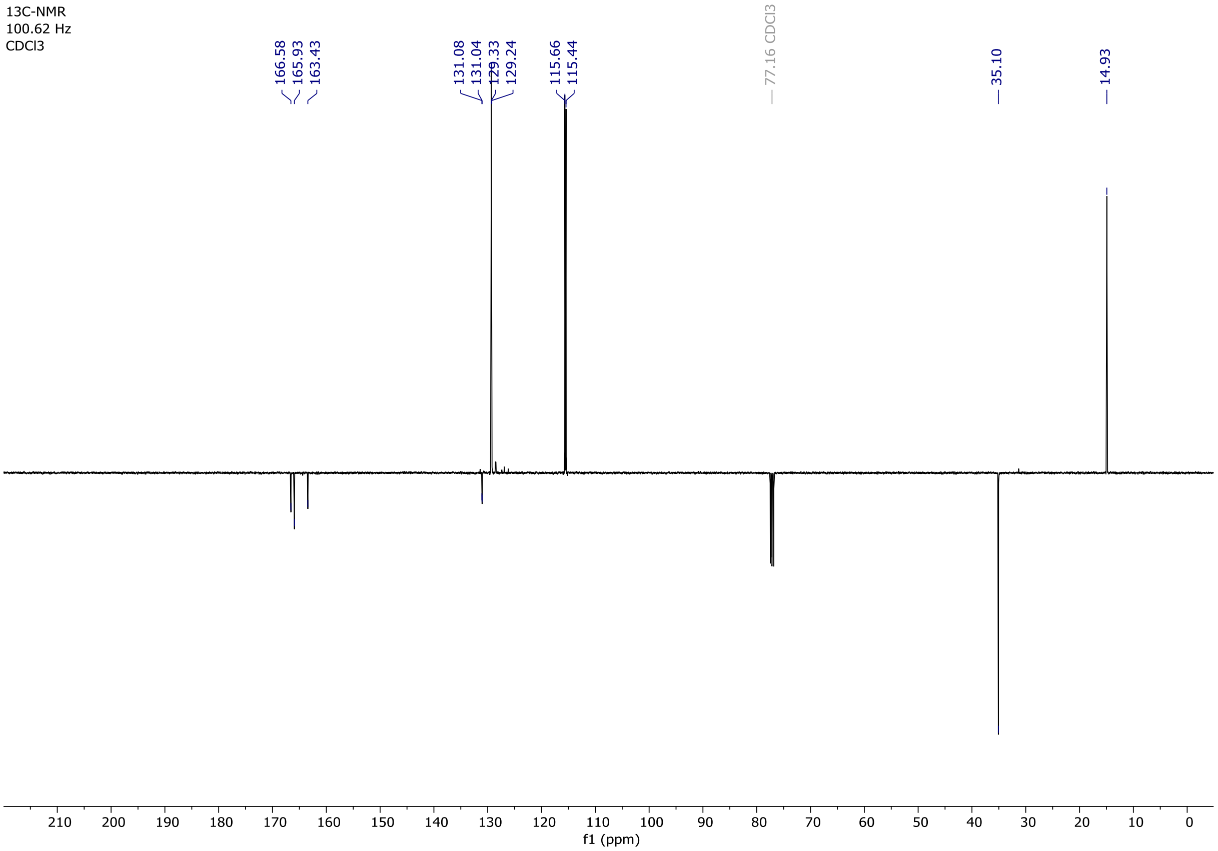

***N*-Ethylbenzamide (4b)**

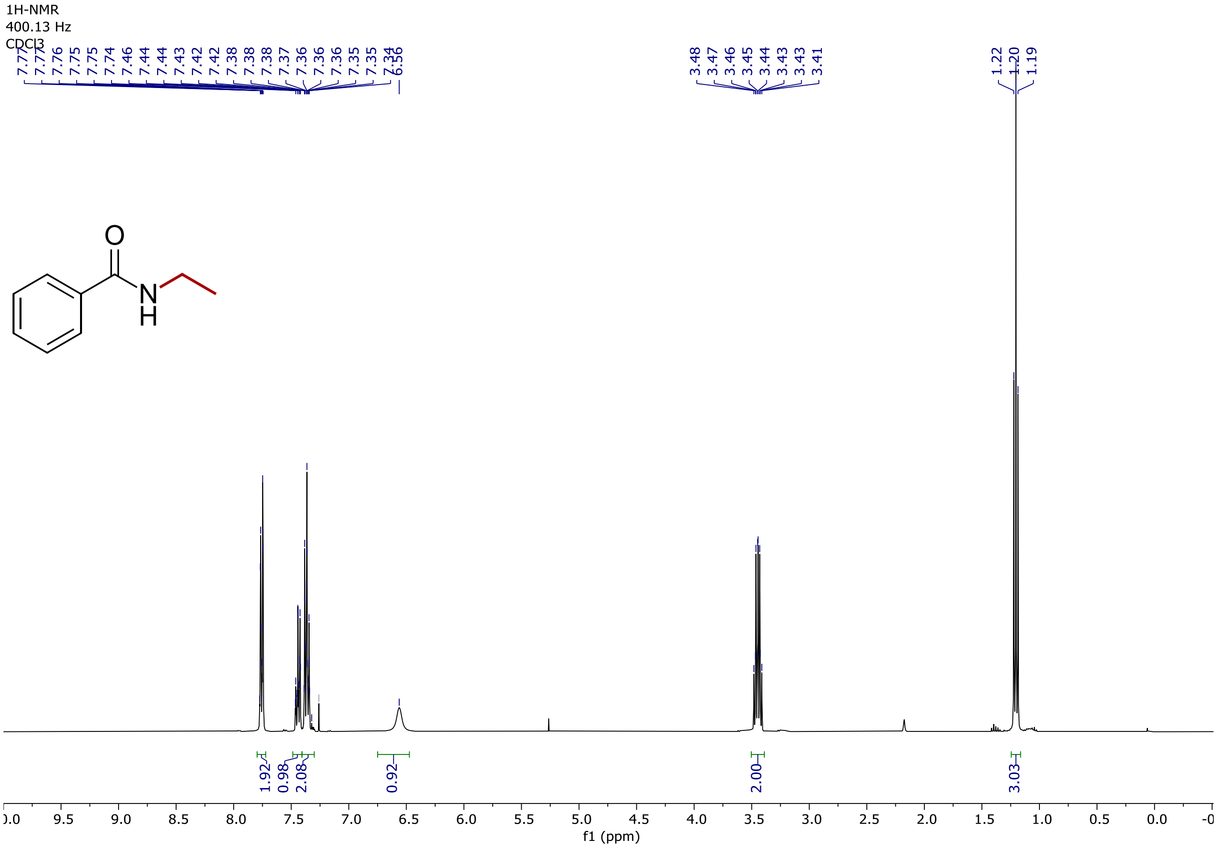

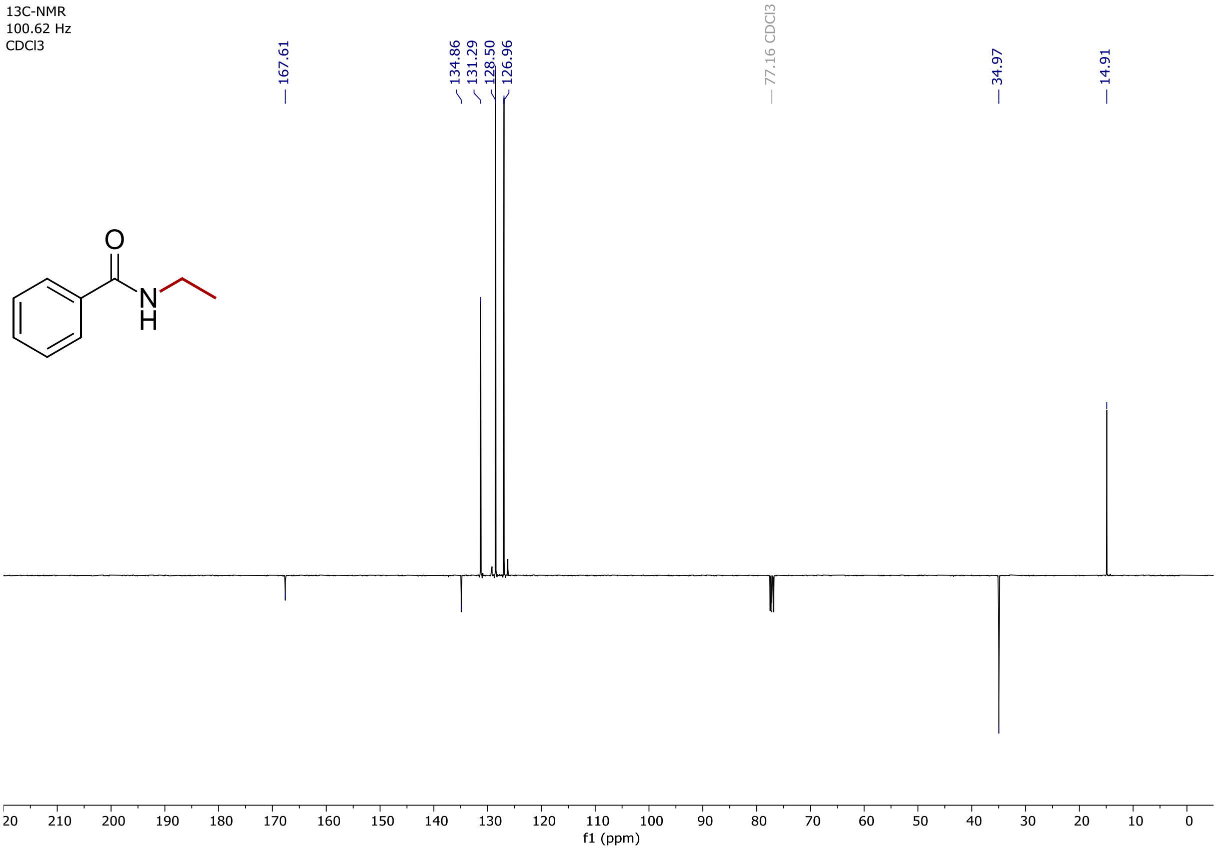

4-Bromo-*N*-ethylbenzamide (4c)

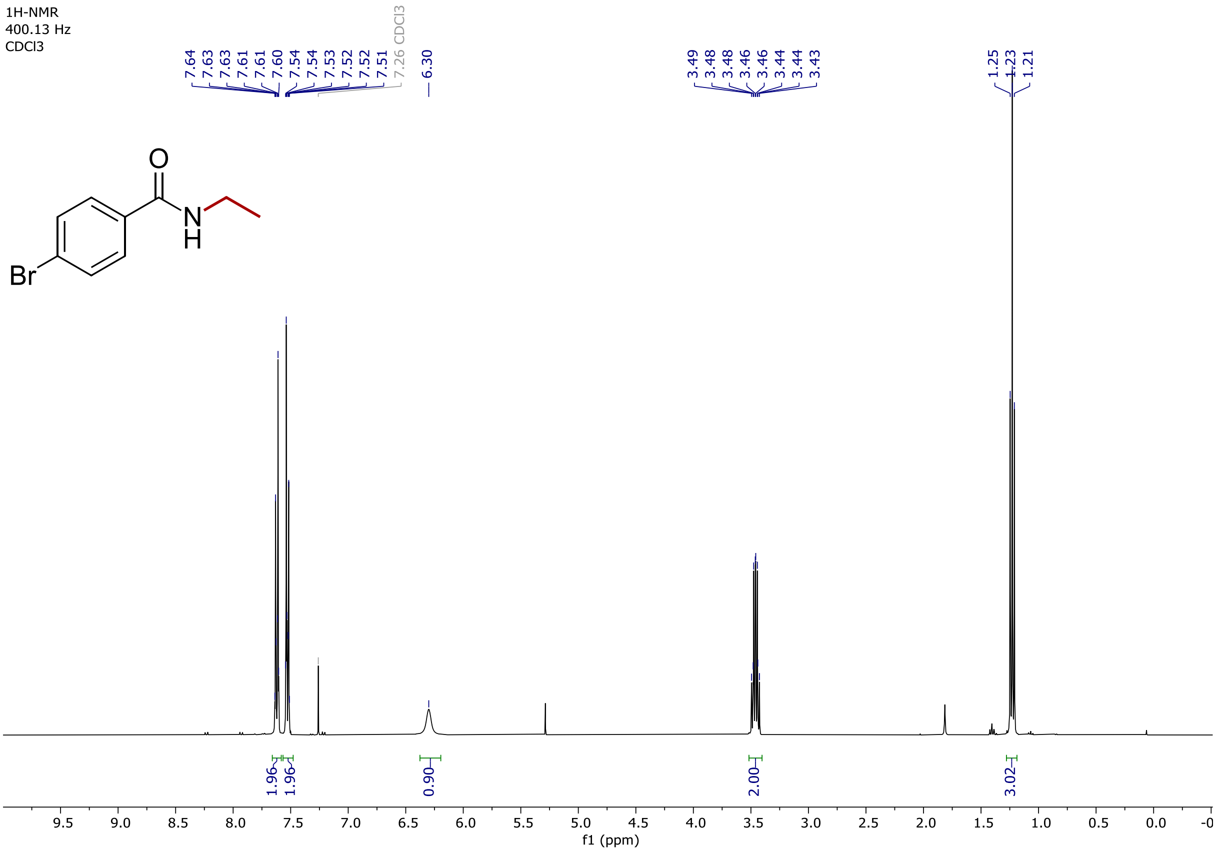

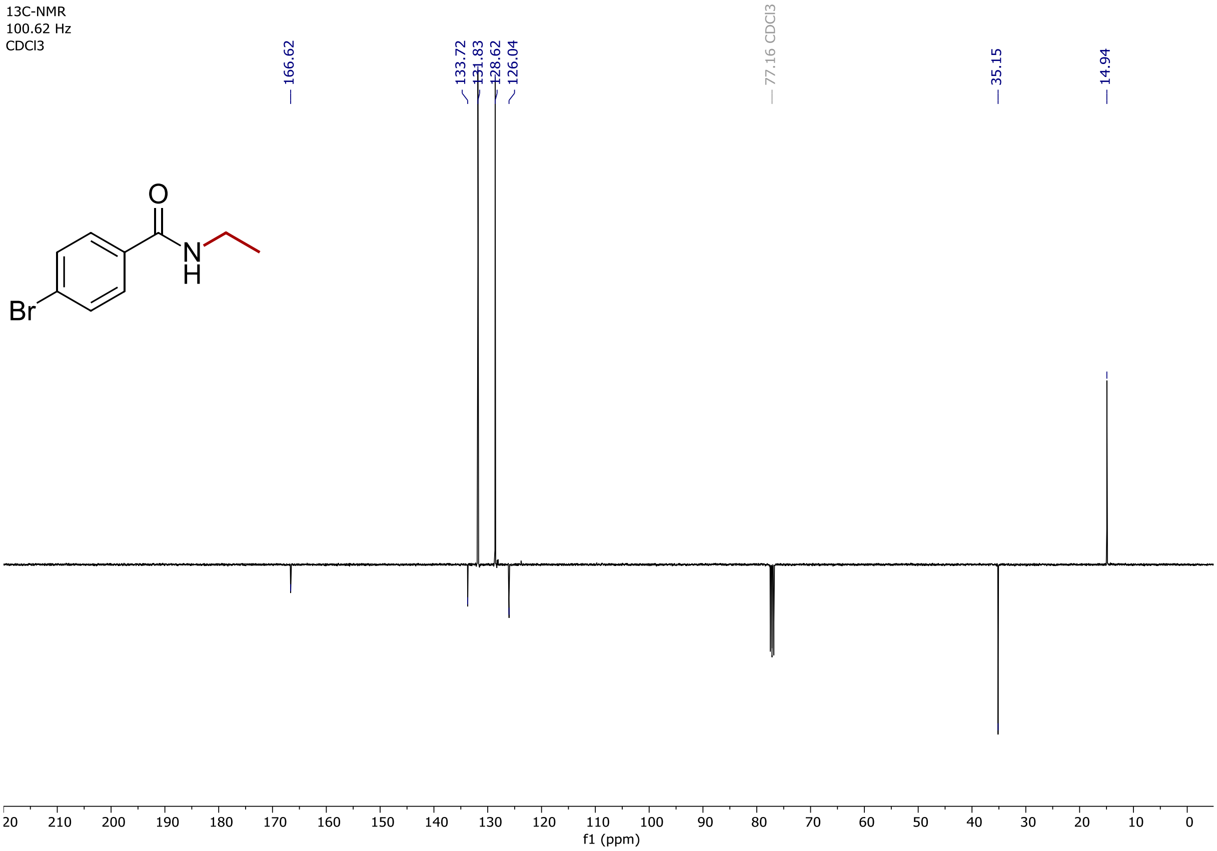

3-Bromo-*N*-ethylbenzamide (4d)

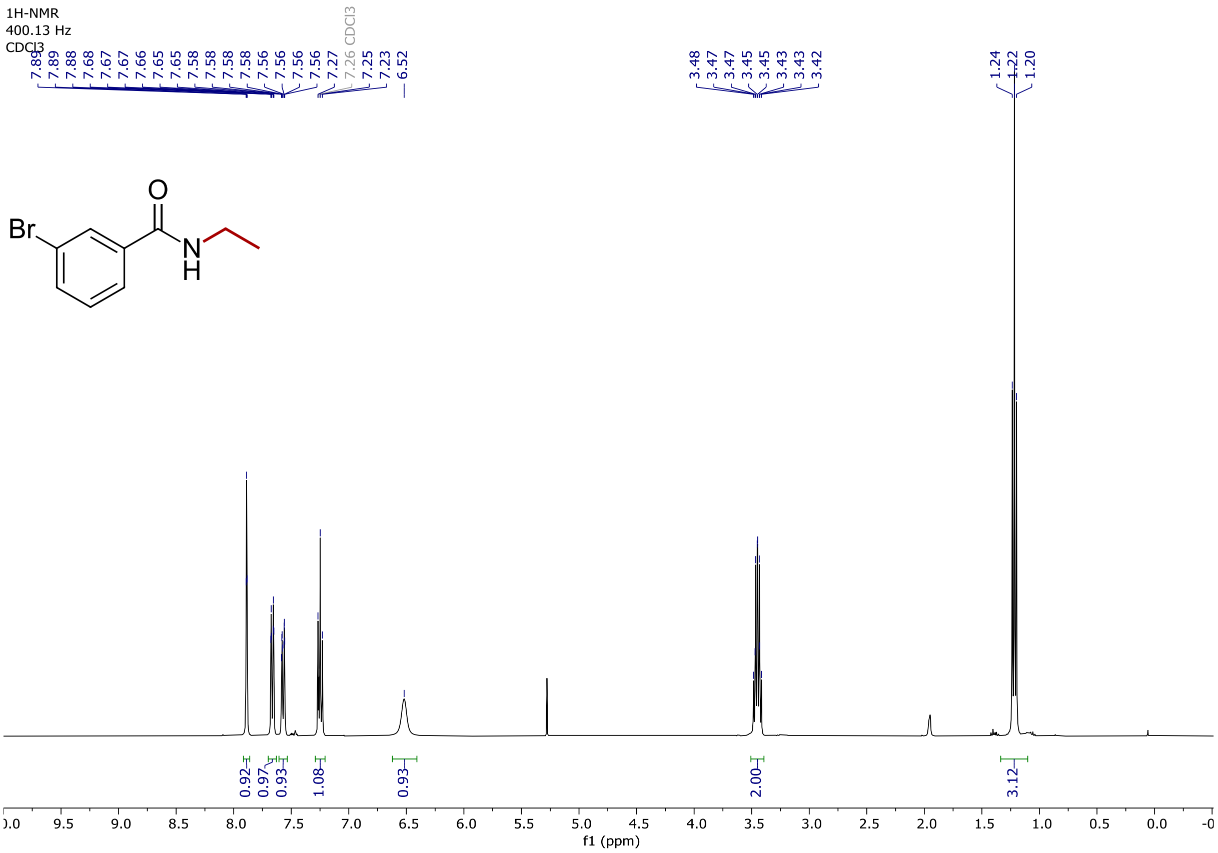

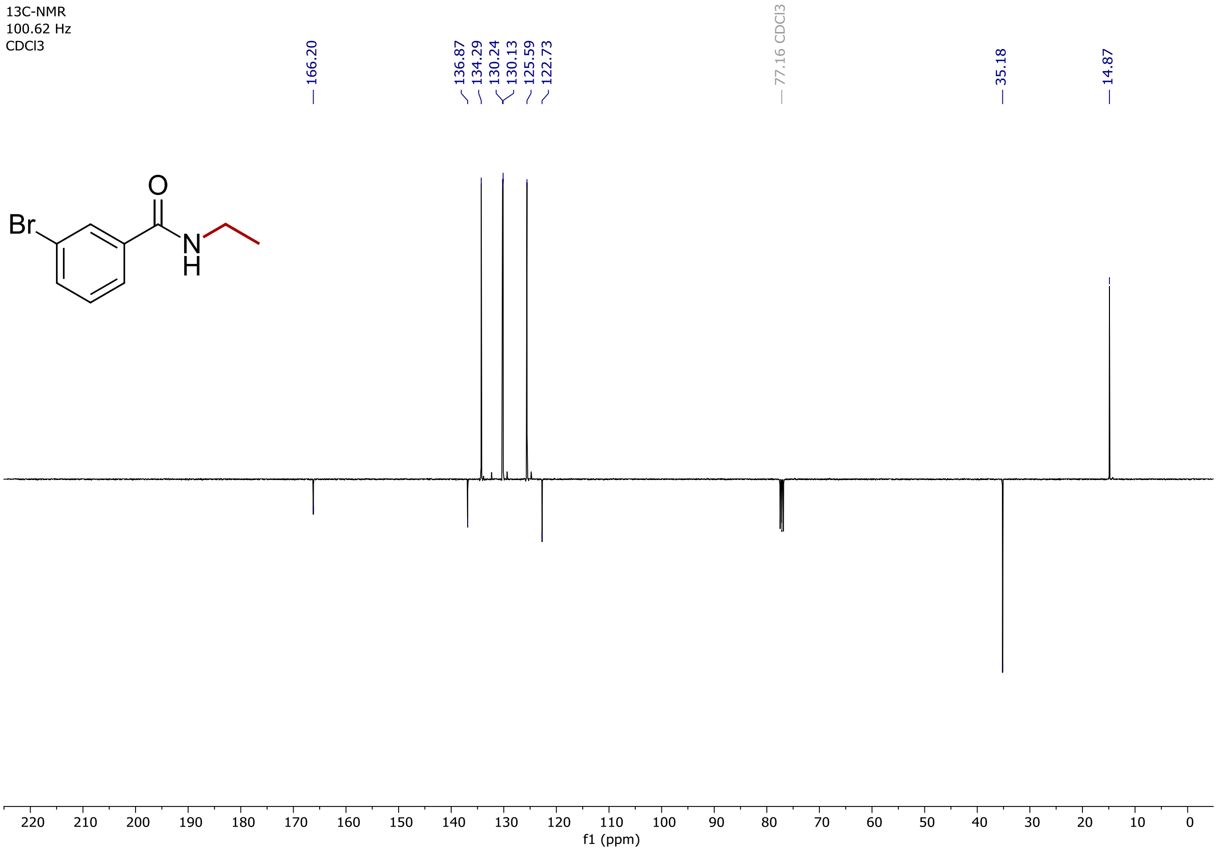

2-Bromo-*N*-ethylbenzamide (4e)

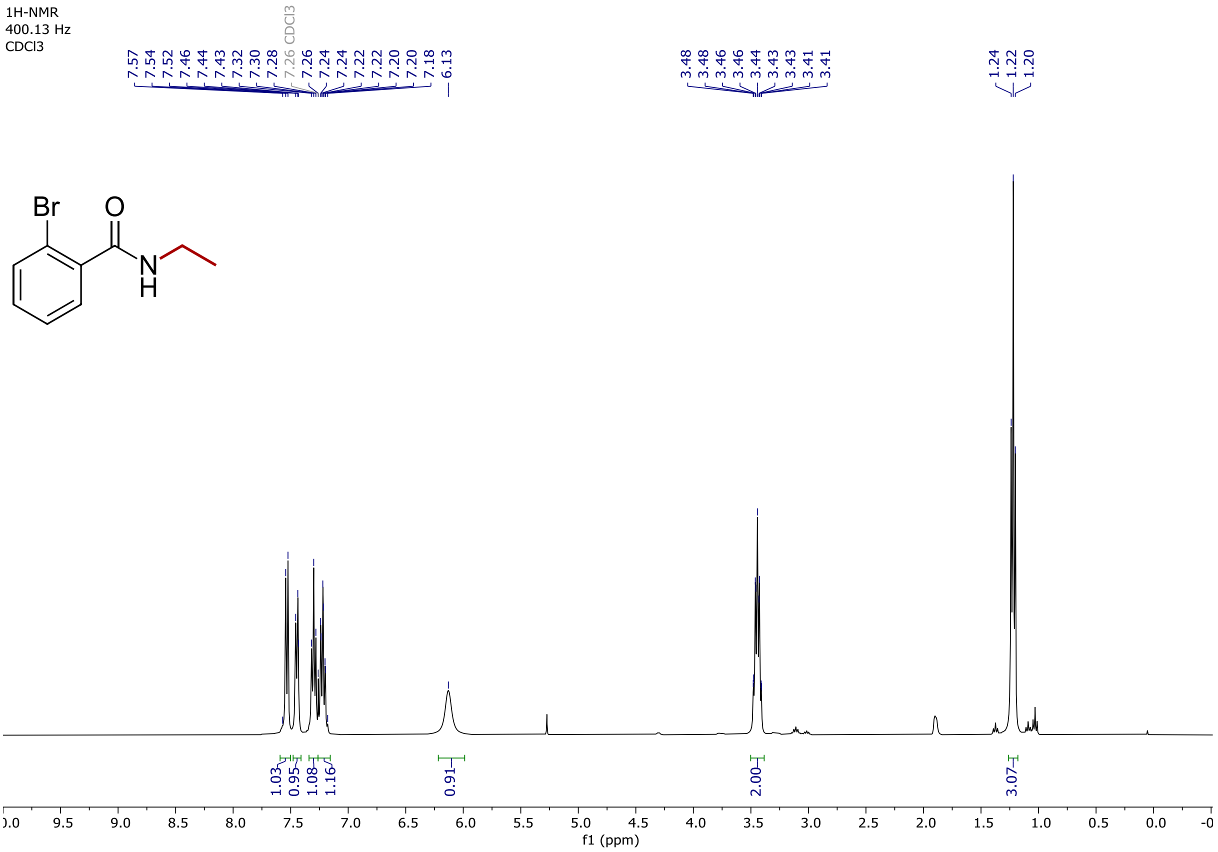

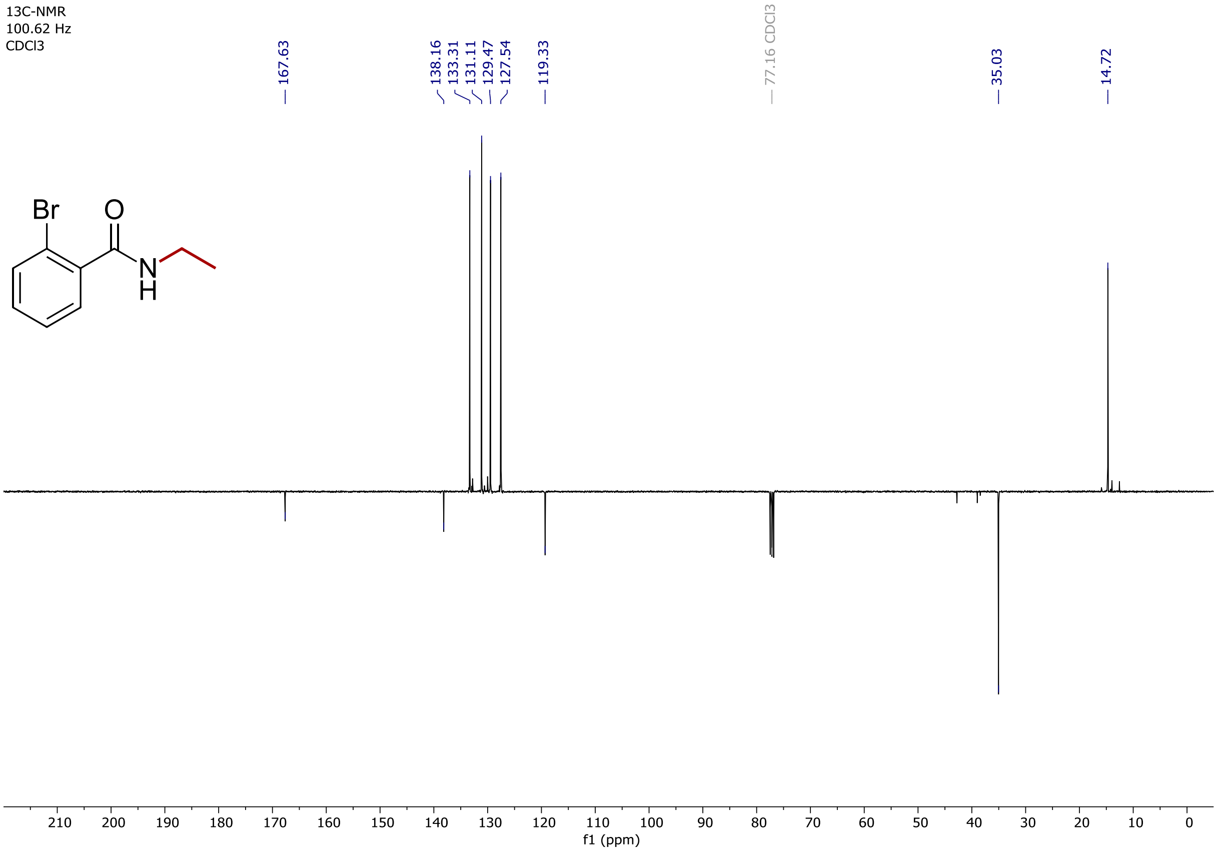

N-Ethyl-4-(trifluoromethyl) benzamide (4f)

1H-NMR
400.13 Hz
CDCl3

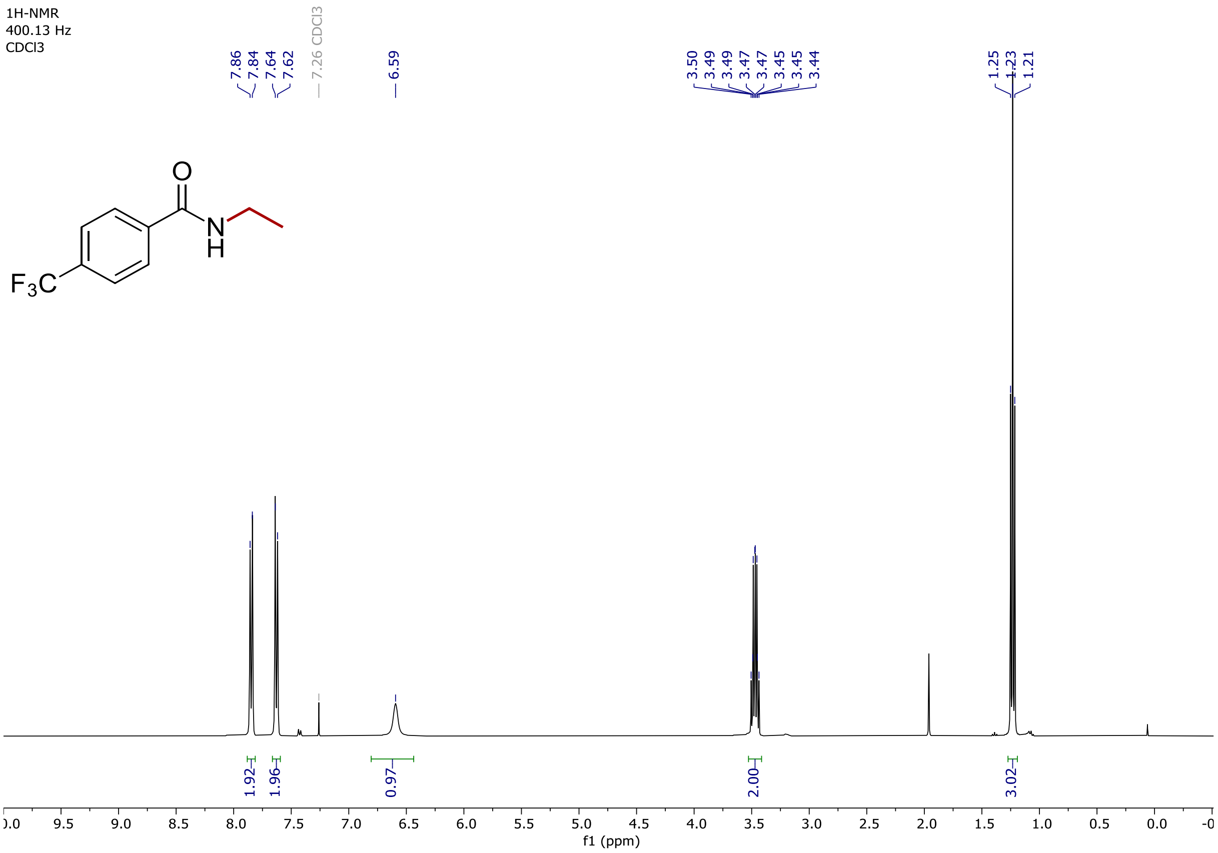

13C-NMR
100.62 Hz
CDCl3

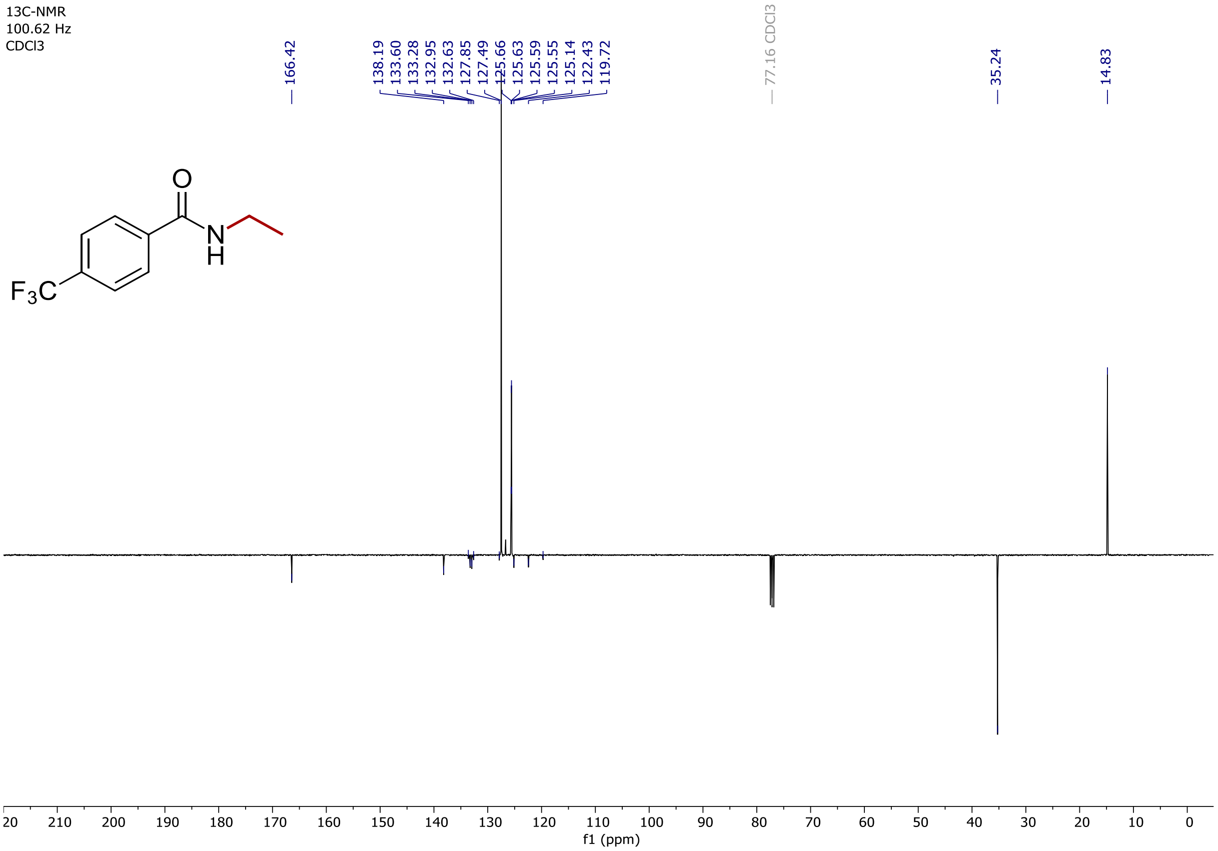

4-Chloro-*N*-ethylbenzamide (4g)

1H-NMR
400.13 Hz
CDCl3

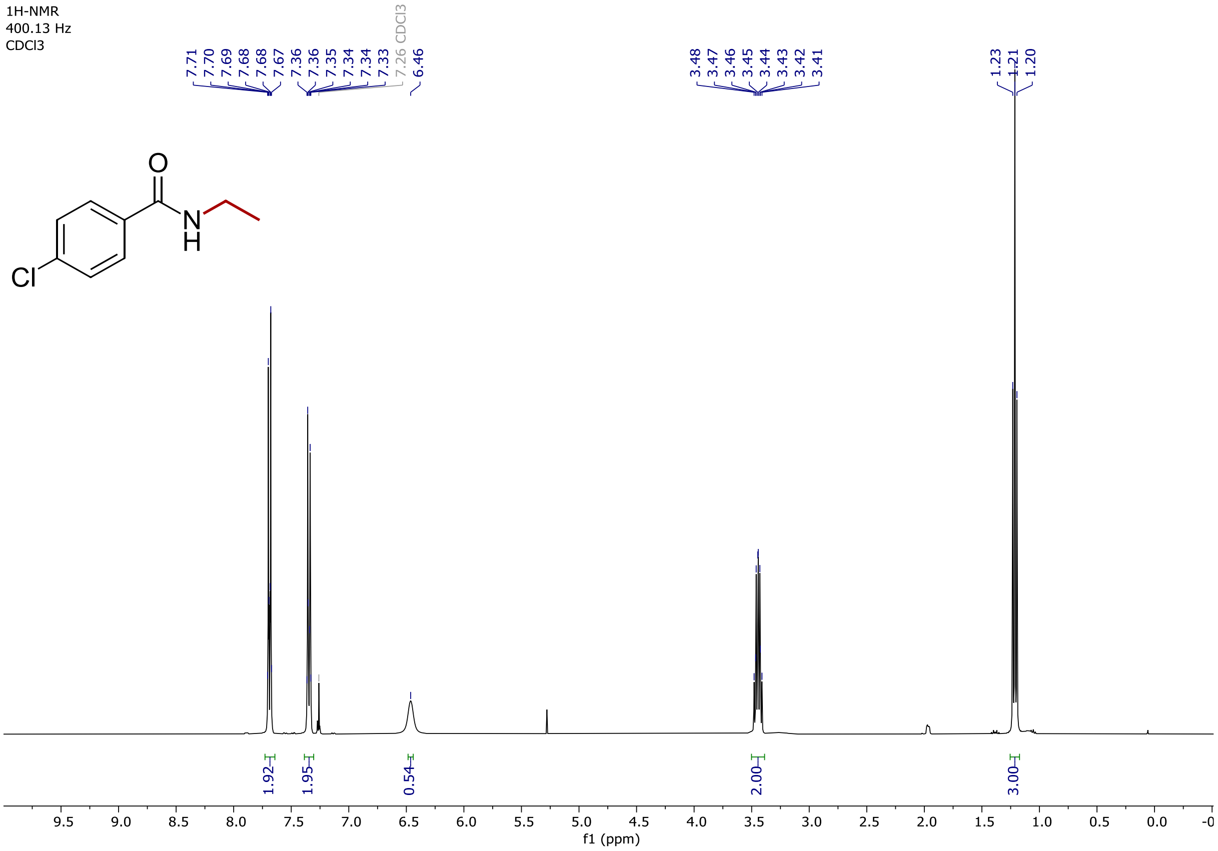

13C-NMR
100.62 Hz
CDCl3

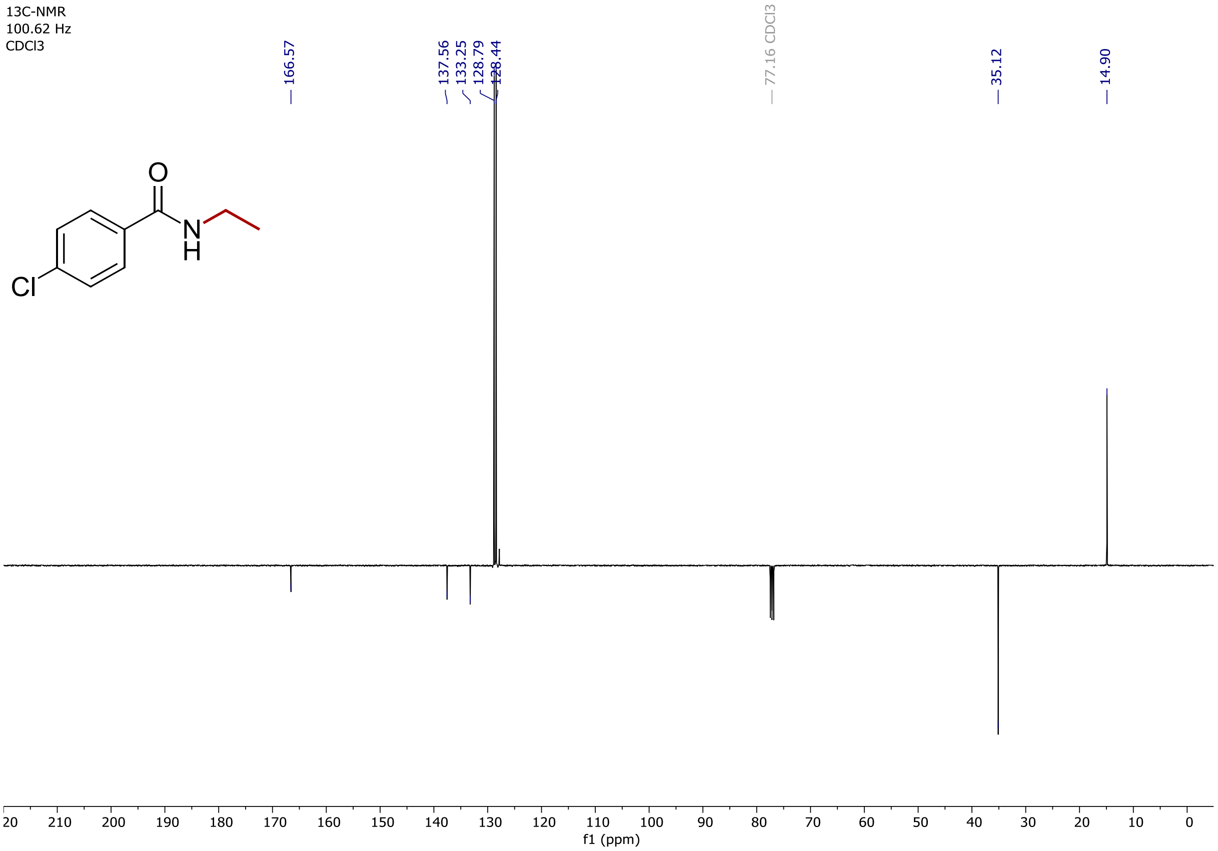

N-Ethylacetanilide (4h)

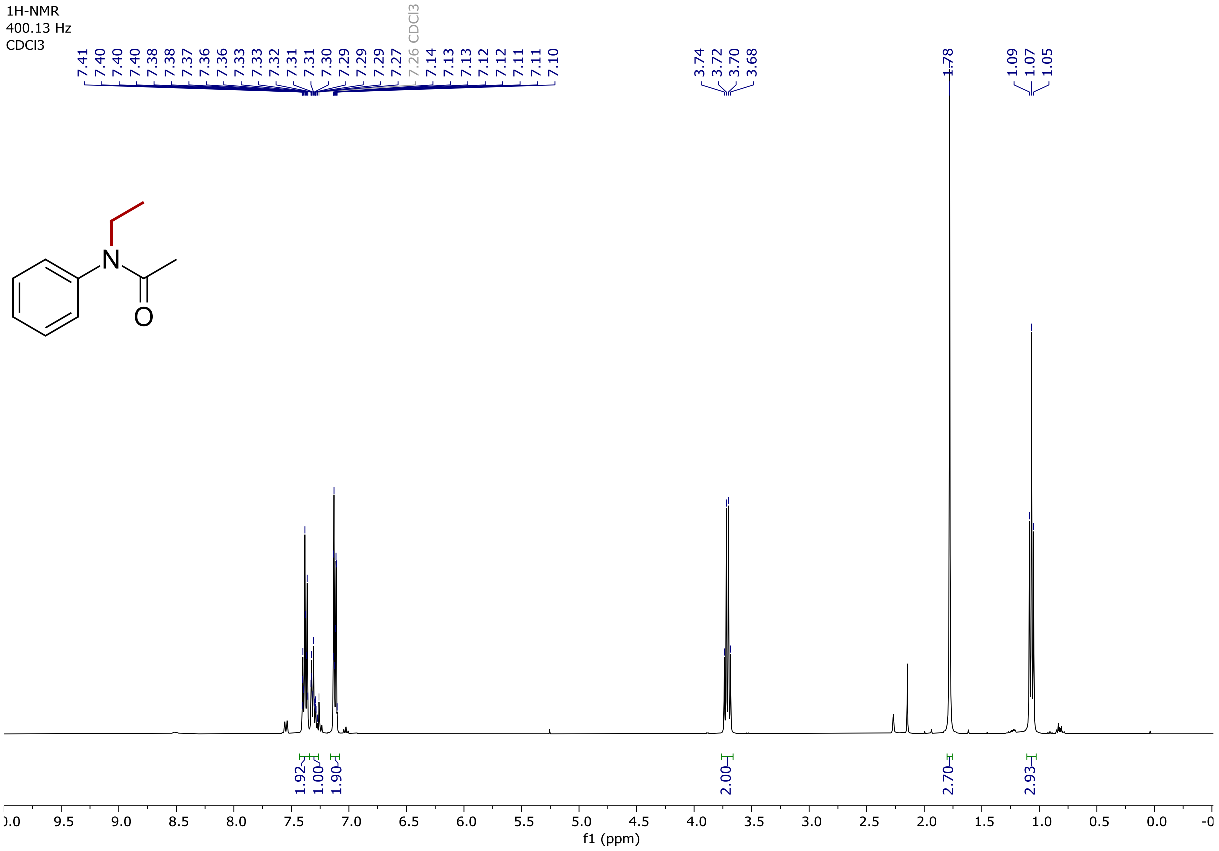

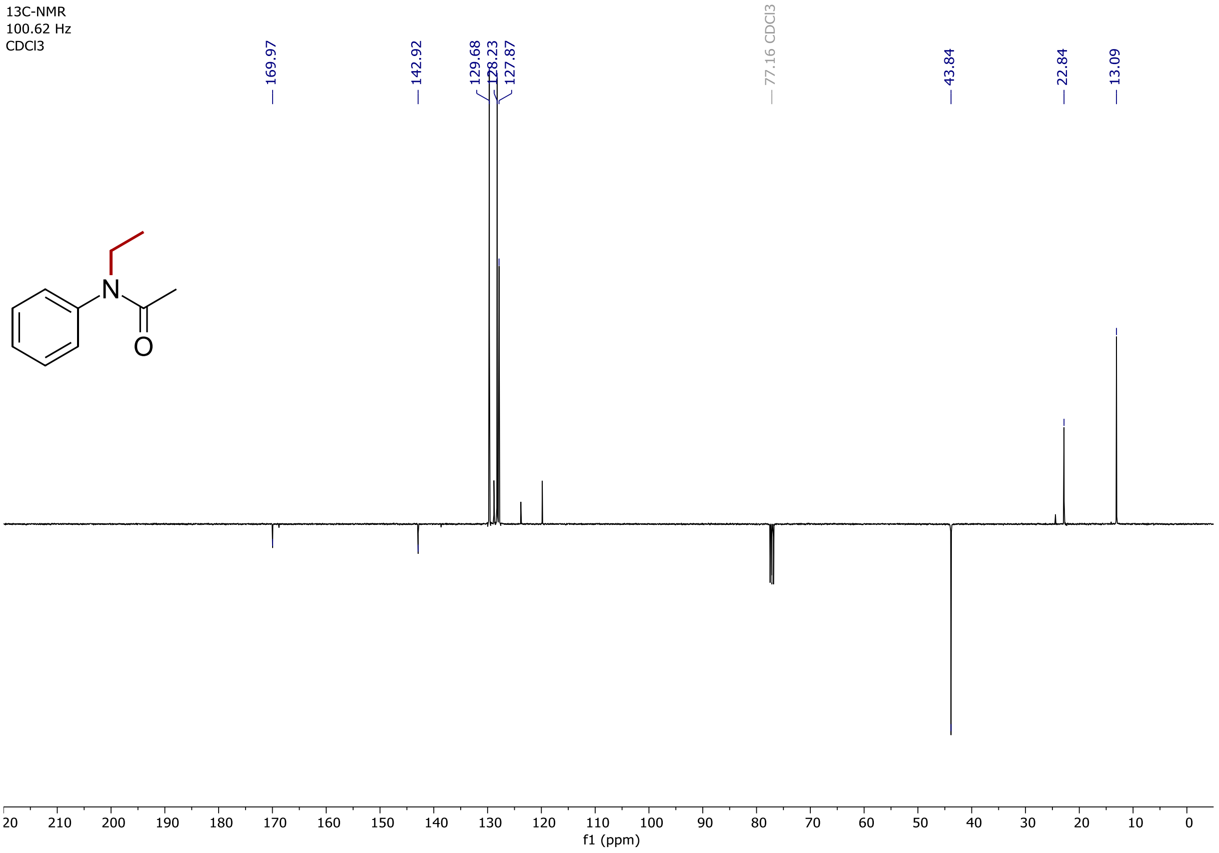

***N*-Ethylphenylacetamide (4i)**

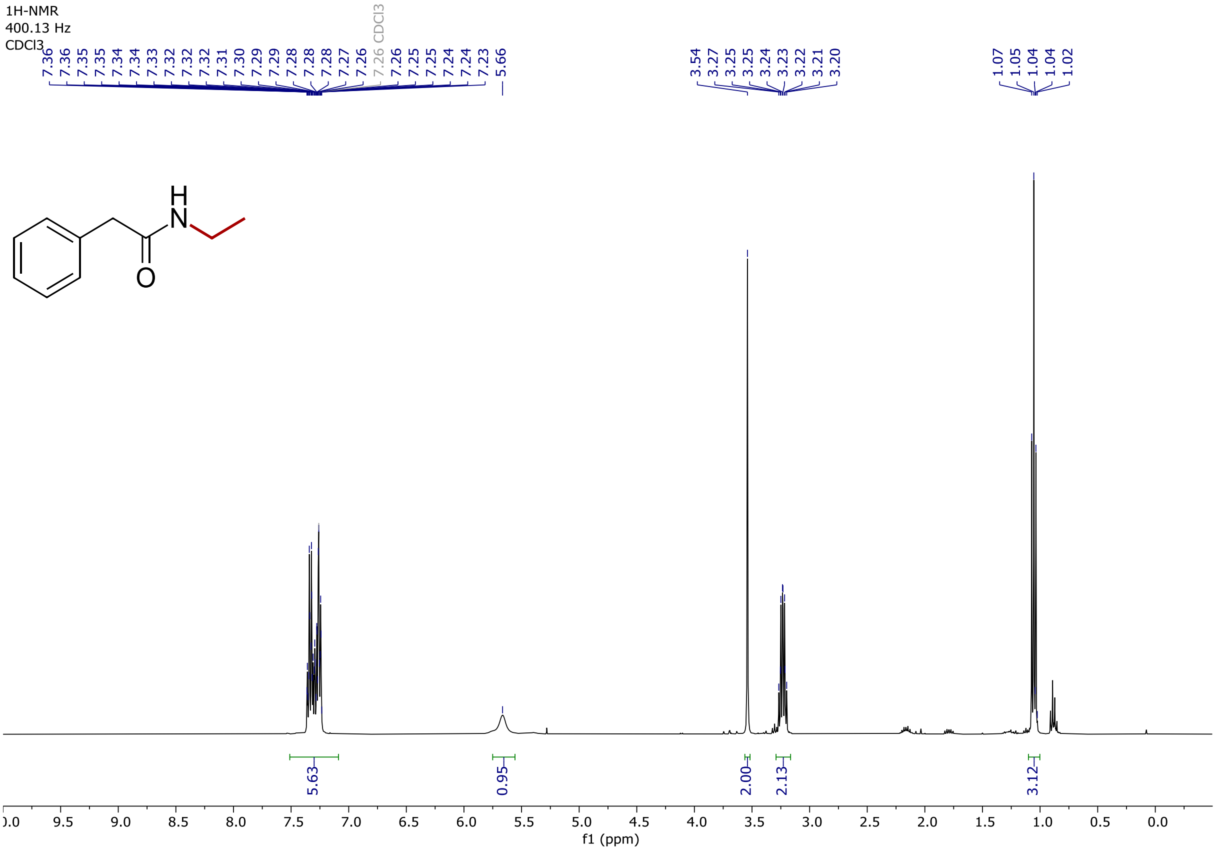

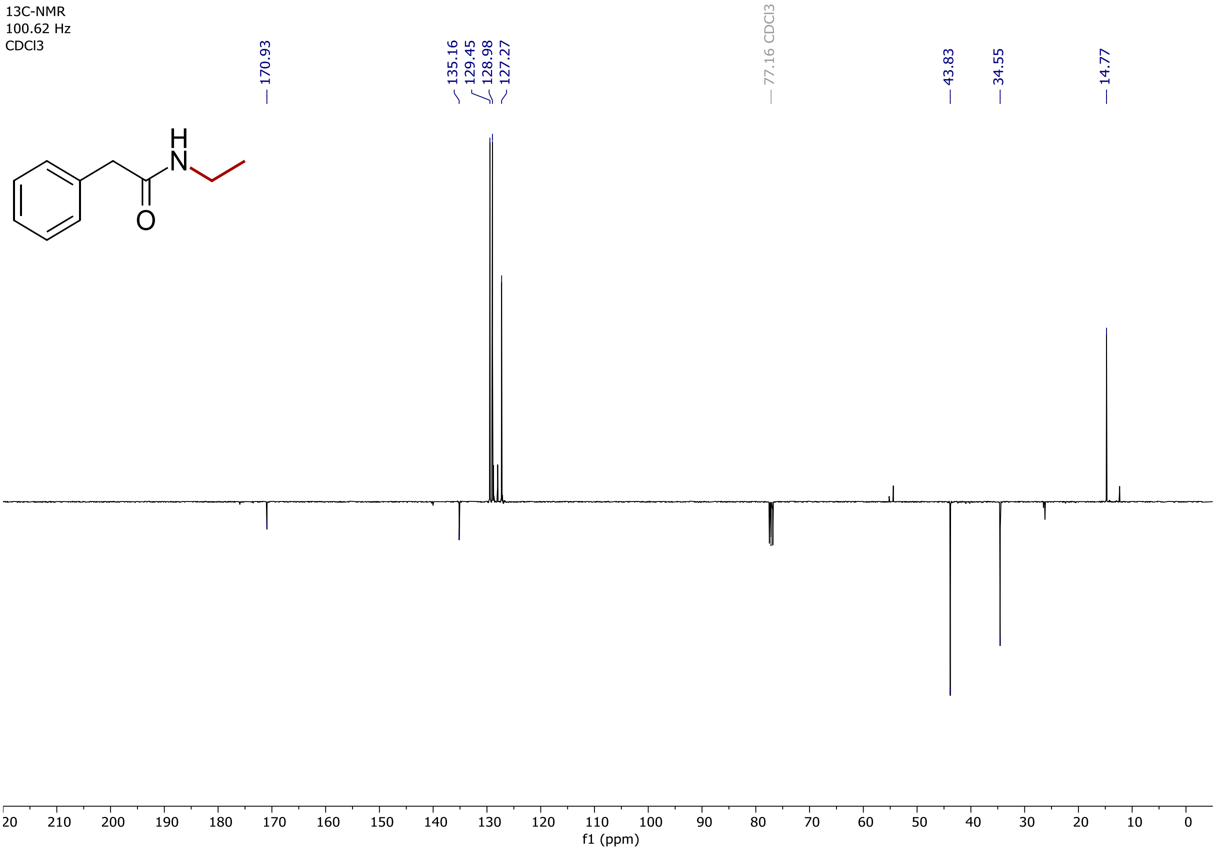

***N*-Ethyl-4-methoxybenzamide (4j)**

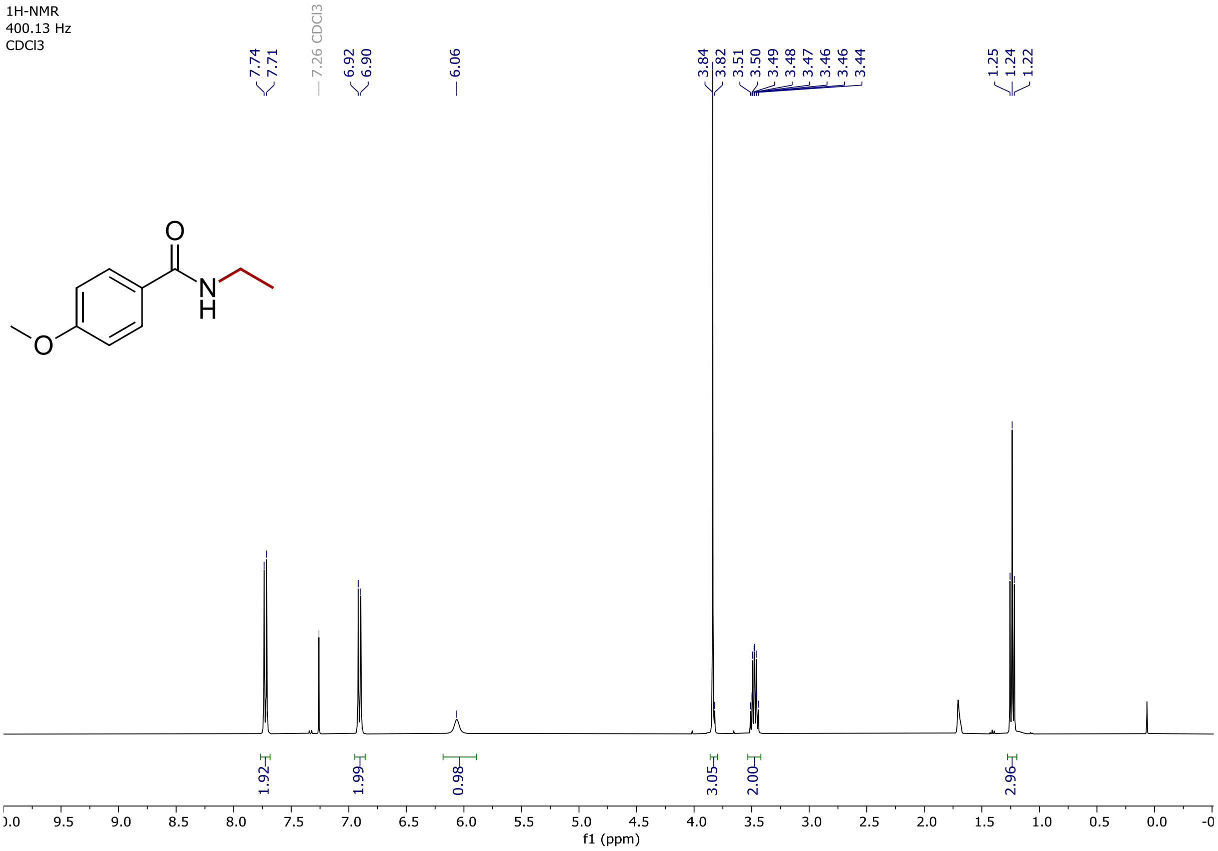

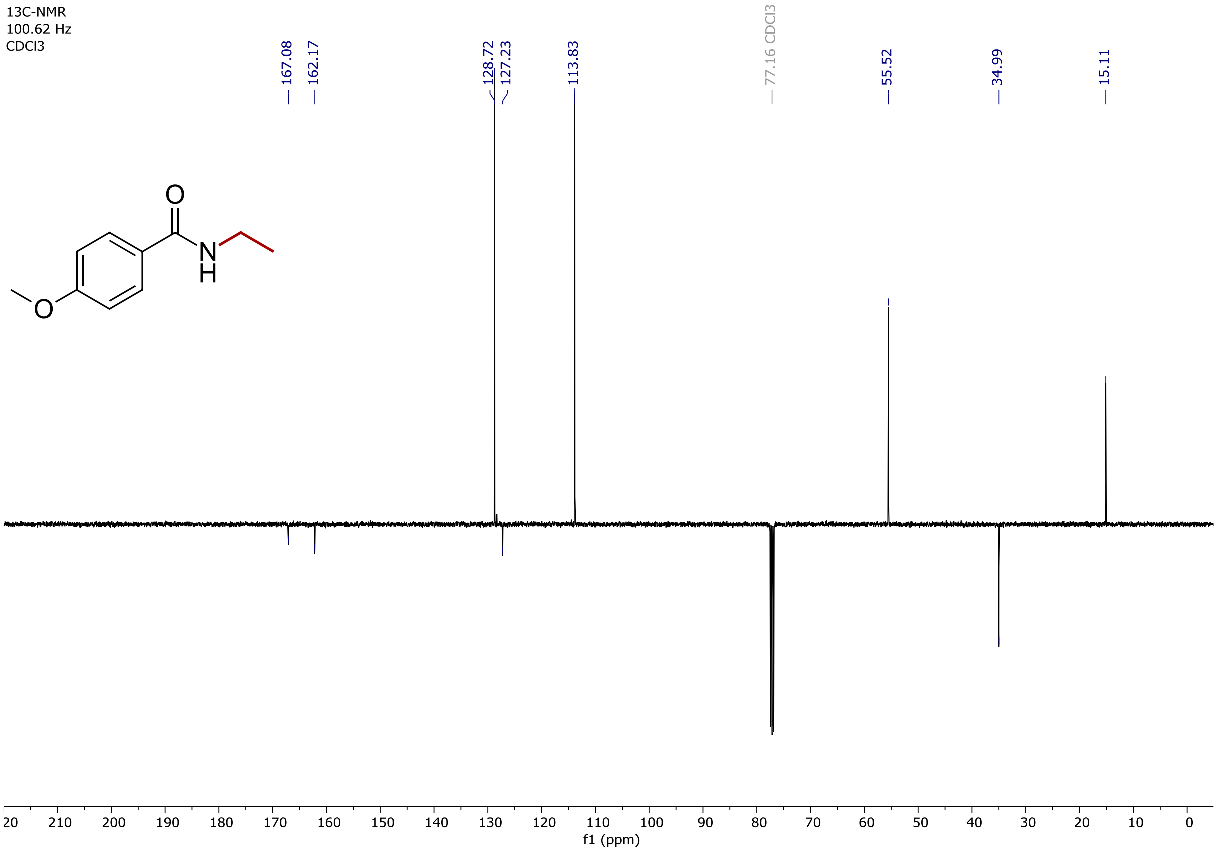

N-Ethyl-3,5-dimethoxbenzamide (4k)

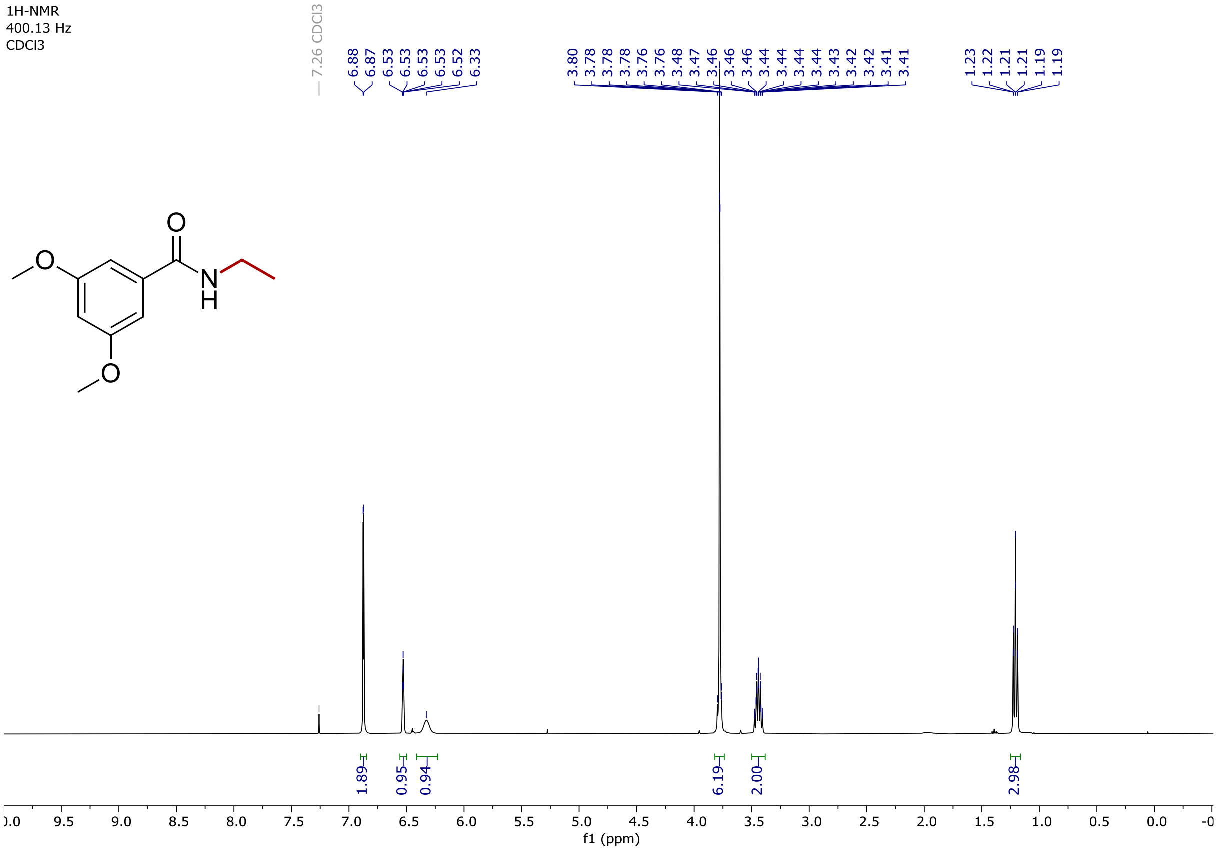

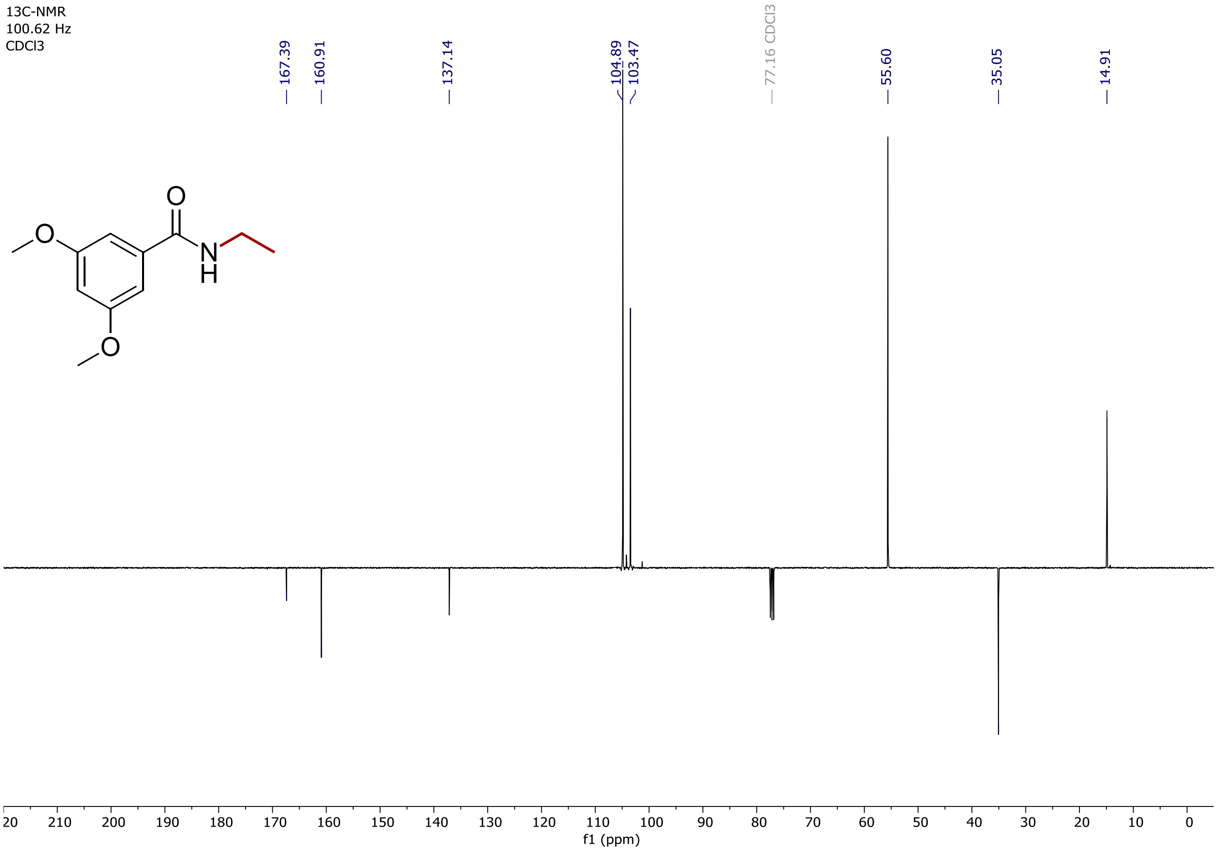

***N*-Ethyl-4-nitrobenzamide (4l)**

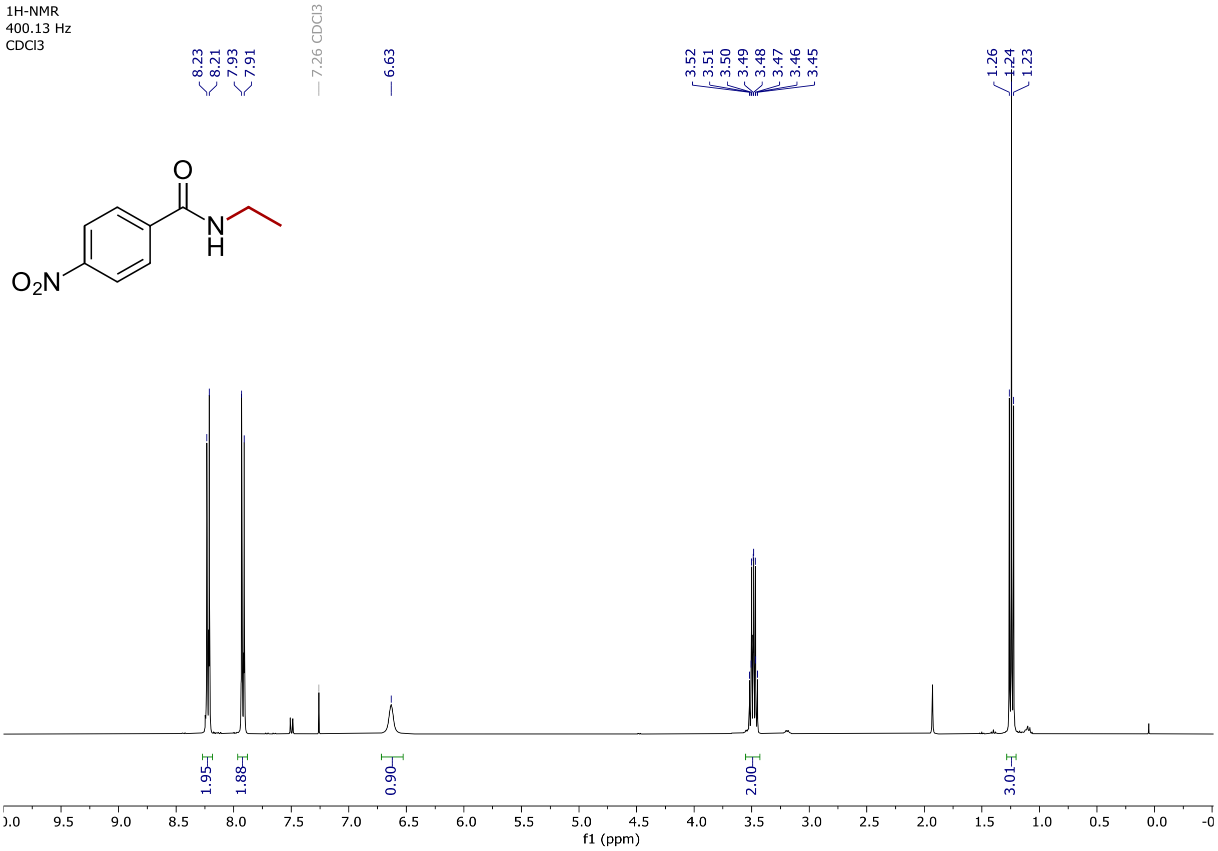

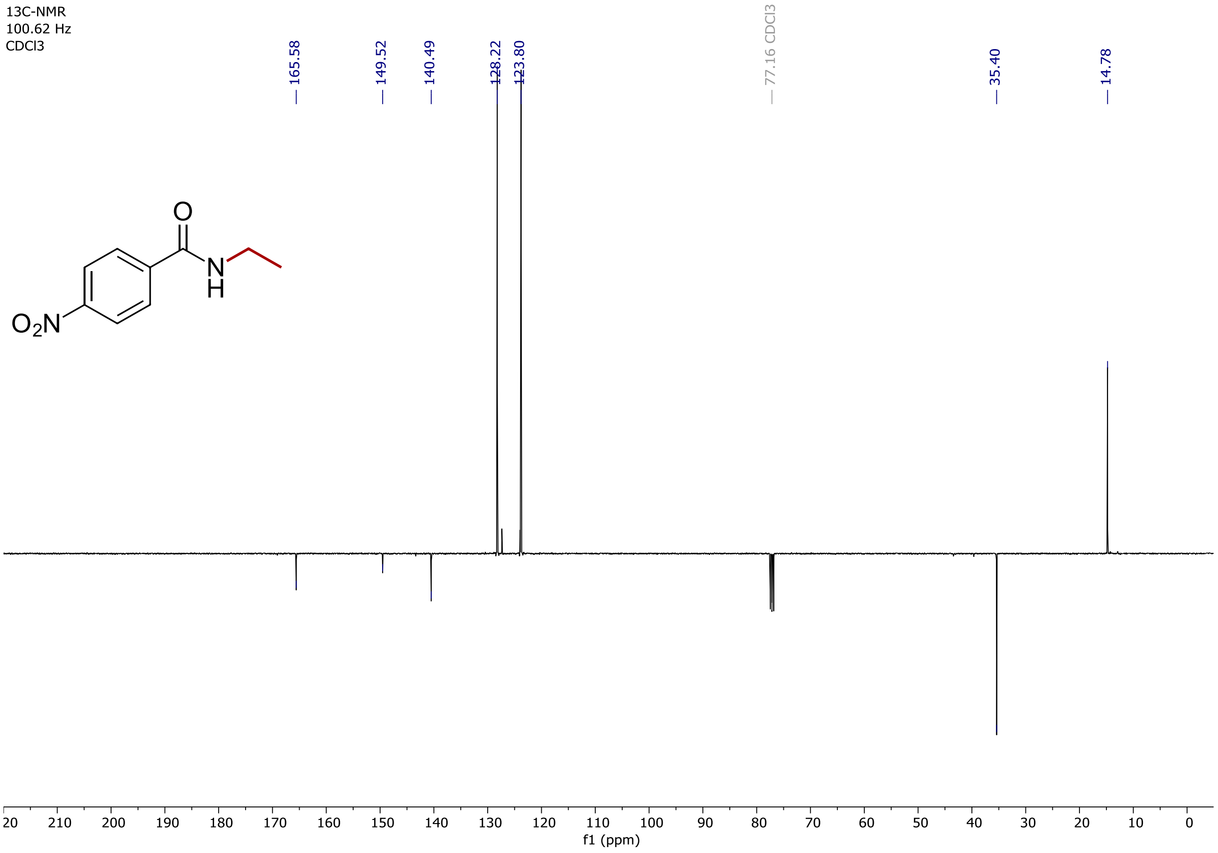

N-Ethyl-α-naphthylacetamide (4m)

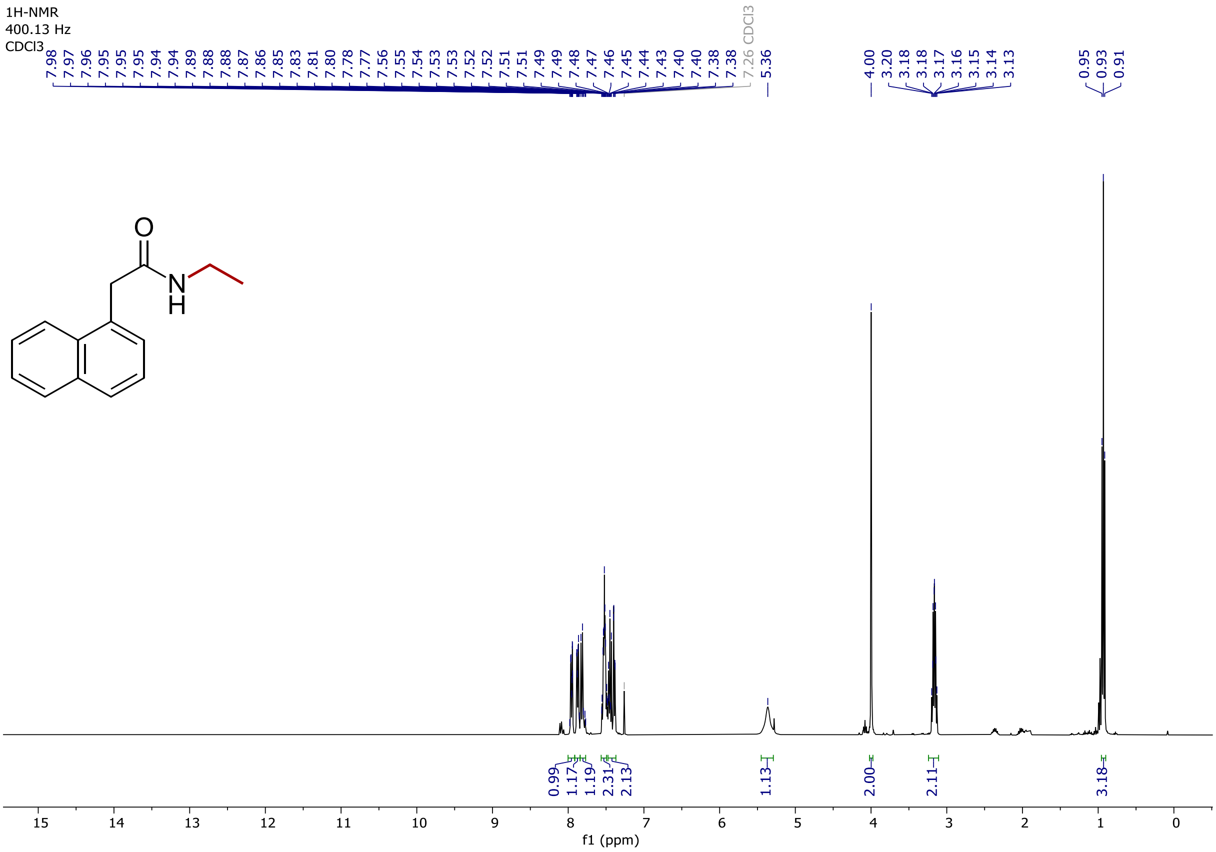

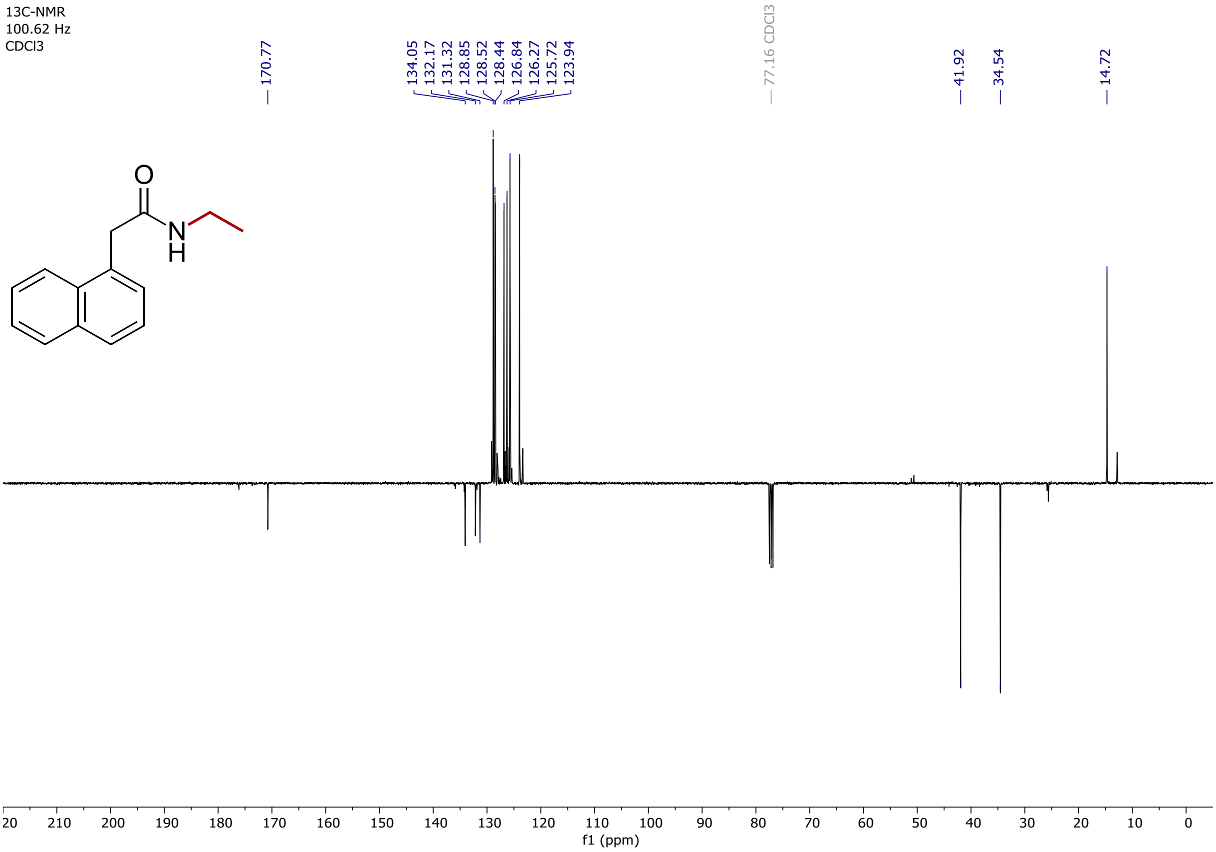

1-Ethylindole (6a)

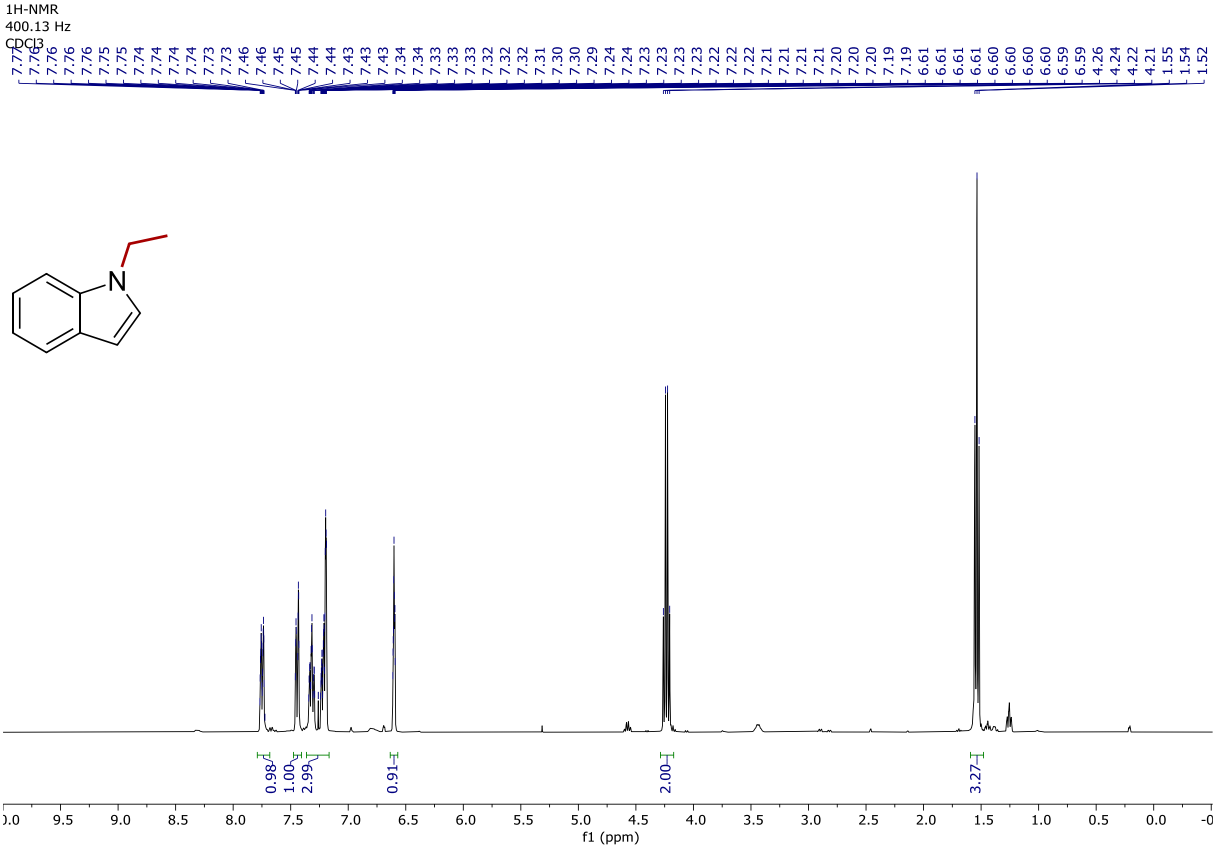

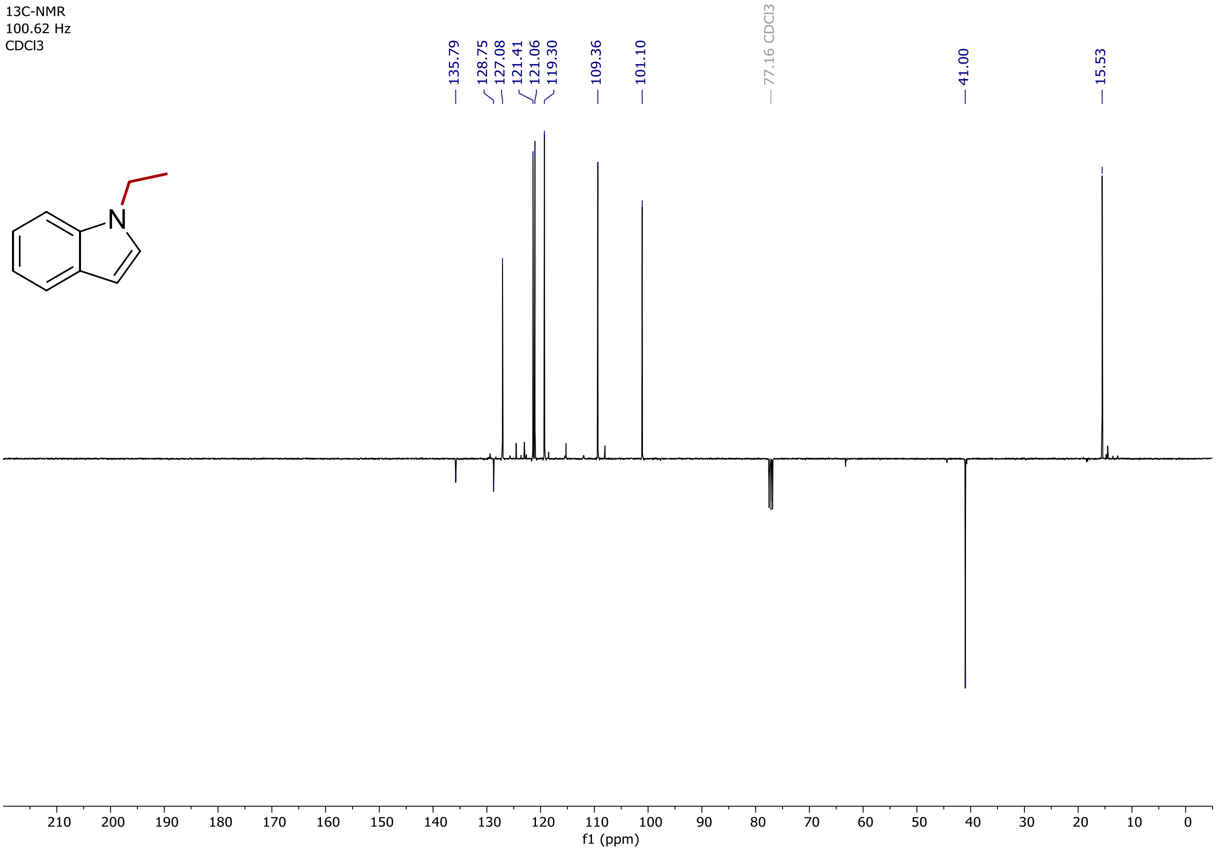

1-Ethyl-5-methoxyindole (6b)

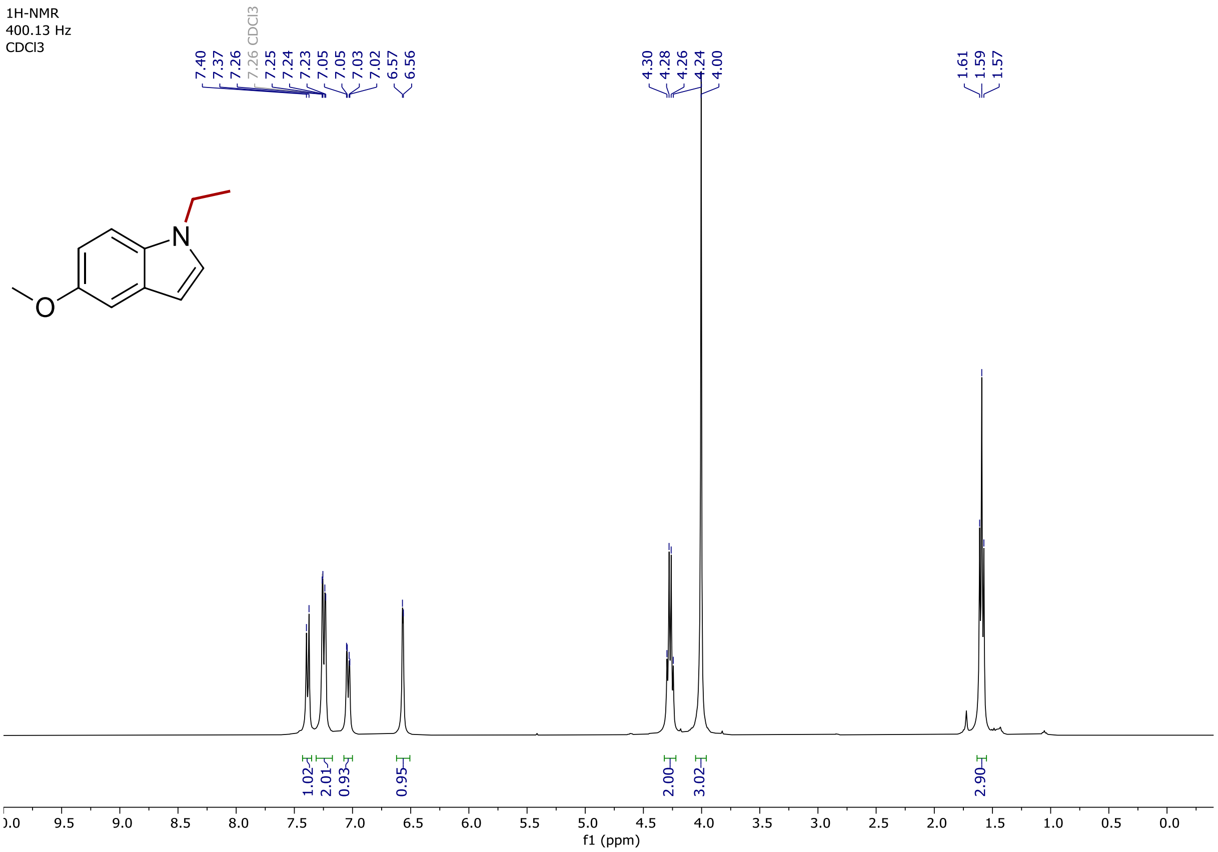

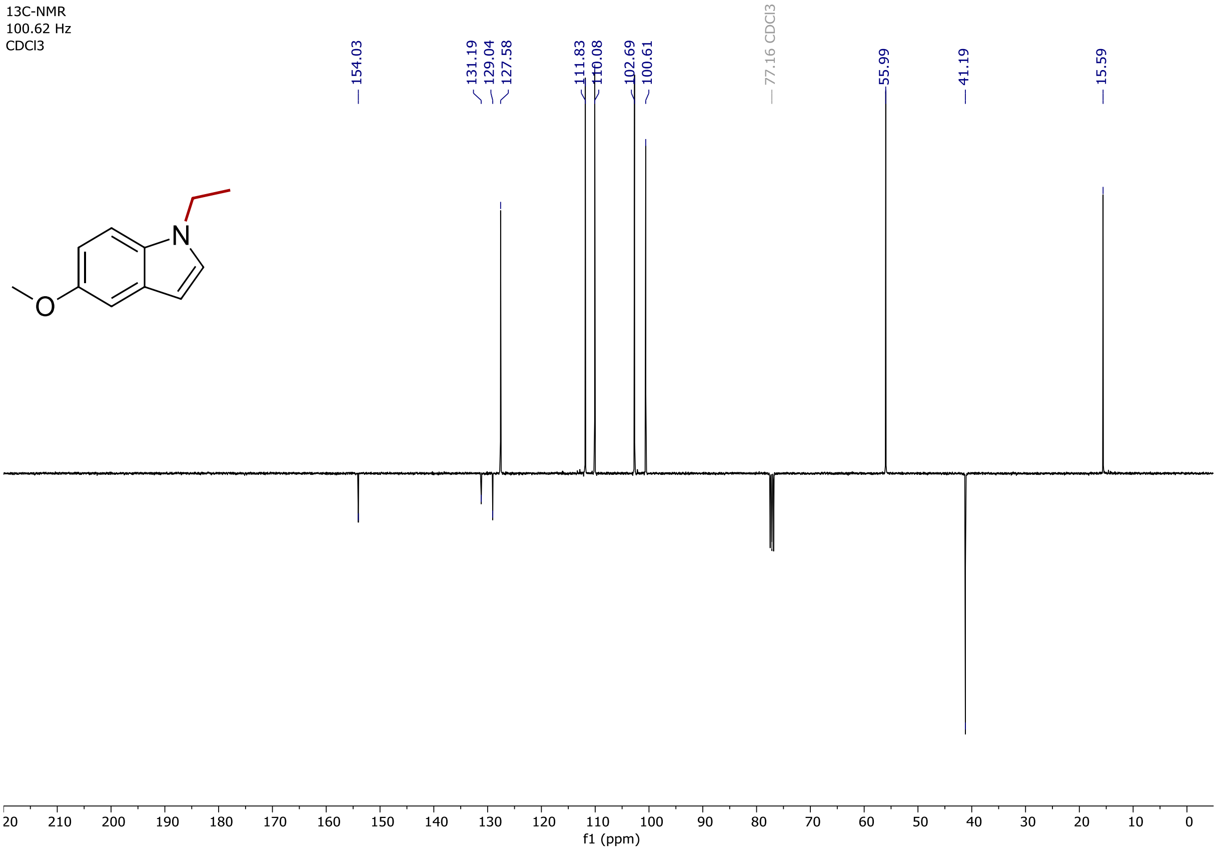

1-Ethyl-5-fluoroindole (6c)

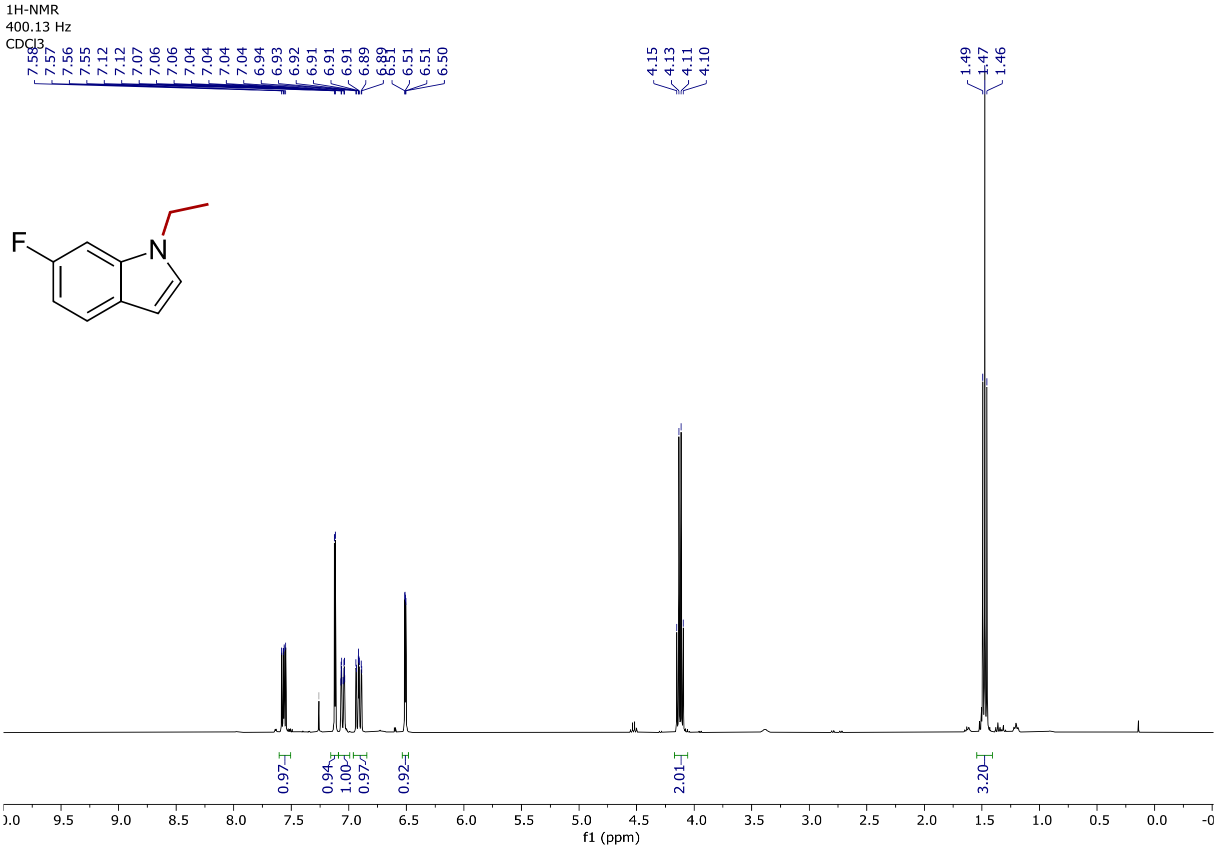

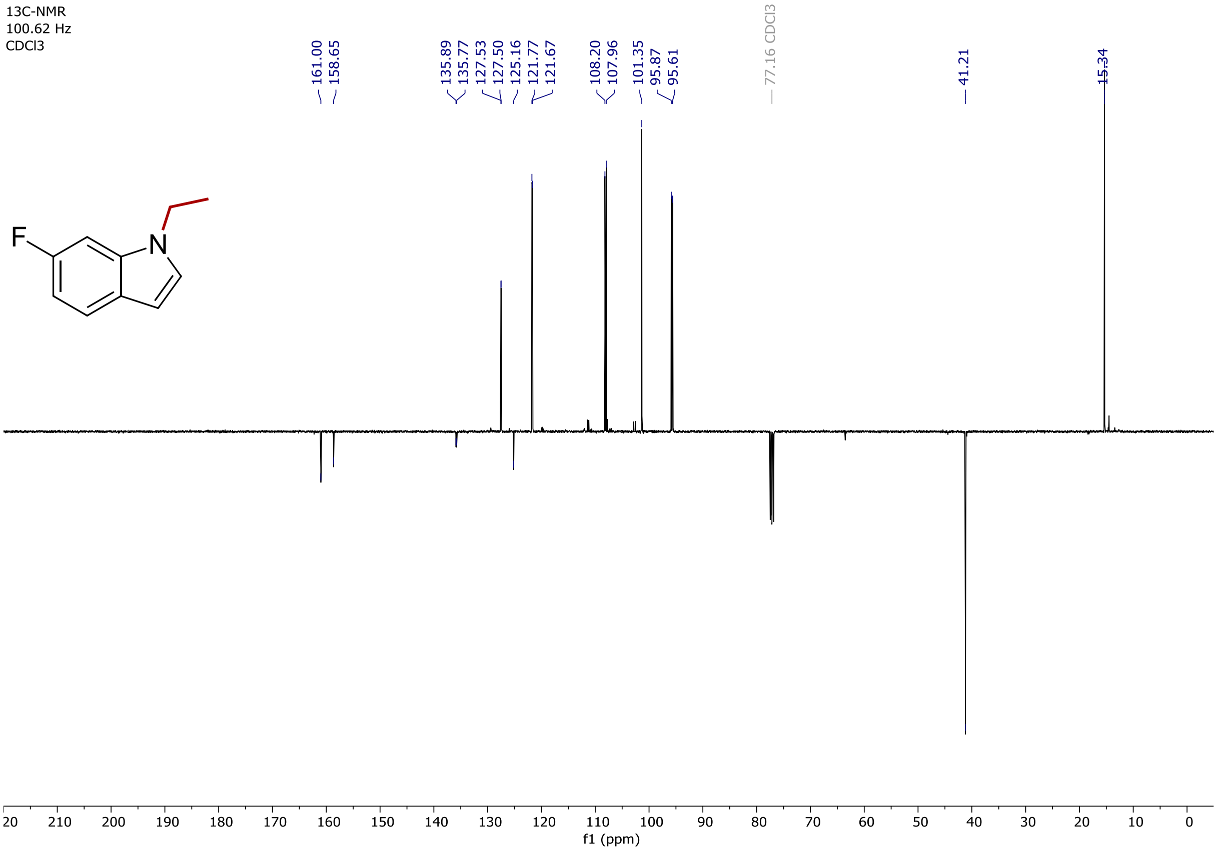

5-Chloro-1-ethylindole (6d)

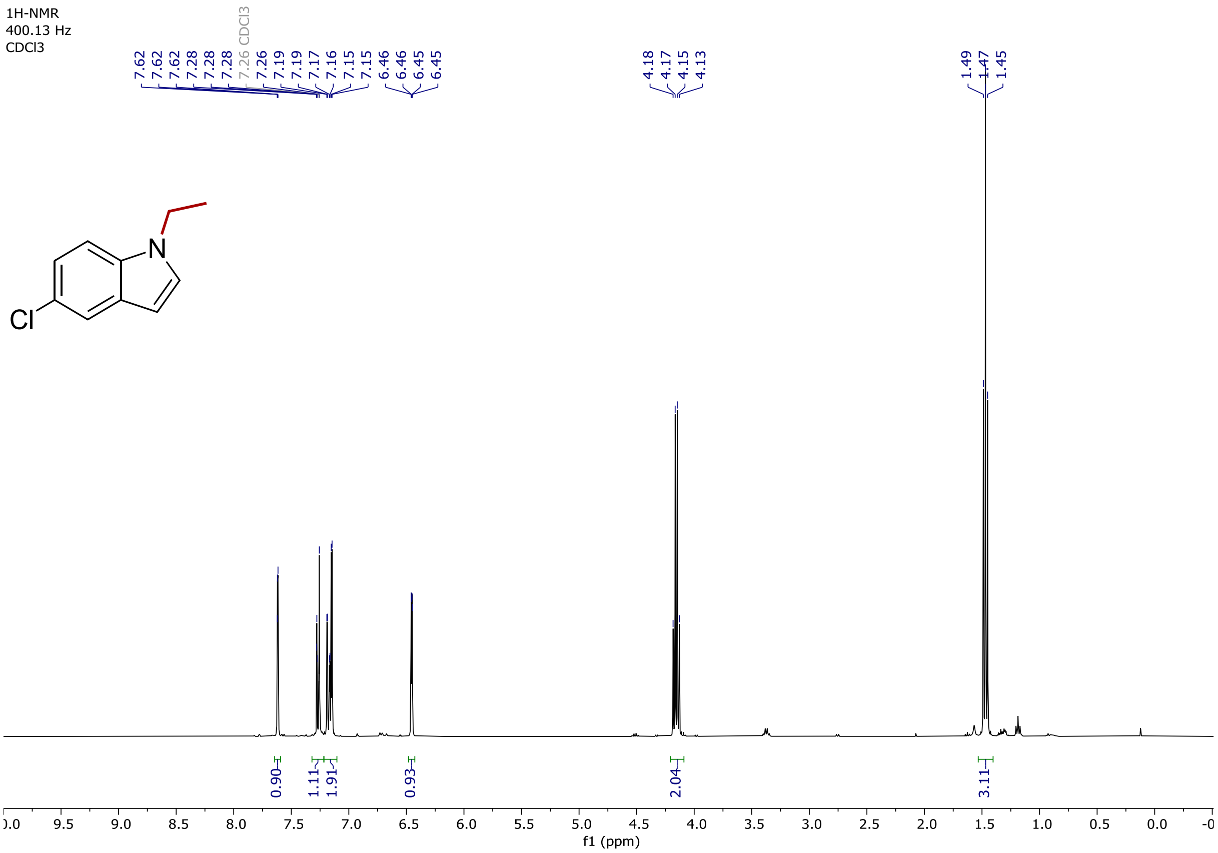

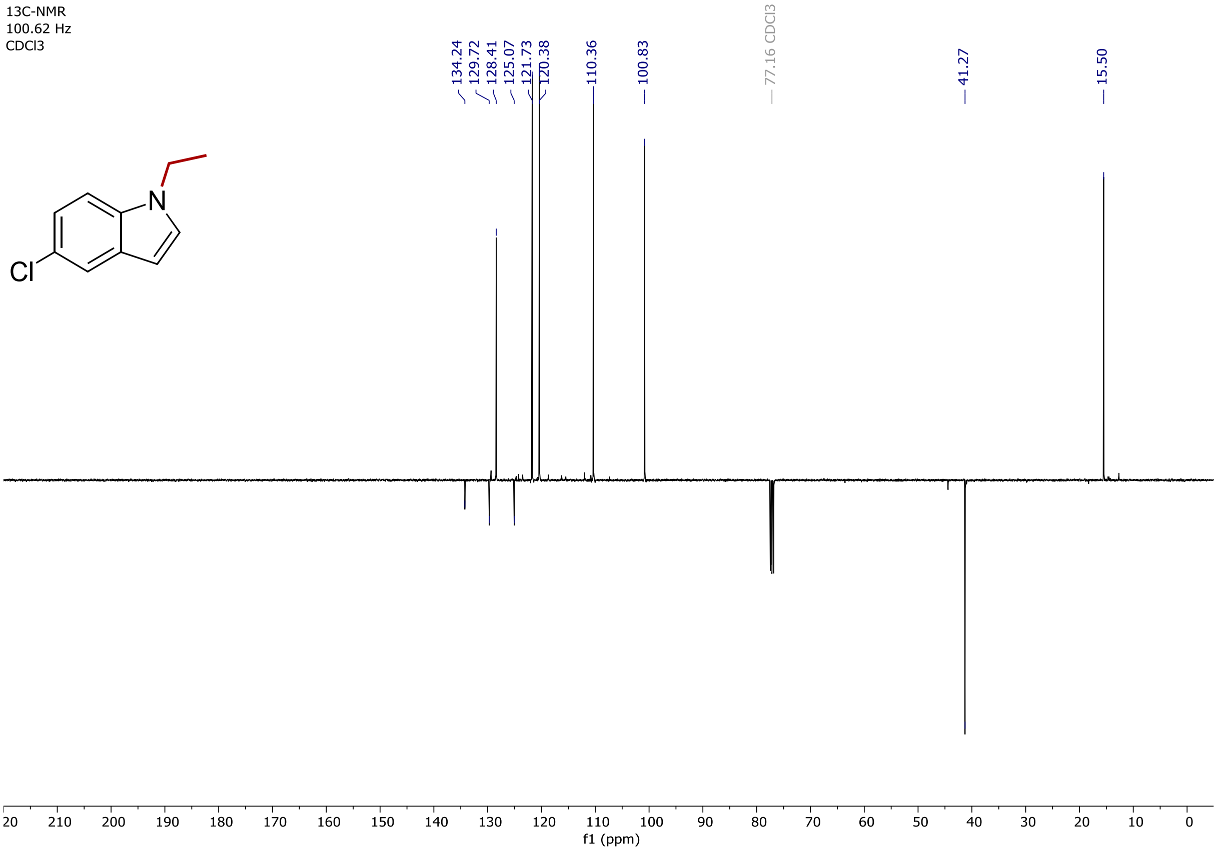

5-Bromo-1-ethylindole (6e)

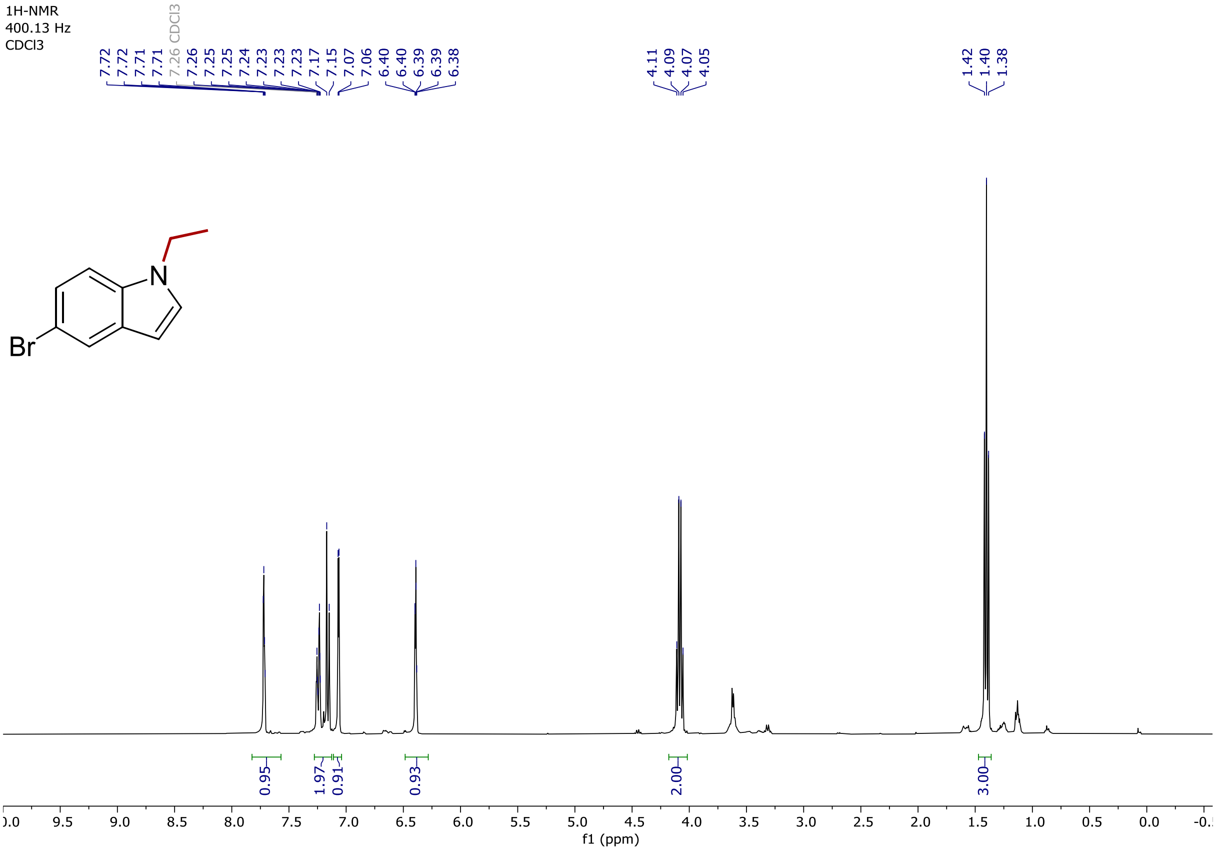

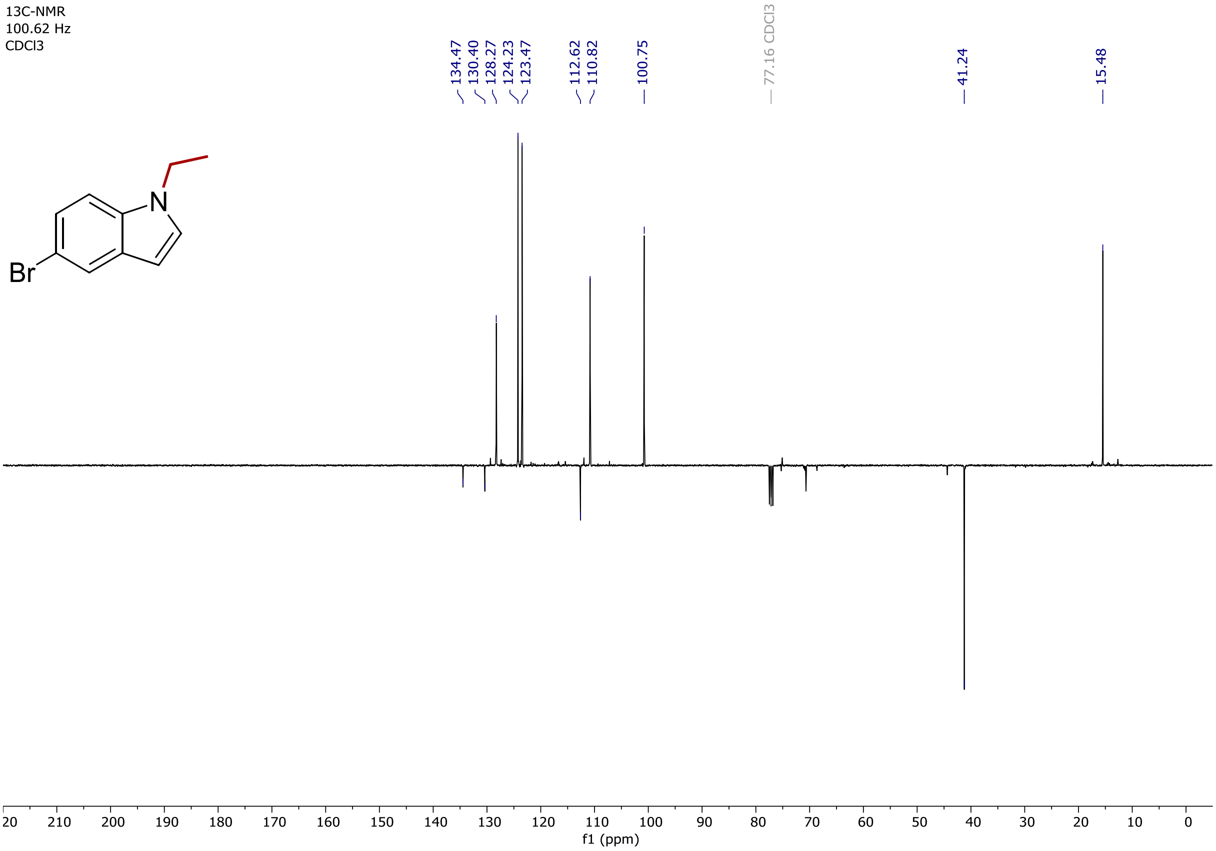

1-Ethyl-5-iodoindole (6f)

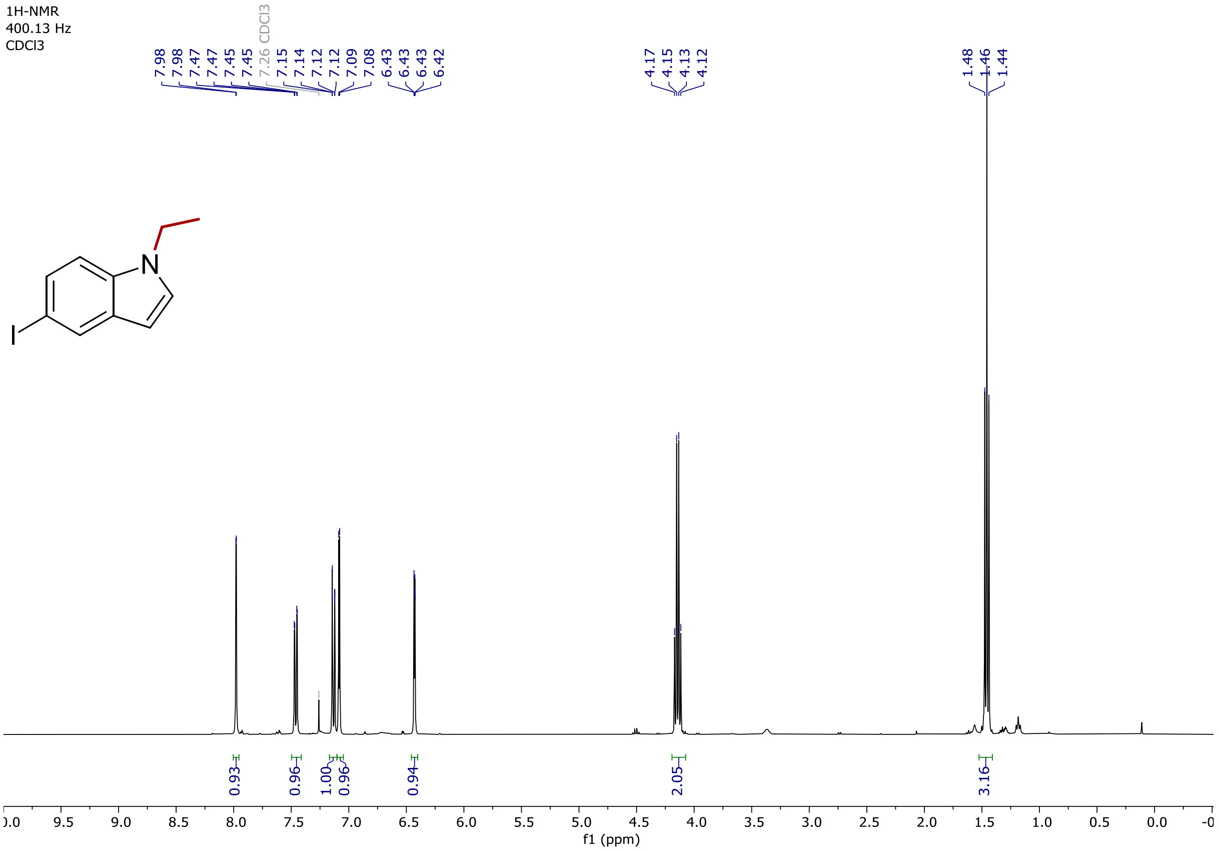

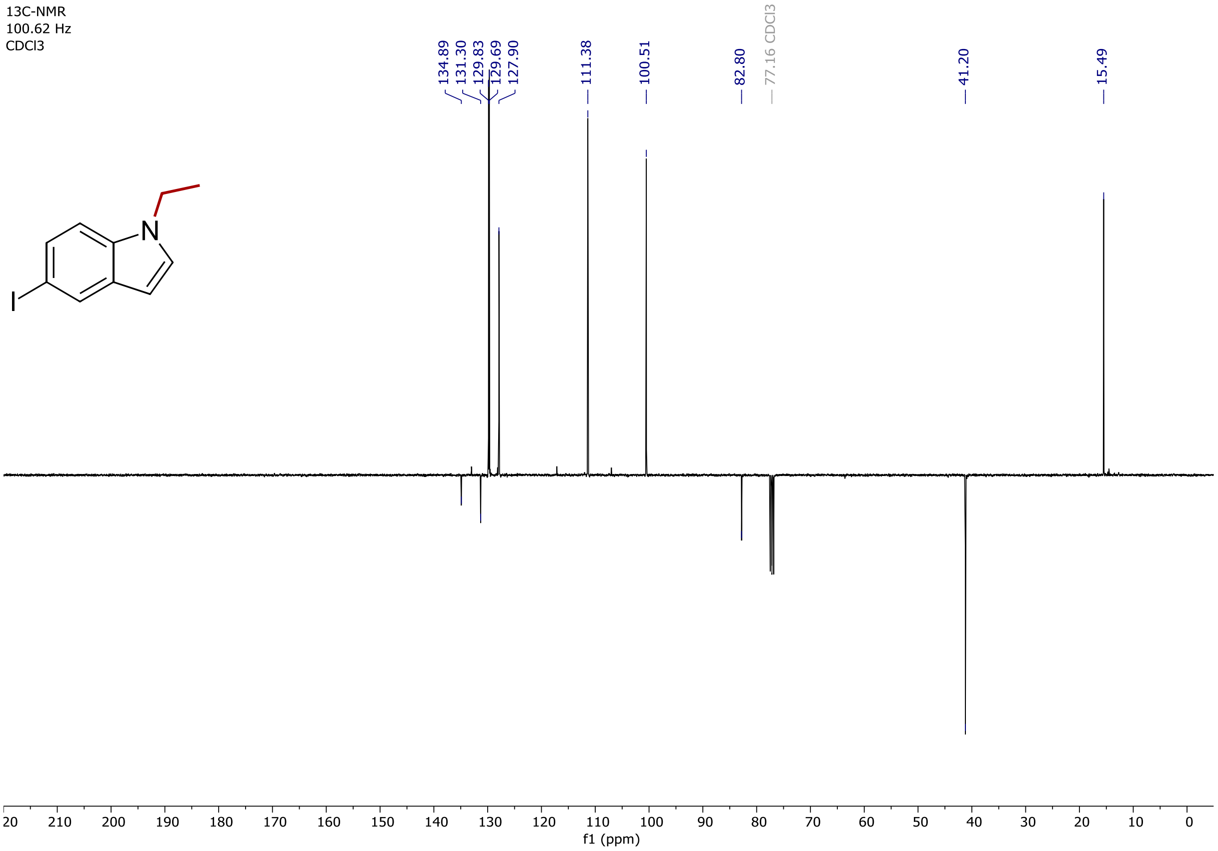

1-Ethyl-5-nitroindole (6g)

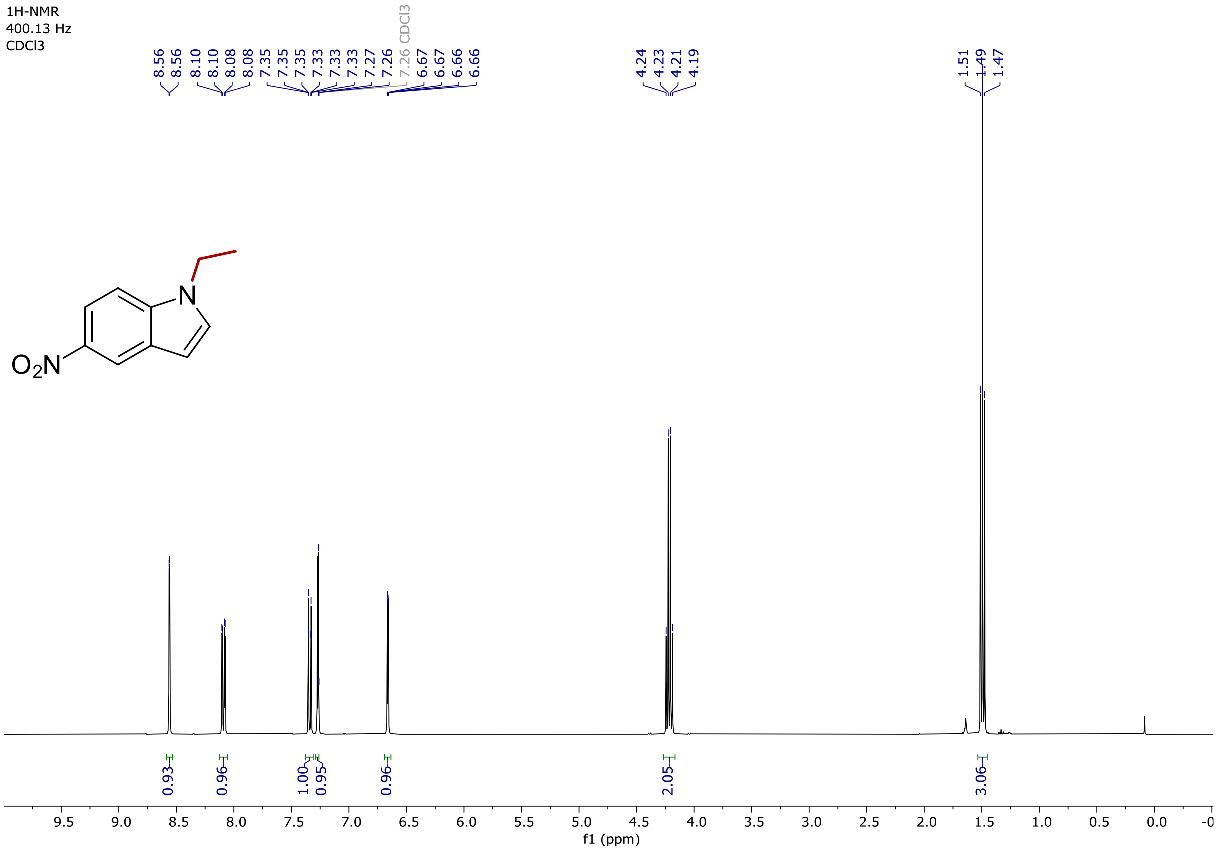

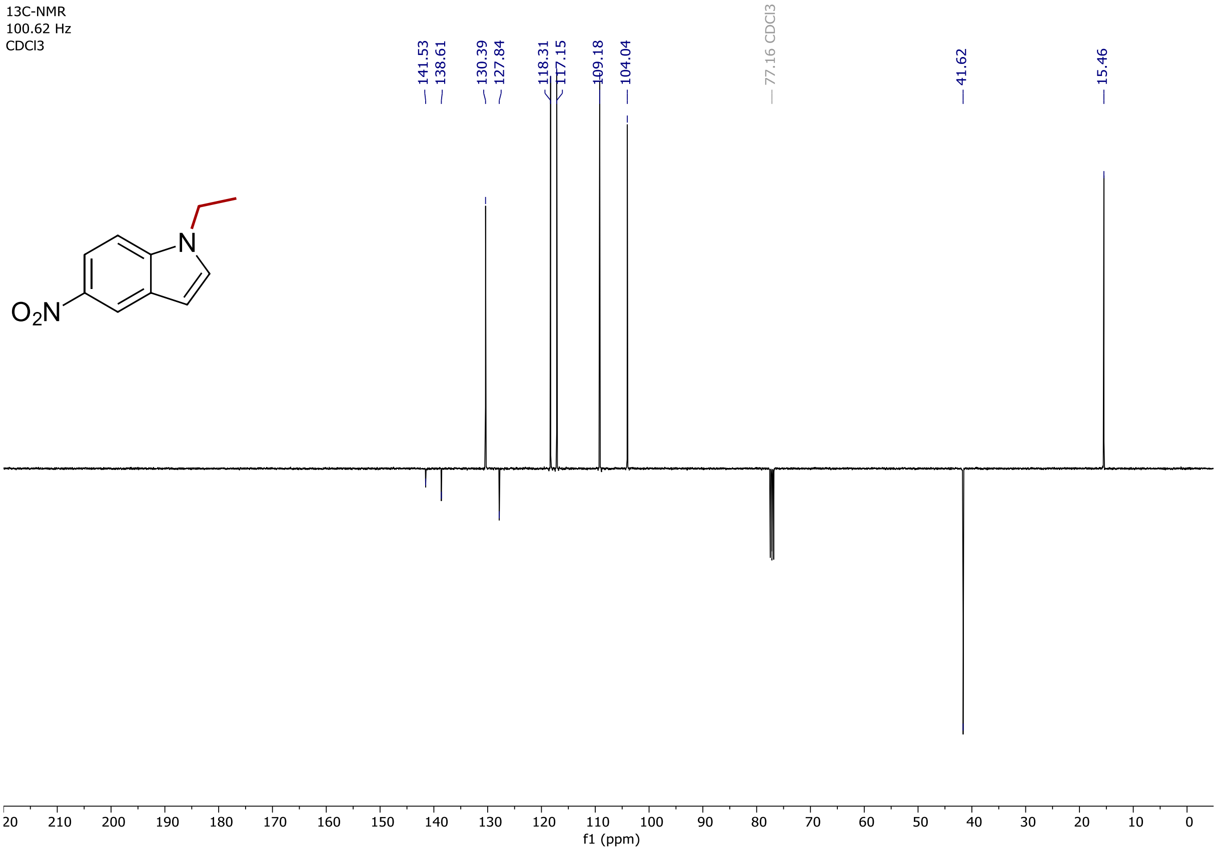

1-Ethyl-2-indolecarbaldehyde (6h)

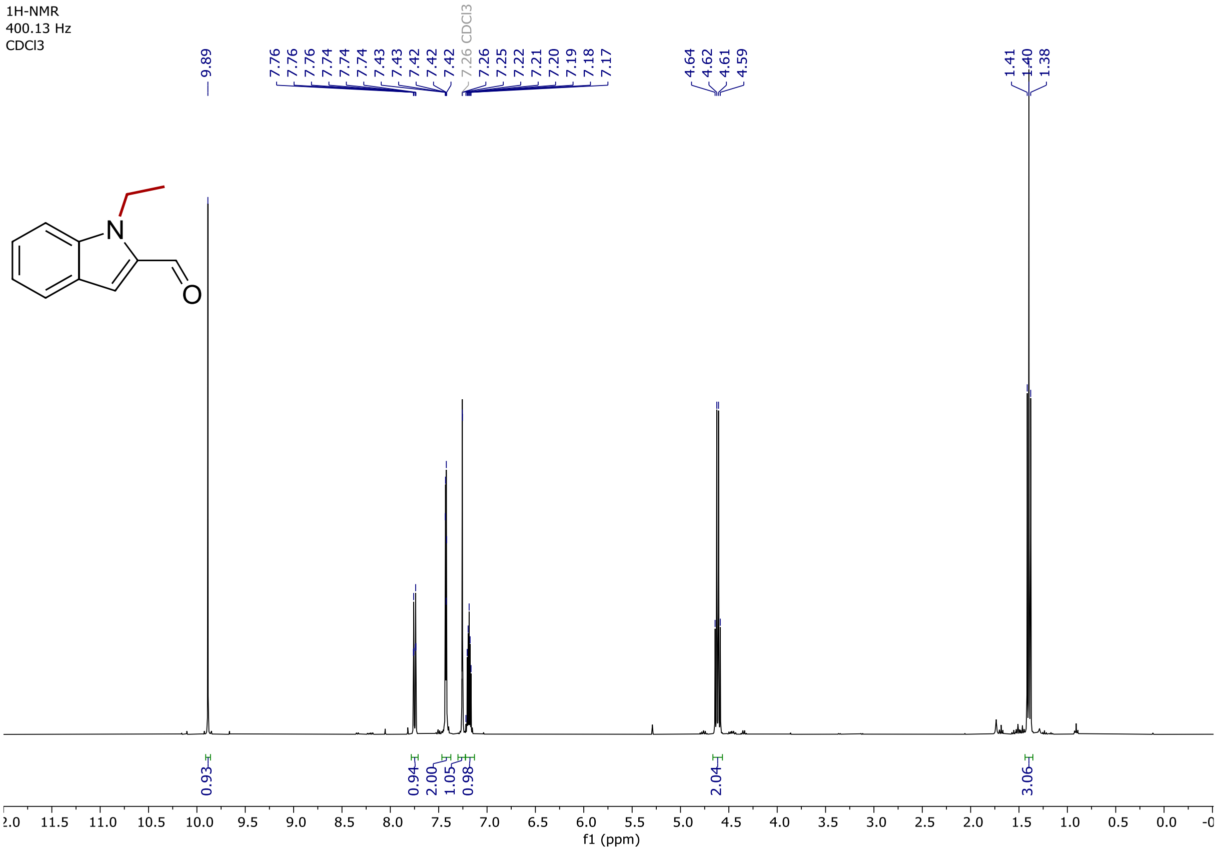

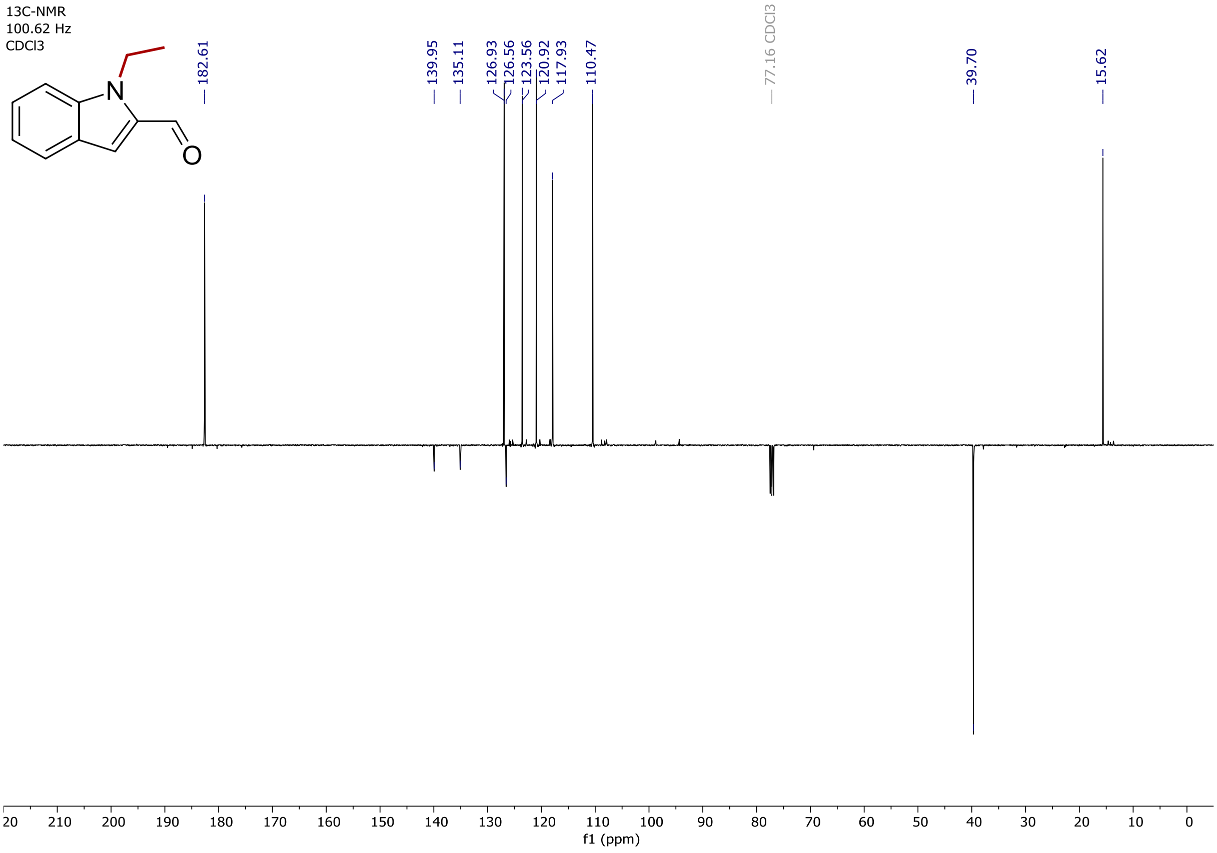

Ethyl 1-ethyl-2-indolecarboxylate (6i)

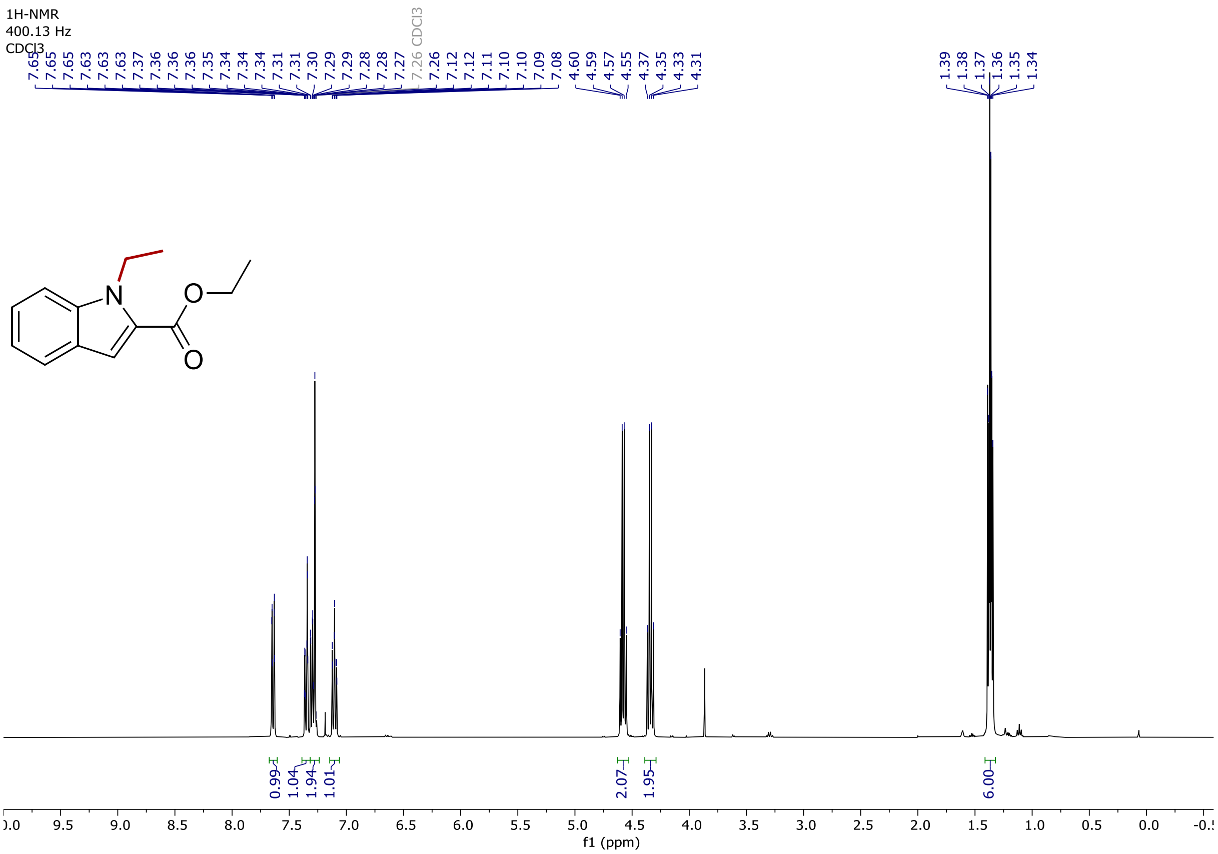

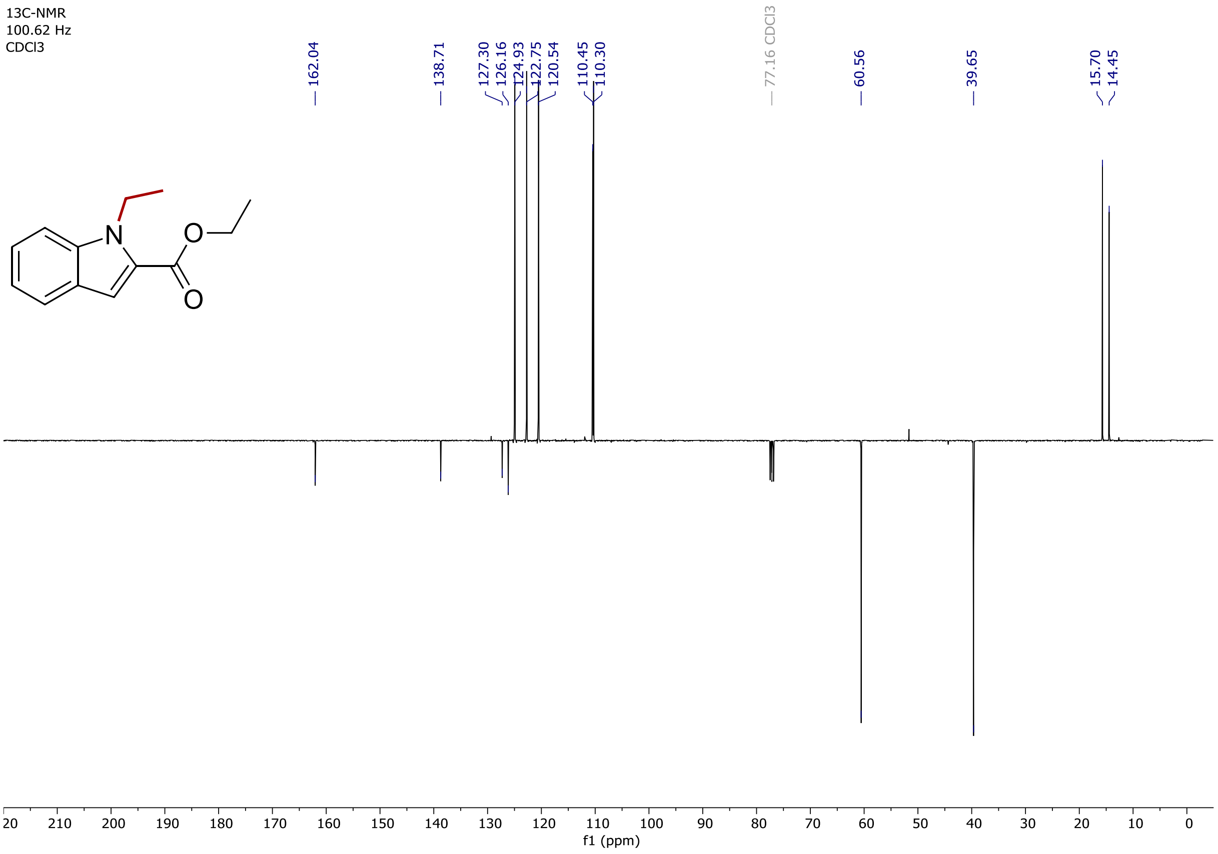

Methyl 1-ethyl-5-indolecarboxylate (6j)

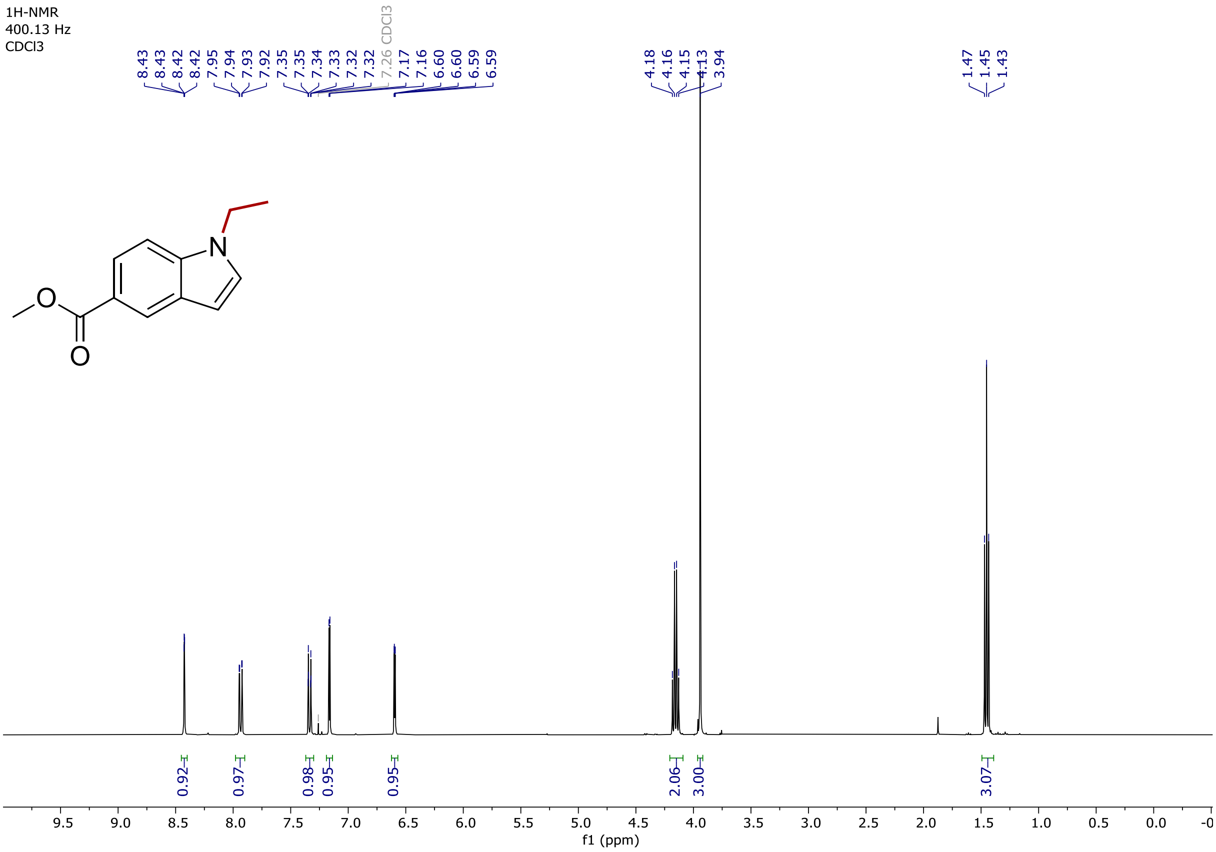

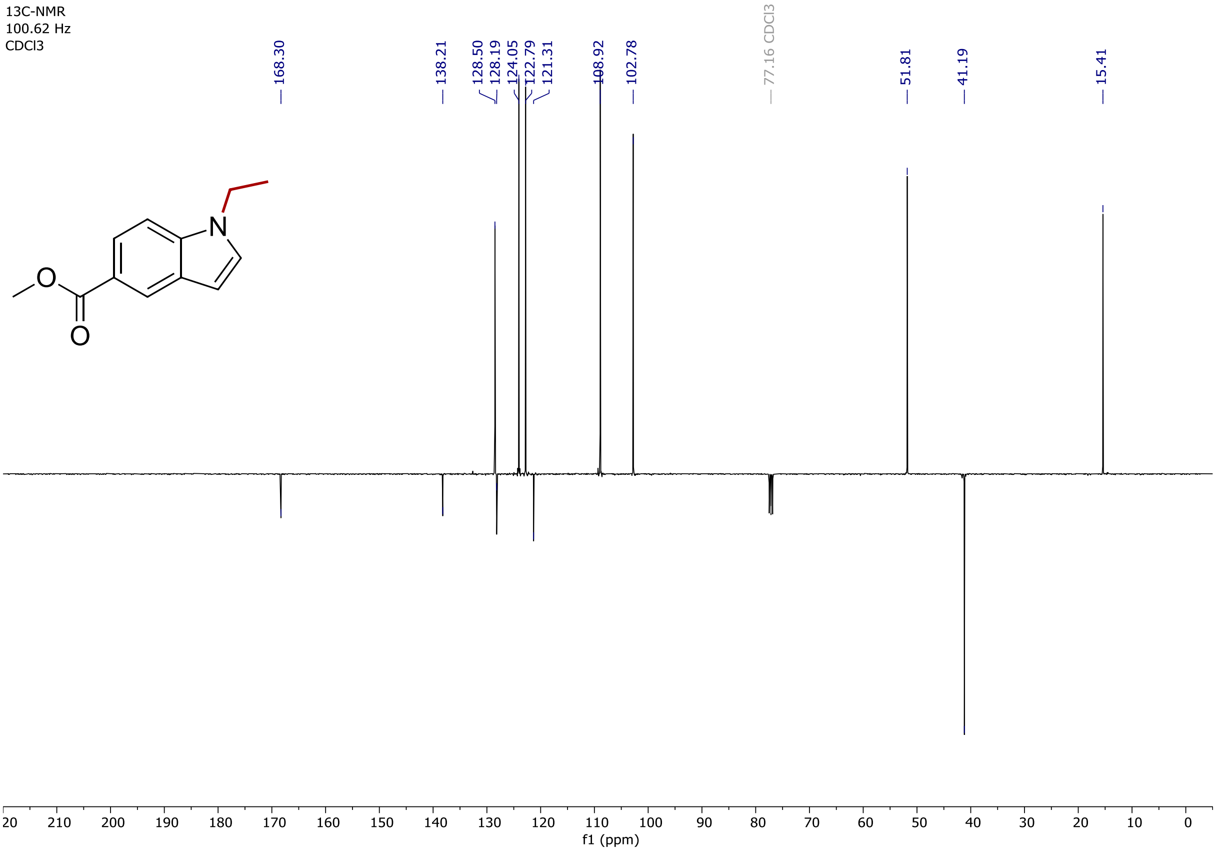

(1-Ethyl-3-indolyl)acetonitrile (6k)

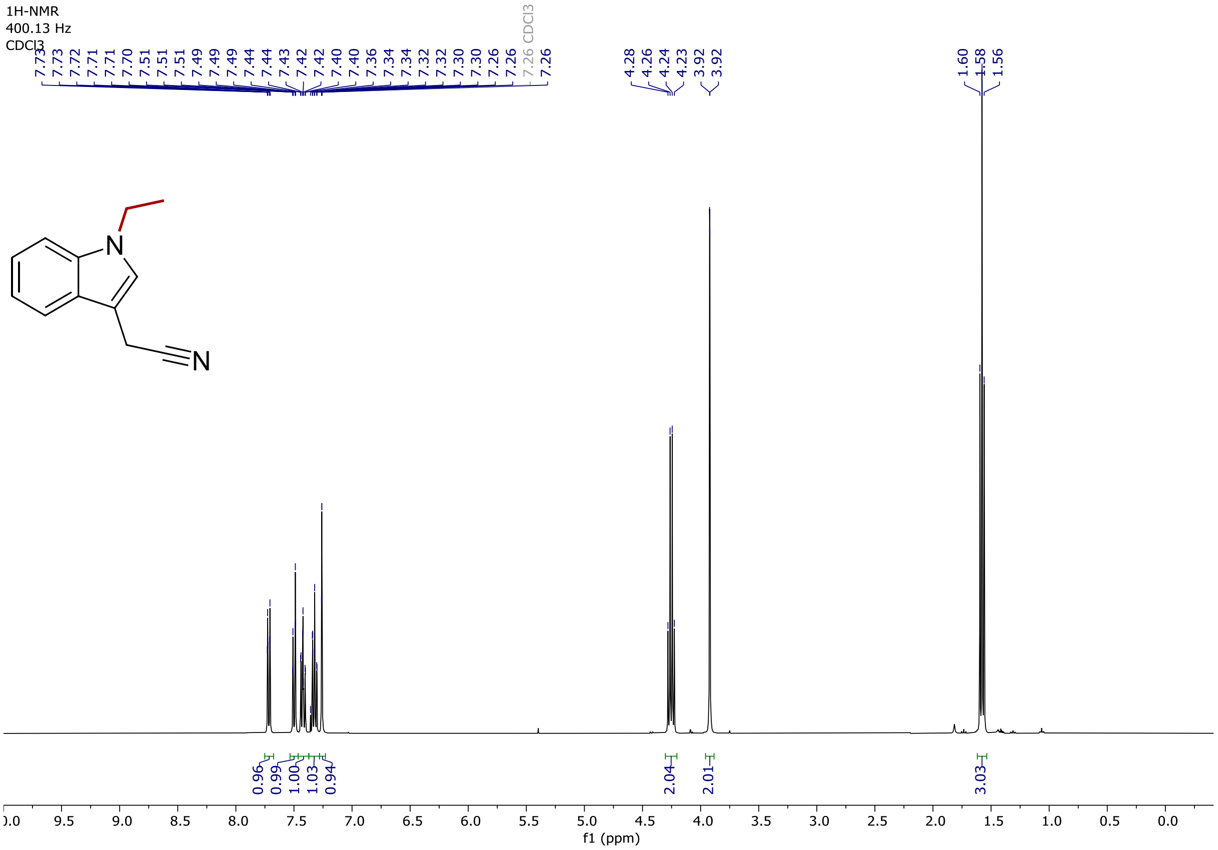

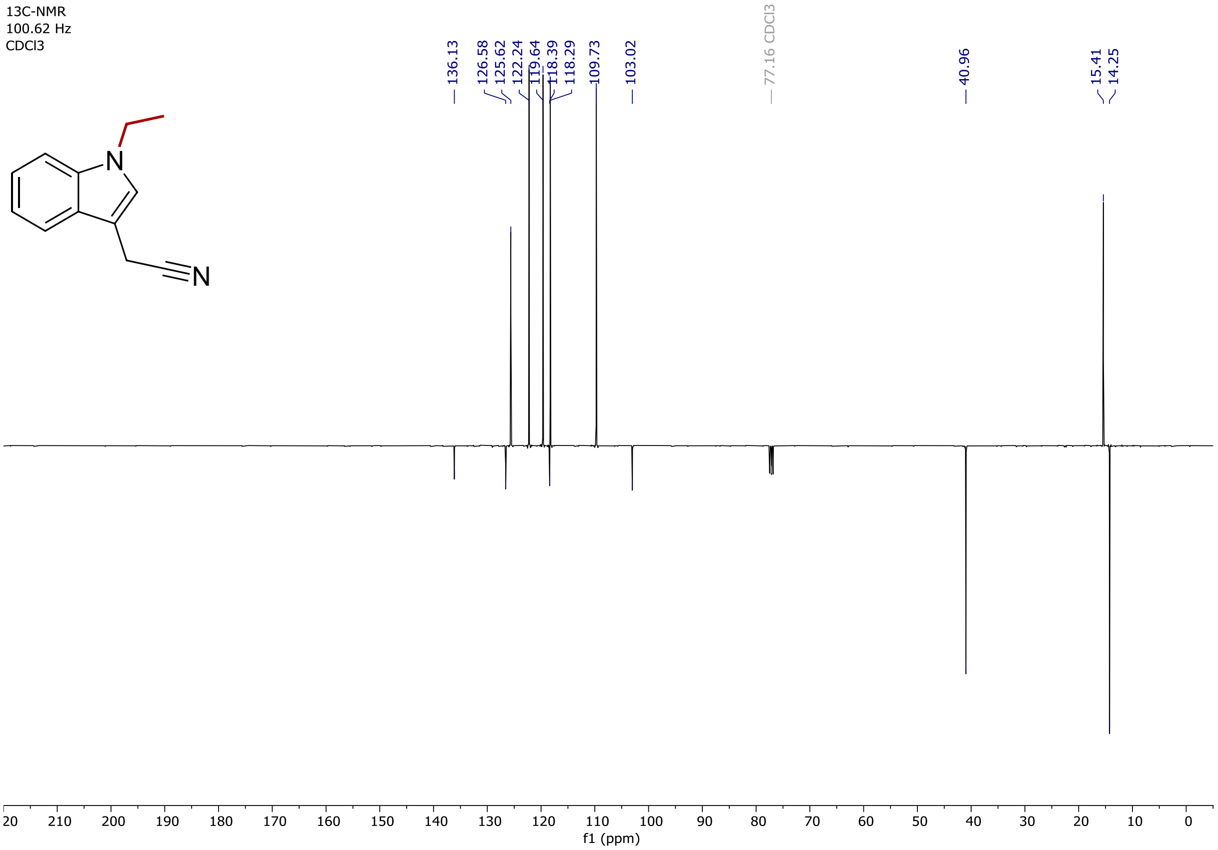

References

1. Mattes, A. O.; Russell, D.; Tishchenko, E.; Liu, Y.; Cichewicz, R. H.; Robinson, S. J., Application of 19F quantitative NMR to pharmaceutical analysis. *Concepts in Magnetic Resonance Part A* **2016,** *45*, e21422.
2. Nageswara Rao, S.; Reddy, N. N. K.; Samanta, S.; Adimurthy, S., I2-Catalyzed Oxidative Amidation of Benzylamines and Benzyl Cyanides under Mild Conditions. *The Journal of Organic Chemistry* **2017,** *82*, 13632-13642.
3. Sun, X.; Zhao, W.; Li, B.-J., Iridium-catalyzed, ligand-controlled directed alkynylation and alkenylation of arenes with terminal alkynes. *Chemical Communications* **2020,** *56*, 1298-1301.
4. Li, Z.-L.; Wu, P.-Y.; Cai, C., Nickel-catalyzed regioselective C–H halogenation of electron-deficient arenes. *New Journal of Chemistry* **2019,** *43*, 3462-3468.
5. Cheng, H.-G.; Pu, M.; Kundu, G.; Schoenebeck, F., Selective Methylation of Amides, N-Heterocycles, Thiols, and Alcohols with Tetramethylammonium Fluoride. *Organic Letters* **2020,** *22*, 331-334.
6. Pintori, D. G.; Greaney, M. F., Oxidative C–H Homodimerization of Phenylacetamides. *Organic Letters* **2011,** *13*, 5713-5715.
7. Sokolovs, I.; Lubriks, D.; Suna, E., Copper-Catalyzed Intermolecular C–H Amination of (Hetero)arenes via Transient Unsymmetrical λ3-Iodanes. *Journal of the American Chemical Society* **2014,** *136*, 6920-6928.
8. Xia, Q.; Liu, X.; Zhang, Y.; Chen, C.; Chen, W., Copper-catalyzed N-methylation of amides and O-methylation of carboxylic acids by using peroxides as the methylating reagents. *Organic letters* **2013,** *15*, 3326-3329.
9. Ramachandran, P. V.; Hamann, H. J.; Choudhary, S., Amine-boranes as Dual-Purpose Reagents for Direct Amidation of Carboxylic Acids. *Organic Letters* **2020,** *22*, 8593-8597.
10. Li, B.; Wendlandt, A. E.; Stahl, S. S., Replacement of Stoichiometric DDQ with a Low Potential o-Quinone Catalyst Enabling Aerobic Dehydrogenation of Tertiary Indolines in Pharmaceutical Intermediates. *Organic Letters* **2019,** *21*, 1176-1181.
11. Chen, W.; Tang, H.; Wang, W.; Fu, Q.; Luo, J., Catalytic Aerobic Dehydrogenatin of N-Heterocycles by N-Hydoxyphthalimide. *Advanced Synthesis & Catalysis* **2020,** *362*, 3905-3911.
12. Zhang, M.; Yuan, X.-A.; Zhu, C.; Xie, J., Deoxygenative Deuteration of Carboxylic Acids with D2O. *Angewandte Chemie International Edition* **2019,** *58*, 312-316.
13. Tang, Y.; Yu, B., A Mild Heteroatom (O-, N-, and S-) Methylation Protocol Using Trimethyl Phosphate (TMP)–Ca(OH)2Combination. *Synthesis* **2022,** *54*, 2373-2390.
14. Kumar, Y.; Ila, H., Synthesis of Substituted Benzo[b]thiophenes via Base-Promoted Domino Condensation–Intramolecular C–S Bond Formation. *Organic Letters* **2021,** *23*, 1698-1702.
15. Mor, M.; Rivara, S.; Silva, C.; Bordi, F.; Plazzi, P. V.; Spadoni, G.; Diamantini, G.; Balsamini, C.; Tarzia, G.; Fraschini, F.; Lucini, V.; Nonno, R.; Stankov, B. M., Melatonin Receptor Ligands: Synthesis of New Melatonin Derivatives and Comprehensive Comparative Molecular Field Analysis (CoMFA) Study. *Journal of Medicinal Chemistry* **1998,** *41*, 3831-3844.
16. Tian, M.; Abdelrahman, A.; Weinhausen, S.; Hinz, S.; Weyer, S.; Dosa, S.; El-Tayeb, A.; Müller, C. E., Carbamazepine derivatives with P2X4 receptor-blocking activity. *Bioorganic & Medicinal Chemistry* **2014,** *22*, 1077-1088.
17. Paul, B.; Panja, D.; Kundu, S., Ruthenium-Catalyzed Synthesis of N-Methylated Amides using Methanol. *Organic Letters* **2019,** *21*, 5843-5847.
18. Ghosh, S. C.; Ngiam, J. S. Y.; Seayad, A. M.; Tuan, D. T.; Johannes, C. W.; Chen, A., Tandem oxidative amidation of benzyl alcohols with amine hydrochloride salts catalysed by iron nitrate. *Tetrahedron Letters* **2013,** *54*, 4922-4925.

19. Sakamoto, R.; Sakurai, S.; Maruoka, K., Bis(trialkylsilyl) peroxides as alkylating agents in the copper-catalyzed selective mono-N-alkylation of primary amides. *Chemical Communications* **2017,** *53*, 6484-6487.
20. Beak, P.; Musick, T. J.; Chen, C. W., Does formal intramolecular transfer of an acidic deuterium to a site of halogen-lithium exchange show that lithium-halogen exchange is faster than loss of the acidic deuterium? Evidence in favor of an alternative mechanism. *Journal of the American Chemical Society* **1988,** *110*, 3538-3542.
21. Alandini, N.; Buzzetti, L.; Favi, G.; Schulte, T.; Candish, L.; Collins, K. D.; Melchiorre, P., Amide Synthesis by Nickel/Photoredox-Catalyzed Direct Carbamoylation of (Hetero)Aryl Bromides. *Angewandte Chemie International Edition* **2020,** *59*, 5248-5253.
22. Cheng, H.-C.; Hou, W.-J.; Li, Z.-W.; Liu, M.-Y.; Guan, B.-T., The copper-catalyzed aerobic oxidative amidation of tertiary amines. *Chemical Communications* **2015,** *51*, 17596-17599.
23. Wang, T.; Yuan, L.; Zhao, Z.; Shao, A.; Gao, M.; Huang, Y.; Xiong, F.; Zhang, H.; Zhao, J., Direct oxidative amidation between methylarenes and amines in water. *Green Chemistry* **2015,** *17*, 2741-2744.
24. Ding, J.; Cao, L.; Wang, J.; Xue, W.; Zhu, Y.; Wu, A., The Conversion of Aryl and Heteroaryl Methylketones to the Corresponding Secondary or Tertiary Amides. *Journal of Chemical Research* **2011,** *35*, 298-301.
25. Wu, Y.; Peng, X.; Luo, B.; Wu, F.; Liu, B.; Song, F.; Huang, P.; Wen, S., Palladium catalyzed dual C–H functionalization of indoles with cyclic diaryliodoniums, an approach to ring fused carbazole derivatives. *Organic & Biomolecular Chemistry* **2014,** *12*, 9777-9780.
26. Dalkilic, O.; Lafzi, F.; Kilic, H.; Saracoglu, N., Novel triphenylamine-connected indolinium fluorescence sensor for detection of the cyanide anion and DFT calculations. *Tetrahedron Letters* **2020,** *61*, 152315.
27. Javed, T.; Shattat, G. F., A synthesis of potential new antiarrhythmic agents. *Journal of Heterocyclic Chemistry* **2005,** *42*, 217-220.
